# Supplementary material for: A scalable and cGMP-compatible autologous organotypic cell therapy for Dystrophic Epidermolysis Bullosa
Source: Nat Commun. 2024 Jul 11;15:5834. doi: 10.1038/s41467-024-49400-z (PMC11239819; doi:10.1038/s41467-024-49400-z)

SUPPLEMENTARY INFORMATION

A

sgRNA	activity score	off-target score
C1	67	95
C2	45	90
C3	40	91
C4	68	95
C5	50	98
C6	42	95

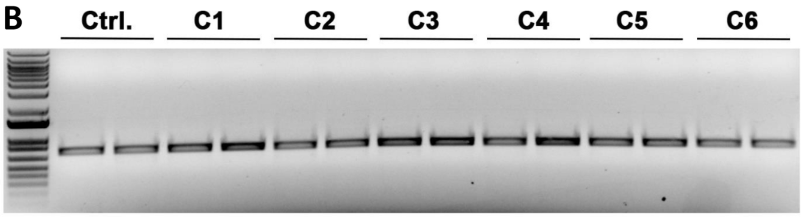

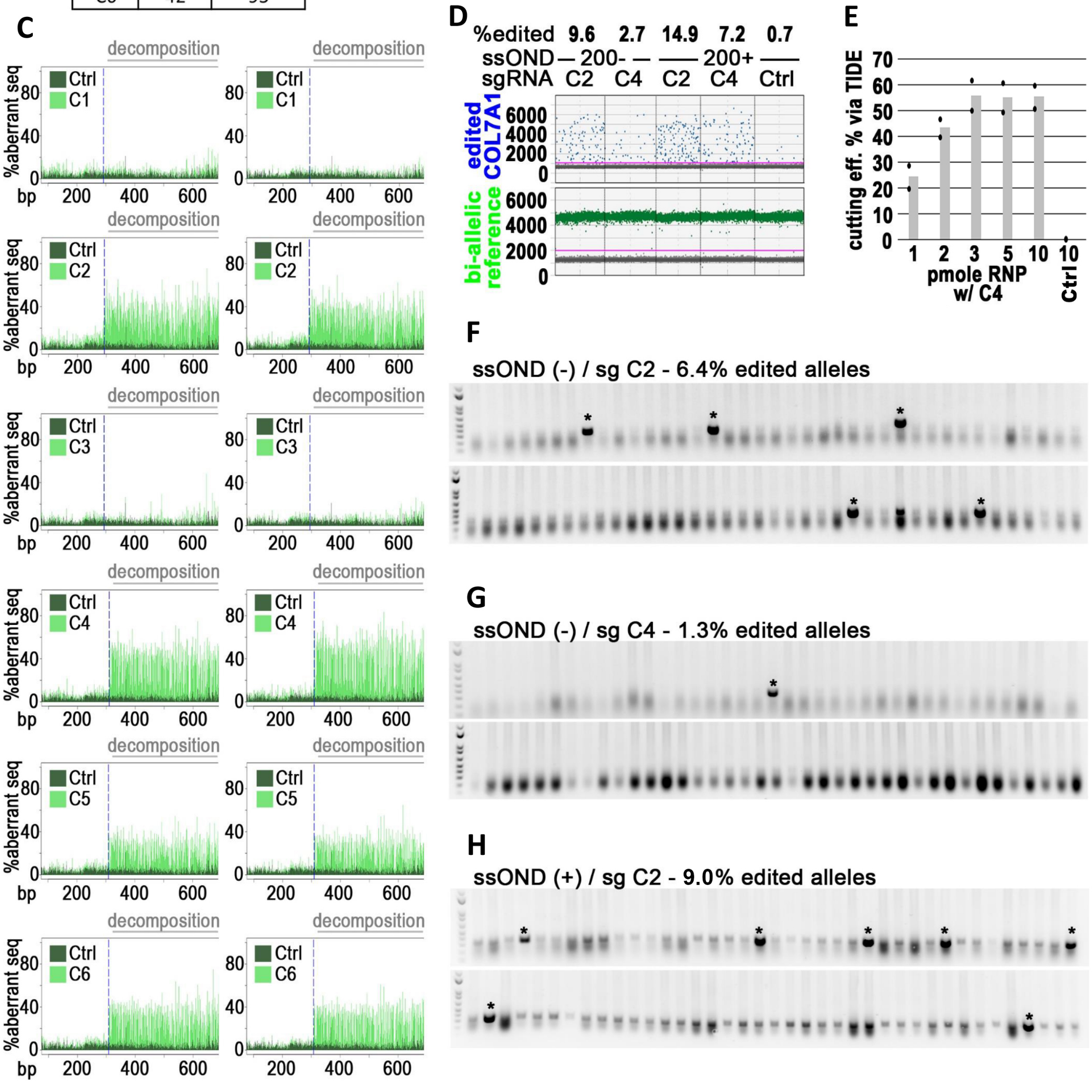

Supplementary Figure 1: Optimized editing of the Colorado mt (7485+5 G>A). (A) *in silico* predicted efficiency (middle column) and specificity (right column) score of the 6 tested sgRNAs (left column) used to mediate CAS9-cutting of the pathogenic Colorado allele. **(B)** Agarose gel visualizing PCR amplicons of a 731bp sequence surrounding the *COL7A1* target locus from homozygous CO2 patient fibroblasts transfected with RNPs containing CAS9 and indicated sgRNAs (Ctrl. omitted sgRNA). Two biological replicates are shown. A DNA size reference was run in most left lane (100bp-15000bp range shown). **(C)** TIDE traces of PCR amplicons shown in Supplementary Figure 1B. **(D)** *COL7A1* editing efficiencies as measured by ddPCR in DEB125 primary patient fibroblasts (het. Colorado mt) after transfection with ssODNs and sgRNA/CAS9-containing RNPs as indicated. A bi-allelic locus (green) is used as a reference for calculating *COL7A1* editing (blue) efficiencies. Ctrl omitted sgRNAs. **(E)** InDel formation in CO2 fibroblasts after transfection with indicated amounts of sgRNA C4-containing RNPs as measured by TIDE (n=2 biological replicates; mean is shown with individual data points overlayed as scatter plot; Ctrl. omitted sgRNA). **(F-H)** Agarose gels visualizing 78 E. coli colony-PCRs to detect edited TOPO-cloned *COL7A1* alleles from CO2 fibroblasts transfected with ssODNs and RNPs containing sgRNAs as indicated. A primer specific for silent mutations (see Figure 1B) only amplifies PCR products of alleles with integration of donor sequences (asterisks). DNA size references were run in most left lanes (100bp-2000bp range shown).

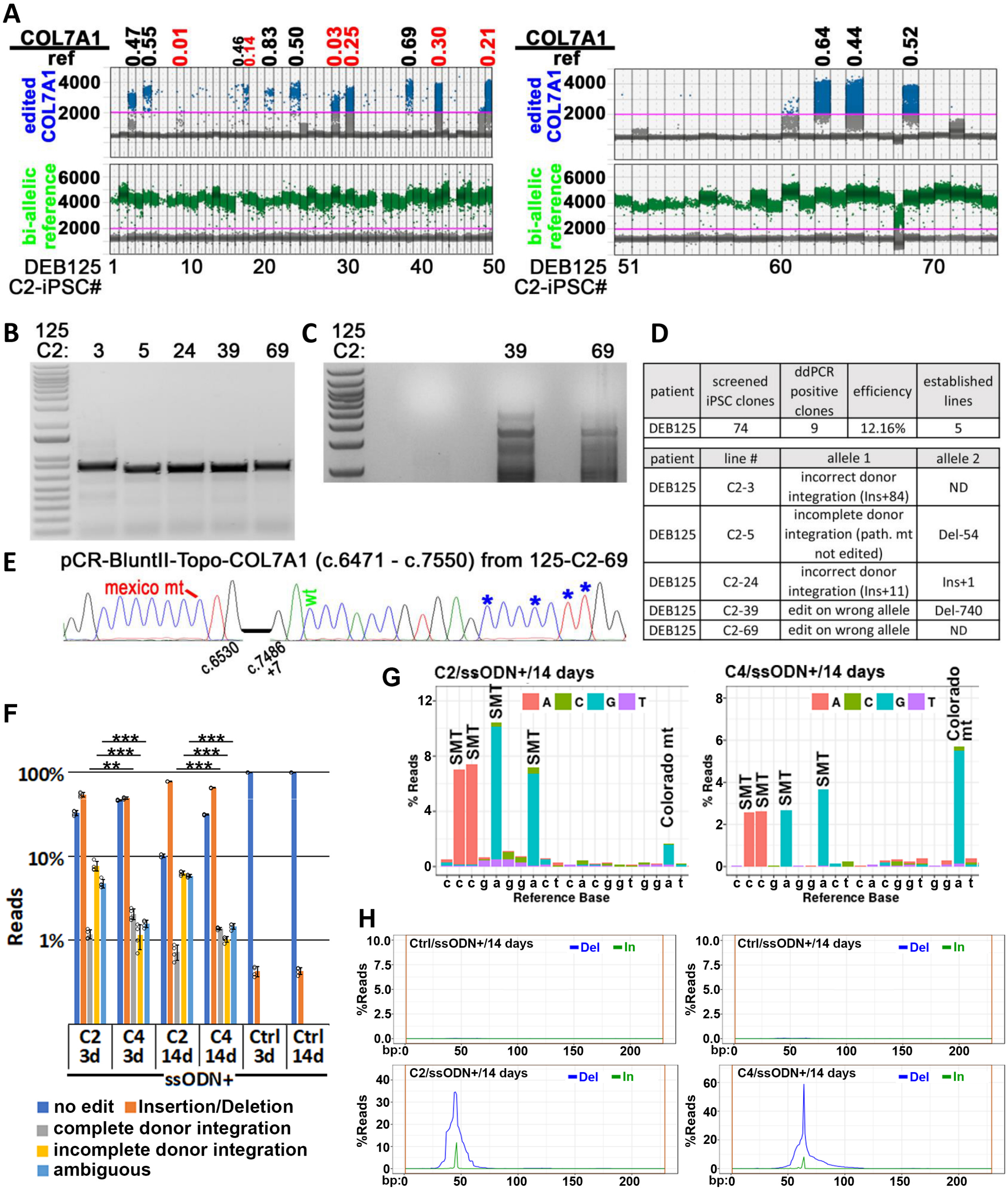

patient	screened iPSC clones	ddPCR positive clones	efficiency	established lines
DEB125	74	9	12.16%	5

patient	line #	allele 1	allele 2
DEB125	C2-3	incorrect donor integration (Ins+84)	ND
DEB125	C2-5	incomplete donor integration (path. mt not edited)	Del-54
DEB125	C2-24	incorrect donor integration (Ins+11)	Ins+1
DEB125	C2-39	edit on wrong allele	Del-740
DEB125	C2-69	edit on wrong allele	ND

Supplementary Figure 2: *COL7A1* repair with the less specific sgRNA C2. (A) ddPCR screening of 74 iPSC lines derived from DEB125 (sgRNA C2/ssODN+) identified 9 candidates. Ratios of signals detecting edited *COL7A1* alleles (blue) and a bi-allelic reference locus (green)

identify mono-allelic (0.5 +/-0.19) or bi-allelic (1.0 +/-0.19) editing events (black values; red values below/above cut off). **(B-C)** 5 of these candidate iPS cell lines were analyzed via agarose gels visualizing PCR amplicons of a 731bp (B) and 4560bp (C) sequence surrounding the edited *COL7A1* locus. (DNA size references in most left lanes). **(D-E)** Cloning and Sanger sequencing revealed correctly edited *COL7A1* in 2 of 5 candidates (D; top: summary of screen). *COL7A1* repair in lines 125-C2-39 and 125-C2-69 occurred, however, on the wrong allele carrying the second heterozygous compound mutation of this patient (E; 'Mexico mt', 6527dupC). This agrees with the low specificity of sgRNA C2. We note that sgRNA C2 is capable of targeting the disease allele as we have successfully used it to derive repaired cell lines from fibroblasts of homozygous patient CO2 (Figure 4A). **(F)** Analysis of next generation sequencing (>700k reads per technical replicate; n=4; mean and SD are shown) of PCR amplicons encompassing the Colorado locus from CO2 primary patient fibroblasts. Employed sgRNAs complexed with CAS9, ssODNs(+), and timepoints (d: days) after transfection are indicated. Compared to sgRNA C2, sgRNA C4-edited populations exhibit significantly (t test; $p<0.05^*$, $p<0.01^{**}$, $p<0.001^{***}$) more alleles with all designed edits encoded by ssODNs (see Figure 1B) incorporated (i.e. complete donor integration). Conversely, sgRNA C2-edited populations exhibit significantly more alleles on which only some, but not all of the designed ssODN-edits were incorporated, when compared to sgRNA C4 (i.e. incomplete and ambiguous donor integration); log10 scale. **(G)** Biological replicate of the 14d time point shown in (F) analyzed at single base pair resolution confirms less efficient repair of the Colorado locus in sgRNA C2 edited fibroblasts compared to sgRNA C4 edited populations (SMT: silent mutation). **(H)** Spectrum of insertions (In; green) and deletions (Del; blue) detected by next generation sequencing of PCR amplicons as in (G; 14d timepoint). sgRNAs as indicated and omitted in controls (Ctrl).

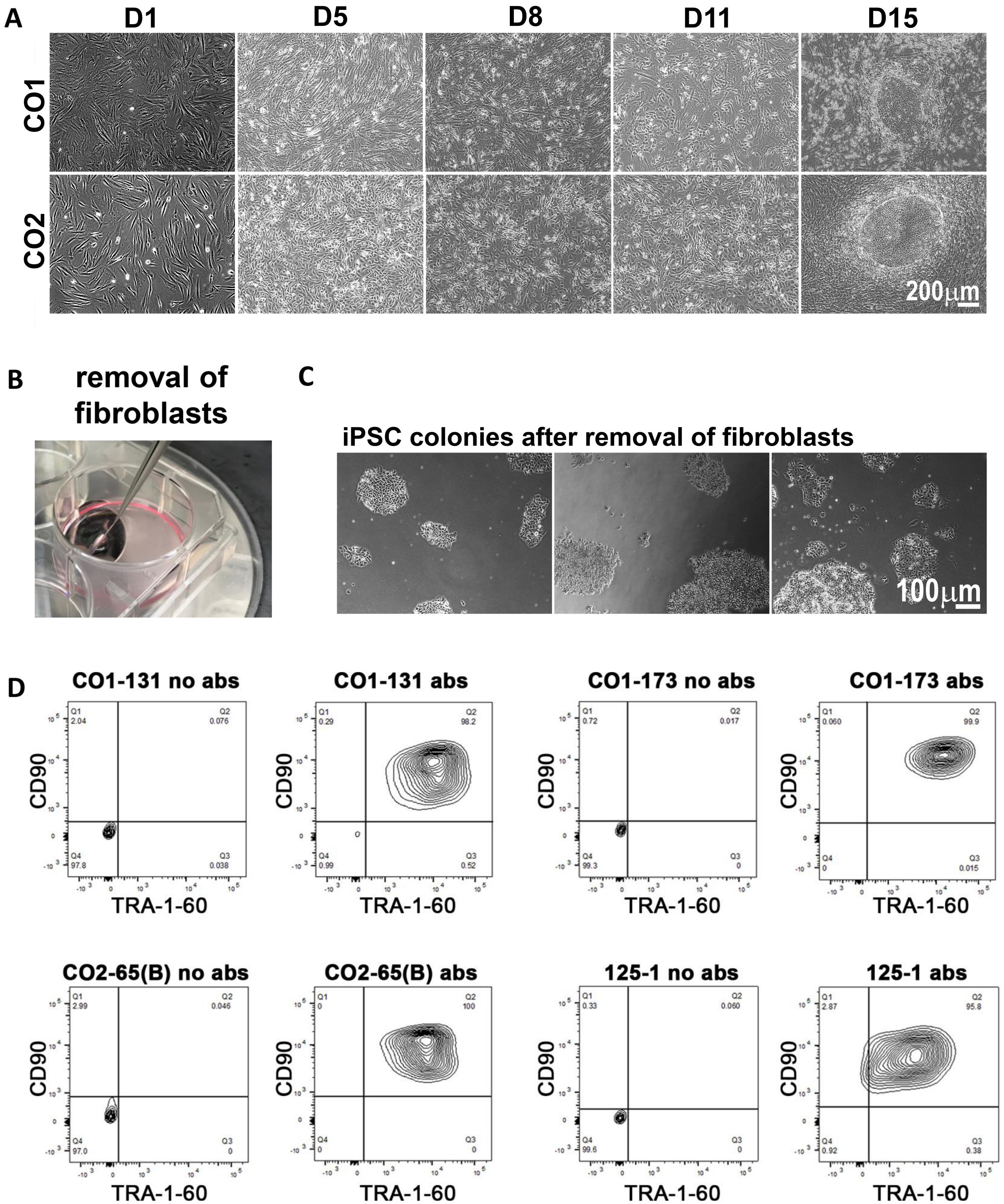

Supplementary Figure 3: Efficient derivation of iPS cells from primary RDEB patient fibroblasts. (A) Phase contrast microscopy pictures of cell cultures during single-step editing/reprogramming from time points (D: day) and patients as indicated. iPS cell colonies

emerge around D11-14 (line dependent). Scale as indicated. **(B)** Non-reprogrammed cells grow to confluency, adhere together, overlay iPS cell colonies and can be mechanically removed. **(C)** iPS cell colonies remain after removal of non-reprogrammed cells. Scale as indicated. **(D)** Flow cytometry analysis of single-step edited/reprogrammed iPS cell lines with and without labeling (antibodies; abs) of the pluripotency surface marker TRA-1-60 as indicated.

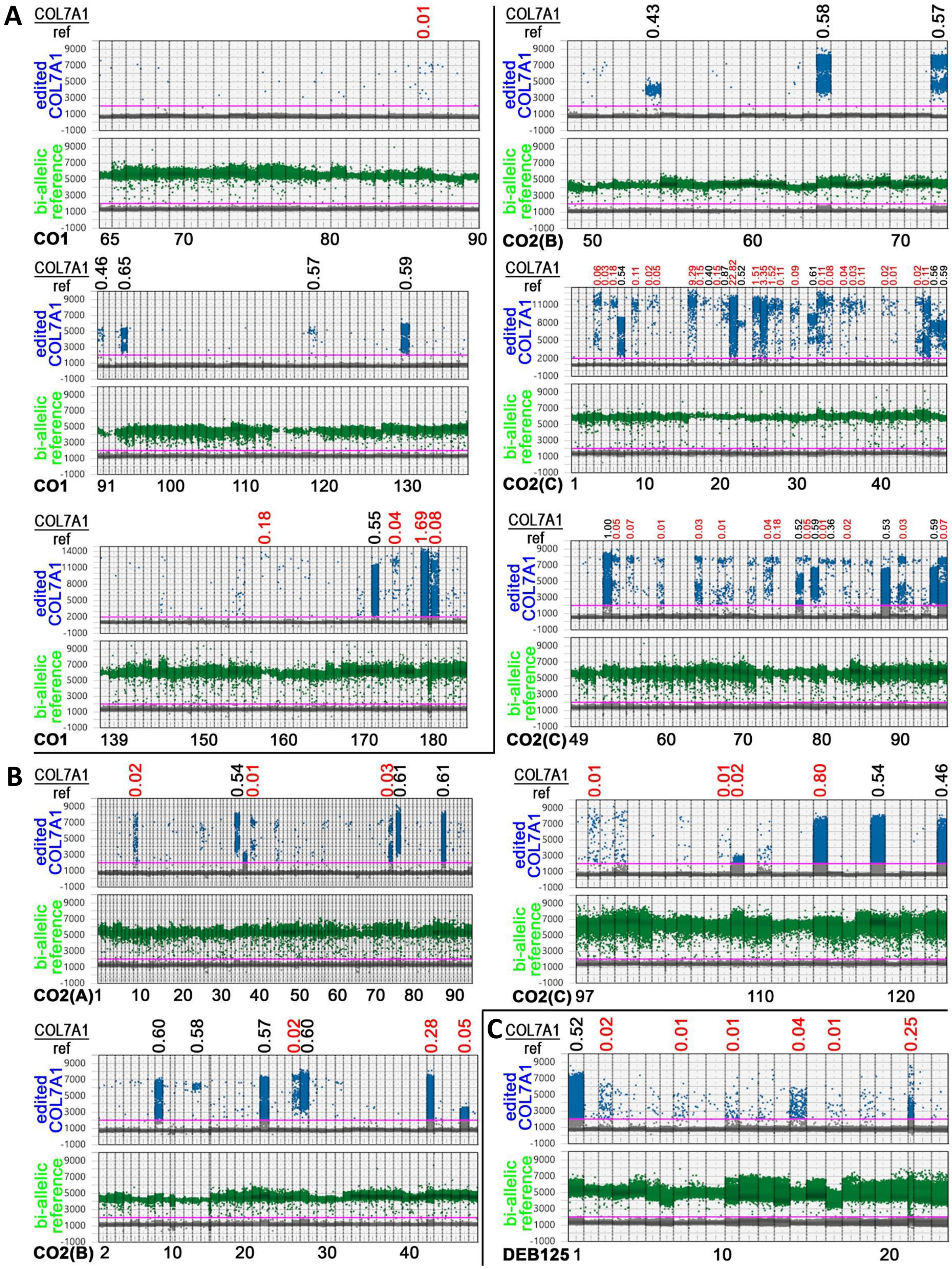
A
B
C
COL7A1
ref
edited COL7A1
bi-allelic reference
CO1
CO2(A)
CO2(B)
CO2(C)
DEB125

61

Supplementary Figure 4: ddPCR screening of single-step edited/reprogrammed iPS cells. (A-C) Screen of 122 (A), 293 (B), and 24 (C) single-step edited/reprogrammed iPS cell lines via ddPCR from patients as indicated. Ratios of signals detecting edited *COL7A1* alleles (blue) and a bi-allelic reference locus (green) are used to identify mono-allelic (0.5 +/-0.19) or bi-allelic (1.0 +/-0.19) editing events (black values; red values below/above cut off indicate potentially mixed or incorrectly edited clones).

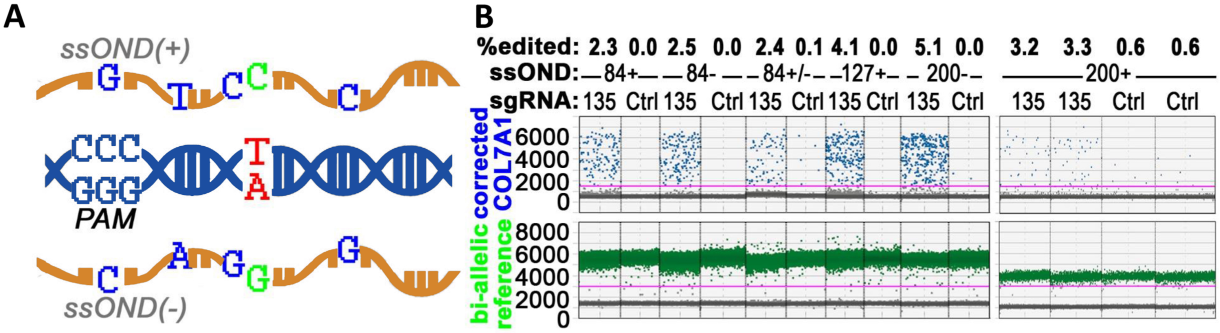

C

patient	mutation	ssOND length	edited cells by ddPCR
DEB135	6781 C>T	84+	2.3%
		84-	2.5%
		84+/-	2.4%
		127+	4.1%
		200+	3.3%
		200-	5.1%

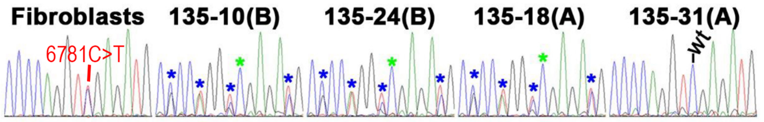

G

patient	screened iPSC clones	ddPCR positive clones	efficiency	established lines
DEB135	83	4	4.82%	4

patient	line #	allele 1	allele 2	# of sequenced alleles (#1/#2)
DEB135	18(A)	correct edit	wt	22 (12/10)
DEB135	31(A)	incorrect edit	wt	15 (0/15)
DEB135	10(B)	correct edit	wt	28 (11/17)
DEB135	24(B)	correct edit	wt	18(6/12)

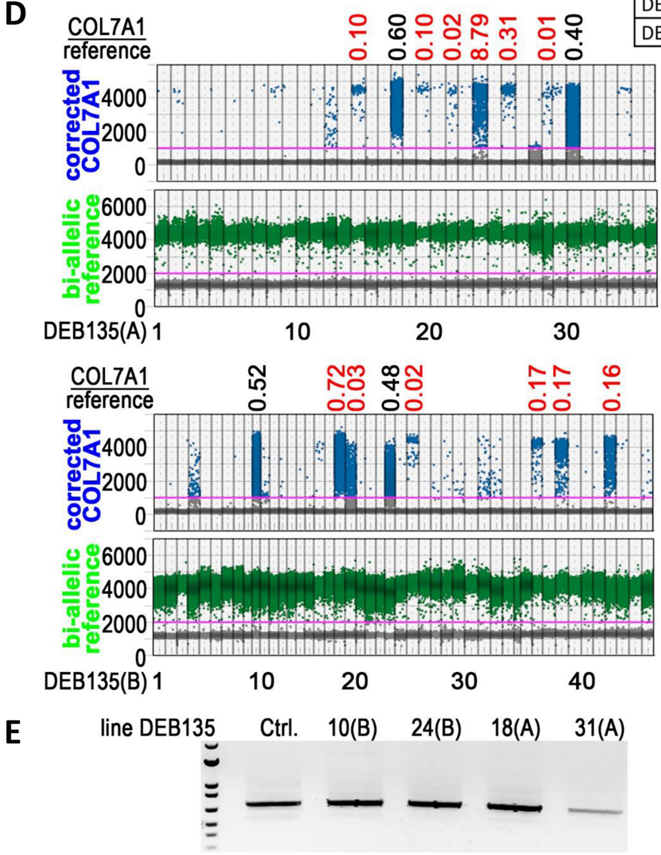

H

Patient fibroblasts STAMP target	iPSC line STAMP target	iKC STAMP target
**DEB135** NONE	**DEB135-10(B)** NONE	**DEB135-10(B)** NONE
	**DEB135-24(B)** NONE	**DEB135-24(B)** NONE

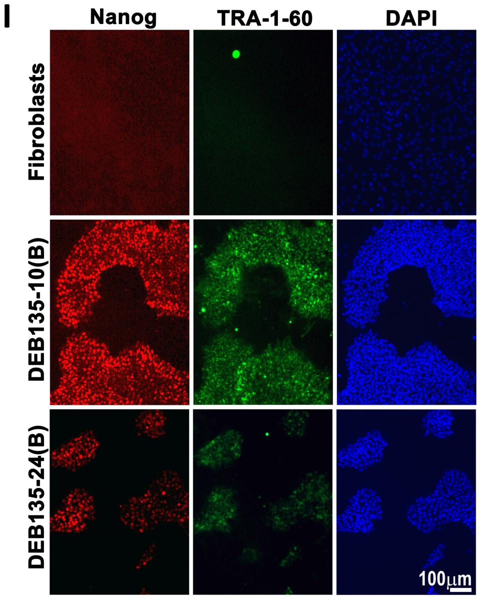

Supplementary Figure 5: Optimized single manufacturing step editing/reprogramming of patient DEB135 (6781C>T). (A) Overview of the *COL7A1* target mutation 6781 C>T (red) and

ssODNs used for editing. PAM site to mediate cutting of the pathogenic allele via CRISPR/CAS9 is shown. ssODNs: wild type sequence (green); 4 silent mutations used for detection of editing events (blue). **(B-C)** *COL7A1* 6781C>T editing efficiencies as measured by ddPCR in DEB135 primary patient fibroblasts after transfection with ssODNs of various lengths, orientation (+/-), and sgRNA 135/CAS9-containing RNPs as indicated. A bi-allelic locus (green) is used as a reference for calculating *COL7A1* editing (blue) efficiencies, assuming mono-allelic integration of donor sequence. The length of ssODNs correlates with editing efficiencies. Ctrls omitted sgRNAs. **(D)** Screen of 83 iPS cell lines via ddPCR after single-step editing/reprogramming of fibroblasts from patient DEB135 with ssODN(-) and sgRNA 135. Ratios of signals detecting edited *COL7A1* alleles (blue) and a bi-allelic reference locus (green) are used to identify mono-allelic (0.5 +/-0.19) or bi-allelic (1.0 +/-0.19) edited iPS cell lines (black values; red values below/above cut off indicate potentially mixed or incorrectly edited clones). **(E)** Agarose gel visualizing PCR amplicons of a 696bp sequence surrounding the edited *COL7A1* locus from established iPS cell lines from Supplementary Figure 5D. Unedited fibroblasts were used as Ctrl. DNA size reference in most left lane. **(F)** Sanger sequencing traces of PCR amplicons shown in Supplementary Figure 5E containing both *COL7A1* alleles. Unedited fibroblasts show the heterozygous 6781C>T mutation as a double peak. Correctly edited iPS cell lines show heterozygous integration of intended silent mutations (blue asterisks) and repair of the pathogenic mutation (green asterisks). iPS cell line 135-31(A) only displayed the wild type sequence, indicative of a large InDel prohibiting amplification of the target allele. **(G)** Summary of single-step editing/reprogramming screen of patient DEB135 (top). Sanger sequencing of individual Topo-cloned alleles (Supplementary Figure 5E) confirmed correct editing in 3 iPS cell lines. See text for details. **(H)** STAMPv2 oncopanel sequencing of indicated cell lines did not identify any variants. **(I)** Immuno-fluorescence microscopy images of DEB135 fibroblasts and thereof derived iPS cells. NANOG (red), TRA-1-60 (green), and DNA (blue). Scale as indicated.

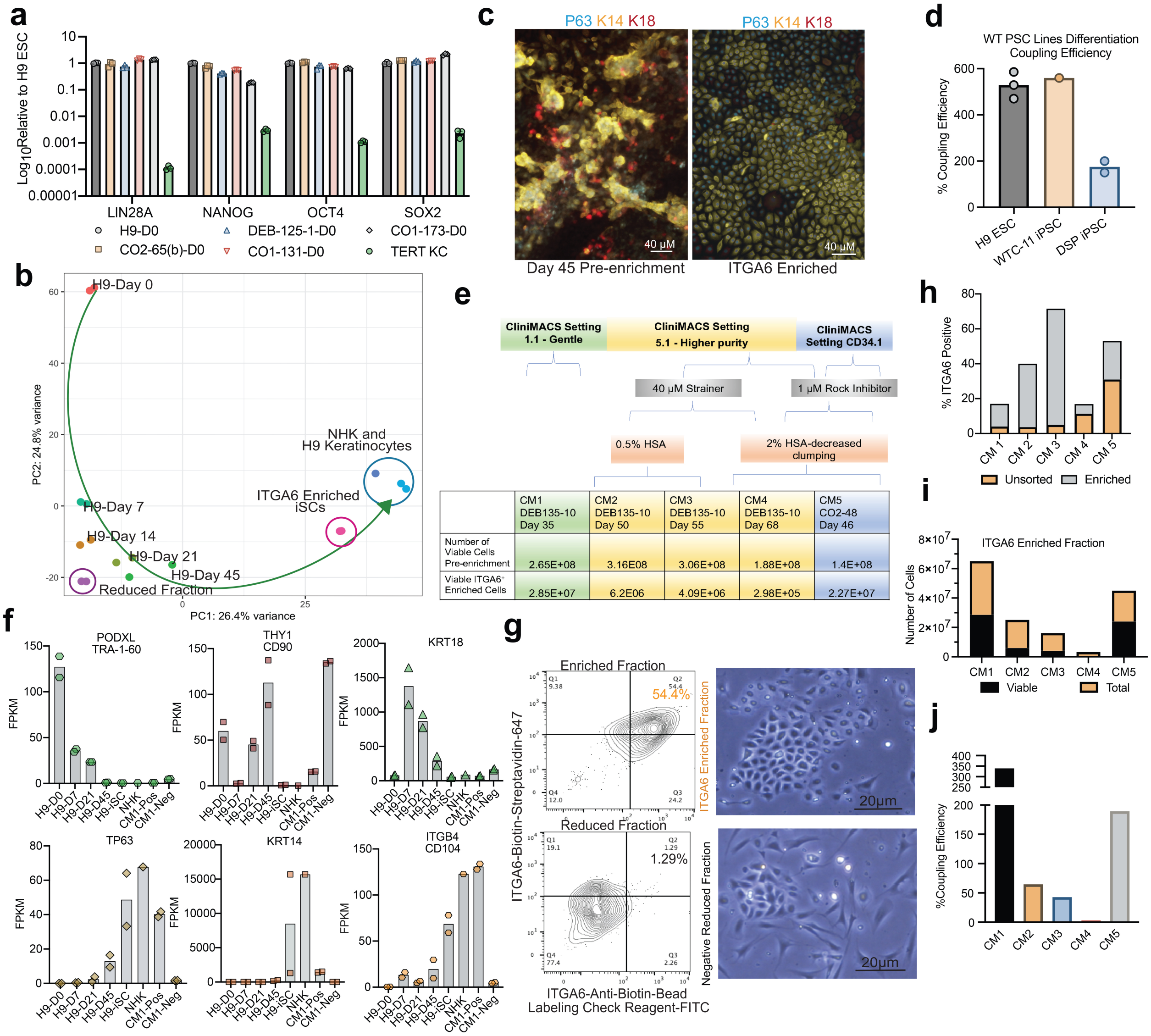

Supplementary Figure 6: iSCs enrichment for clinical scale manufacturing. (A) qRT-PCR of pluripotency marker expression of iPS and ES cell lines. TERT keratinocytes (KC) were used as the negative control (n= 3 technical replicates; mean and SEM are shown). Note log scale. **(B)** Principal component analysis (PCA) of RNA-seq from CliniMACS Plus ITGA6-enriched and reduced iSC cells compared to a H9-ES cells differentiation time course (D0-D45); H9 keratinocytes (KC) n = 2 biologically independent samples, and NHK (positive control, n=1), n =2 for CliniMACS (CM) positive and negative samples. **(C)** Immunofluorescent images of AutoMACS-ITGA6 enriched (and pre-enriched, left), DEB135-10 iSCs; p63 (Cyan), K18 (red),

and K14 (yellow). **(D)** % Coupling efficiency (CE) of H9 ES- and iPS cell- derived iSCs determined by the equation (6) %CE= live sorted iSCs/iPS cell input x 100. Note line-to-line variability between used iPS cells, both without genetic pathogenicity (see text for details). H9 n=3 independent biological replicates, WTC-11 n=1 and, DSP n=2. **(E)** Table of optimizations for 5 CliniMACS Plus-mediated ITGA6 enrichments, including program and reagent modifications for each run with pre- and post-enrichment cell counts. **(F)** Expression of CliniMACS Plus ITGA6-enriched and reduced populations analyzed via RNAseq, compared to H9 differentiation time course, H9 iSCs, and NHKs, illustrating the non-target population markers (TRA-1-60, Thy1/CD90, KRT18) and the target population markers (p63, KRT14 and ITGB4). **(G)** Flow cytometry plots and corresponding bright field images of ITGA6 enriched and reduced Fractions from CM3 CliniMACS Plus separation (10x Magnification, scale as indicated). **(H)** Flow cytometry analysis for % ITGA6 positive cells of CM-sorted populations compared to the unsorted (pre-enriched) population. **(I)** Number of both viable and non-viable CliniMACS Plus-enriched iSCs. **(J)** %Coupling efficiency (CE) for CliniMACS Plus runs CM 1-5.

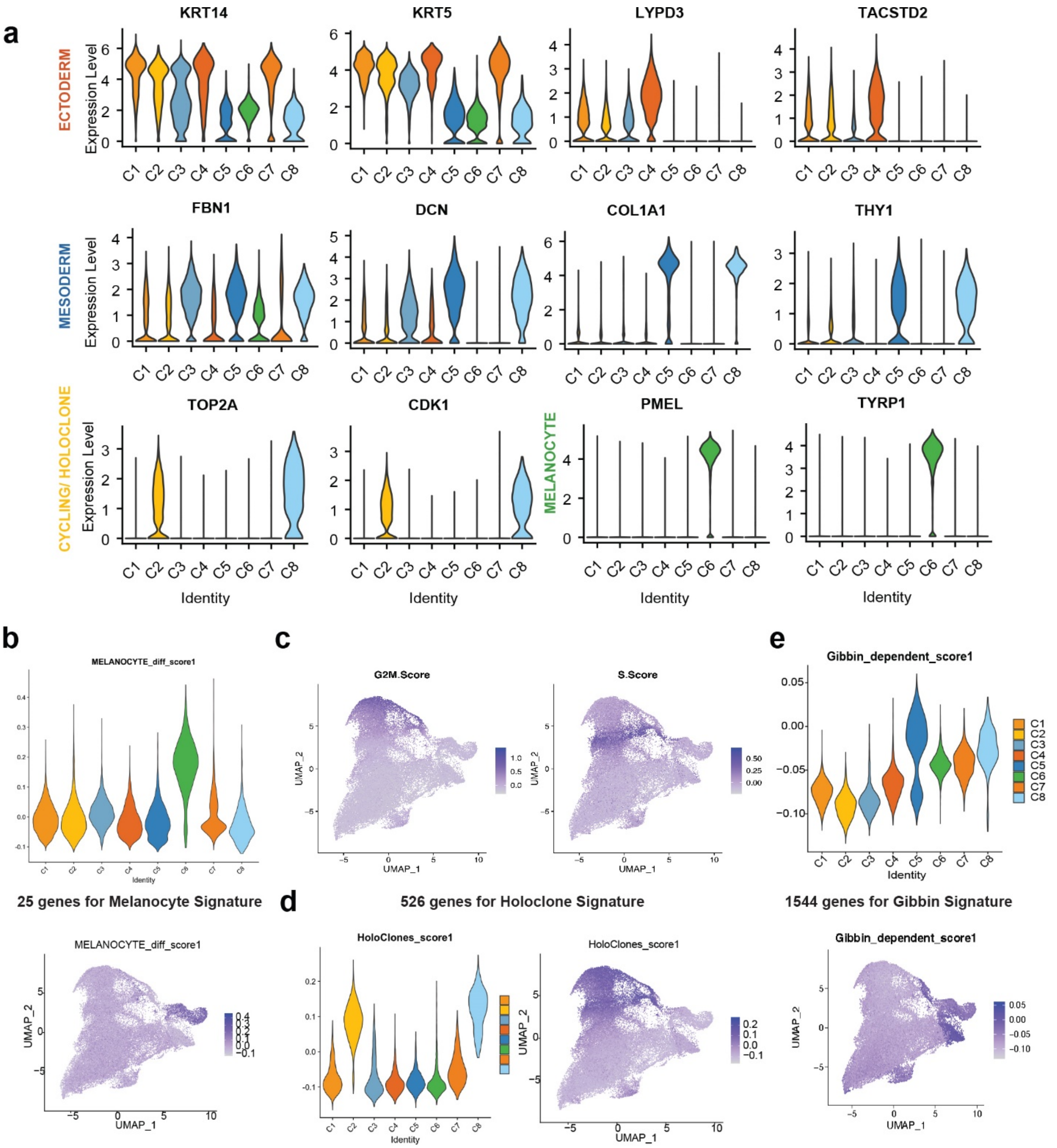

Supplementary Figure 7: Analysis of iSC populations using scRNA-seq and canonical markers. (A) Violin plots of relative expression levels of indicated genes, i.e. markers for basal keratinocytes (C1), cycling holoclones (C2), early differentiating keratinocytes (C4), mesoderm (C5, C8), pre-vascular mesoderm (C3), and melanocytes (C6), define each cell cluster contained

in the iSC product (see also Figure 3). **(B)** Violin plot and Uniform manifold approximation projection (UMAP) illustrate the melanocyte population identified by a signature of 25 genes expressed in the C6 cluster. **(C)** UMAP illustrates clusters of cycling cells (i.e. C2 and C8) via G2/M and S phase expressed gene sets. **(D)** Violin and UMAP plots show the overlap of cycling cells with the keratinocyte stem cell/holoclone-like population (C2) using an enrichment score based on a 526 gene set. **(E)** A signature of 1544 genes defining the Gibbin-dependent mesoderm is expressed in the C5/C8 clusters, as shown by violin and UMAP plots. See material and methods and Supplementary Data 1 for references and used gene-expression sets.

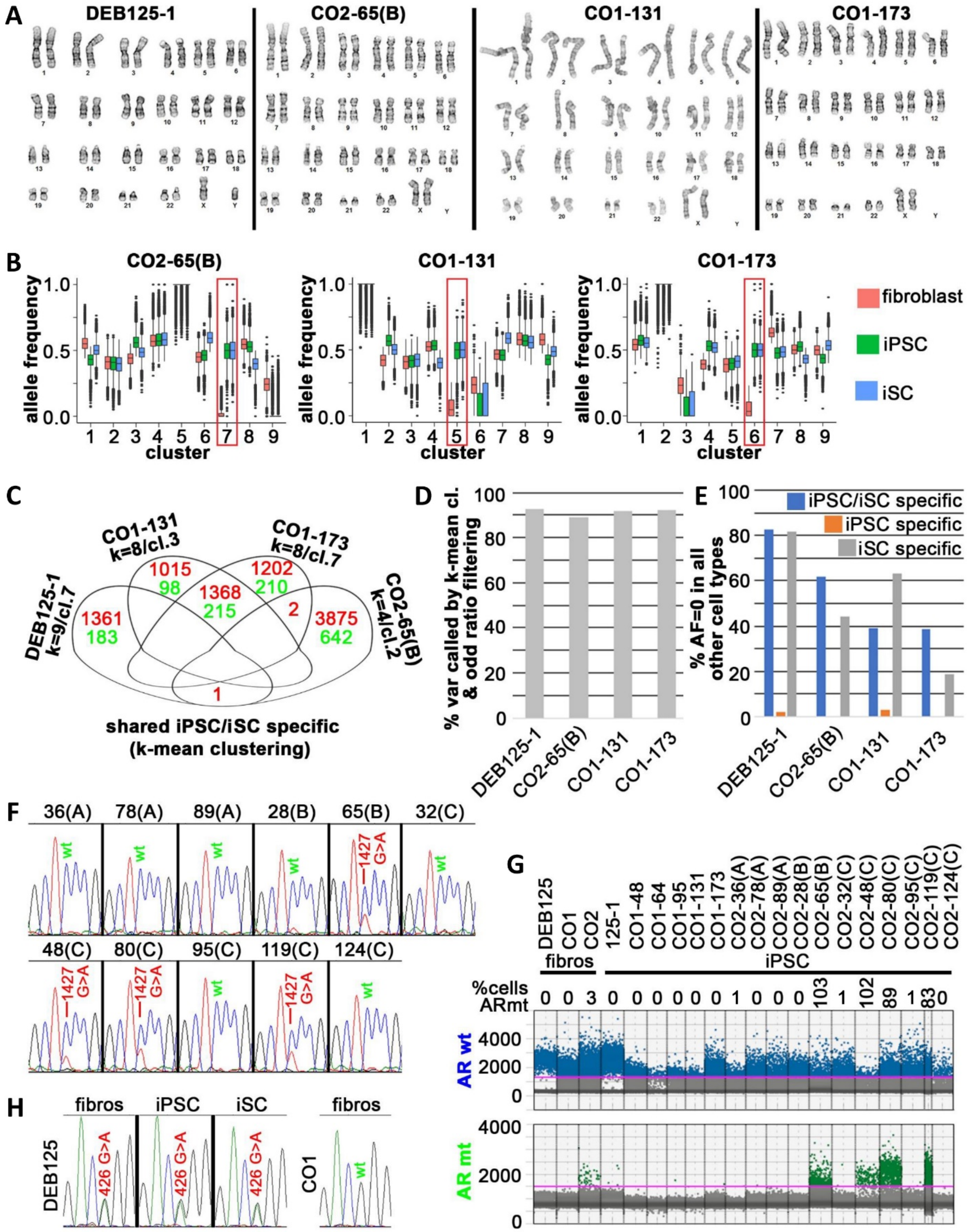

Supplementary Figure 8: Genomic and chromosomal stability of single step edited/reprogrammed iPS cells and iSCs. (A) Representative normal karyotypes of four single-

step edited/reprogrammed iPS cell lines from 3 individuals. **(B)** K-means clustering of all variants found by 40x whole genome sequencing (WGS) in fibroblasts and thereof derived iPS and iSC cells from patients CO1 and CO2 (CO2-65(B) n=114594; CO1-131 n=102278; CO1-173 n=101915; see Supplementary Data 2). The red frame highlights differentially expressed allele frequencies (AFs) of variants in iPS/iSC cells compared to parental fibroblasts. Box plot shows interquartile ranges of data (25%-75%, boxes), with medians (center lines) and whiskers extending to 1.5 times the interquartile range; outliers as circles. **(C)** Variants (red: SNPs; green: InDels) that are specifically found in iPS and iSC cells of indicated cell lines as identified by k-means clustering. Grouping of variants in Venn diagrams indicates virtually no selection for mutations. Variants of clusters (cl.) showing clear separation between AFs found in fibroblasts and AFs found in iPS/iSC cells from feature spaces with the lowest k (indicated) were selected for this analysis. **(D)** AF cut-off filtering and k-means clustering identify virtually the same (>89%) variants specific to iPS/iSC cells. **(E)** Percentage of identified cell type-specific variants with no reads in other cell types as indicated. Note that virtually all iPS cell-specific variants identified by AF cut-off filtering (Figure 4C) were also found at lower AFs in other cell types. **(F)** Sanger sequencing traces of a PCR amplicon containing the androgen receptor (*AR*) locus c.1427 from indicated iPS cell lines of patient CO2. A heterozygous mutation was found in 4 of 11 iPS cell lines (double peaks). **(G)** A competitive ddPCR assay with probes specific for the wt and mt *AR* sequence from indicated cell lines confirms that 4 lines derived from patient CO2 harbor the mutation. Note that ddPCR detects this mt*AR* also in CO2 fibroblasts at low frequencies. **(H)** Sanger sequencing traces of a PCR amplicon containing the *CDKN1B* locus c.426 confirm a heterozygous germline mt (double peak) present in fibroblasts and thereof derived iPS/iSC cells of patient DEB125. Fibroblasts of patient CO1 were used as a negative control.

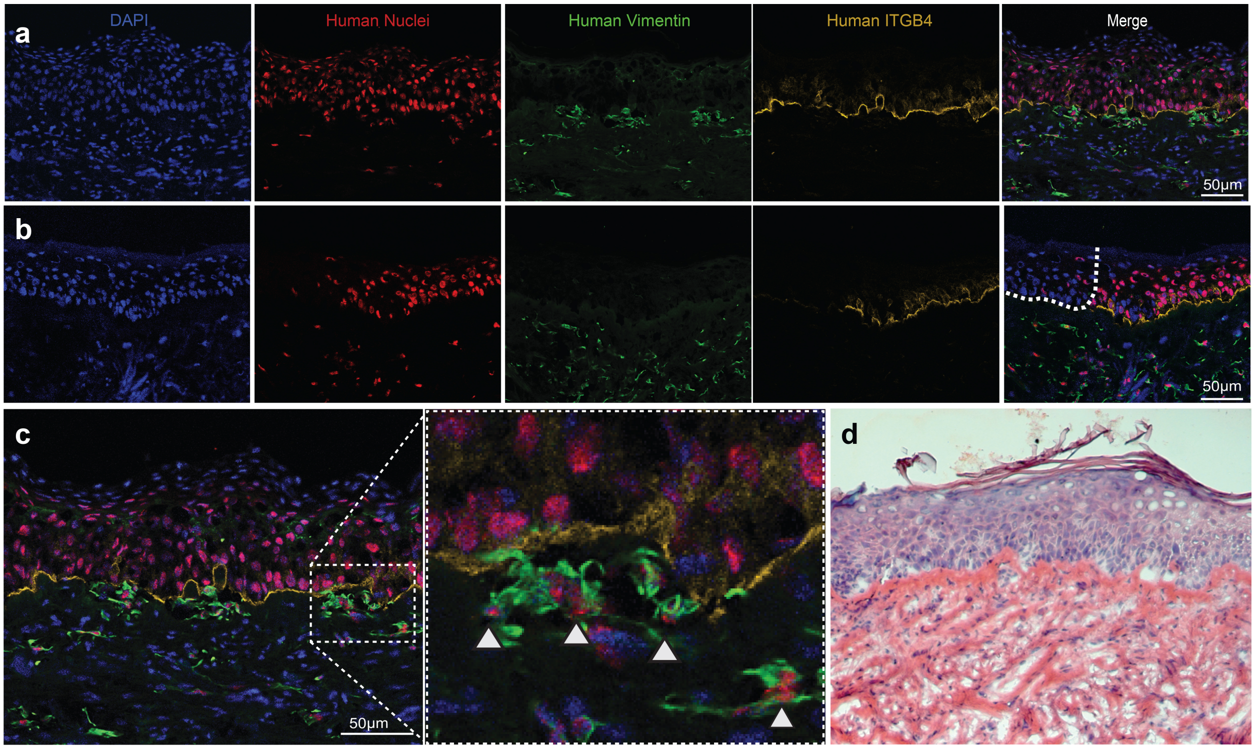

Supplementary Figure 9: Human fibroblasts in the dermal layer of iSCs grafted onto mice. **(A)** Immunofluorescence staining of iSC graft sites positive for human specific markers, i.e. human nuclei (red), human vimentin (green) and human ITGB4 (yellow). DAPI (blue); Scale as indicated. **(B)** Immunofluorescence staining as in (A) from graft border depicted by the dotted line in the merged image. Note mouse cells (negative for above human-specific markers) infiltrating the edge of graft. **(C)** Increased magnification of immunofluorescence staining from (A), merged image, demonstrates that the mesoderm marker vimentin (green) is specific to the human nuclei stain (red), as highlighted by white arrows. **(D)** Corresponding H&E histology of graft site displaying the dermis and stratified epidermis.

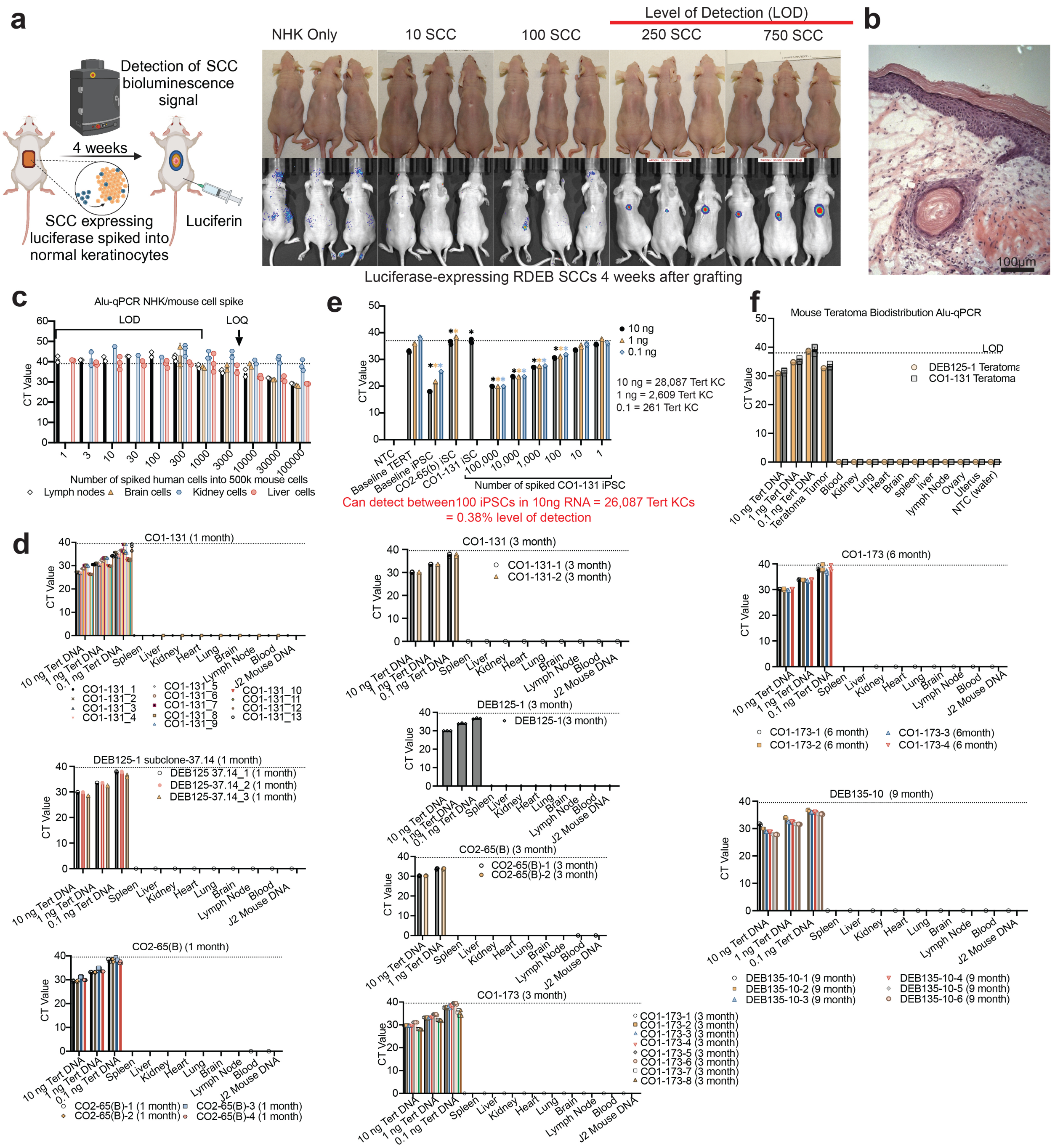

Supplementary Figure 10: Biodistribution and tumorigenicity. (A) Schematic of the bioluminescent assay to detect luciferase expressing RDEB-squamous cell carcinoma (SCC)-cells in iSC grafts at 4-weeks. Bioluminescent detection of NHKs and 10, 100, 250 and 750 RDEB patient-derived SCC cells in 4-week-old grafts on mice determines a level of detection (LOD) as indicated. **(B)** Histology (H&E stain) of graft site from positive sample from (A). **(C)** Alu-qPCR

detecting TERT-human keratinocytes in mouse cells as indicated to determine the level of detection (LOD) and quantification (LOQ) for human DNA (n=3 technical replicates; mean with SD). **(D)** Alu-qPCR experiments performed on organs and blood of iSC grafted mice at 1-9 months as indicated. At the left of each graph are the positive controls. Tissue sampled as indicated on the x axis (n=3 technical replicates; mean and SEM). **(E)** qRT-PCR of *LIN28A* to detect remnant iPS cells in expanded ITGA6-enriched iSC cultures. LOD was determined via spike in experiments as indicated; (n=3 technical replicates; mean and SD; Student's t test was used to determine the significance of differences with the annotations: * $p < 0.05$). **(F)** Mouse teratoma biodistribution assay with indicated iPS cell lines. Human specific Alu-qPCR did not detect any metastasizing cells. Human TERT keratinocytes and dissected iPS cell-induced teratomas were used as positive controls. Sampled tissues as indicated (n=3 technical replicates; mean and SEM; LOD: level of detection). Supplementary Figure 10/panel (a) created with BioRender.com released under a Creative Commons Attribution-NonCommercial-NoDerivs 4.0 International license.

Supplementary Data 1

Lists of used gene expression sets to define Gibbin-dependent mesoderm-like, holoclone-like and melanocyte-like cell clusters contained in the iSCs (also see Figure 3, Supplementary Figure 7).

Supplementary Data 2

List of variants represented by k-means clustering (see Figure 4B and Supplementary Figure 8B).

Supplementary Data 3

Plots of whole genome sequencing coverage 1kbp and 1Mbp up-/downstream of 57 *in silico*

predicted exonic, intronic, and intergenic off-targets for sgRNA C4 from fibroblasts and thereof derived iPS cells and iSCs.

Supplementary Table 1

target	primer 1	primer 2	size
Colorado mt allele (Figures 1C-D, 2D, 4E&G; Supplementary Figures 1B-D, 2B)	TAGGTGGGACAAGTGCTGCTGACTC	CCTTTAGTCCTGCACTCCCAACATCAC	731bp
Colorado mt allele big (Figure 2E)	TCATGTCTGAGCTCCTGTGAGCC	TCCACAGACTGGCTCATTTCTCACC	2418bp
Colorado/Mexico mt allele (Supplementary Figure 2C)	GCGTGGTATGGCTGGGCCTGAAG	CCTTTAGTCCTGCACTCCCAACATCAC	4560bp
DEB135 allele (Supplementary Figure 5E&F)	CTGACTGGACCTACTGGAGCTGTG	CCTGTGGGGAATGCTAGTGAGTTTCC	696bp
E. coli colony PCR (Figure 1F; Supplementary Figure 1F-H)	GGACAAGTGCTGCTGACTCT	CACCGTGAGCCCCCTT	327bp
AR (Supplementary Figure 8F)	CTCGCATCAAGCTGGAGAAC	ACACATCAGGTGCGGTGAAG	354bp
CDKN1B (Supplementary Figure 8H)	CTTGGAGAAGCACTGCAGAGAC	CGAAAAGCAAGCTAAGGTTAACACC	426bp
in silico predicted off-targets (Figure 4E&G)	primer 1	primer 2	size
NOTCH1	GTGTGACGCAGCCTGTGGGTGC	AAGCGCACCAGTTCTTCAGGACAGAC	648bp
AC144450.2	GAGCTTGCAGTGAGCAGAGATCG	CCTCTTCCCTGACTTCCTACACTG	727bp
PAK4	CAGAGAAGAGGCCCAAGTCTTCC	GCACCTCGTTGAAGAGCAGCTC	835bp
CAPN12	GCATTGAGTTCTTCCTCCTAGTCC	GTAATTTGCAGGGAGATCGACGAC	702bp
FXYD1/CTD-2527I21.4	GAGGAAACTAAGGCACAGGGAGG	CCTTCAACTCTGGCACTTGGCC	763bp
HS3ST4	CGTGACCAGGGCCATCTCTG	CCAGATGCTGCTGCCCAGATGG	723bp
MARS	GGAATAGGGCAGAGCCTTGGG	GAGATCAGCAAACGGGACCTAGC	357bp
PAQR7/RP1-125I3.2	CCCACATAGTCCAGGAAGAAGAAGC	CCTCTCATGTGTTTCACATGGCCAG	829bp
RP11-334A14.8/SLC1A7	CTGGGCTTTCTGCCTACCAGTG	CCATTTCCTCTGGCTGCACAATCC	773bp
RP5-1159O4.1/MIOS	CGACAGAGTGGTTCTGAGAAGC	GGGTACAACGGGAACAGGGCA	746bp
SLC25A29	AGCACTGGTGTCCCATCTGCAG	GTTTGTTACTCAGCAGATGCCAGCTG	751BP
UBE2I	CAGTCCCTCACCACACACAC	AGGTCCTGGGAATCTGCTTT	719BP
ZNF385A	GCATCCTAGTTTCCAGCTTCGTCC	CCCAGAAGGATGTCGGAGTATC	767bp
FAM207A	CAGAGTCAAGGGTTAGAGG	GACAGGCTTCTTCCTCTGTGCCCT	705bp
LINC00710	CAGAGCAGCCAGTCCAAAGACC	GGCCACACTGATAAGGTGGAGAC	776bp
OLFM1	CCATGGATCCCCTAATCCAAATGCC	TGAGAGGAACAACGCCTTCCTGG	760bp
PADI3	CGTGATAAGAGTGCAGAGGCTGG	GCAGCTCCCTCCACTCTTACAAG	825bp
PLXNA1	GTCATGGACTGCCCAACTCAGC	GCACGGGTCTAGAATGTTCCACTG	714bp
PRR26	CGGAGATTCTAGCCCTTGTCCCTG	CCAACACTGTGCGTGTTGACTCAG	746bp
RBM10	CTGAGCTCAAGCAGTCCTCACG	CCATTTACTCGGCAGGGACAGTC	558bp
TIMM44	CAGCCTCCTCAGAAAACAGCCTCG	GTCCTGTGAGGACTGCTAGAGGC	741bp
UST	GTCCAGGGCACCTGTAAGTAAGCC	GGGTGTCAATGCACGACATTCTCCC	766bp
VWA3A	TAGGCAGGAGGTCCCAAACAGC	GCTAGTCCTTCTCAAGCCCCTTC	653bp
VWF	TAGTCACTGGCTGGCTGGGTGTG	ACAGCATTCCTGGACTCTGCAGCC	709bp
ZBED4	GGCTCCTGCTGGAATTTGTGGCAG	GGGCCAGTGAGCAAAGTCGCATC	744bp
ZNF534	CTGAAATGCCAGGCATTGGAGTTGC	GCAGCAGATCACAGGTGTCTGAGC	868bp

Source Data – uncropped gels:

Supplementary Figure 1B

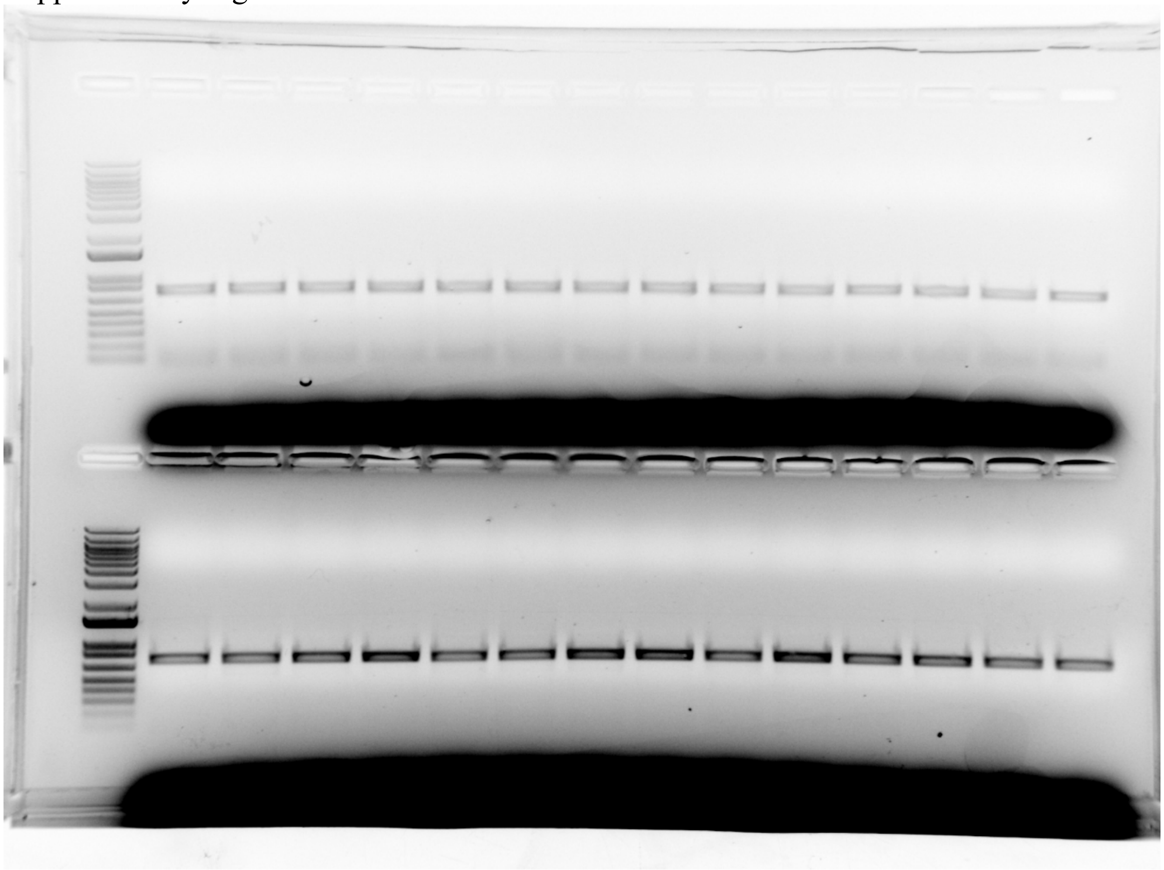

Supplementary Figure 1F

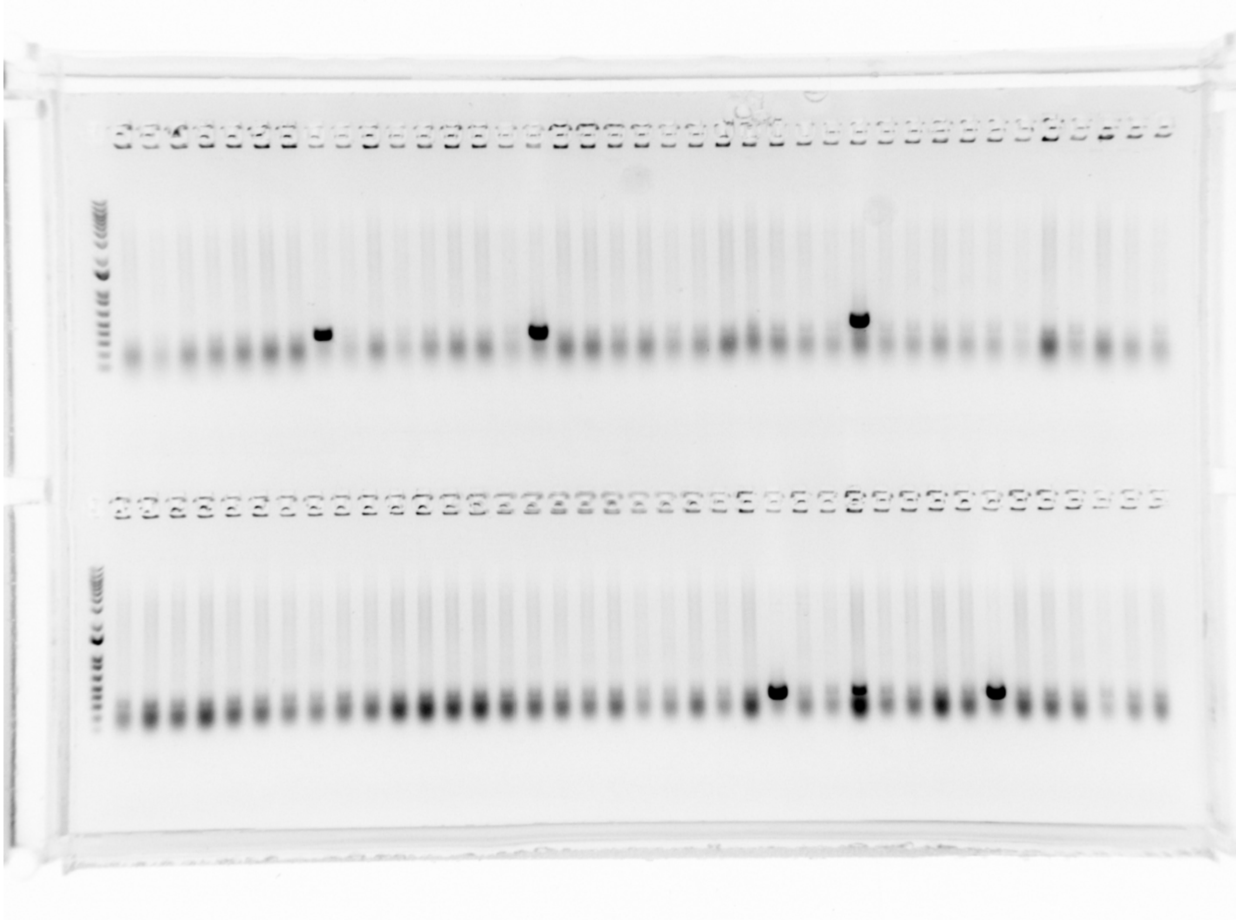

Supplementary Figure 1G

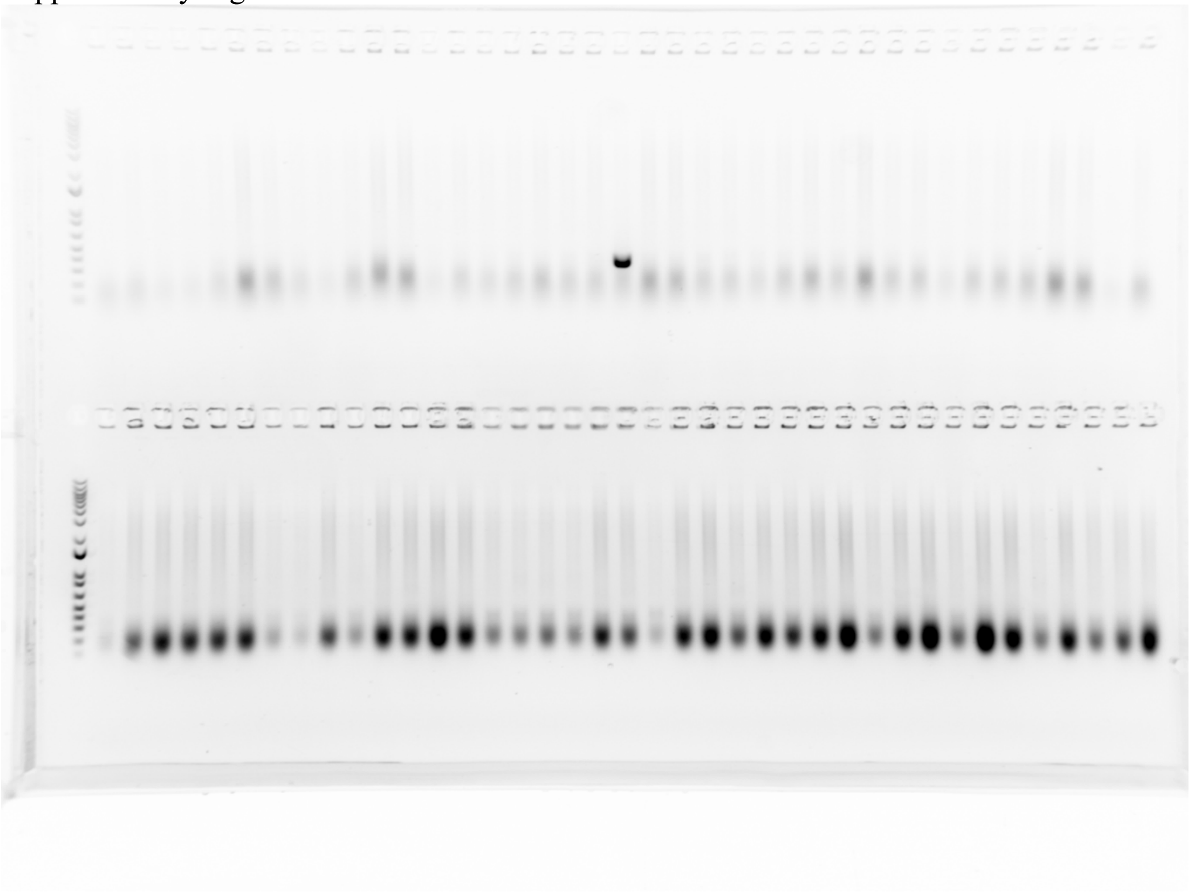

Supplementary Figure 1H

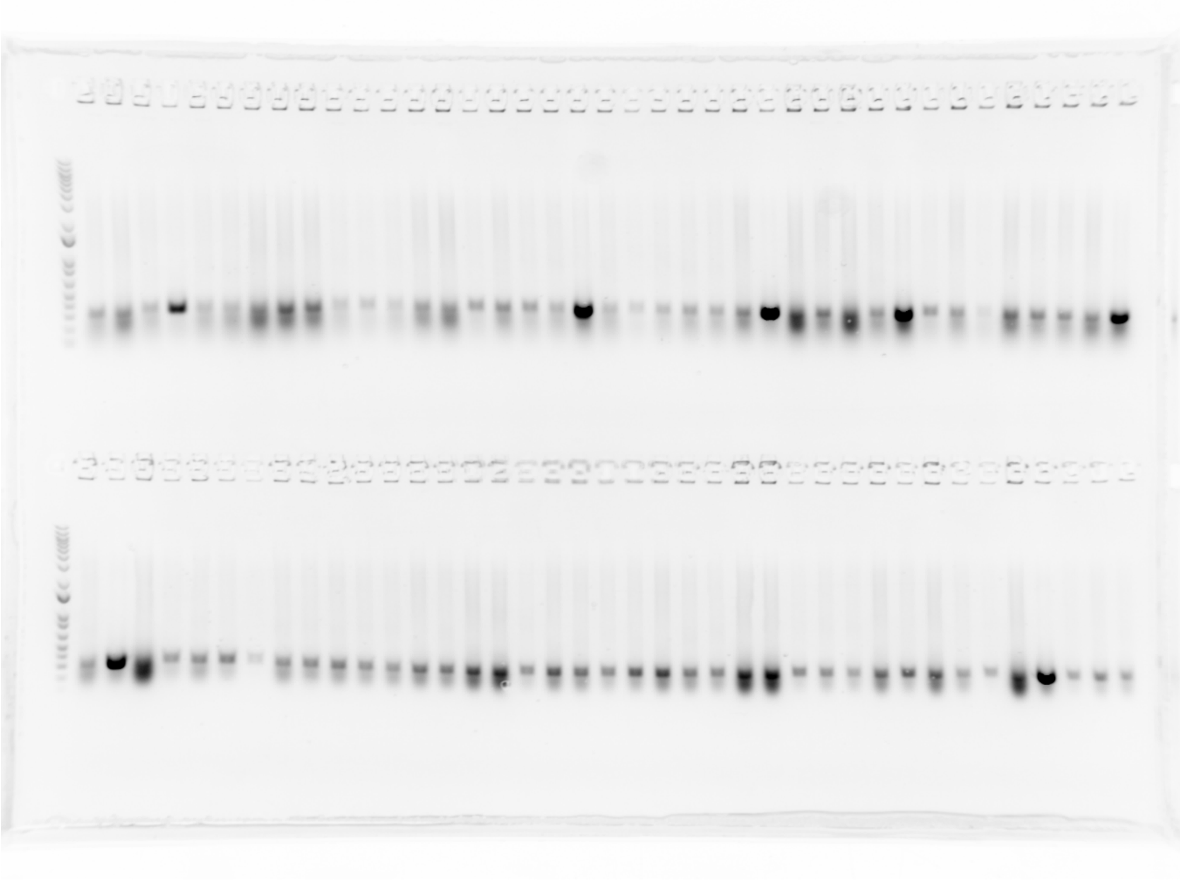

Supplementary Figure 2B

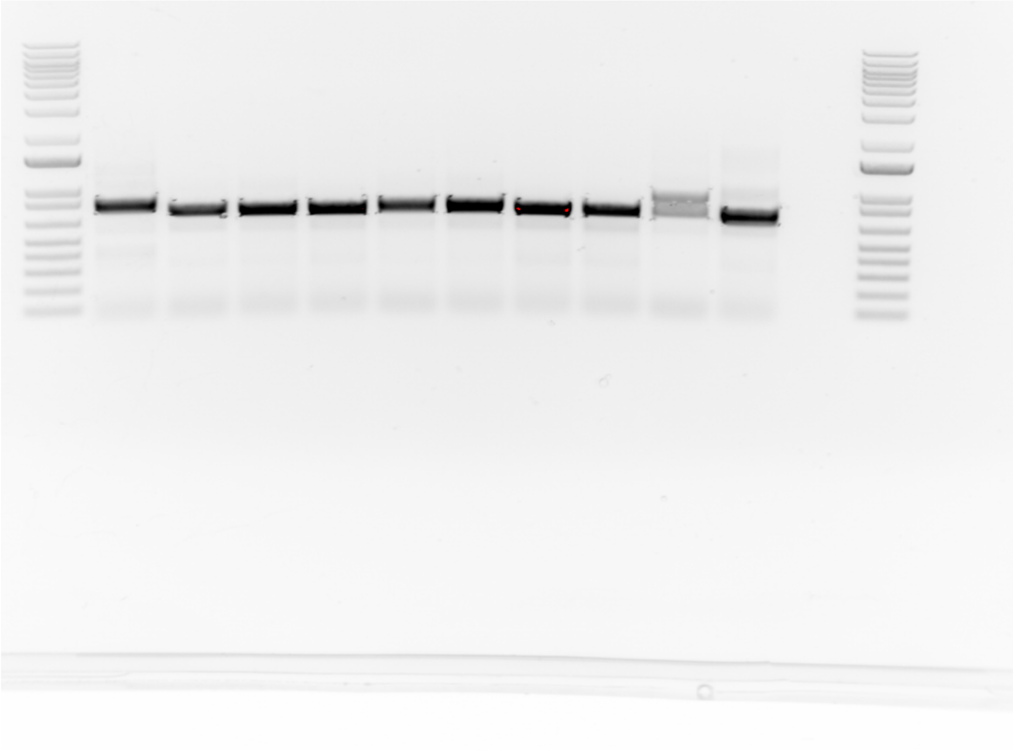

Supplementary Figure 2C

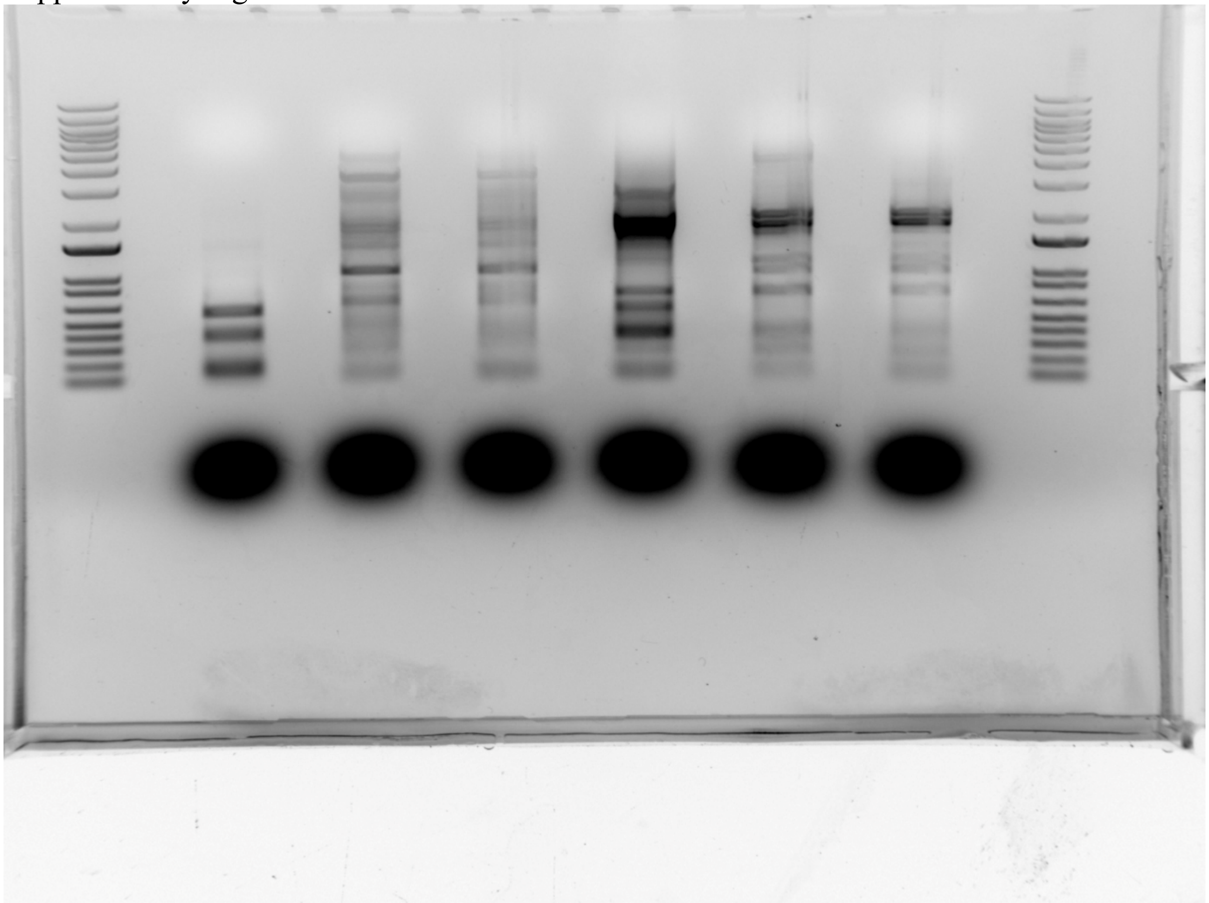

Supplementary Figure 5E

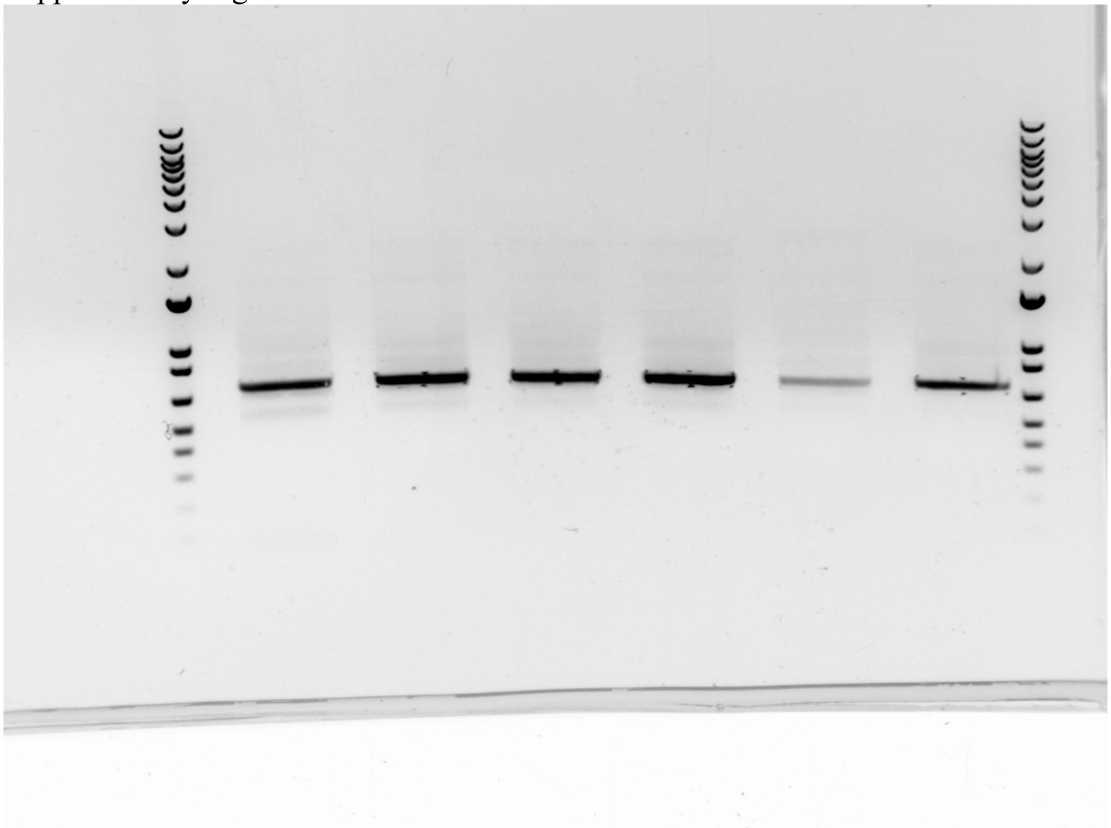

Supplement: Supplementary file 1 — Supplementary Information [file 41467_2024_49400_MOESM1_ESM.pdf]
