# Supplementary material for: A scalable and cGMP-compatible autologous organotypic cell therapy for Dystrophic Epidermolysis Bullosa
Source: Nat Commun. 2024 Jul 11;15:5834. doi: 10.1038/s41467-024-49400-z (PMC11239819; doi:10.1038/s41467-024-49400-z)
Supplement: Supplementary file 3 — Description of Additional Supplementary Files [file 41467_2024_49400_MOESM3_ESM.pdf]

Description of Additional Supplementary Files

File Name: Supplementary Data 1
Description: Lists of used gene expression sets to define Gibbin-dependent mesoderm-like, holoclone-like and melanocyte-like cell clusters contained in the iSCs (also see Figure 3, Supplementary Figure 7).

File Name: Supplementary Data 2
Description: List of variants represented by k-means clustering (see Figure 4B and Supplementary Figure 8B).

File Name: Supplementary Data 3
Description: Plots of whole genome sequencing coverage 1kbp and 1Mbp up-/downstream of 57 in silico predicted exonic, intronic, and intergenic off-targets for sgRNA C4 from fibroblasts and thereof derived iPS cells and iSCs.
