# Supplementary figures and images for: A scalable and cGMP-compatible autologous organotypic cell therapy for Dystrophic Epidermolysis Bullosa (part 2 of 3)
Source: Nat Commun. 2024 Jul 11;15:5834. doi: 10.1038/s41467-024-49400-z (PMC11239819; doi:10.1038/s41467-024-49400-z)

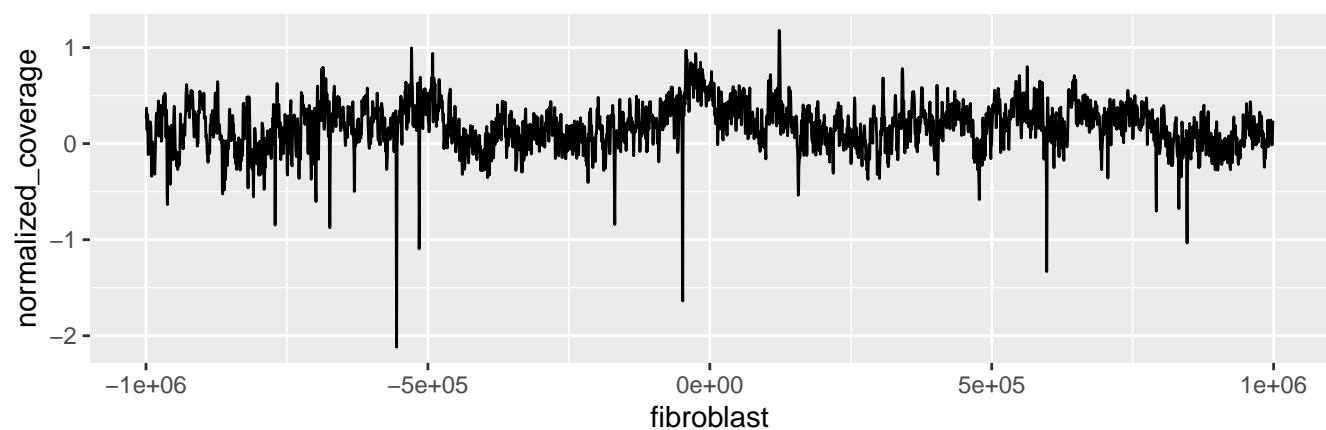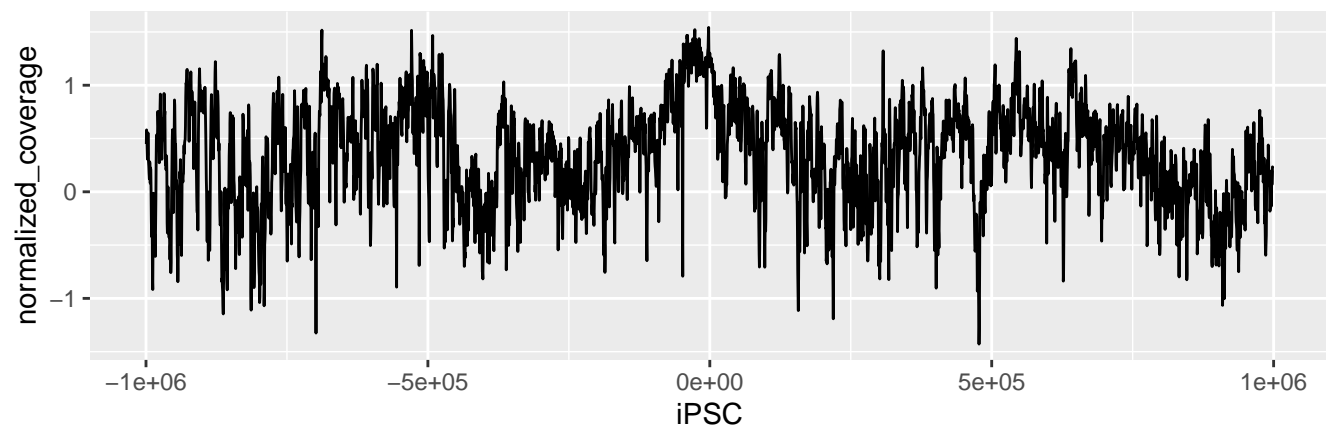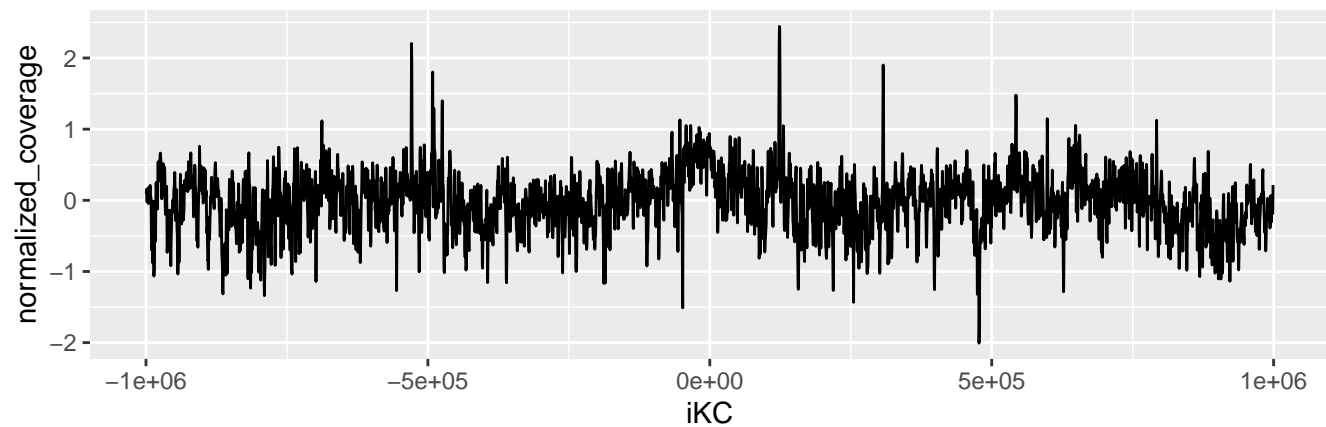

Supplement: Supplementary file 6 — Supplementary Data 3 [file 41467_2024_49400_MOESM6_ESM.zip › Supplementary Data 3/57_offtarget_sites/125-1_2MB/patient1.chr3_127031465_127031487.2MB.pdf]

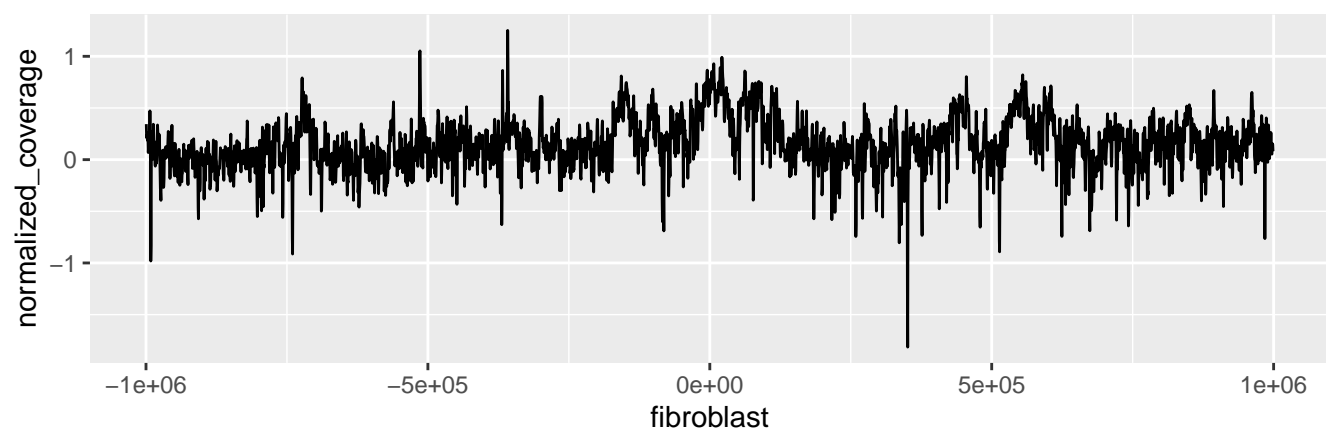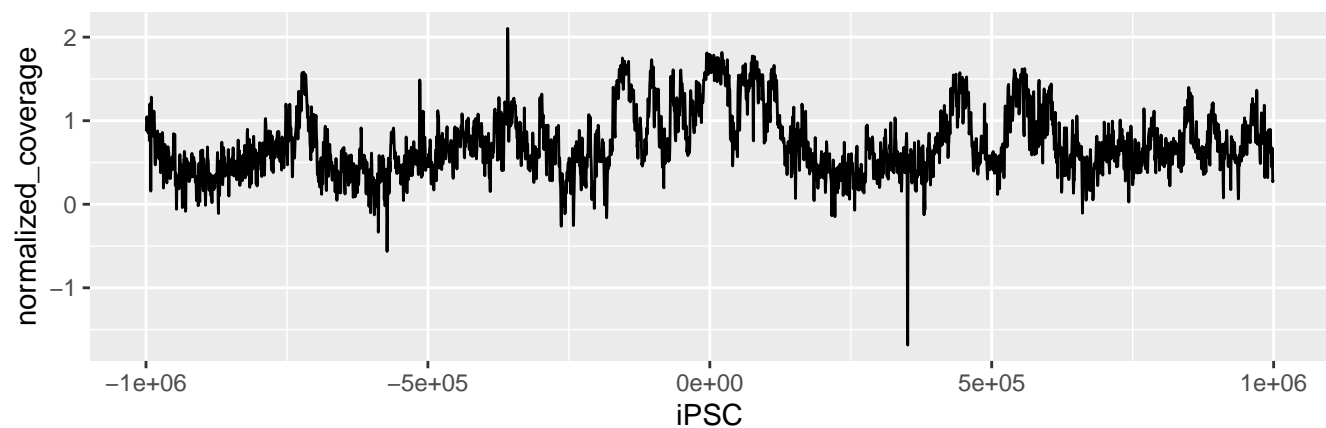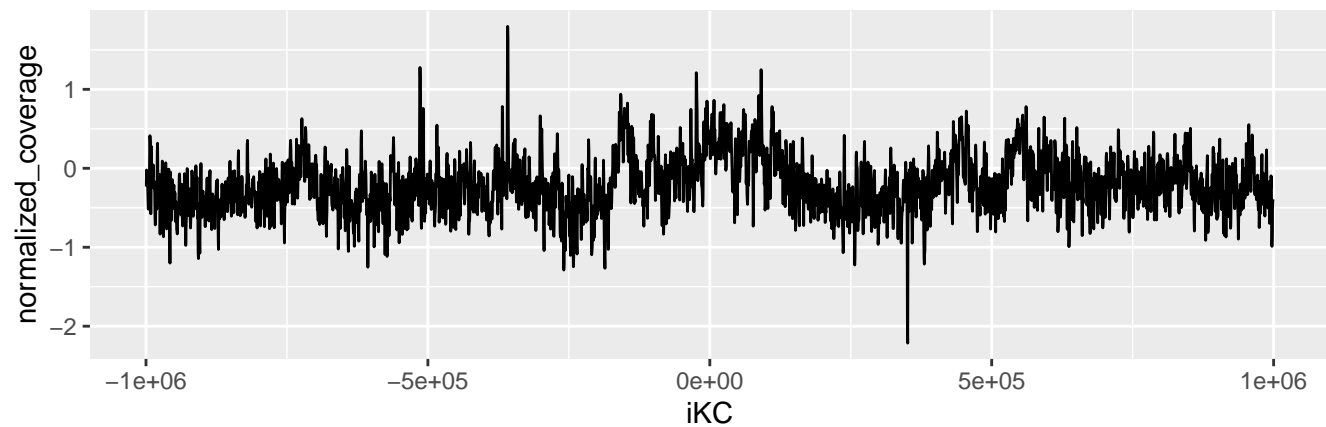

Supplement: Supplementary file 6 — Supplementary Data 3 [file 41467_2024_49400_MOESM6_ESM.zip › Supplementary Data 3/57_offtarget_sites/125-1_2MB/patient1.chr3_48570118_48570140.2MB.pdf]

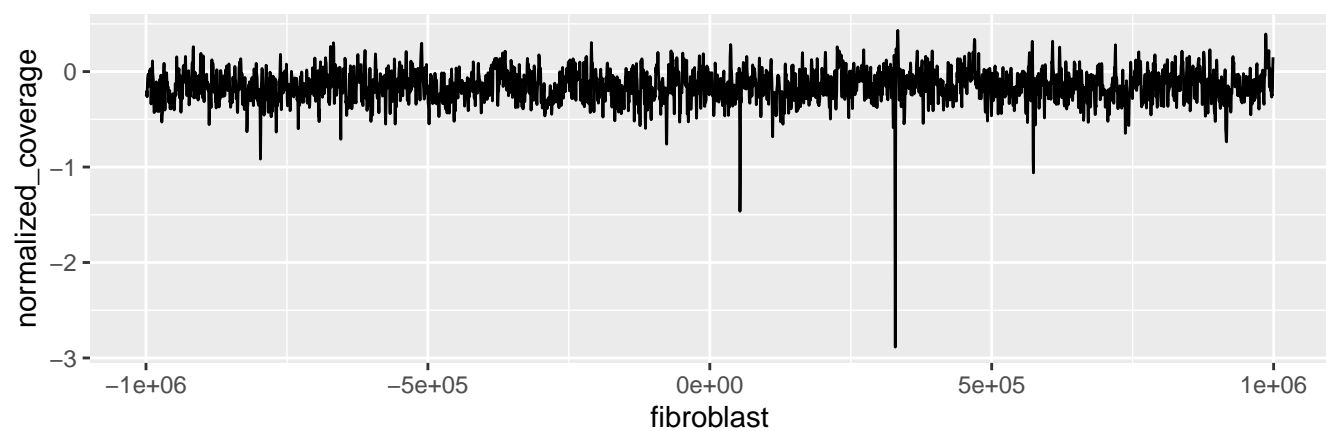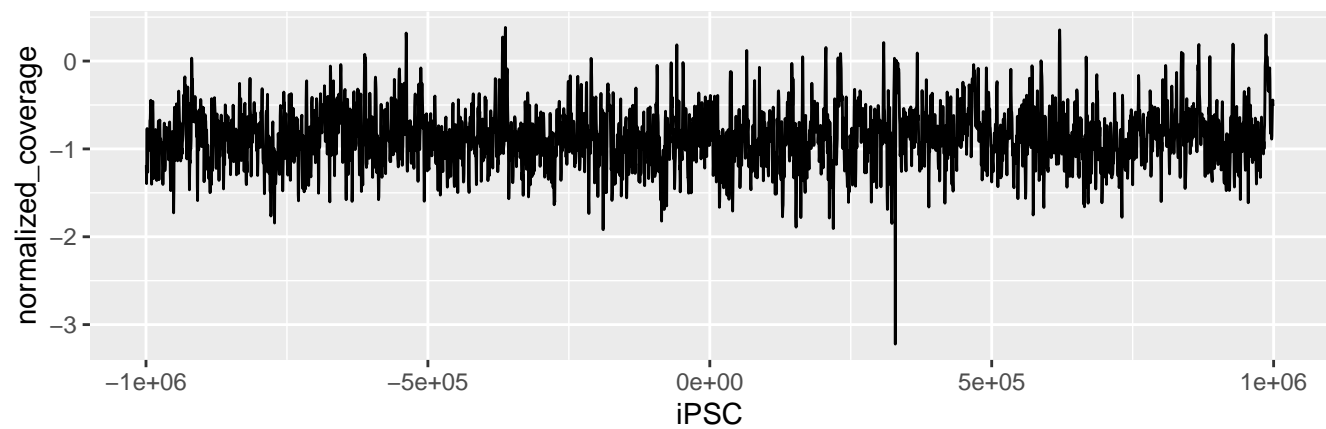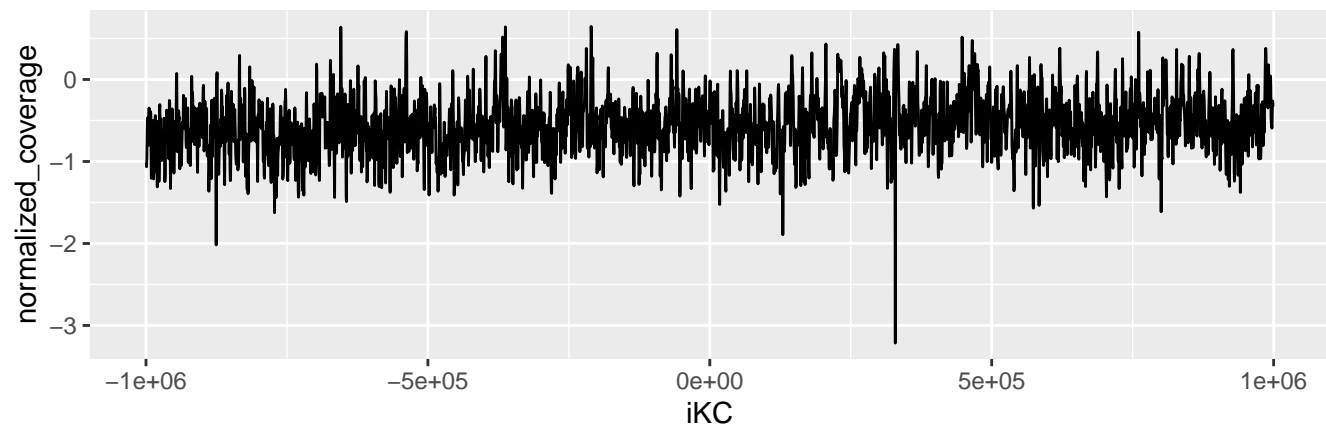

Supplement: Supplementary file 6 — Supplementary Data 3 [file 41467_2024_49400_MOESM6_ESM.zip › Supplementary Data 3/57_offtarget_sites/125-1_2MB/patient1.chr4_34873979_34874001.2MB.pdf]

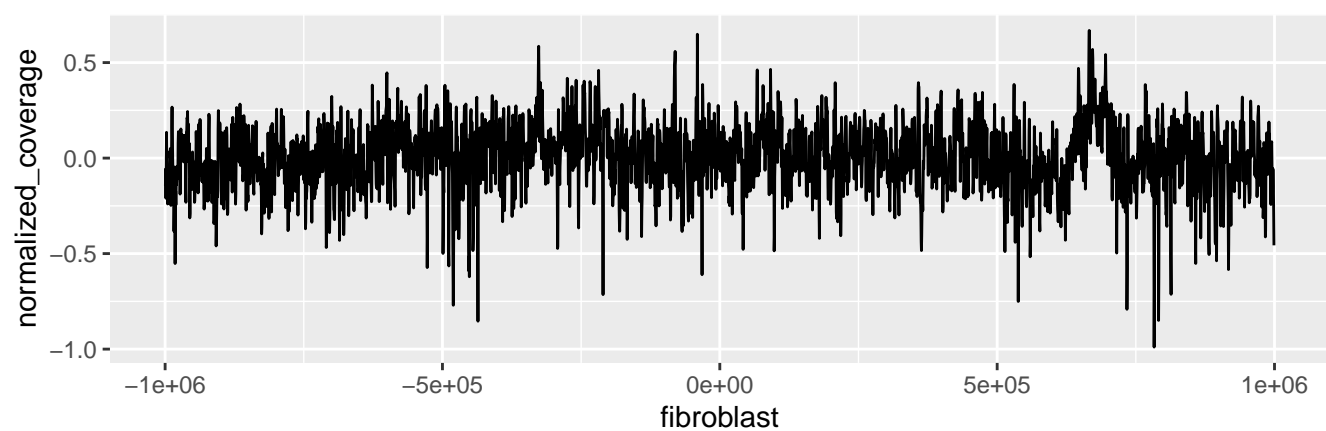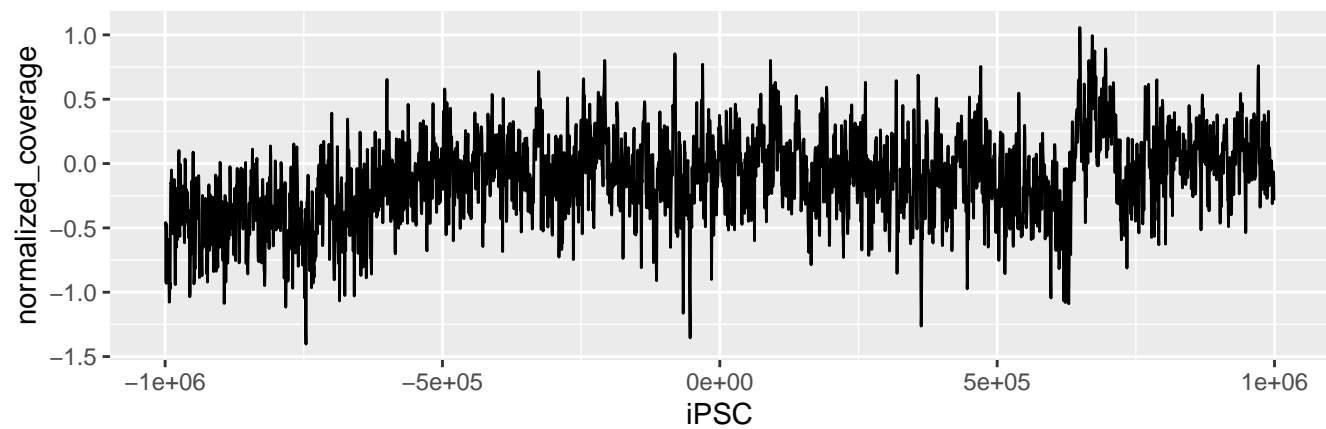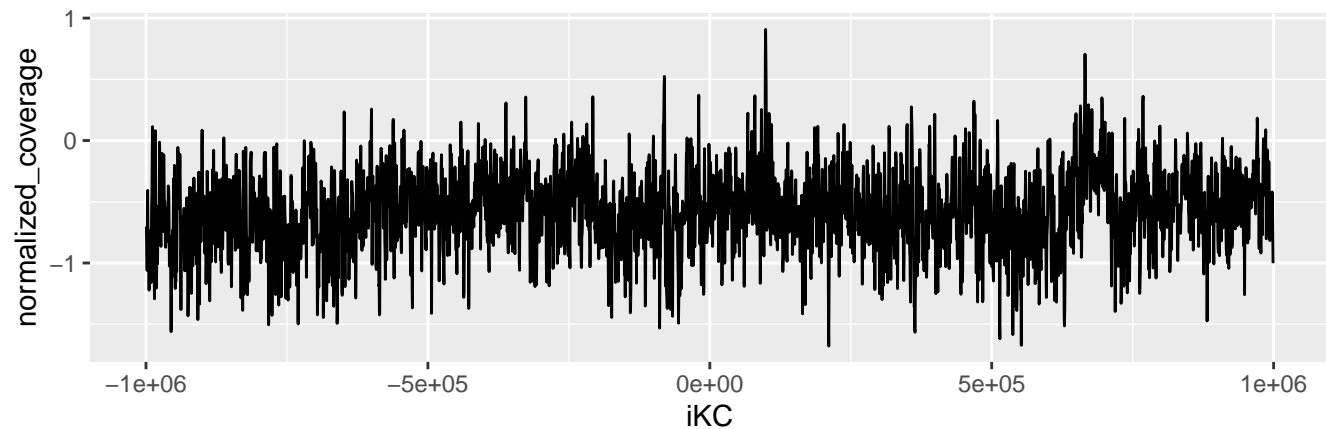

Supplement: Supplementary file 6 — Supplementary Data 3 [file 41467_2024_49400_MOESM6_ESM.zip › Supplementary Data 3/57_offtarget_sites/125-1_2MB/patient1.chr6_148779037_148779059.2MB.pdf]

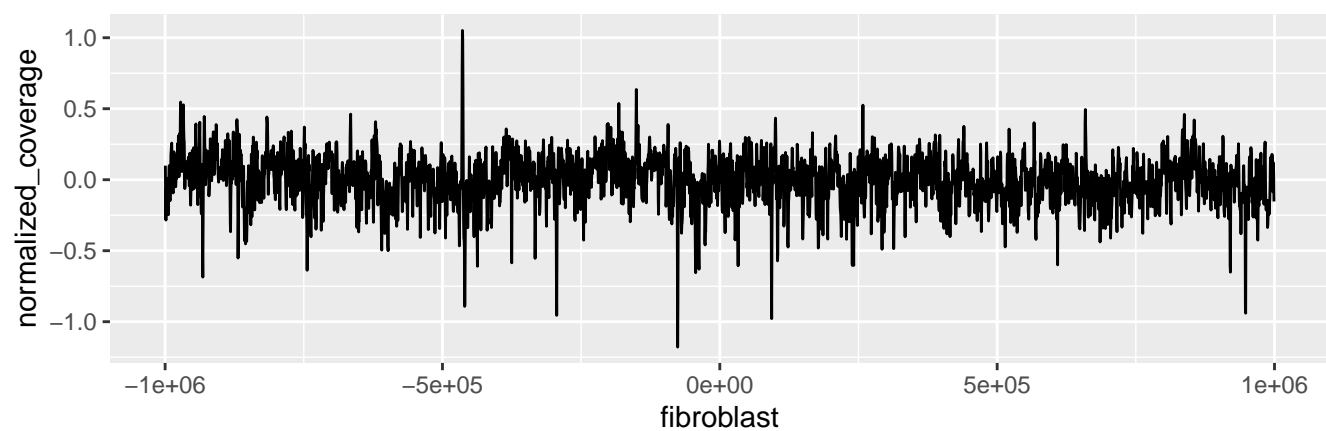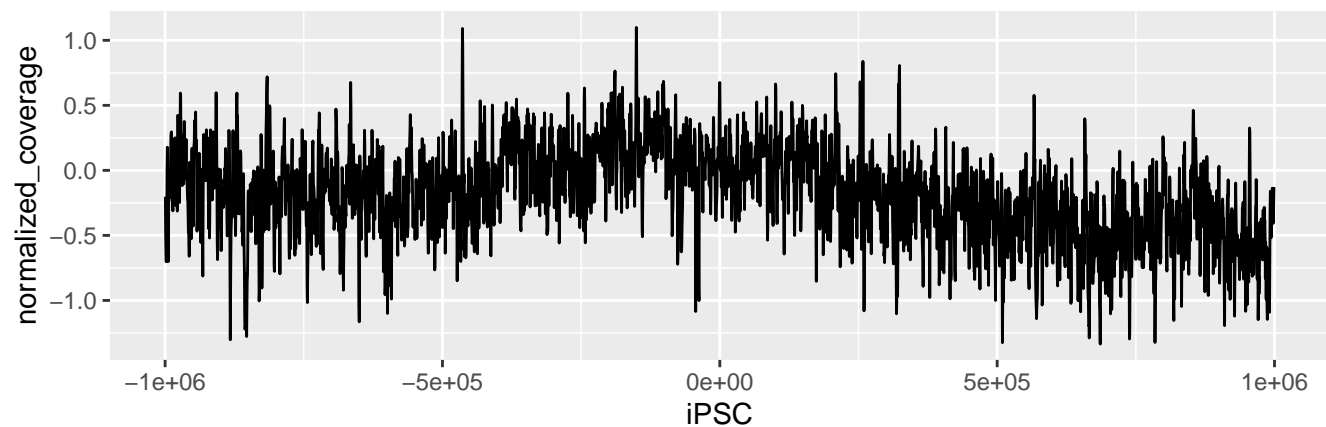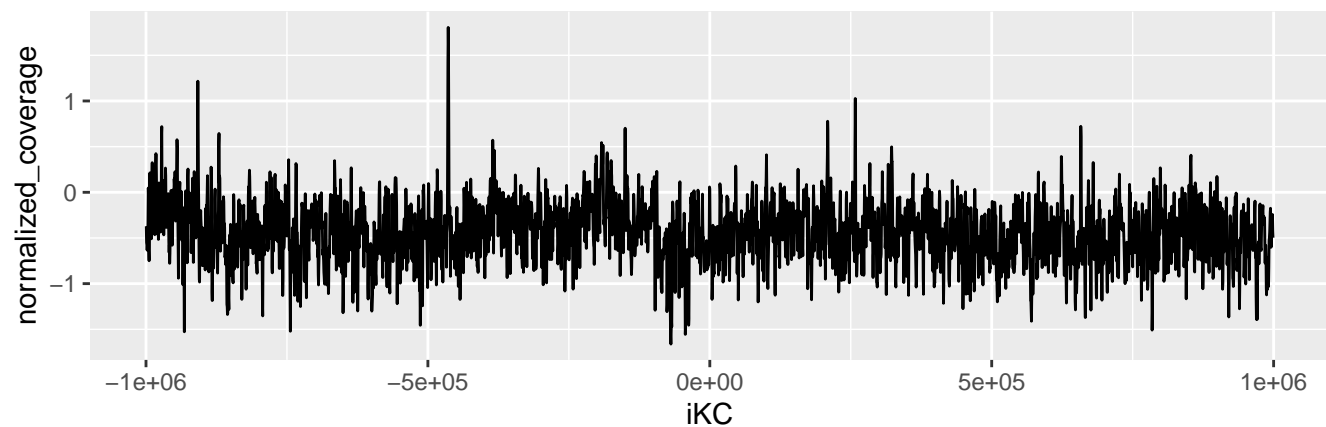

Supplement: Supplementary file 6 — Supplementary Data 3 [file 41467_2024_49400_MOESM6_ESM.zip › Supplementary Data 3/57_offtarget_sites/125-1_2MB/patient1.chr6_151241153_151241175.2MB.pdf]

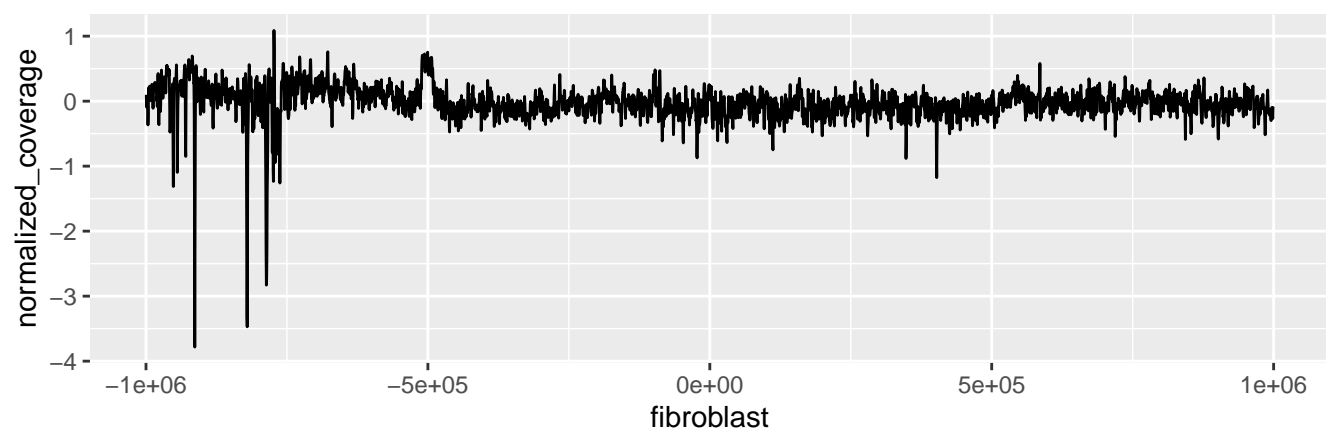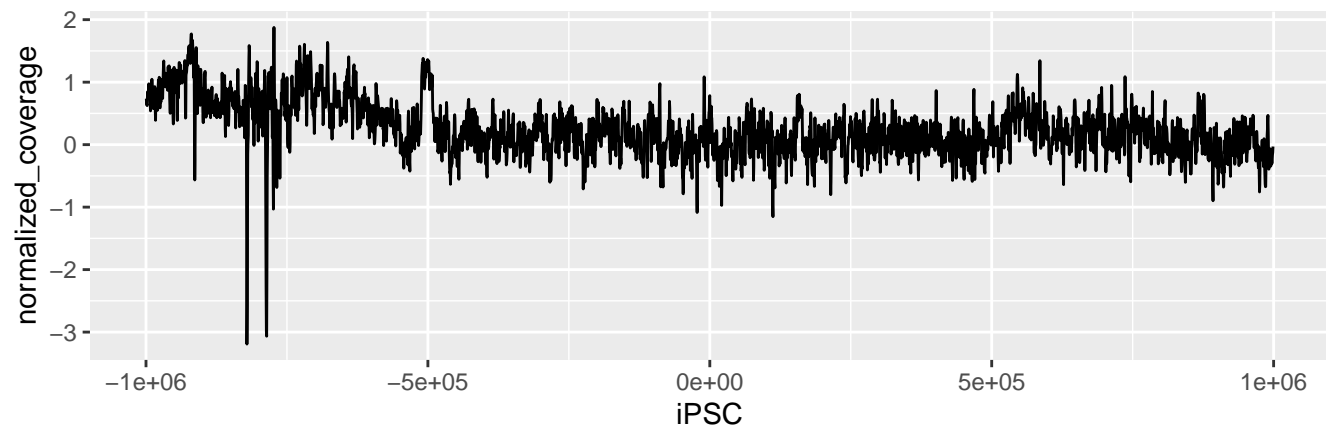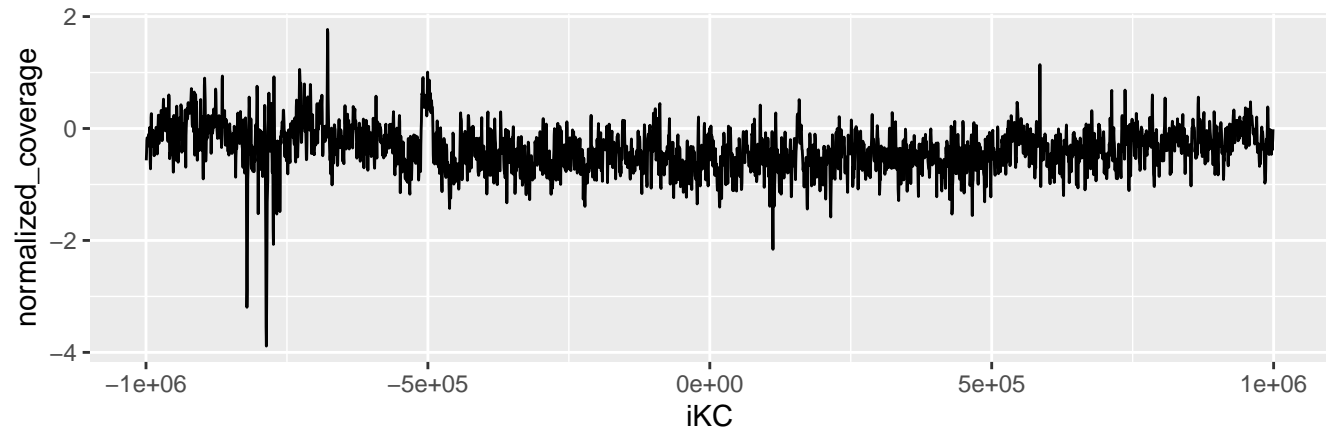

Supplement: Supplementary file 6 — Supplementary Data 3 [file 41467_2024_49400_MOESM6_ESM.zip › Supplementary Data 3/57_offtarget_sites/125-1_2MB/patient1.chr7_7567231_7567253.2MB.pdf]

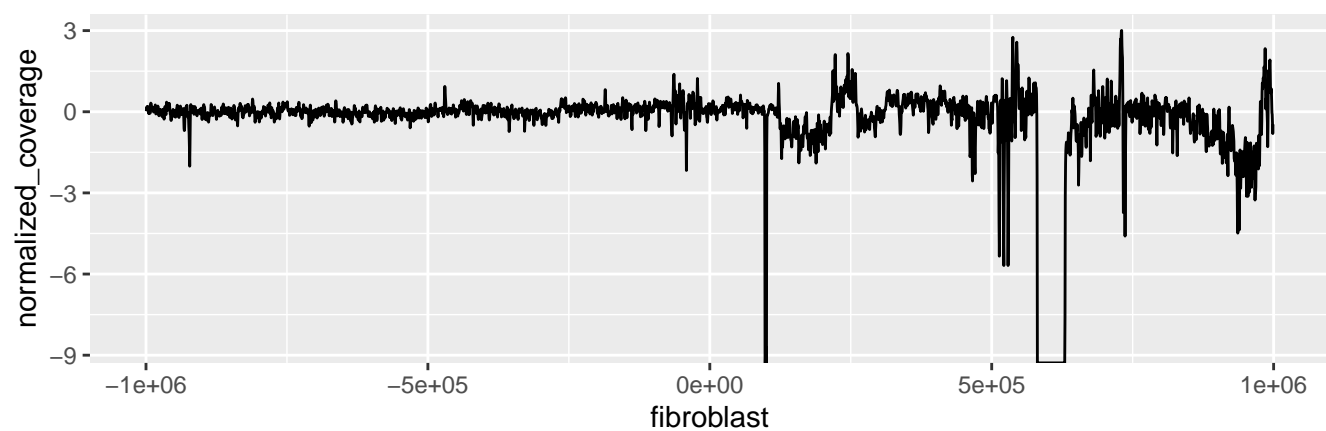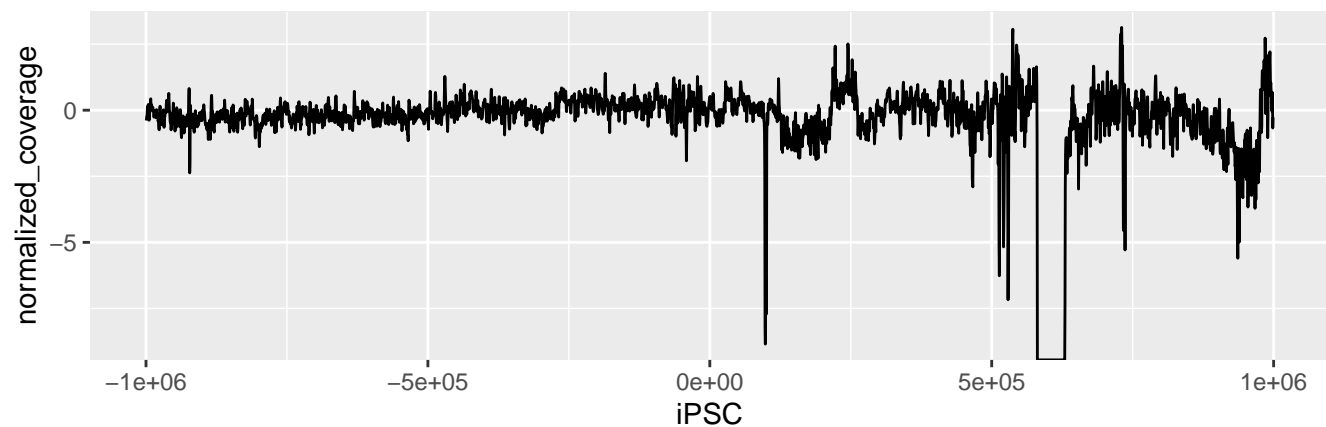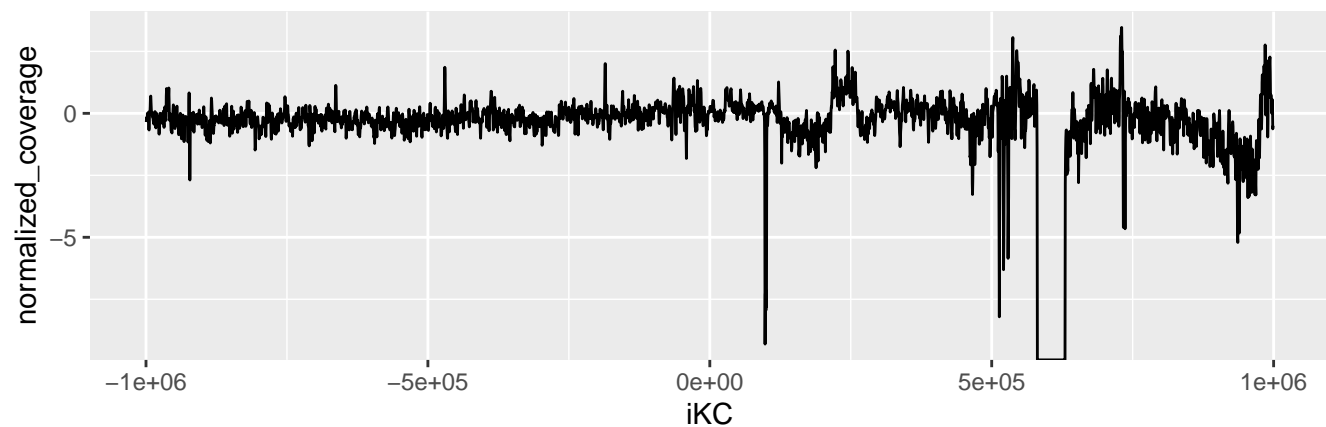

Supplement: Supplementary file 6 — Supplementary Data 3 [file 41467_2024_49400_MOESM6_ESM.zip › Supplementary Data 3/57_offtarget_sites/125-1_2MB/patient1.chr8_7036812_7036834.2MB.pdf]

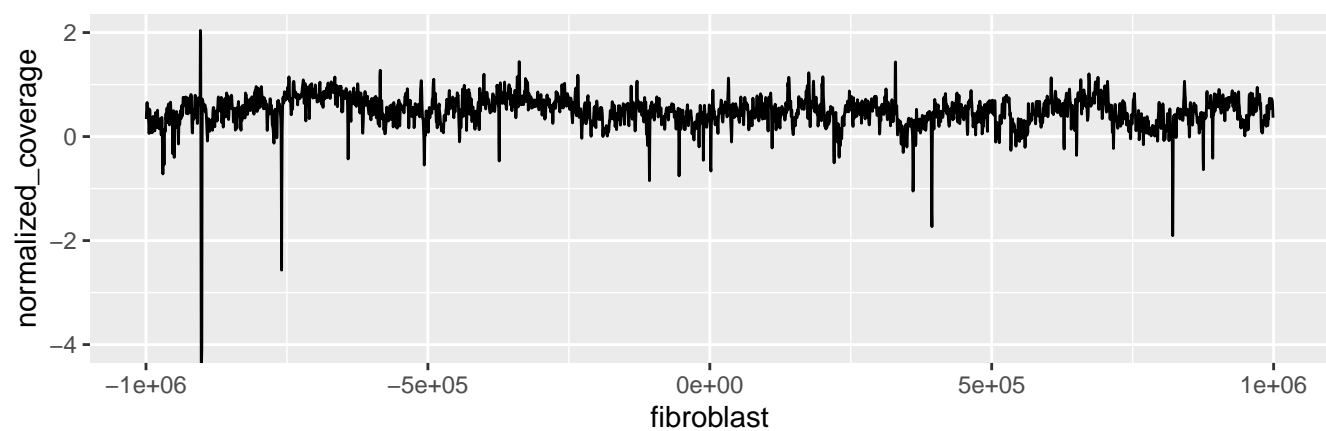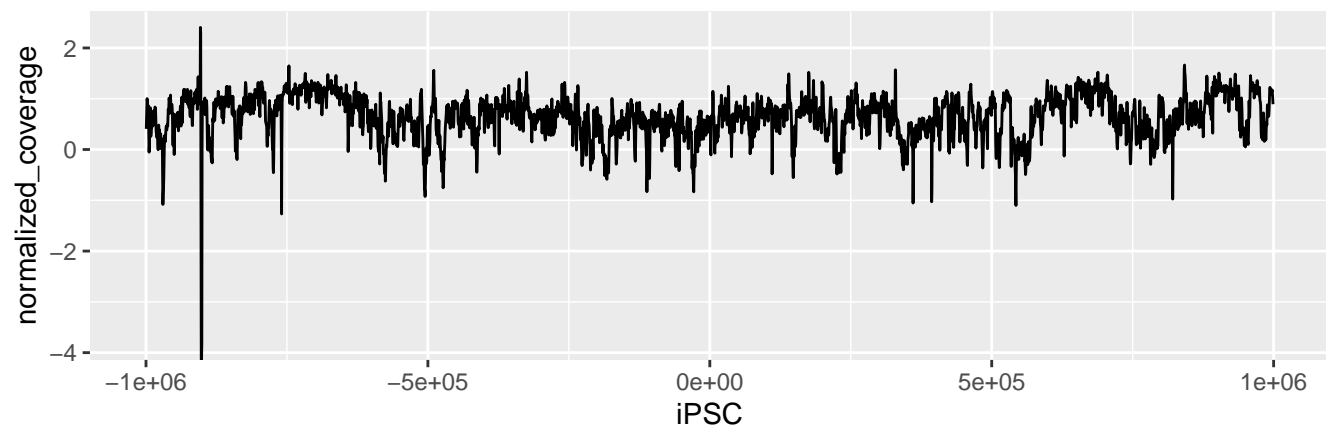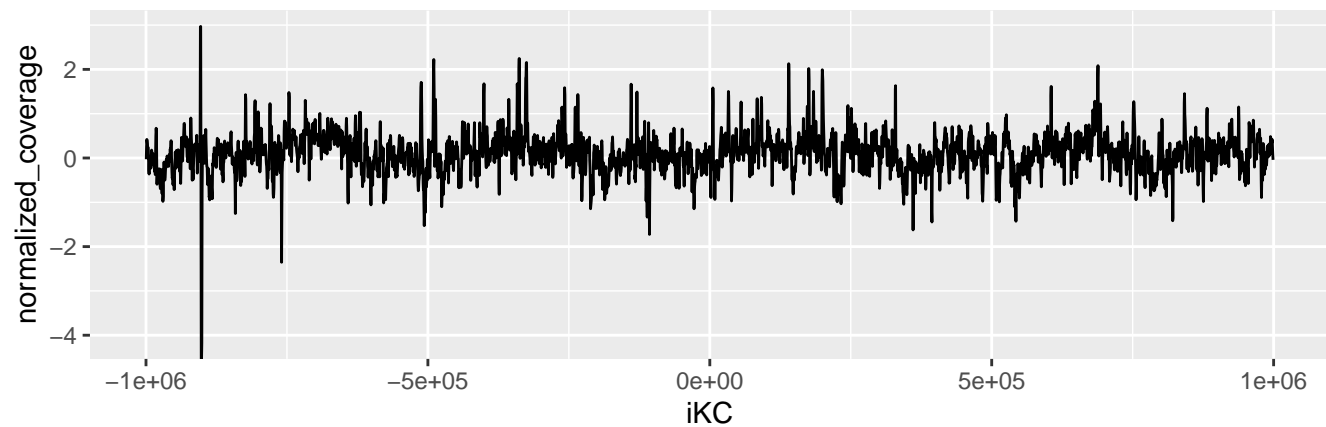

Supplement: Supplementary file 6 — Supplementary Data 3 [file 41467_2024_49400_MOESM6_ESM.zip › Supplementary Data 3/57_offtarget_sites/125-1_2MB/patient1.chr9_135086067_135086089.2MB.pdf]

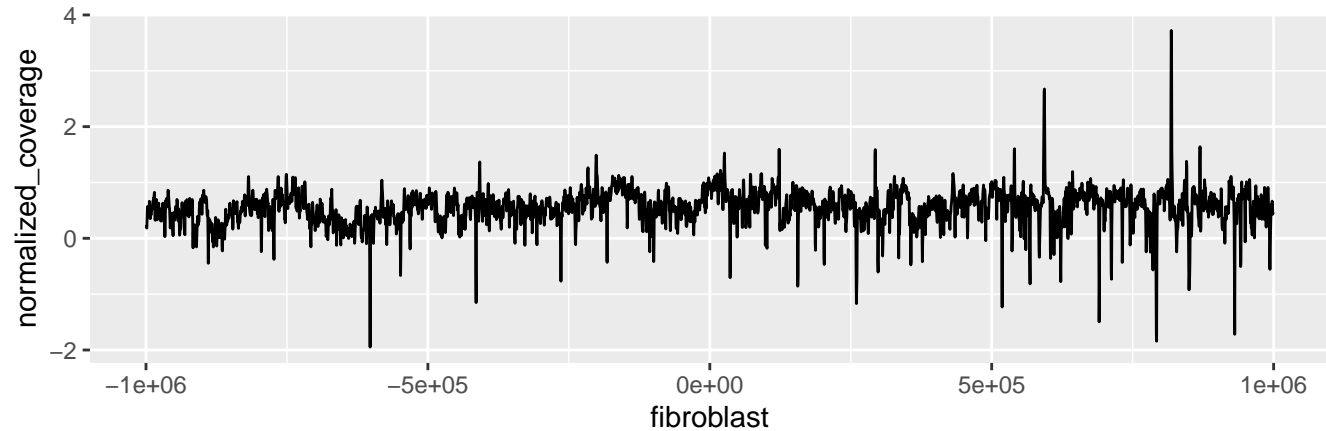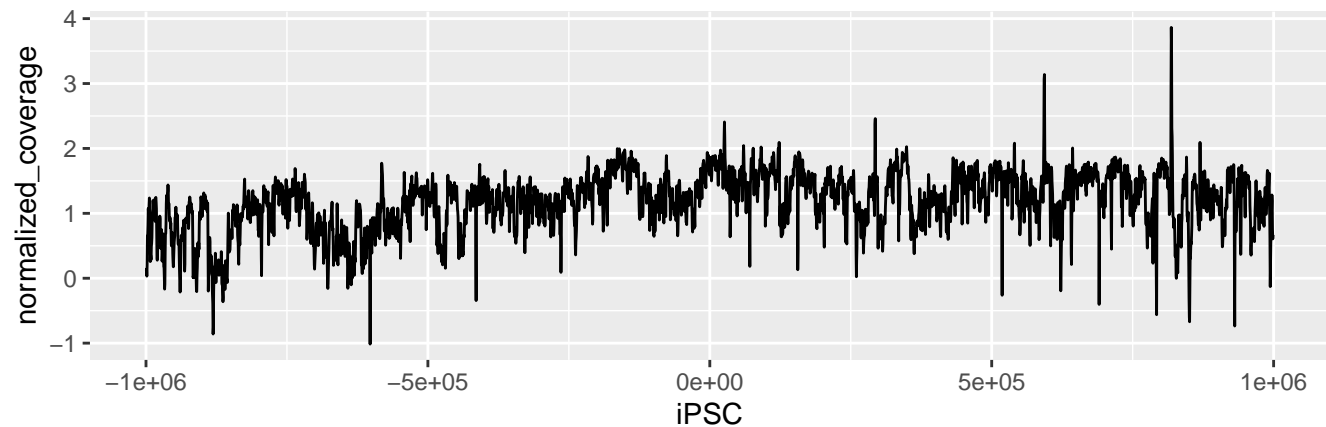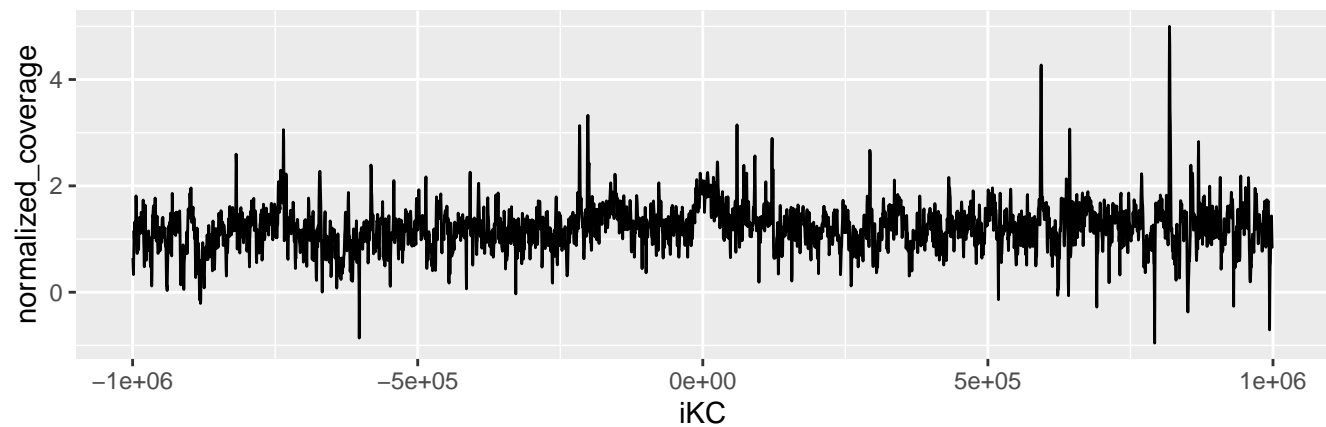

Supplement: Supplementary file 6 — Supplementary Data 3 [file 41467_2024_49400_MOESM6_ESM.zip › Supplementary Data 3/57_offtarget_sites/125-1_2MB/patient1.chr9_136509758_136509780.2MB.pdf]

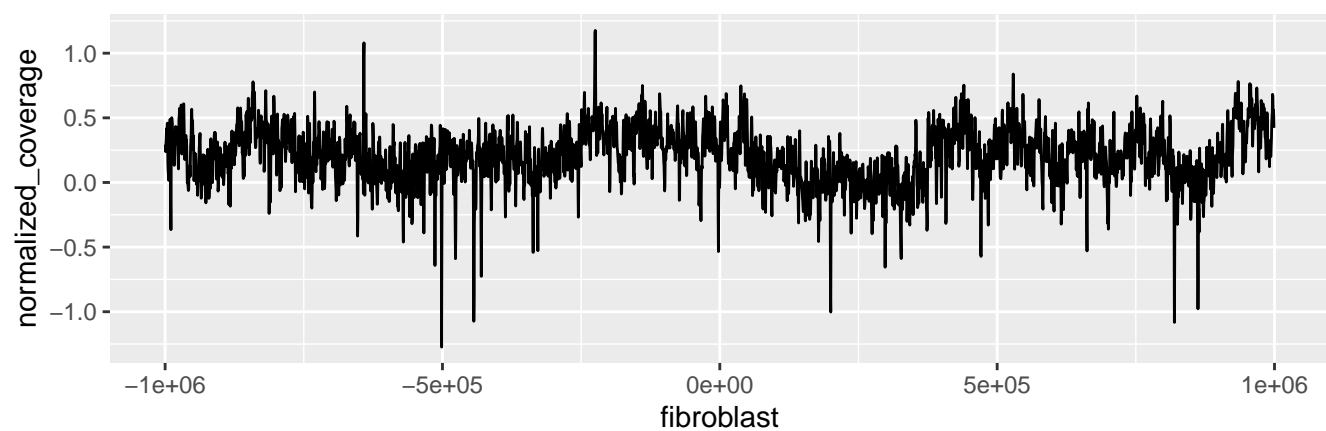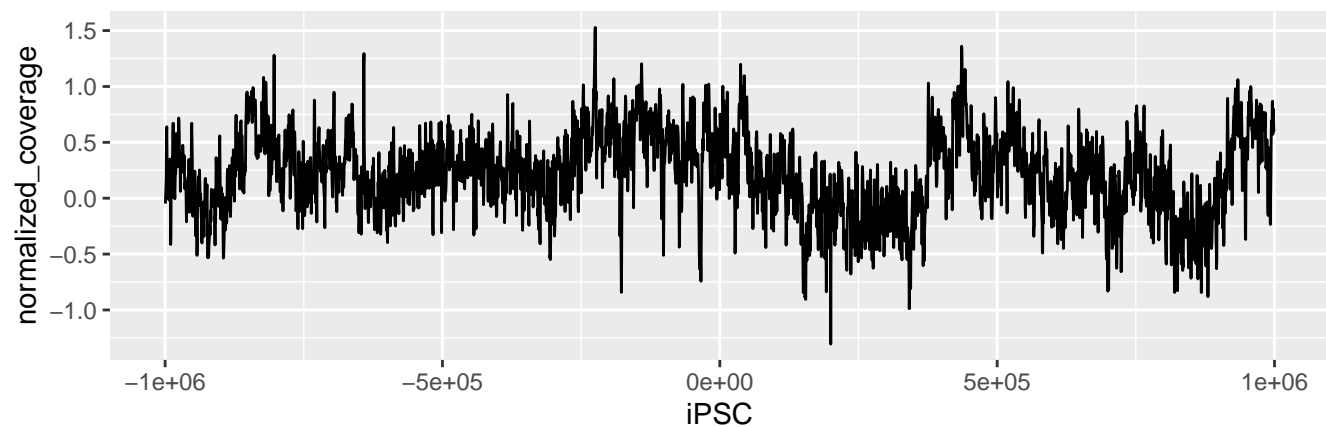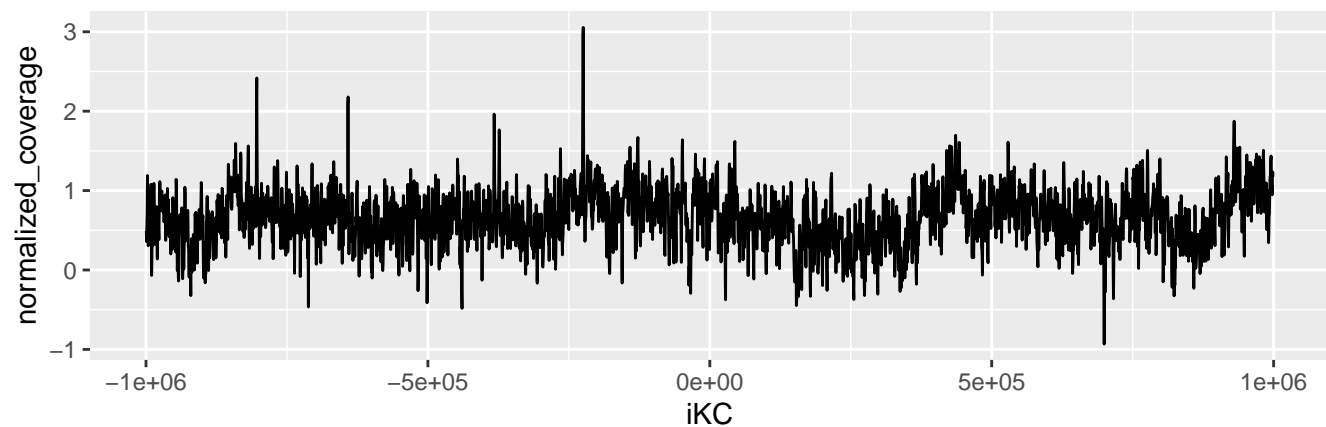

Supplement: Supplementary file 6 — Supplementary Data 3 [file 41467_2024_49400_MOESM6_ESM.zip › Supplementary Data 3/57_offtarget_sites/125-1_2MB/patient1.chr9_36989005_36989027.2MB.pdf]

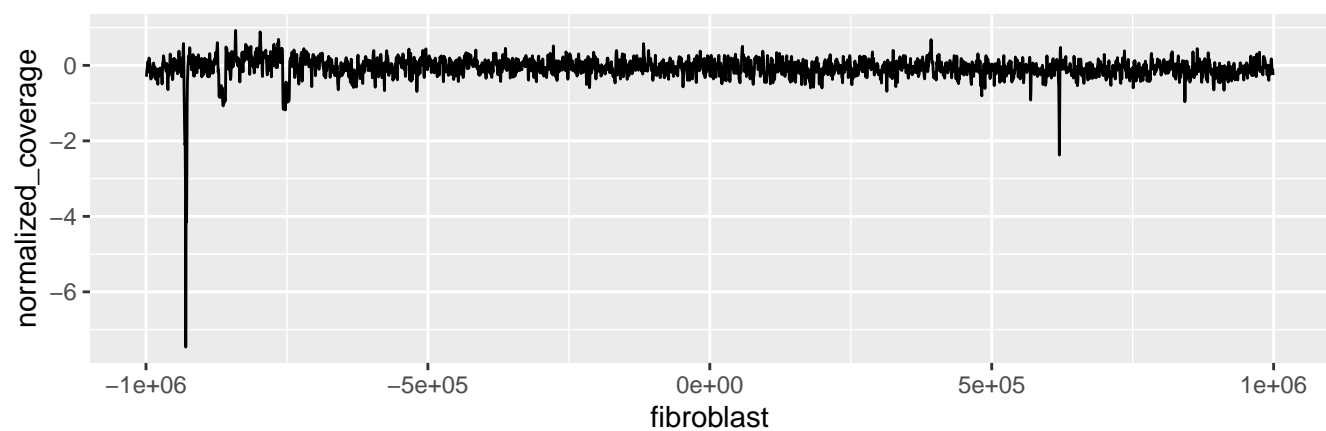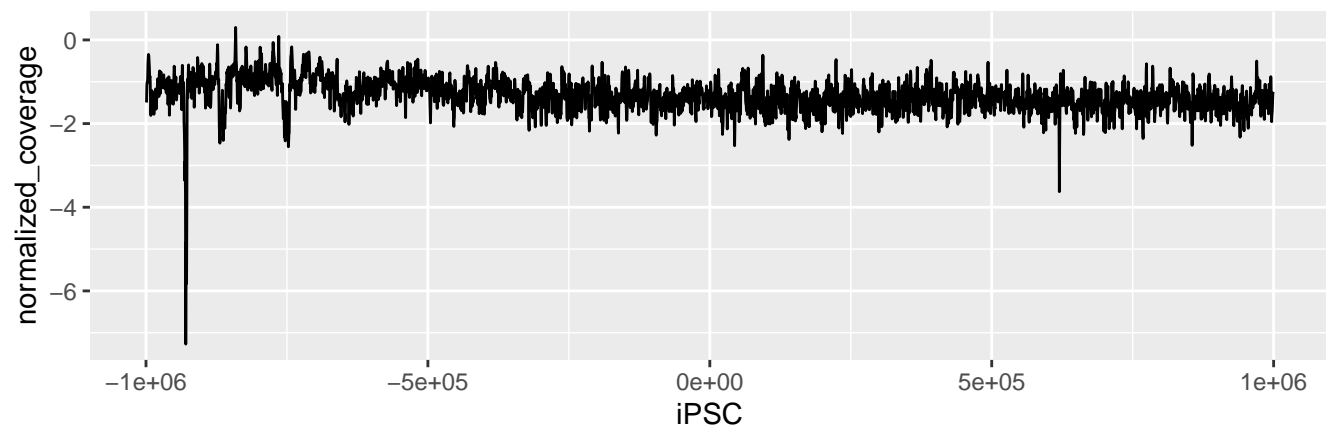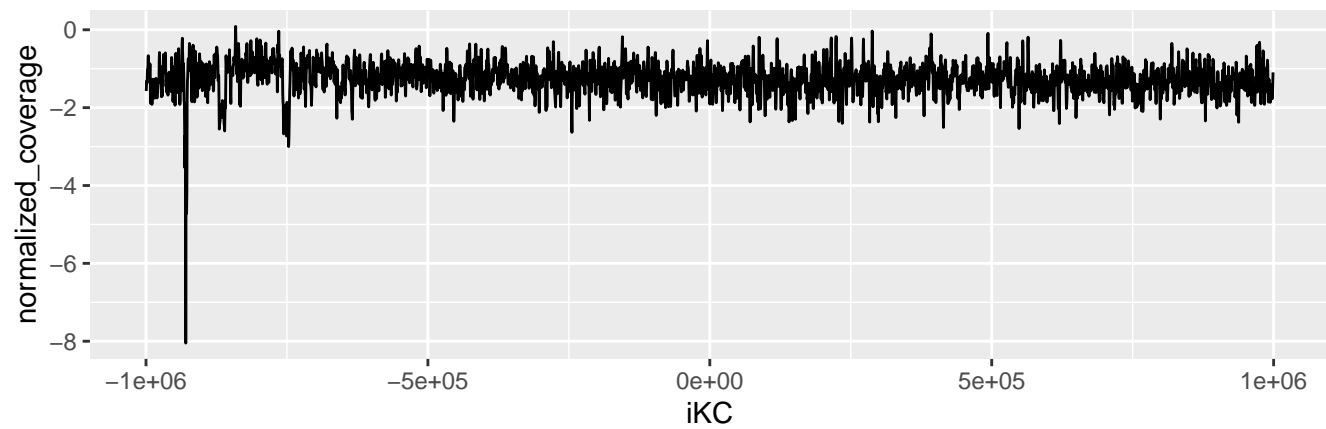

Supplement: Supplementary file 6 — Supplementary Data 3 [file 41467_2024_49400_MOESM6_ESM.zip › Supplementary Data 3/57_offtarget_sites/125-1_2MB/patient1.chrX_104827480_104827502.2MB.pdf]

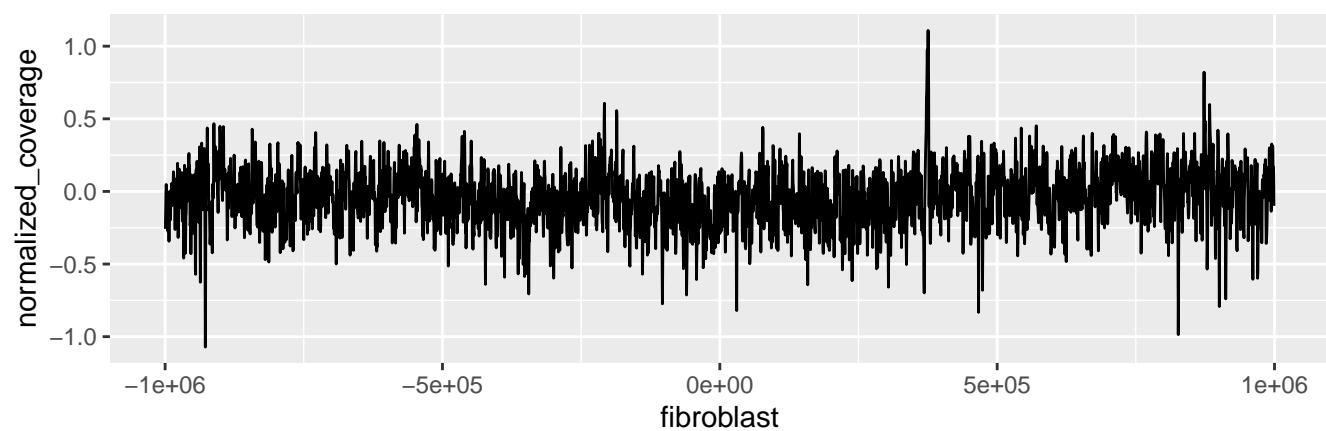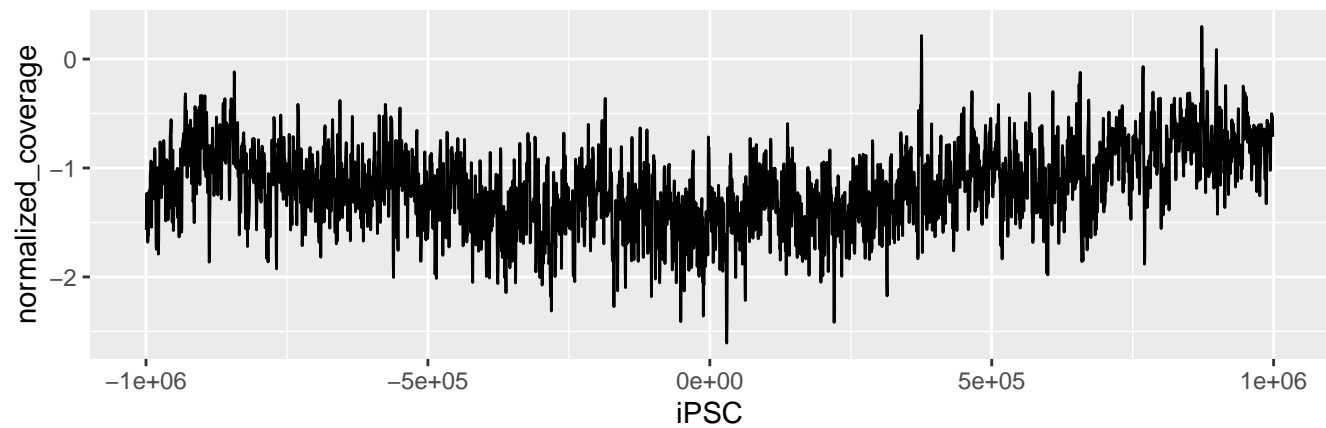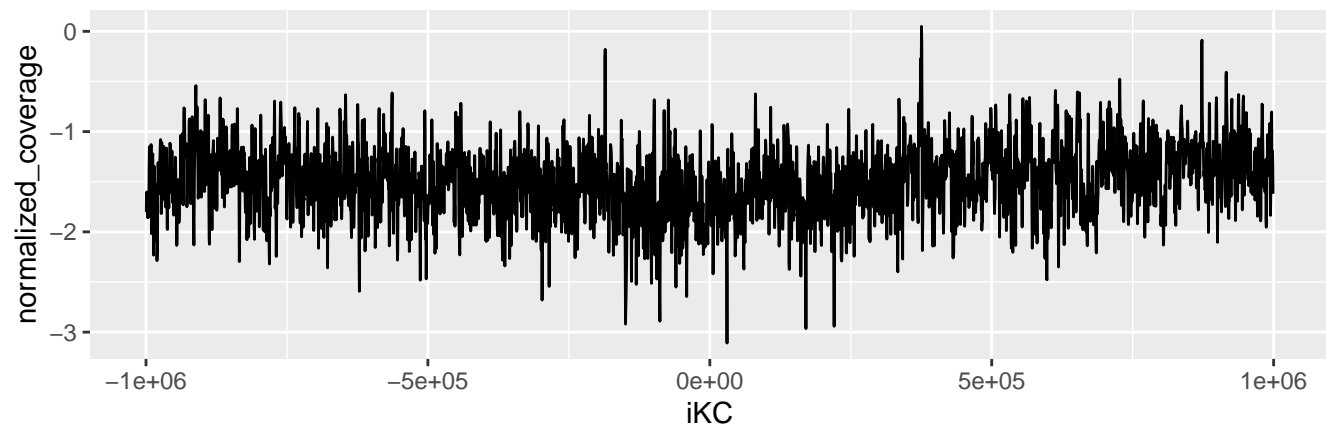

Supplement: Supplementary file 6 — Supplementary Data 3 [file 41467_2024_49400_MOESM6_ESM.zip › Supplementary Data 3/57_offtarget_sites/125-1_2MB/patient1.chrX_22868452_22868474.2MB.pdf]

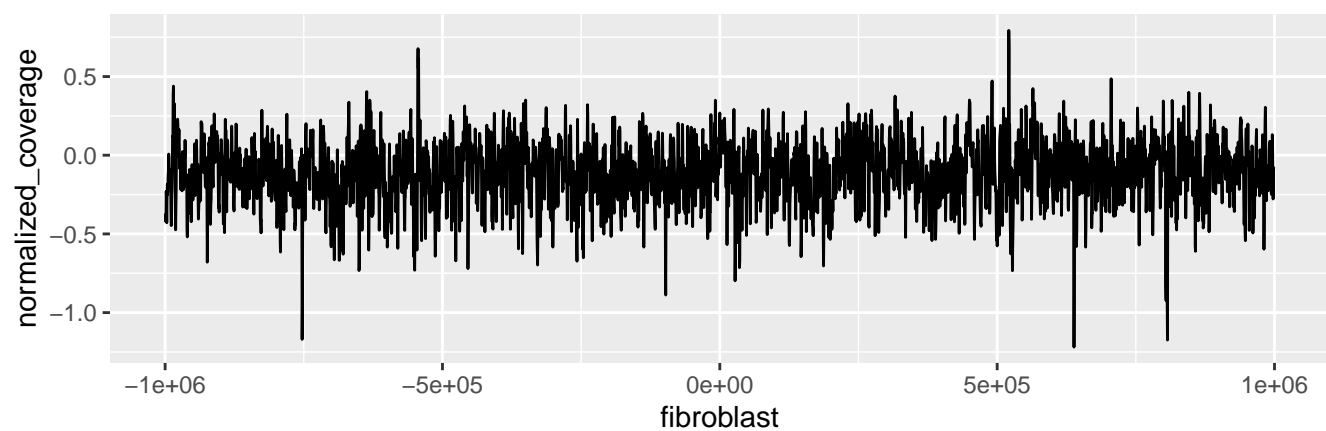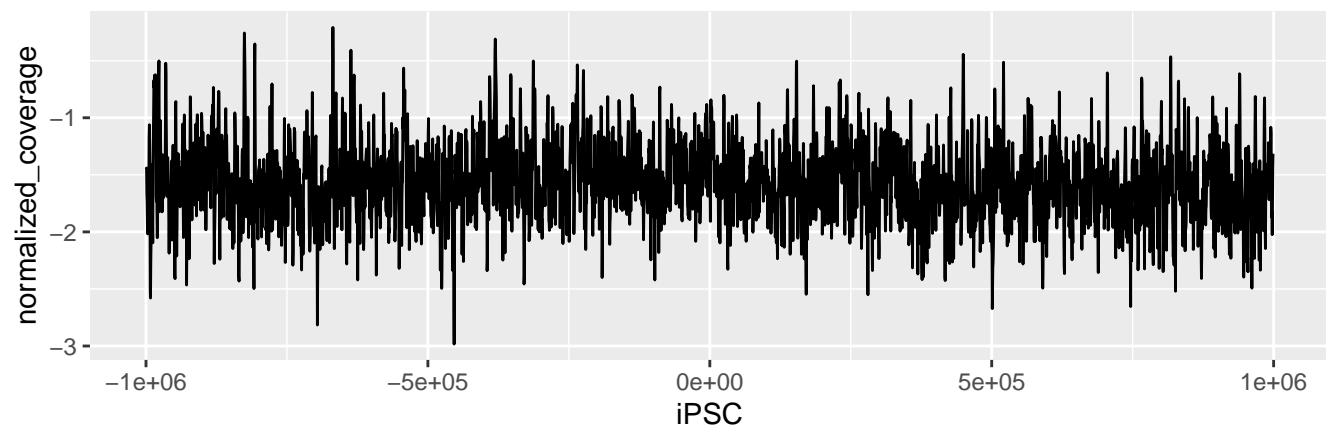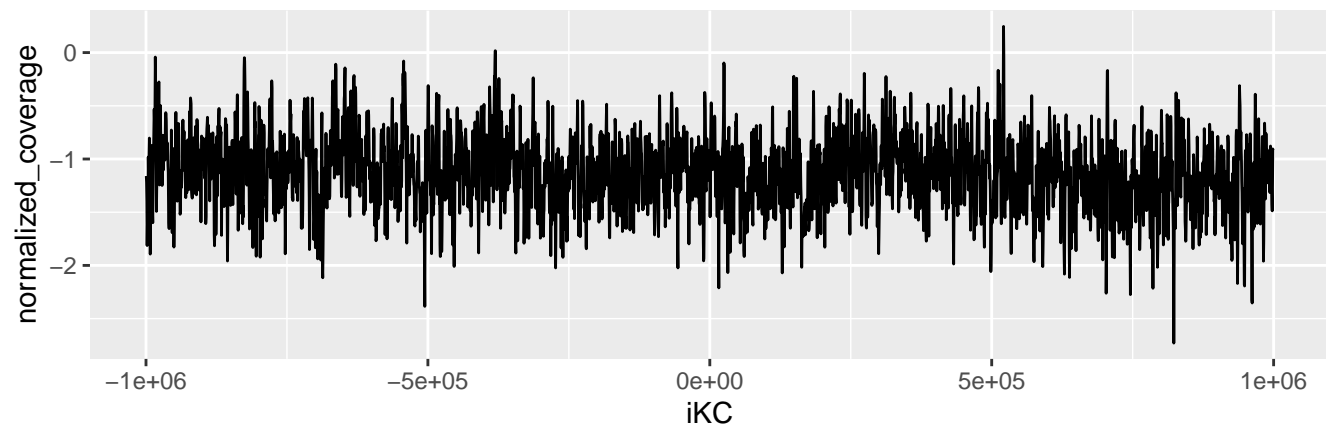

Supplement: Supplementary file 6 — Supplementary Data 3 [file 41467_2024_49400_MOESM6_ESM.zip › Supplementary Data 3/57_offtarget_sites/125-1_2MB/patient1.chrX_34794493_34794515.2MB.pdf]

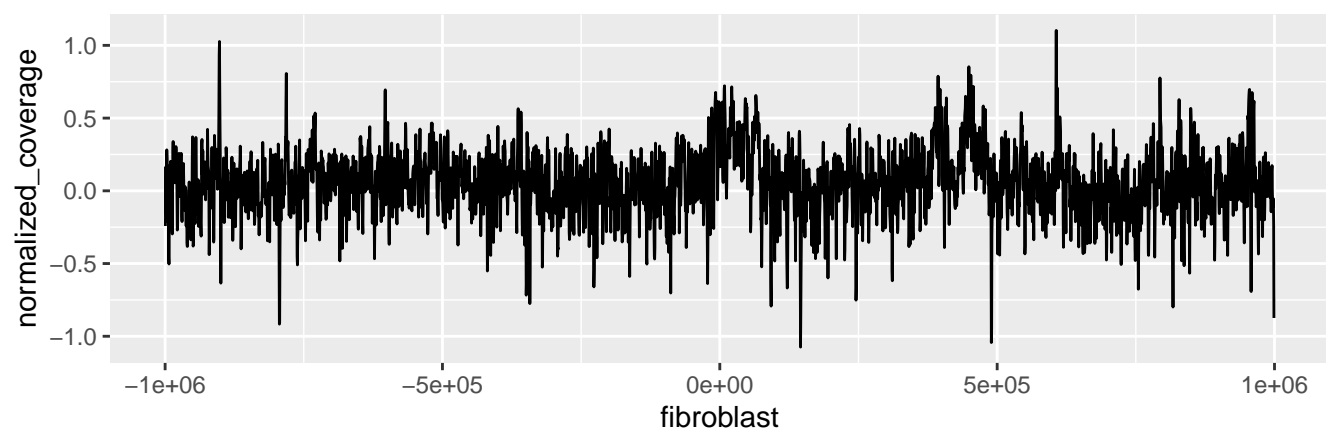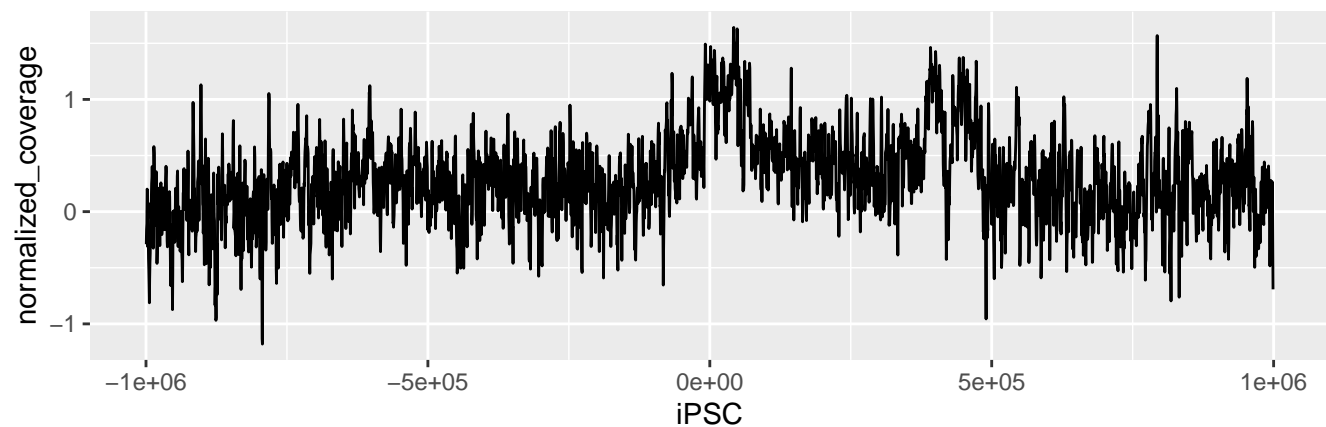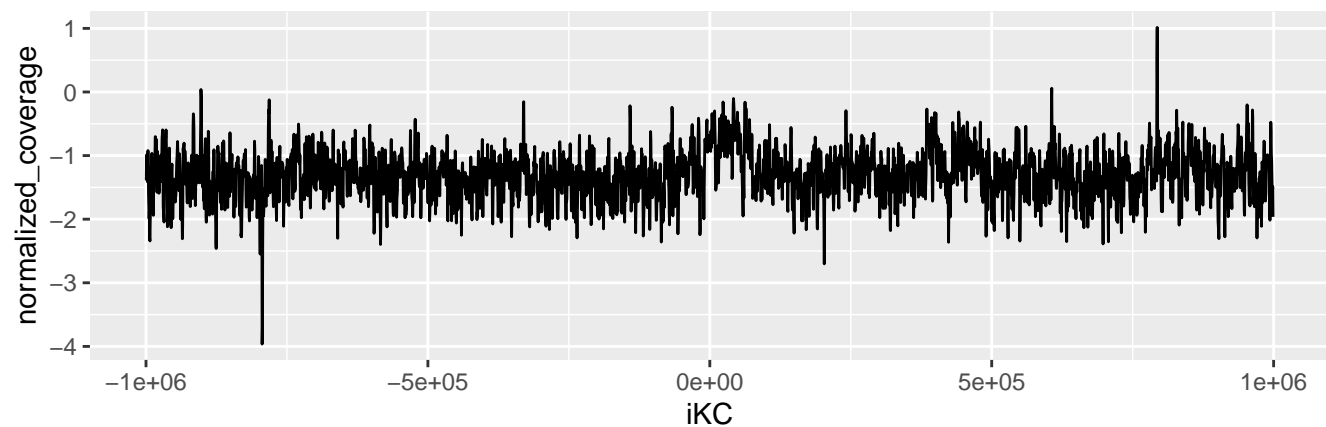

Supplement: Supplementary file 6 — Supplementary Data 3 [file 41467_2024_49400_MOESM6_ESM.zip › Supplementary Data 3/57_offtarget_sites/125-1_2MB/patient1.chrX_47178057_47178079.2MB.pdf]

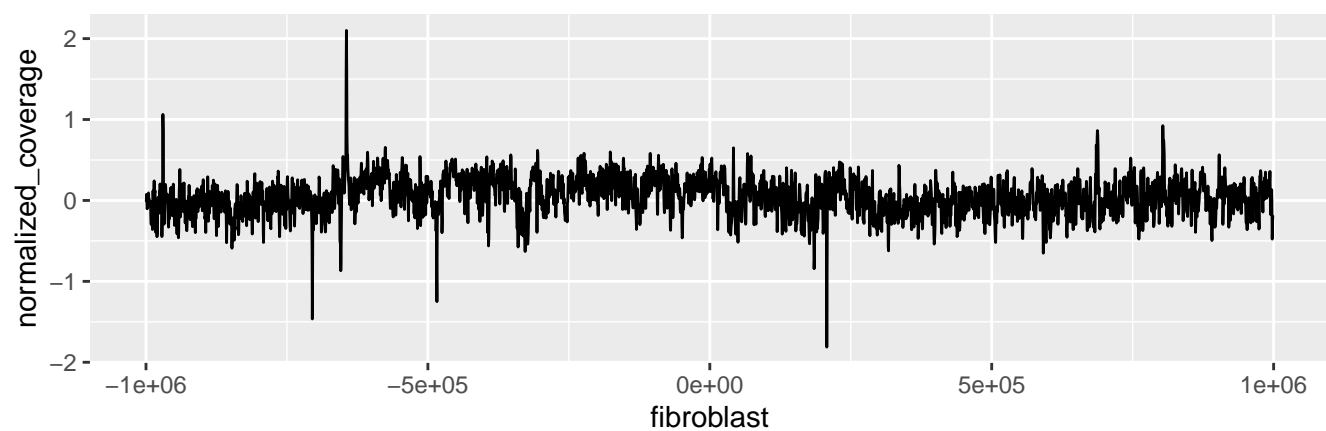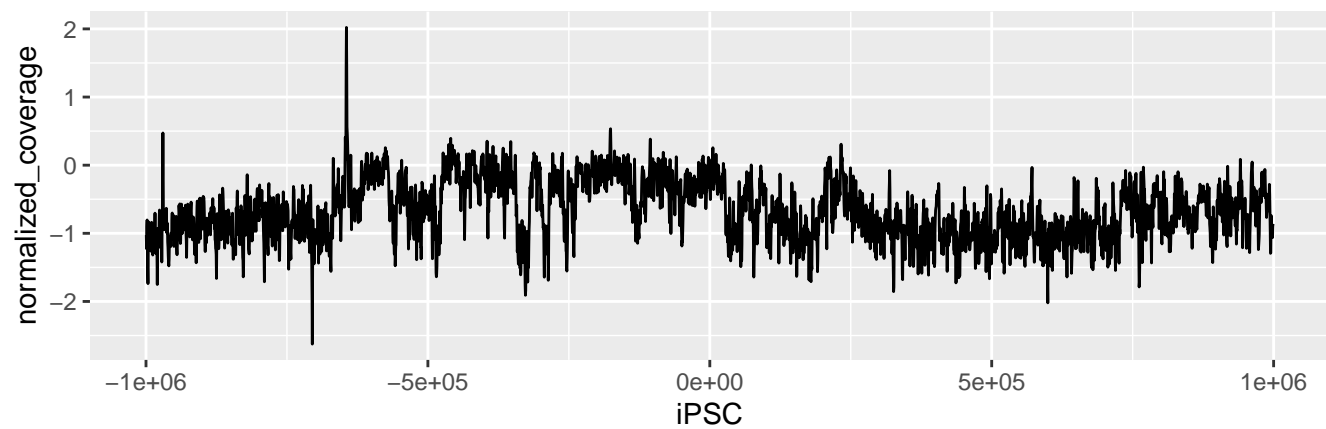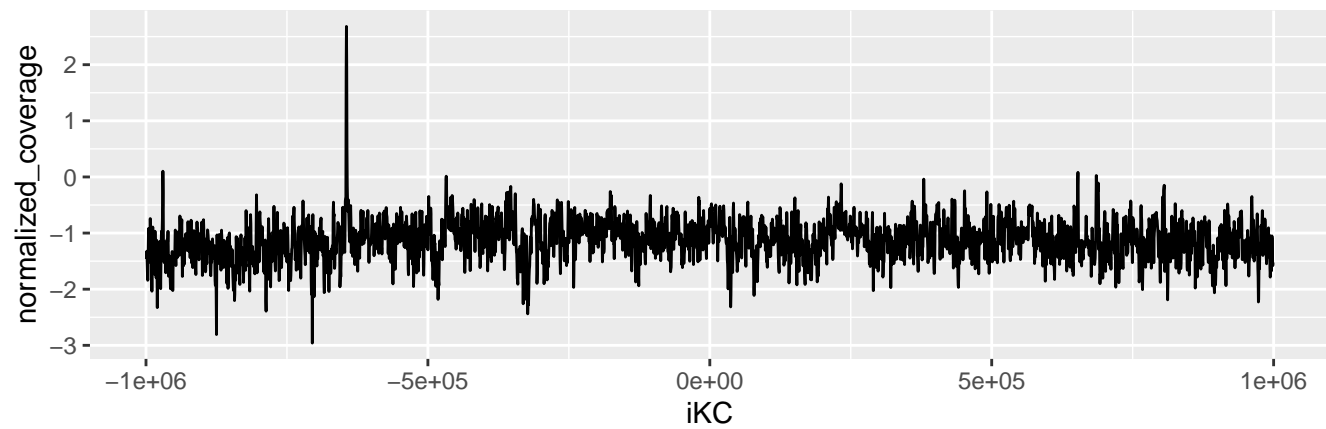

Supplement: Supplementary file 6 — Supplementary Data 3 [file 41467_2024_49400_MOESM6_ESM.zip › Supplementary Data 3/57_offtarget_sites/125-1_2MB/patient1.chrX_69297624_69297646.2MB.pdf]

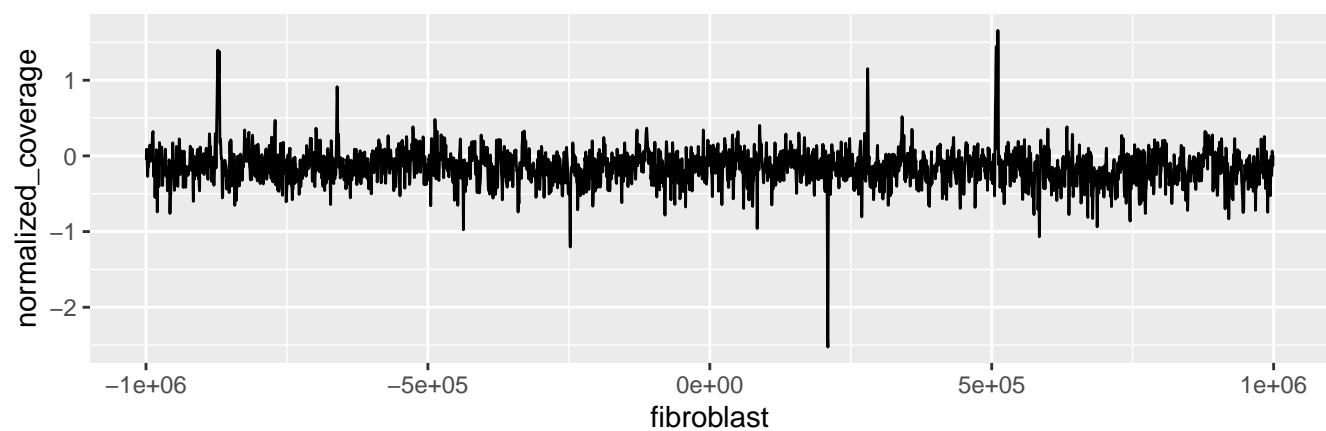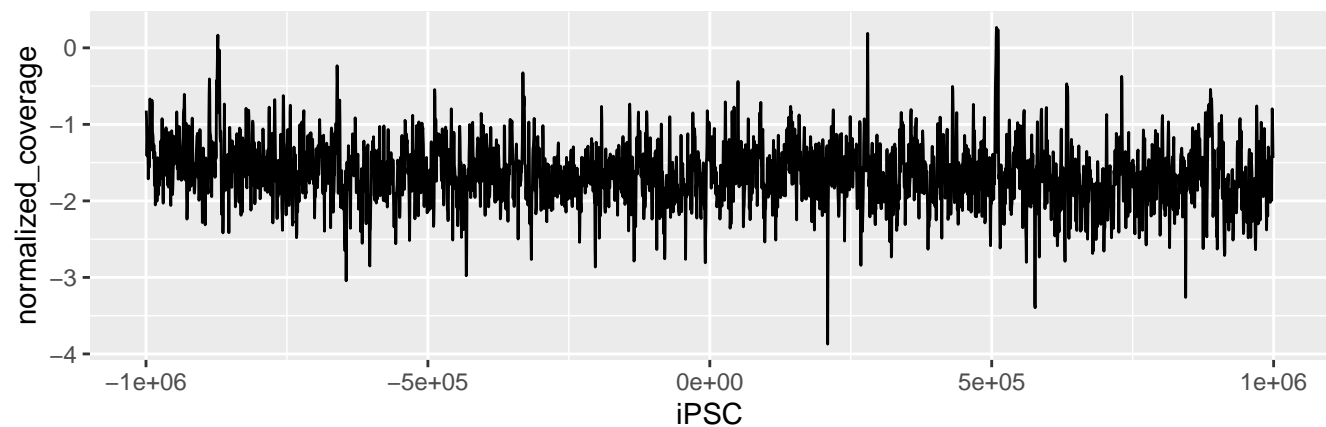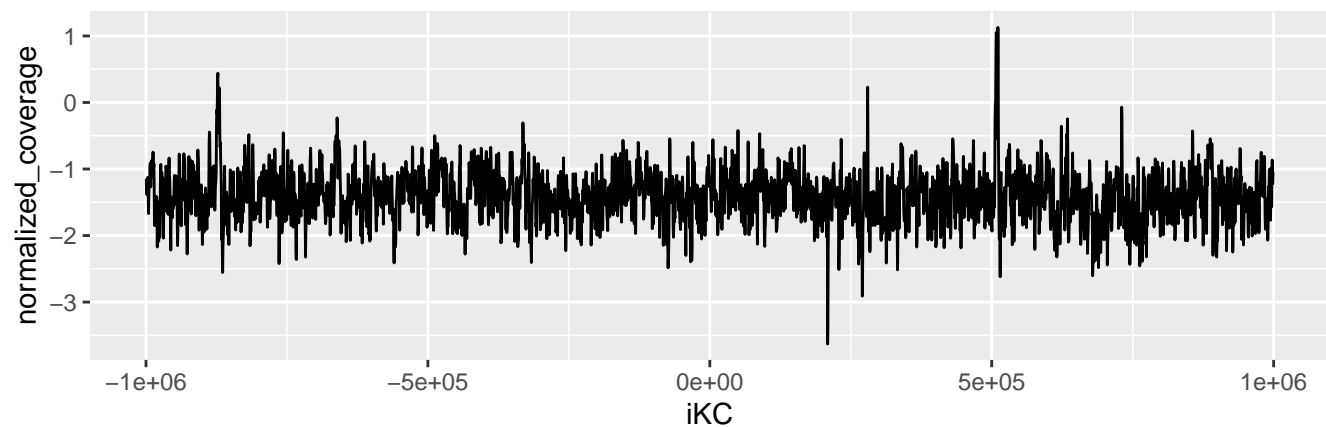

Supplement: Supplementary file 6 — Supplementary Data 3 [file 41467_2024_49400_MOESM6_ESM.zip › Supplementary Data 3/57_offtarget_sites/125-1_2MB/patient1.chrX_88696315_88696337.2MB.pdf]

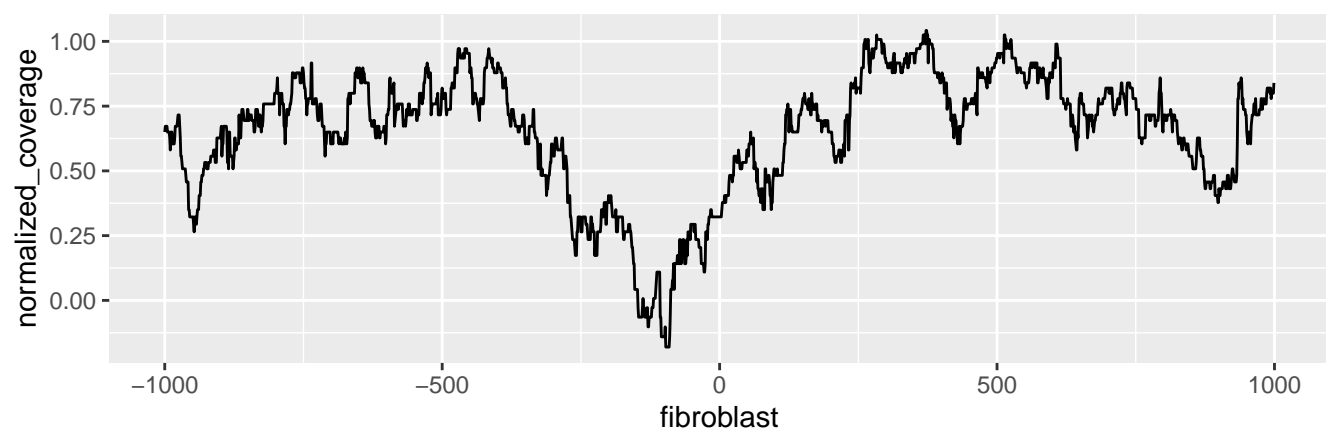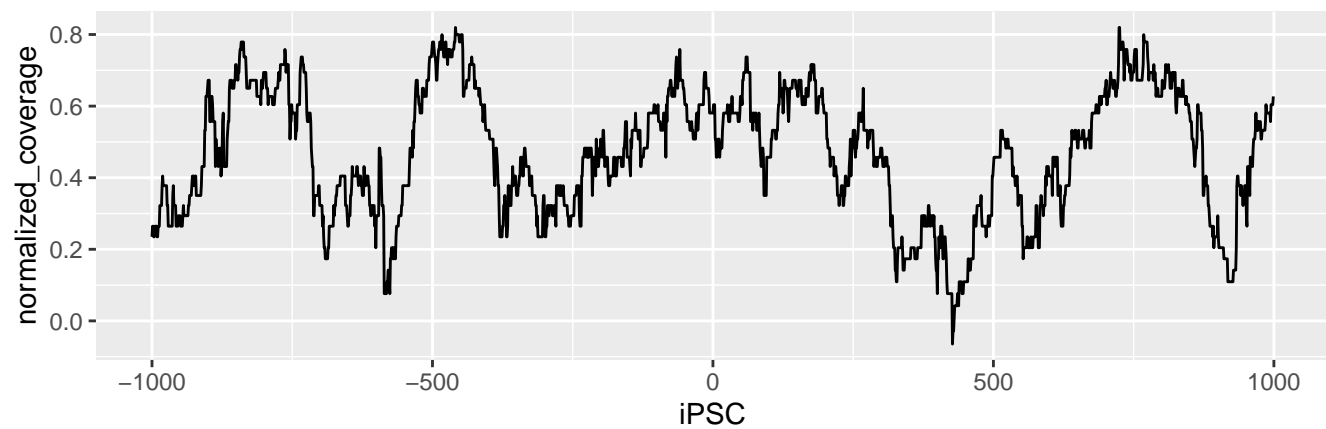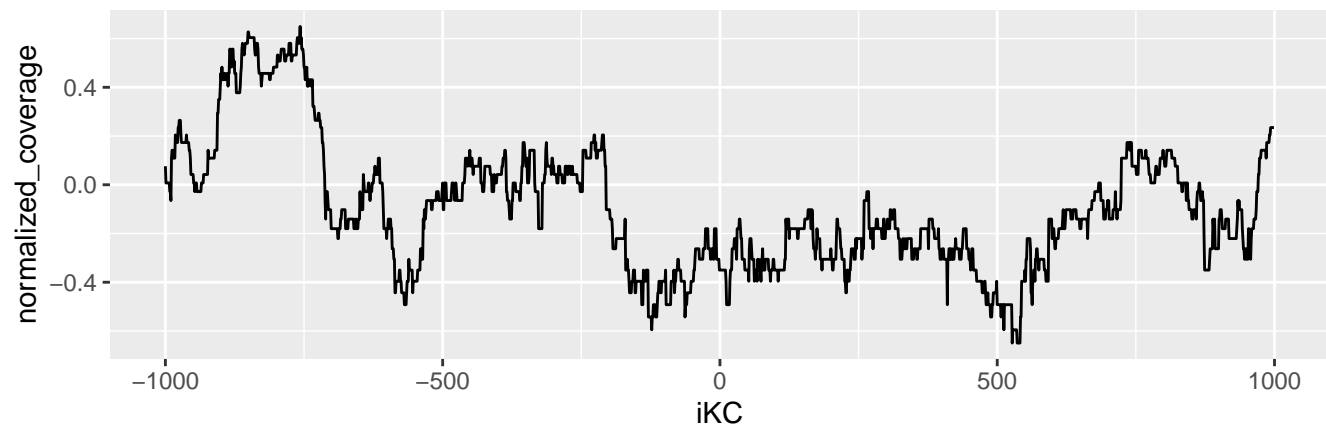

Supplement: Supplementary file 6 — Supplementary Data 3 [file 41467_2024_49400_MOESM6_ESM.zip › Supplementary Data 3/57_offtarget_sites/CO1-131_2KB/patient3_1.chr1_17258328_17258350.2KB.pdf]

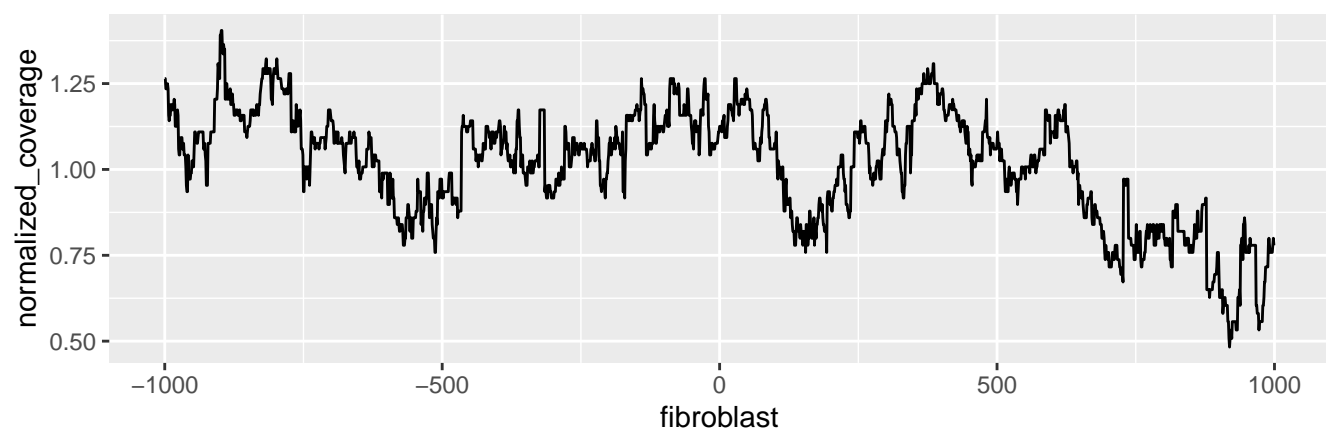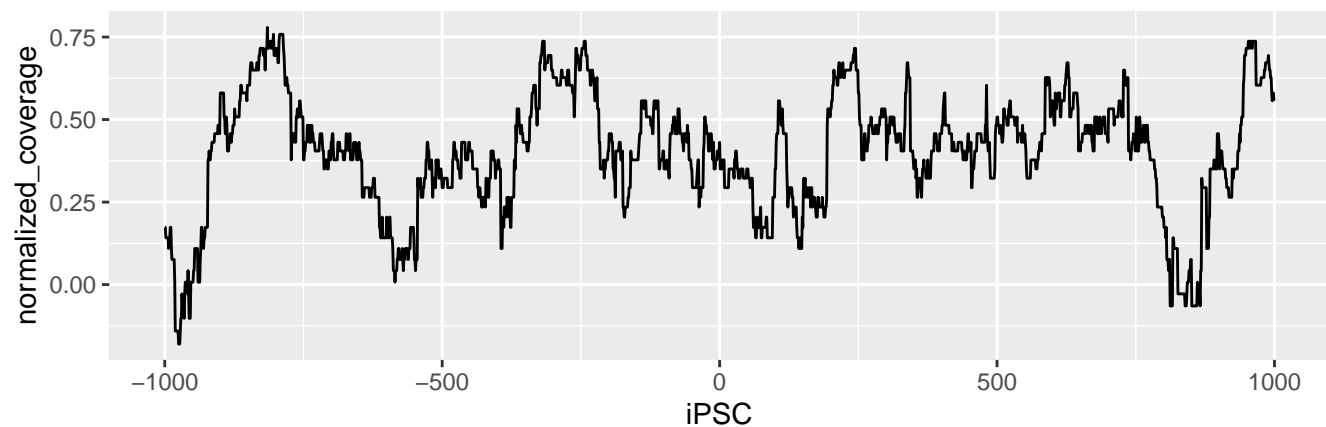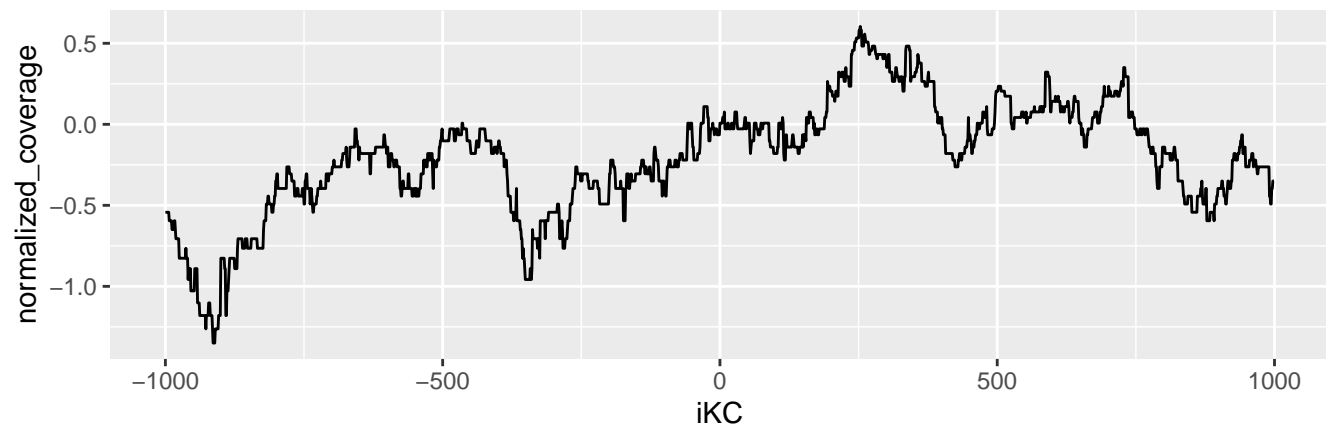

Supplement: Supplementary file 6 — Supplementary Data 3 [file 41467_2024_49400_MOESM6_ESM.zip › Supplementary Data 3/57_offtarget_sites/CO1-131_2KB/patient3_1.chr1_25863728_25863750.2KB.pdf]

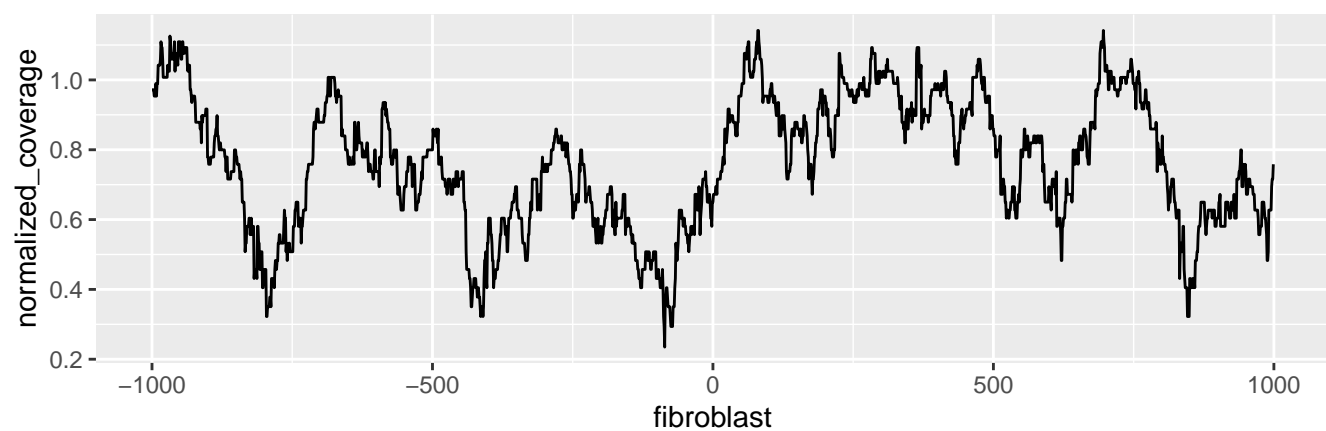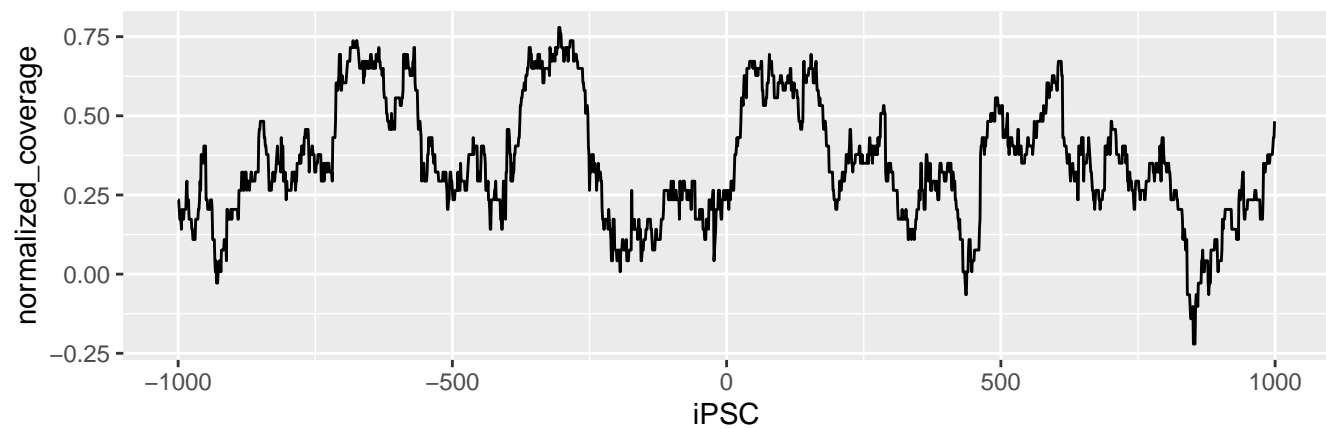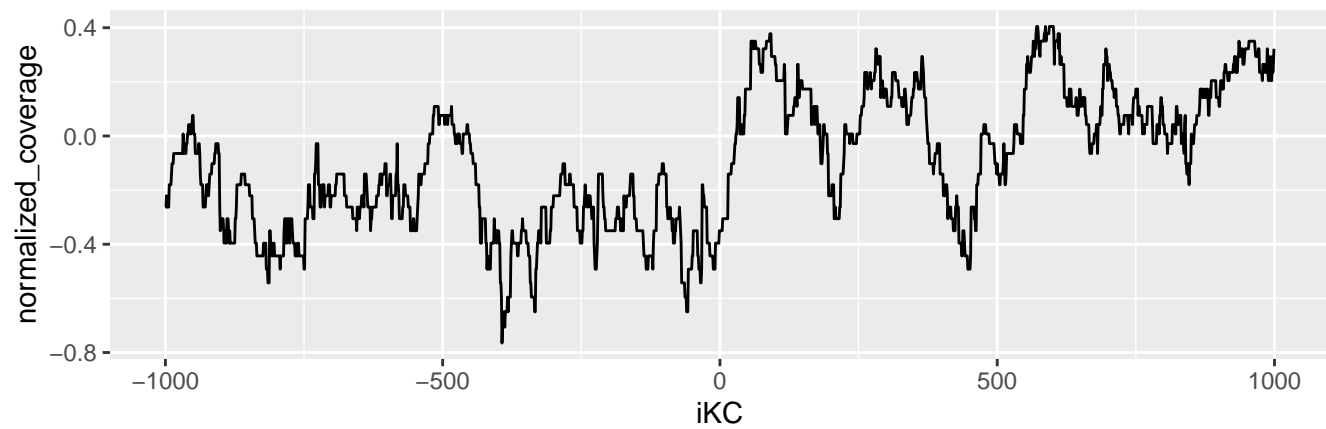

Supplement: Supplementary file 6 — Supplementary Data 3 [file 41467_2024_49400_MOESM6_ESM.zip › Supplementary Data 3/57_offtarget_sites/CO1-131_2KB/patient3_1.chr1_53116163_53116185.2KB.pdf]

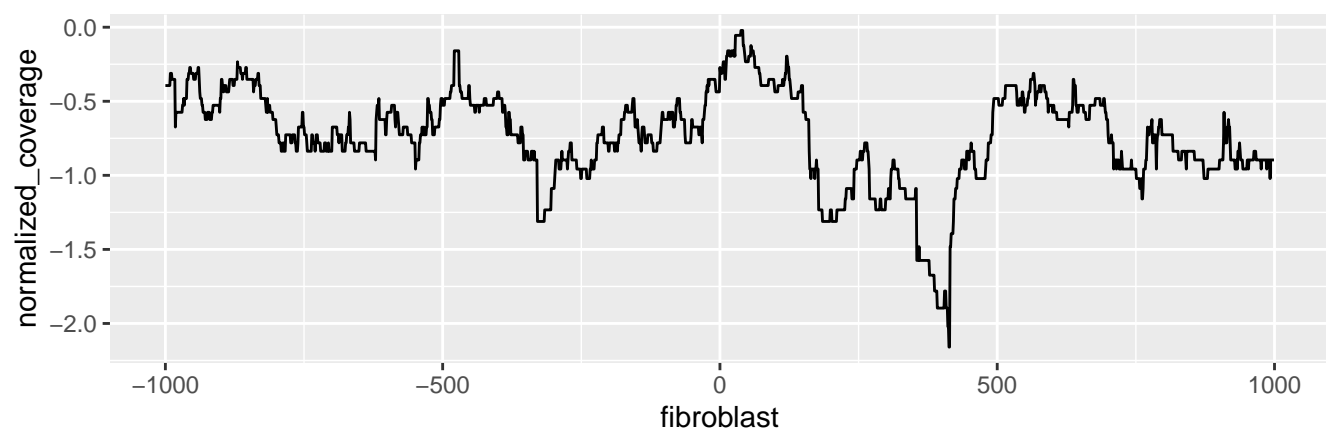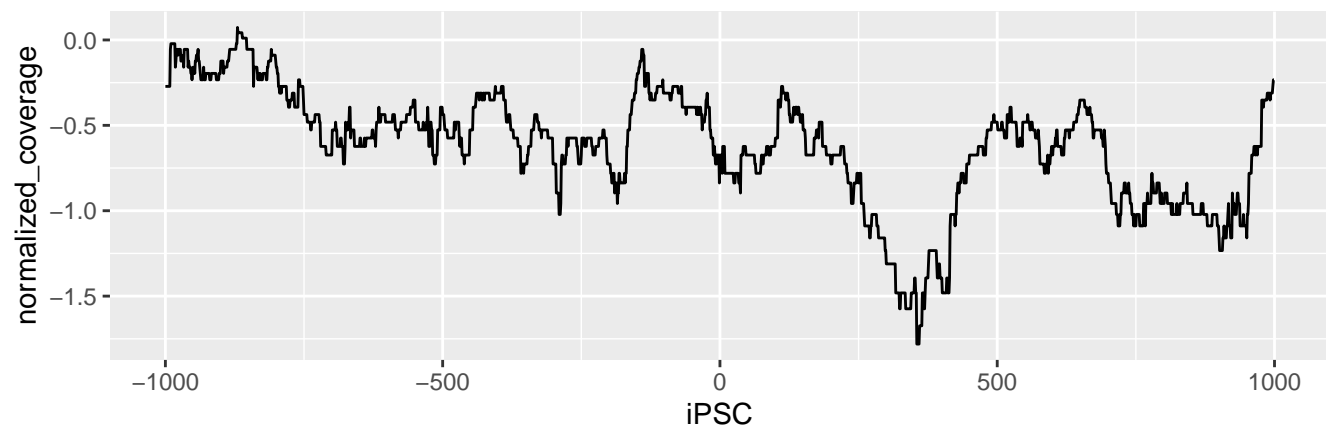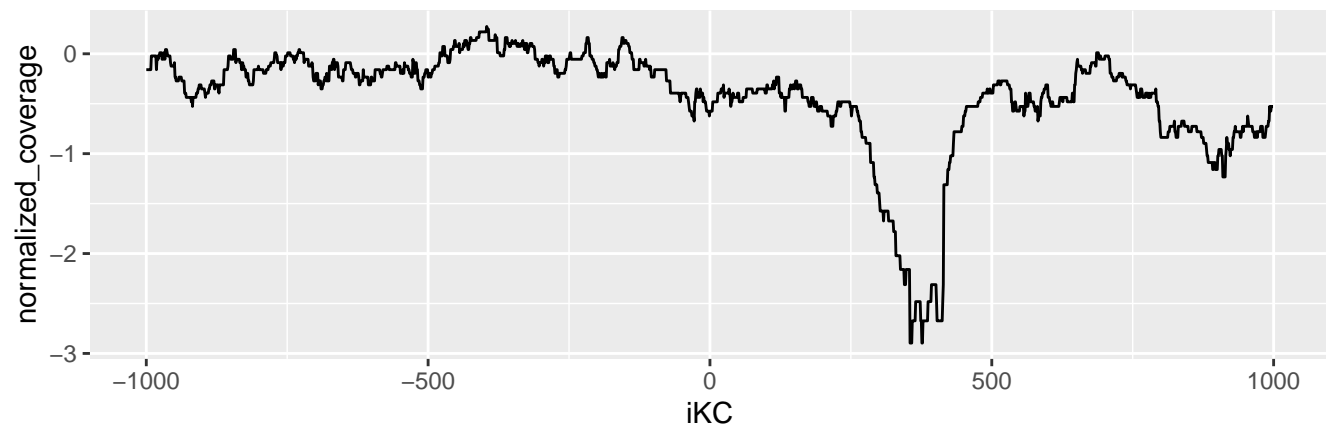

Supplement: Supplementary file 6 — Supplementary Data 3 [file 41467_2024_49400_MOESM6_ESM.zip › Supplementary Data 3/57_offtarget_sites/CO1-131_2KB/patient3_1.chr10_10938785_10938807.2KB.pdf]

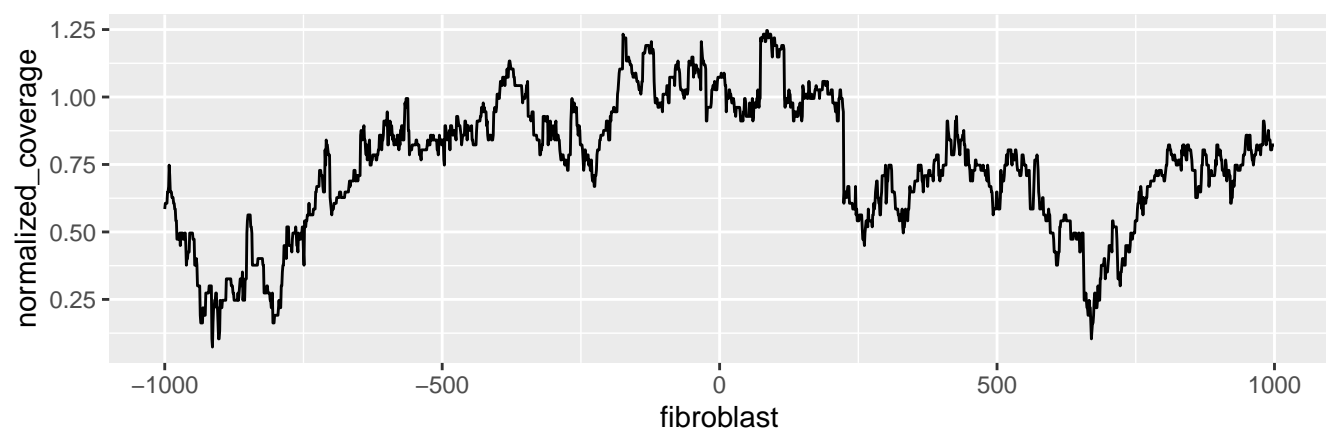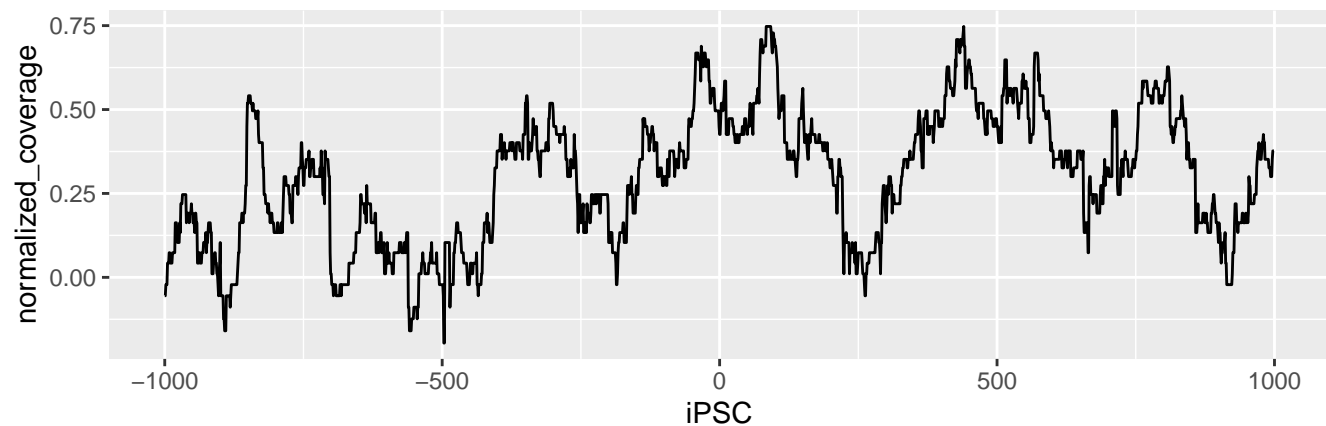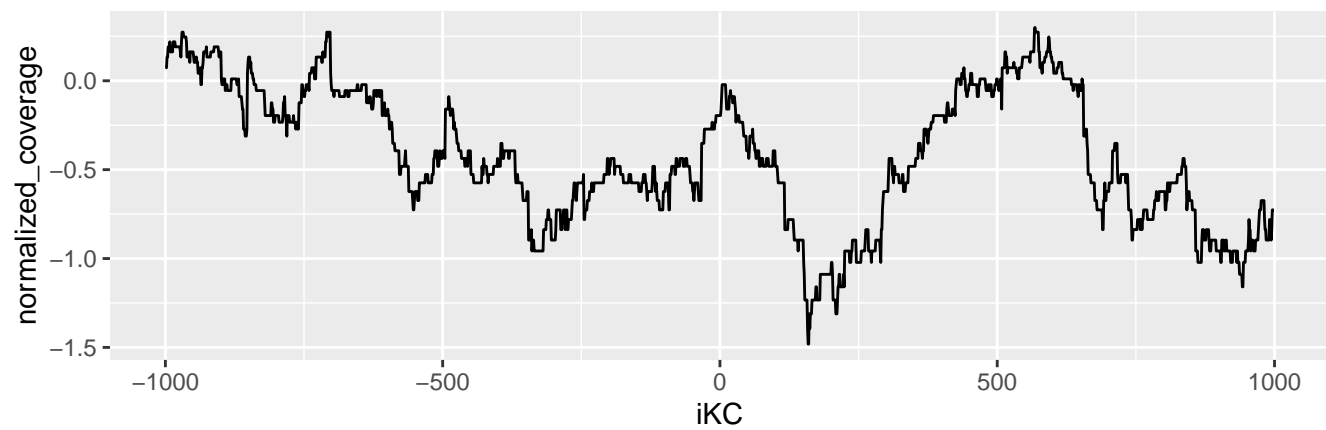

Supplement: Supplementary file 6 — Supplementary Data 3 [file 41467_2024_49400_MOESM6_ESM.zip › Supplementary Data 3/57_offtarget_sites/CO1-131_2KB/patient3_1.chr10_132681079_132681101.2KB.pdf]

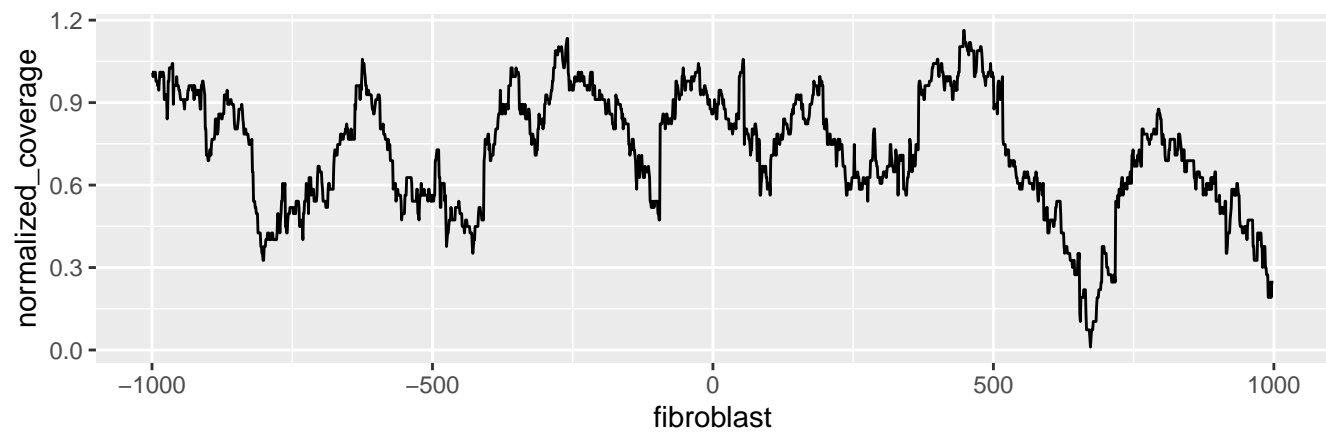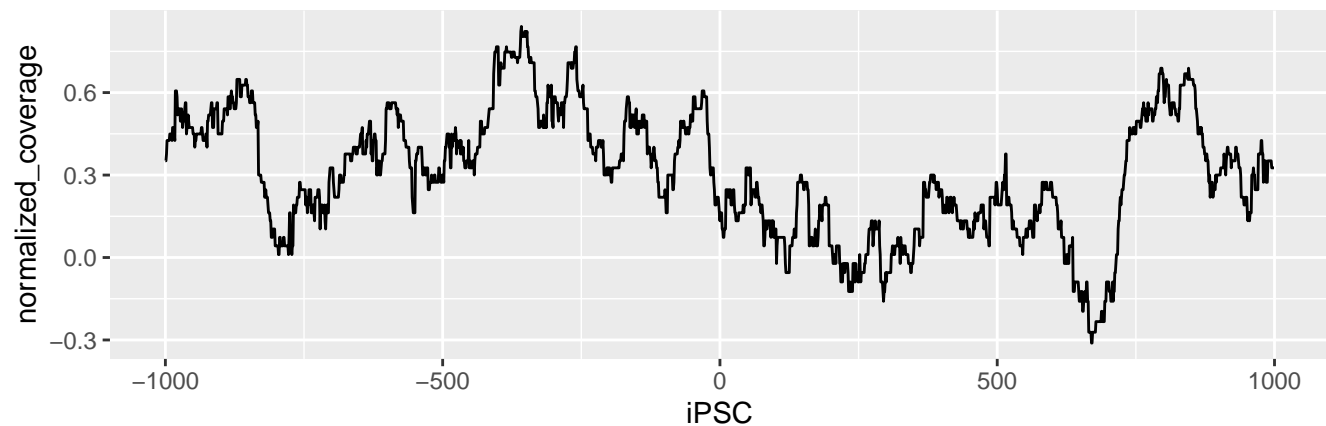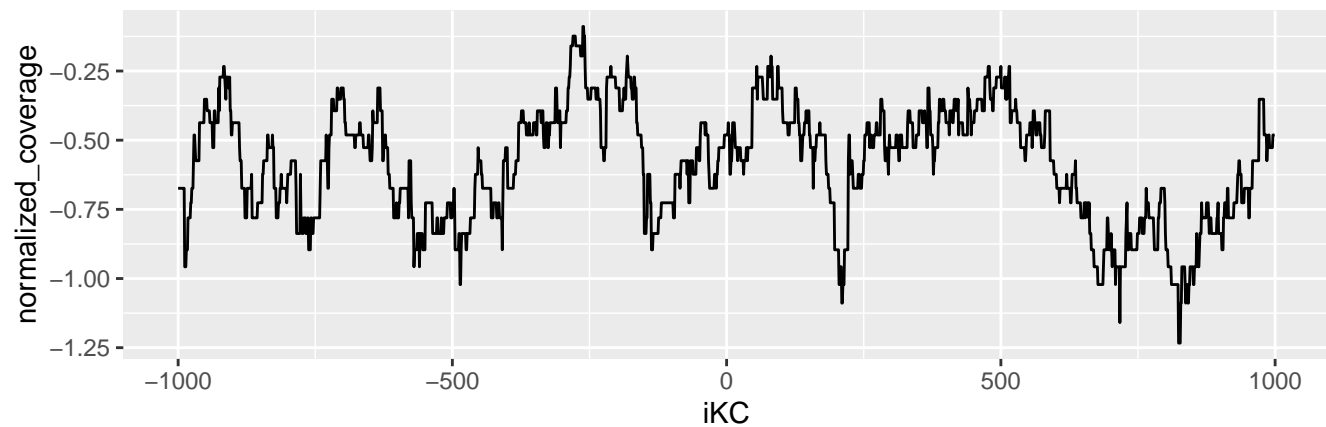

Supplement: Supplementary file 6 — Supplementary Data 3 [file 41467_2024_49400_MOESM6_ESM.zip › Supplementary Data 3/57_offtarget_sites/CO1-131_2KB/patient3_1.chr10_132800880_132800902.2KB.pdf]

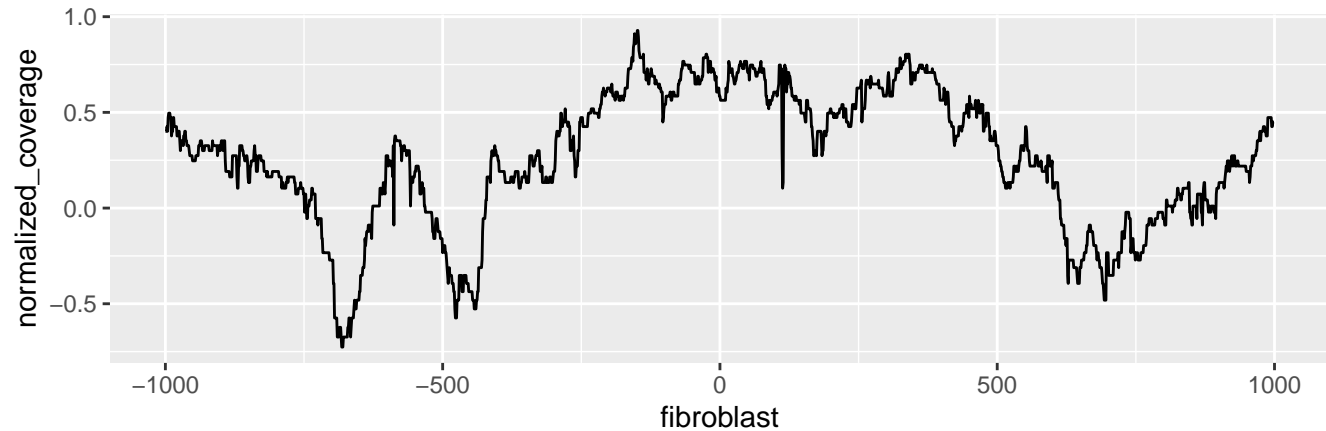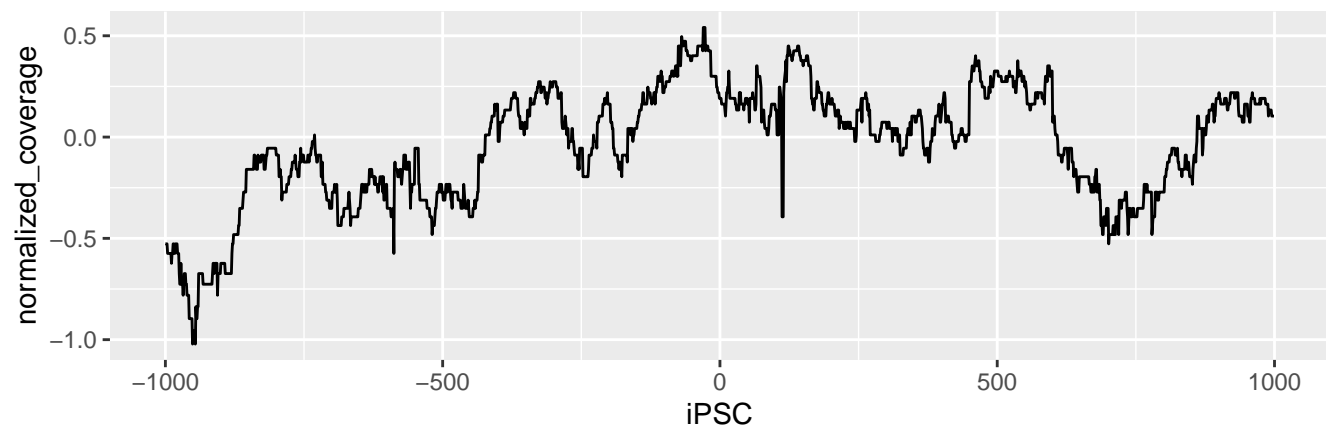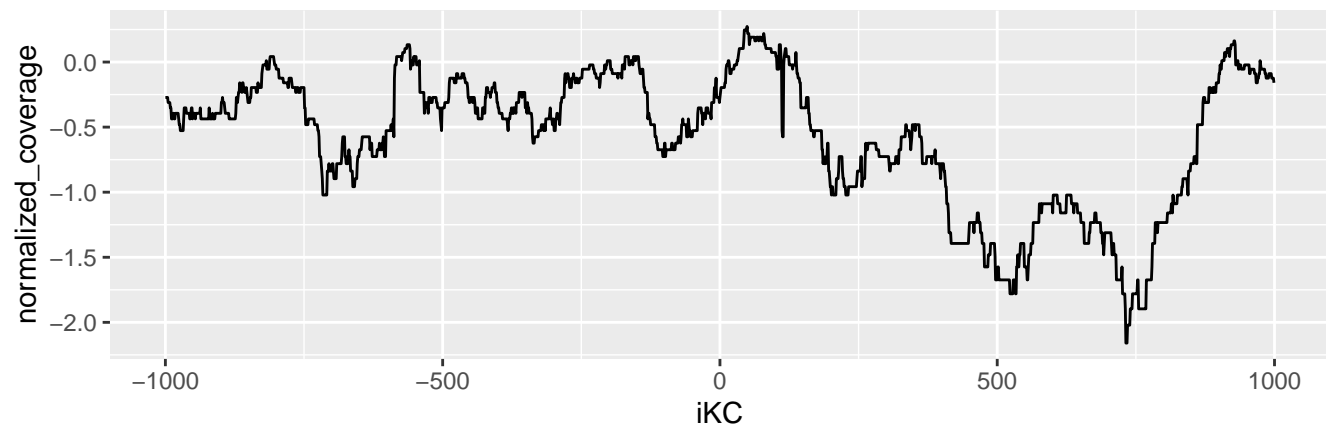

Supplement: Supplementary file 6 — Supplementary Data 3 [file 41467_2024_49400_MOESM6_ESM.zip › Supplementary Data 3/57_offtarget_sites/CO1-131_2KB/patient3_1.chr10_15034690_15034712.2KB.pdf]

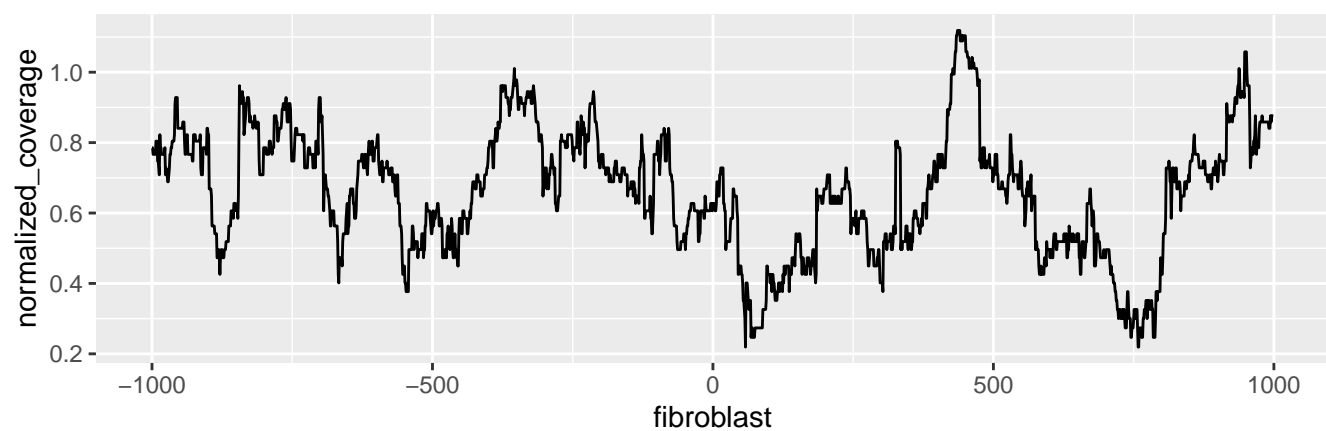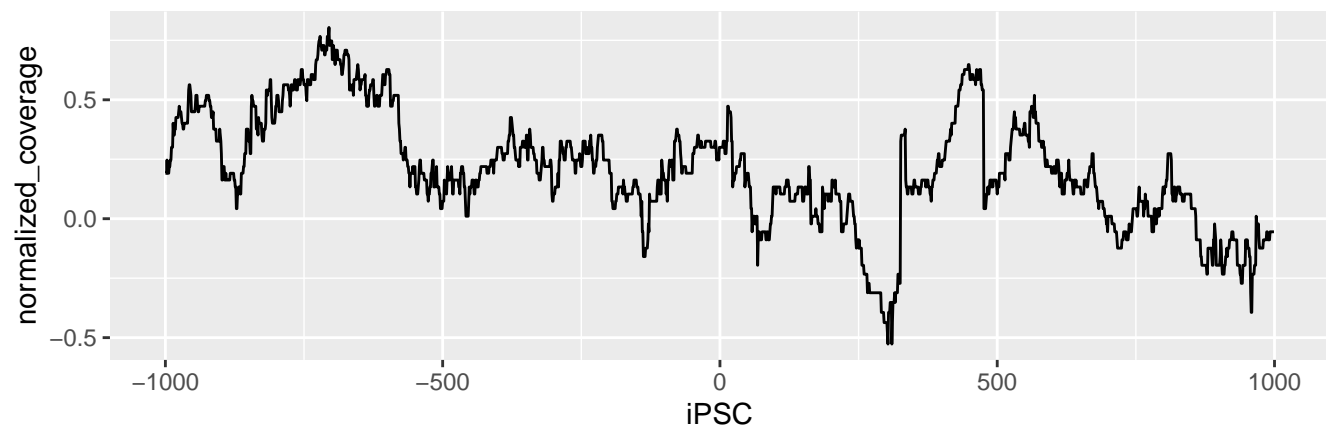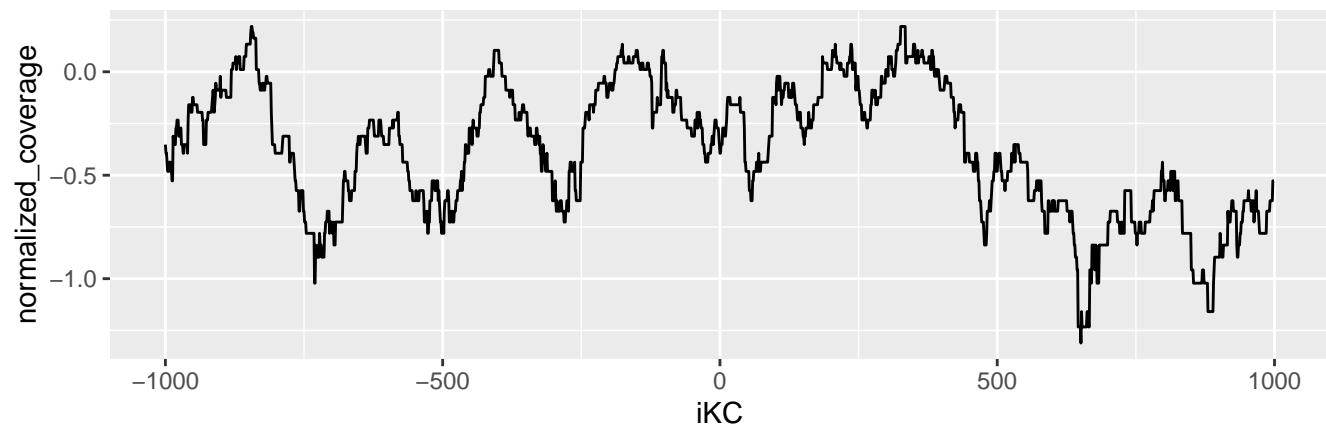

Supplement: Supplementary file 6 — Supplementary Data 3 [file 41467_2024_49400_MOESM6_ESM.zip › Supplementary Data 3/57_offtarget_sites/CO1-131_2KB/patient3_1.chr10_660796_660818.2KB.pdf]

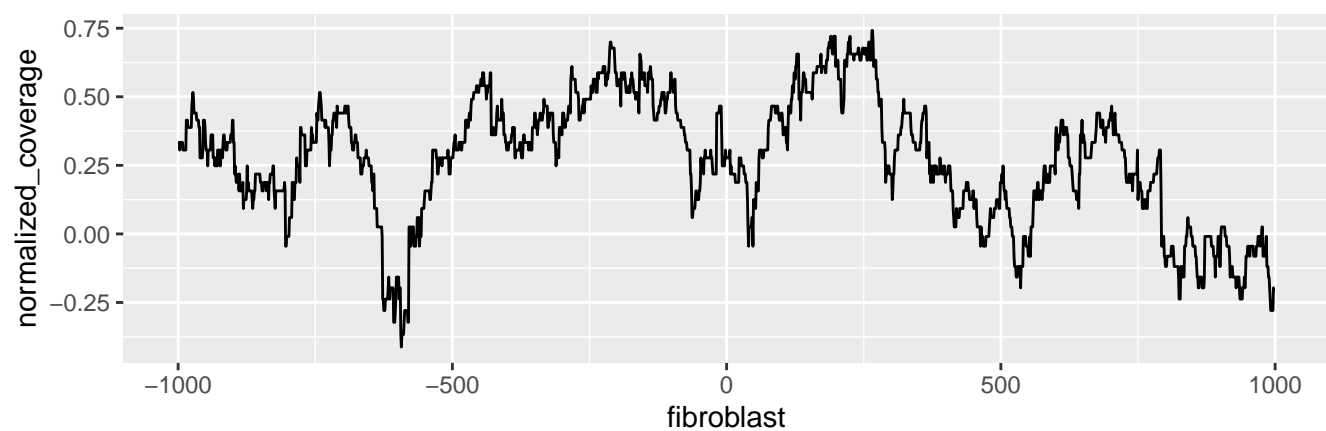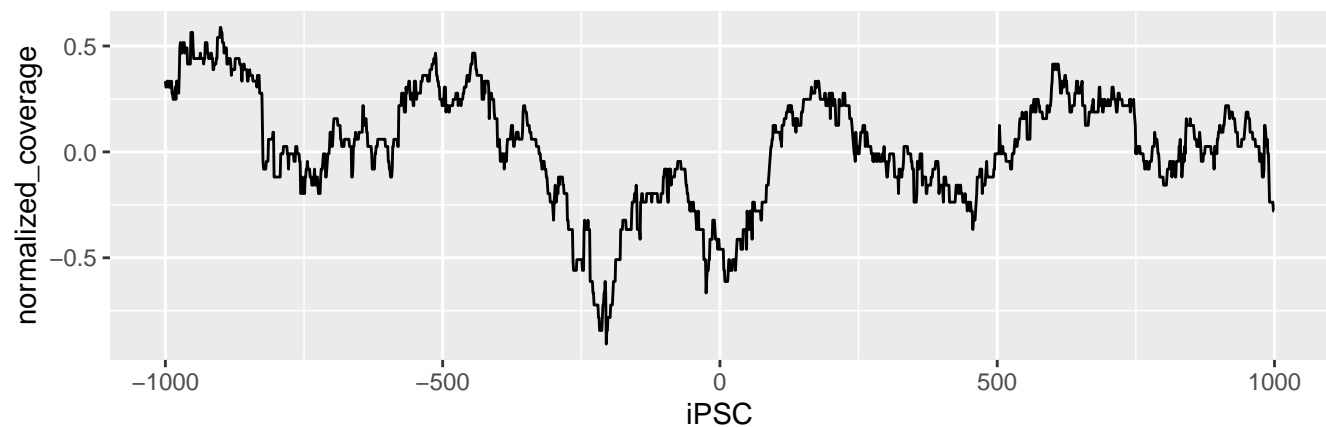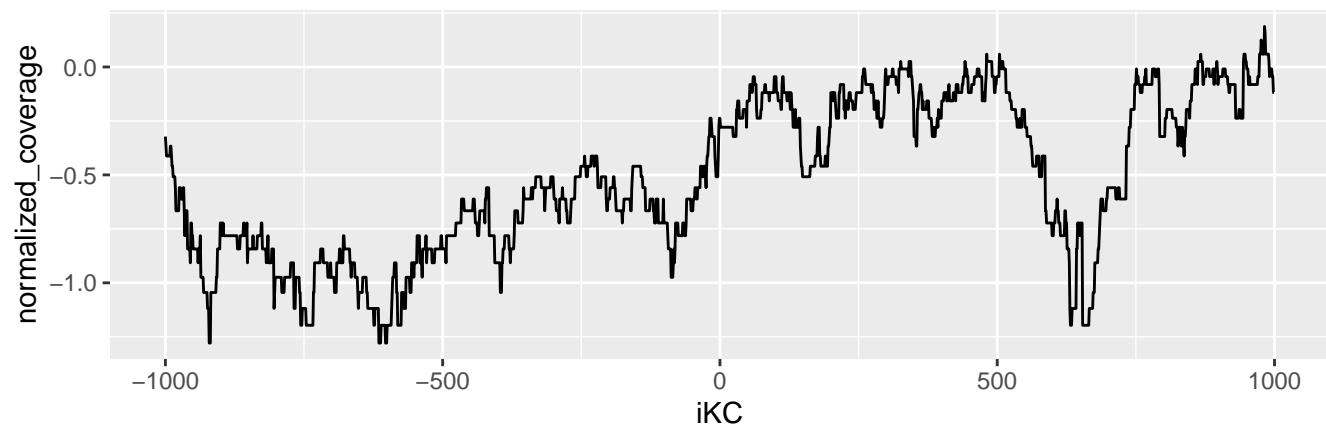

Supplement: Supplementary file 6 — Supplementary Data 3 [file 41467_2024_49400_MOESM6_ESM.zip › Supplementary Data 3/57_offtarget_sites/CO1-131_2KB/patient3_1.chr11_128871452_128871474.2KB.pdf]

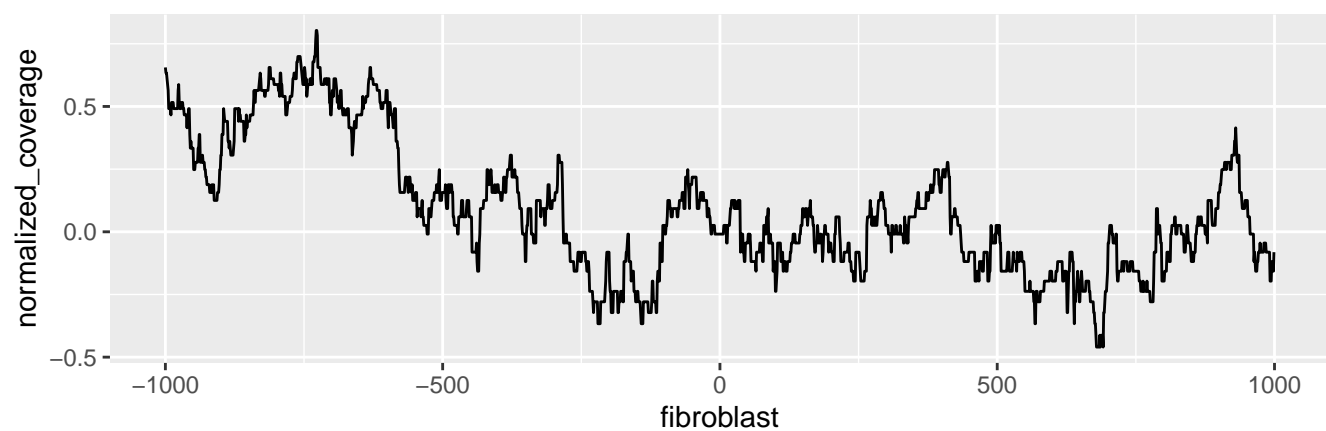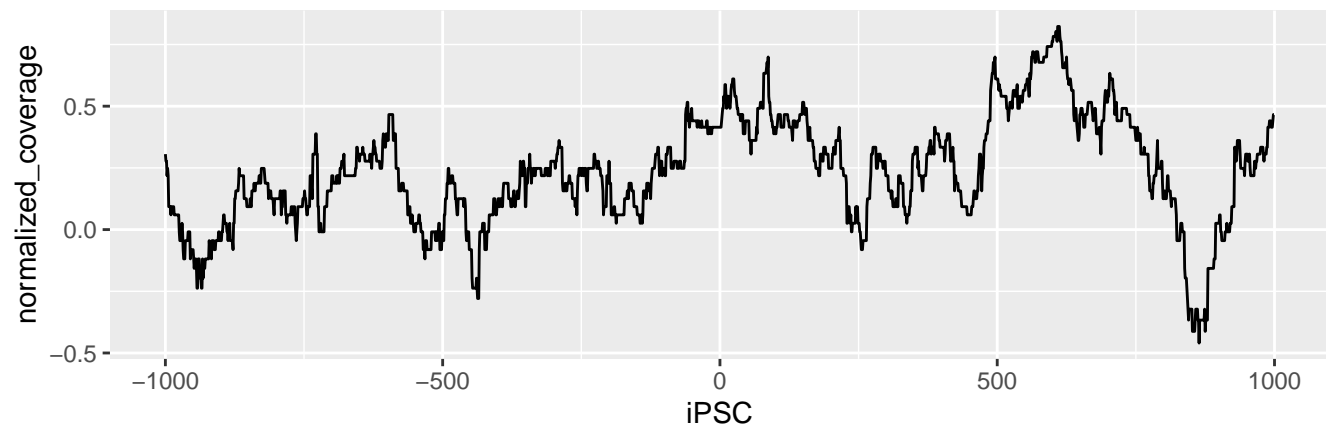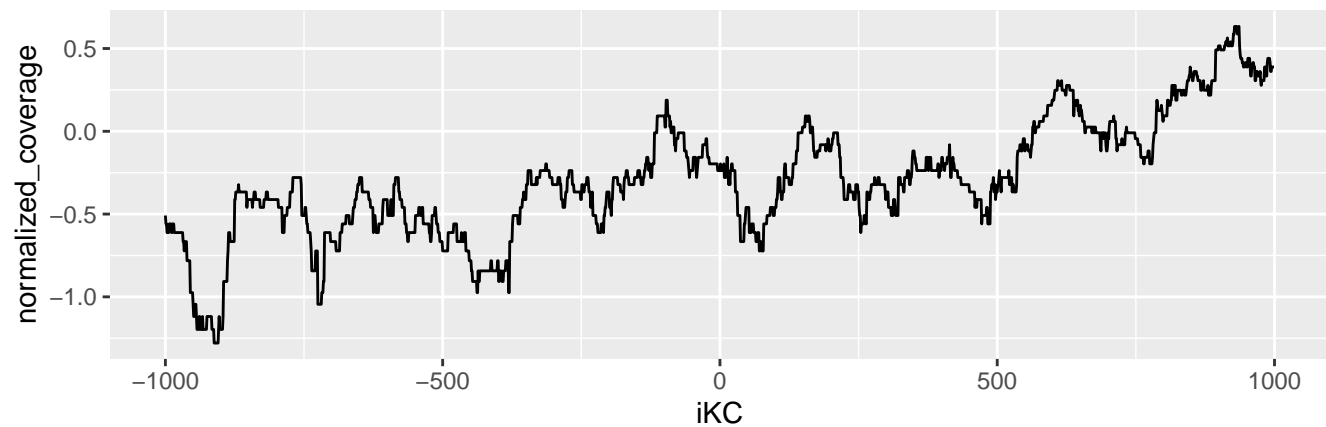

Supplement: Supplementary file 6 — Supplementary Data 3 [file 41467_2024_49400_MOESM6_ESM.zip › Supplementary Data 3/57_offtarget_sites/CO1-131_2KB/patient3_1.chr11_4168526_4168548.2KB.pdf]

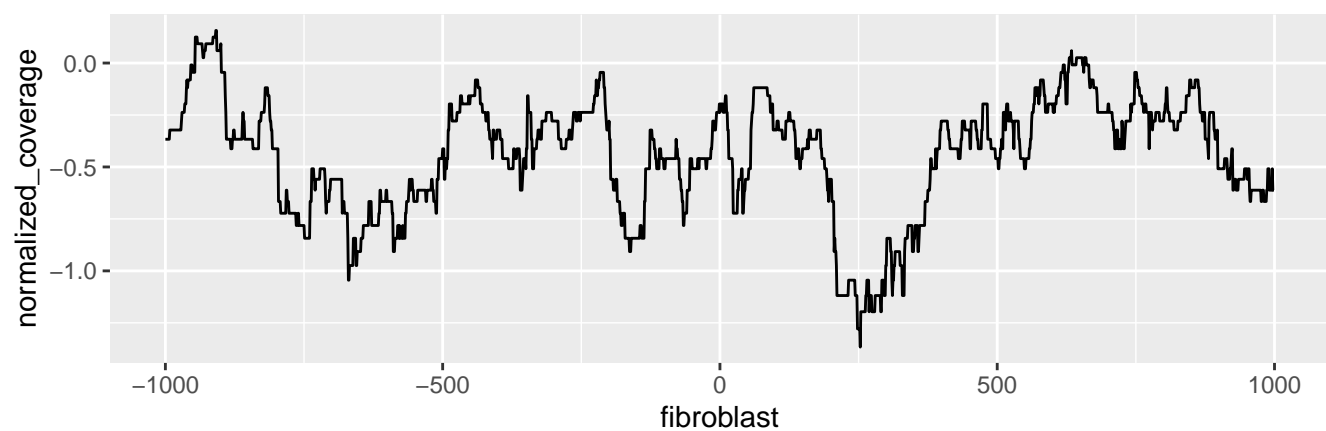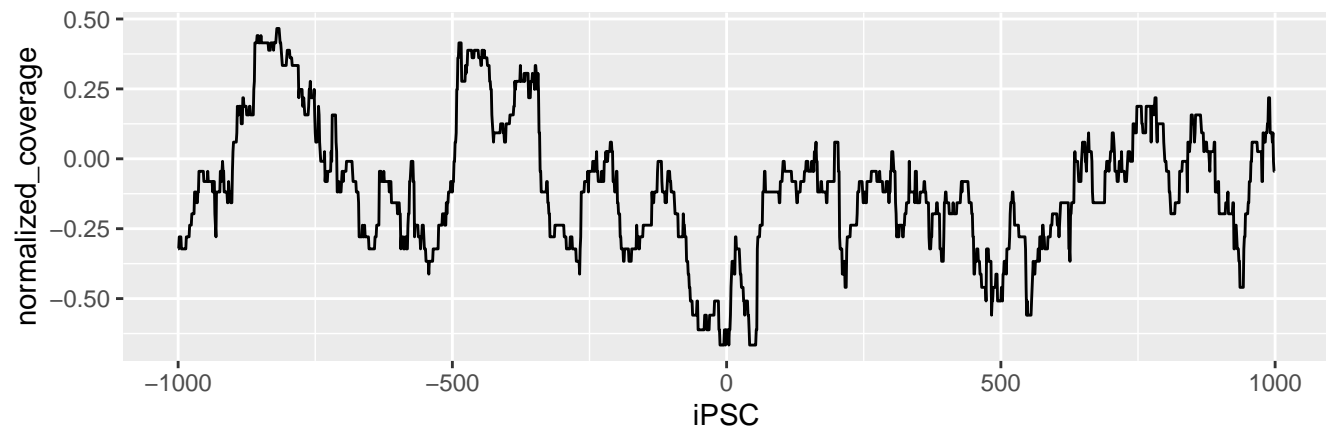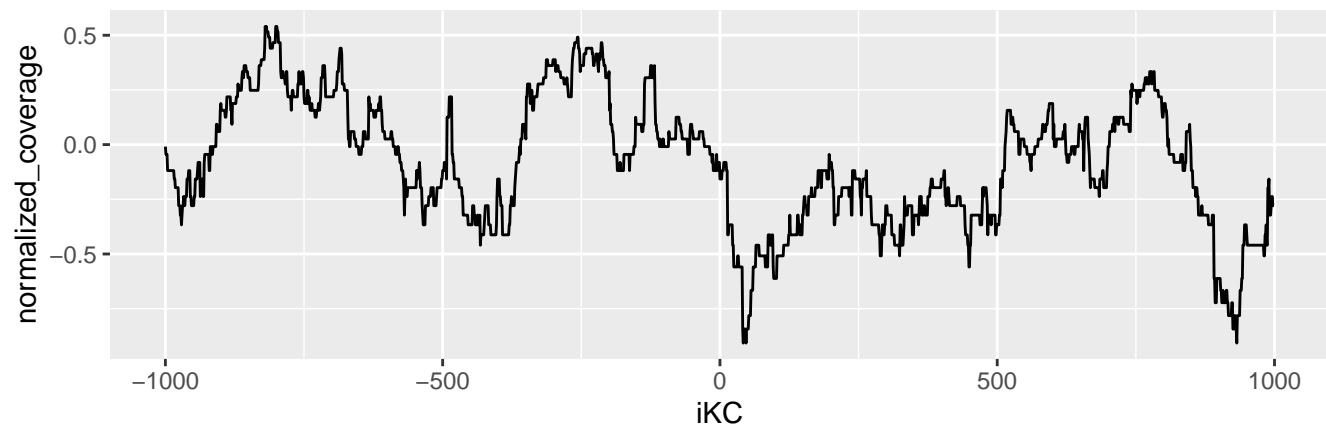

Supplement: Supplementary file 6 — Supplementary Data 3 [file 41467_2024_49400_MOESM6_ESM.zip › Supplementary Data 3/57_offtarget_sites/CO1-131_2KB/patient3_1.chr11_60159330_60159352.2KB.pdf]

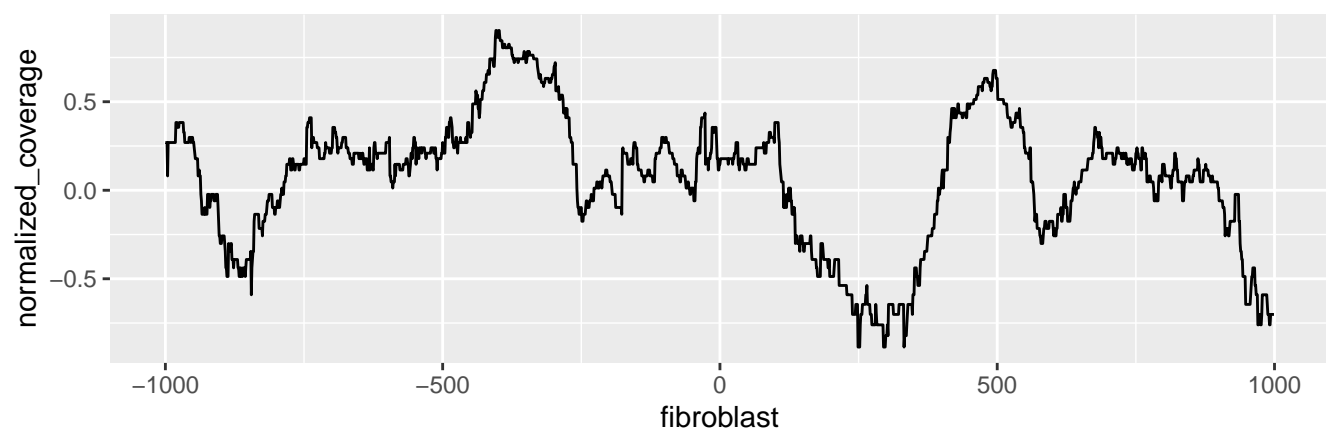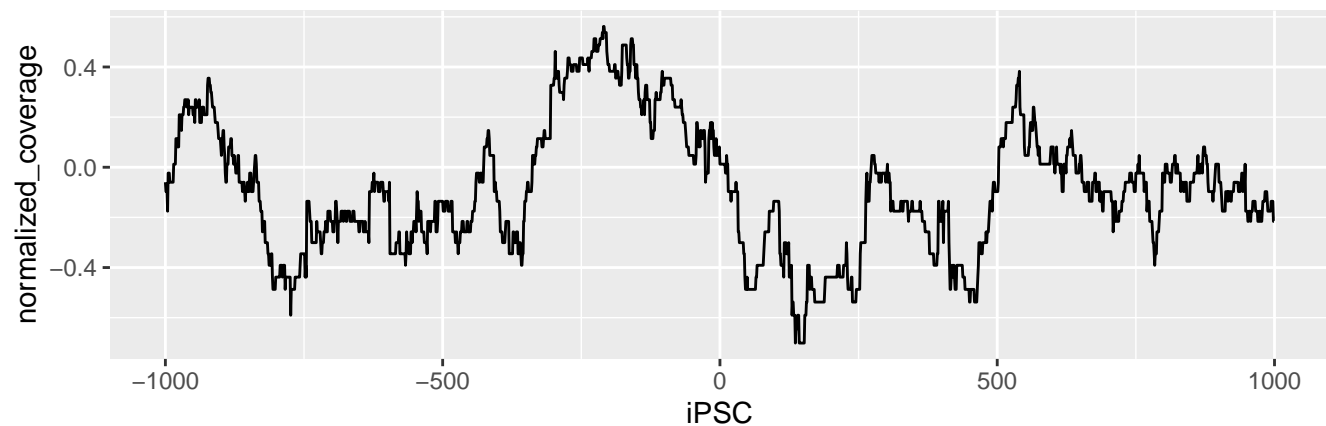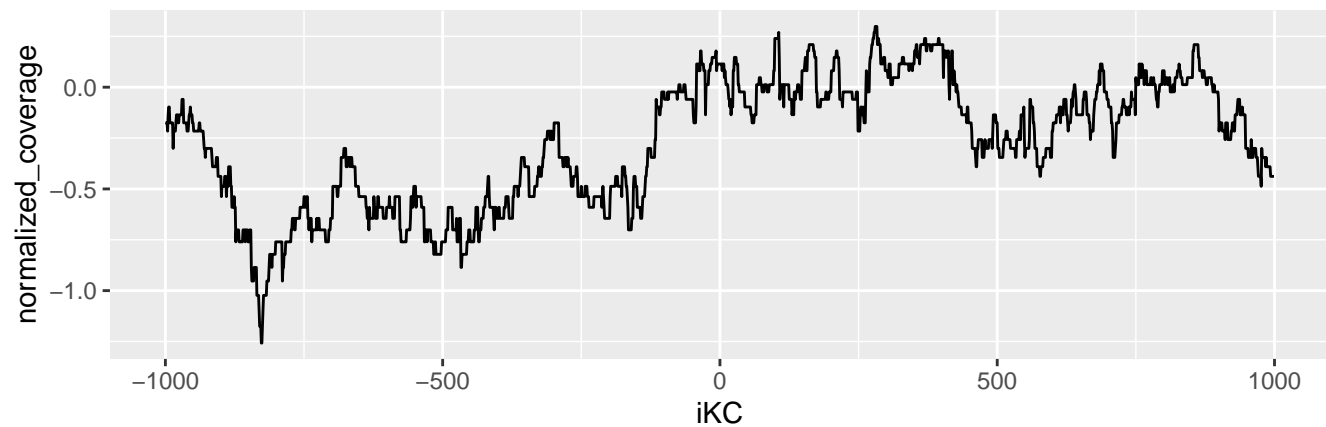

Supplement: Supplementary file 6 — Supplementary Data 3 [file 41467_2024_49400_MOESM6_ESM.zip › Supplementary Data 3/57_offtarget_sites/CO1-131_2KB/patient3_1.chr12_12267463_12267485.2KB.pdf]

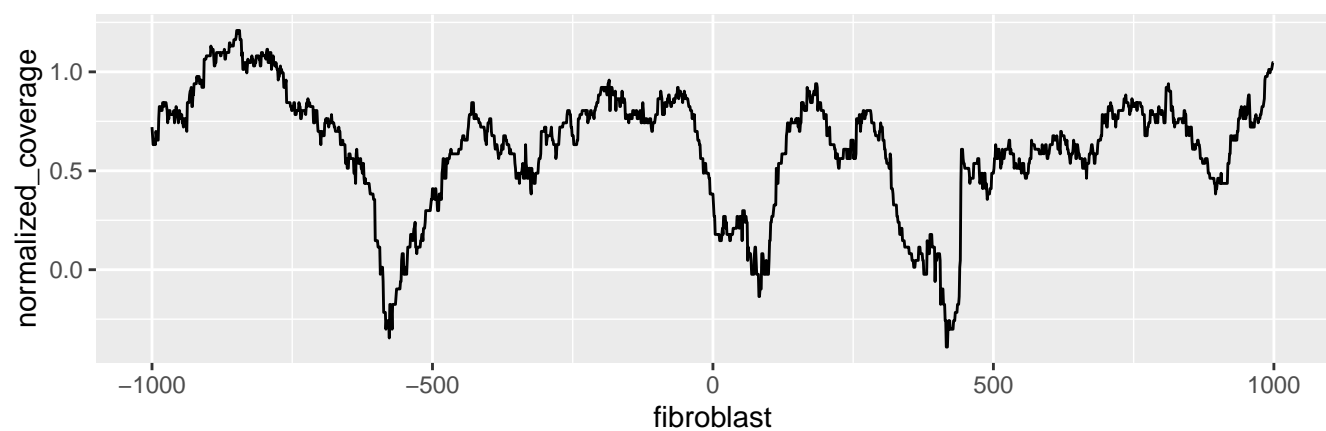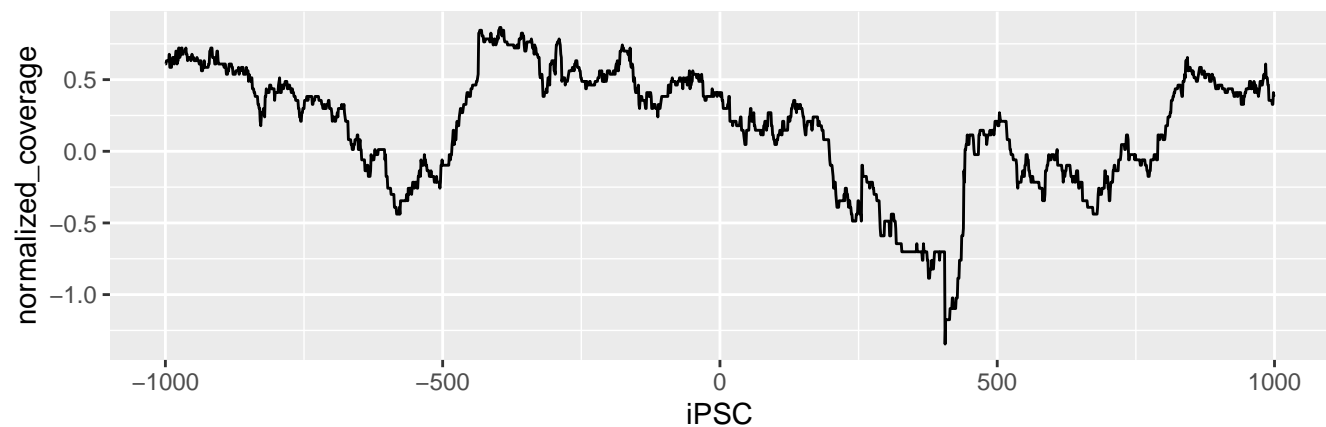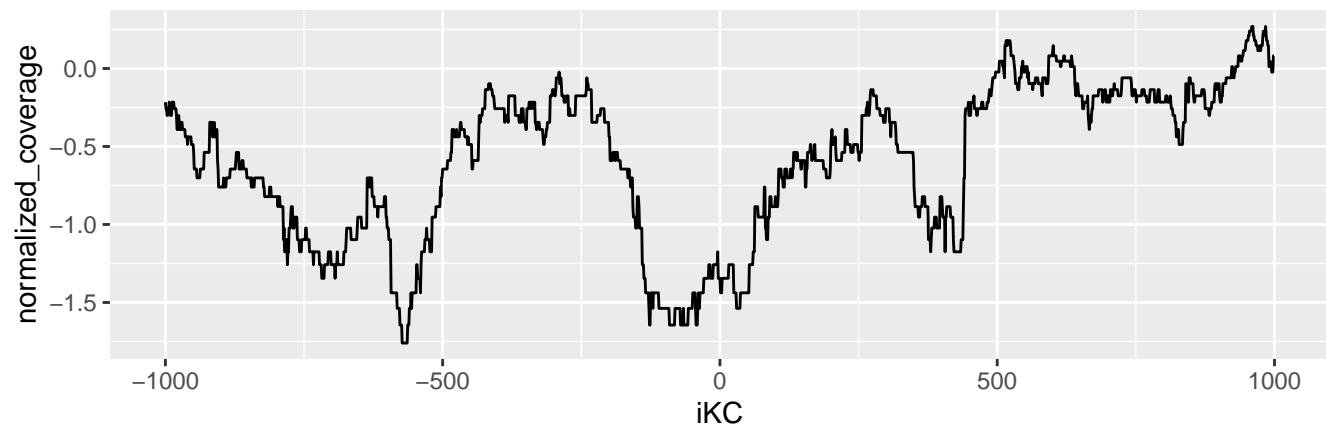

Supplement: Supplementary file 6 — Supplementary Data 3 [file 41467_2024_49400_MOESM6_ESM.zip › Supplementary Data 3/57_offtarget_sites/CO1-131_2KB/patient3_1.chr12_54391229_54391251.2KB.pdf]

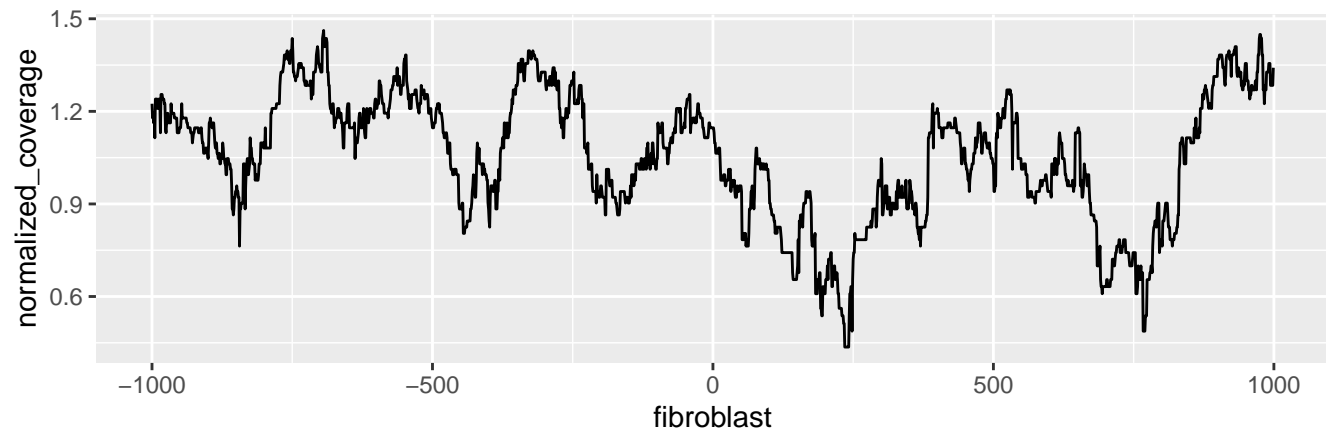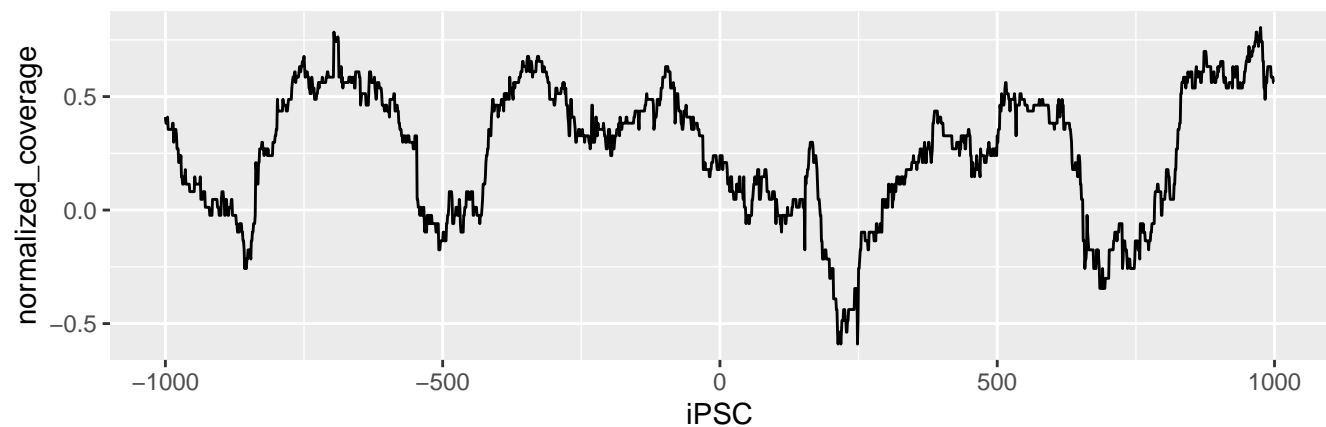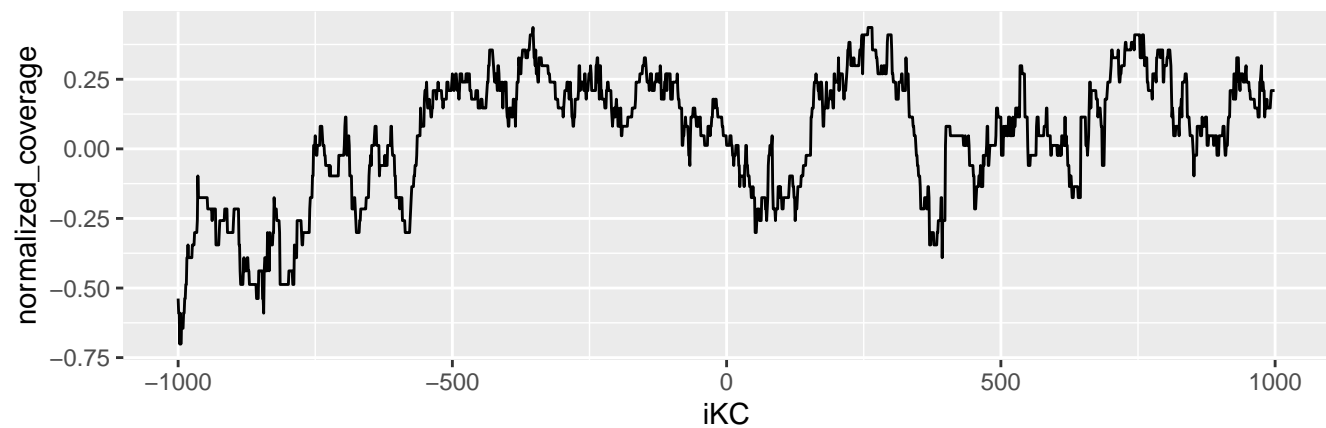

Supplement: Supplementary file 6 — Supplementary Data 3 [file 41467_2024_49400_MOESM6_ESM.zip › Supplementary Data 3/57_offtarget_sites/CO1-131_2KB/patient3_1.chr12_57490628_57490650.2KB.pdf]

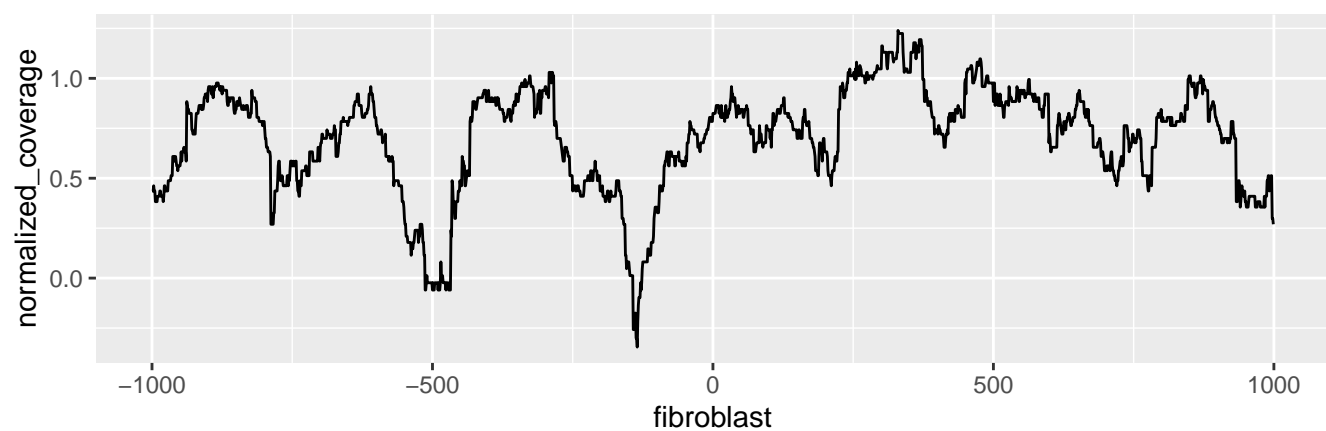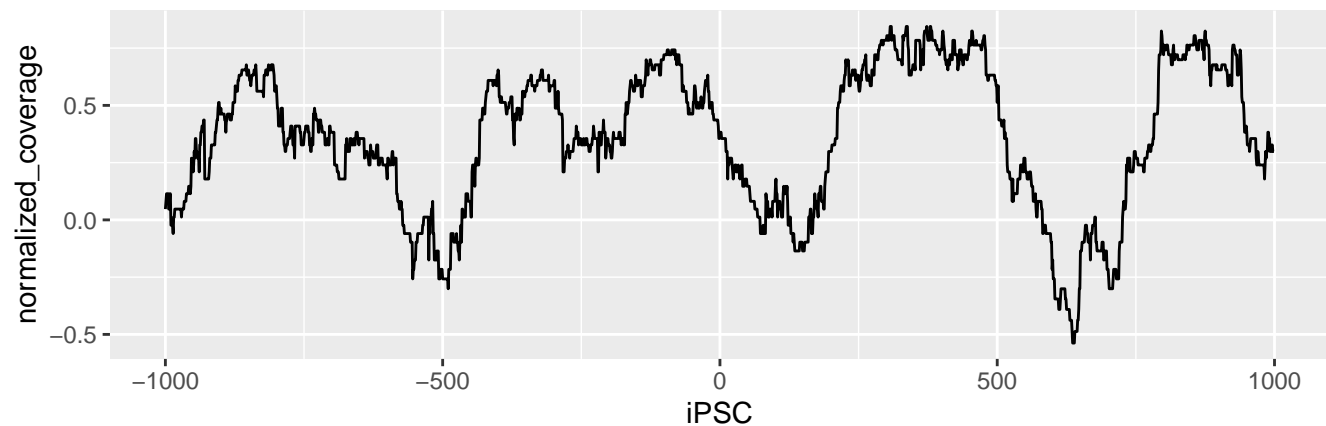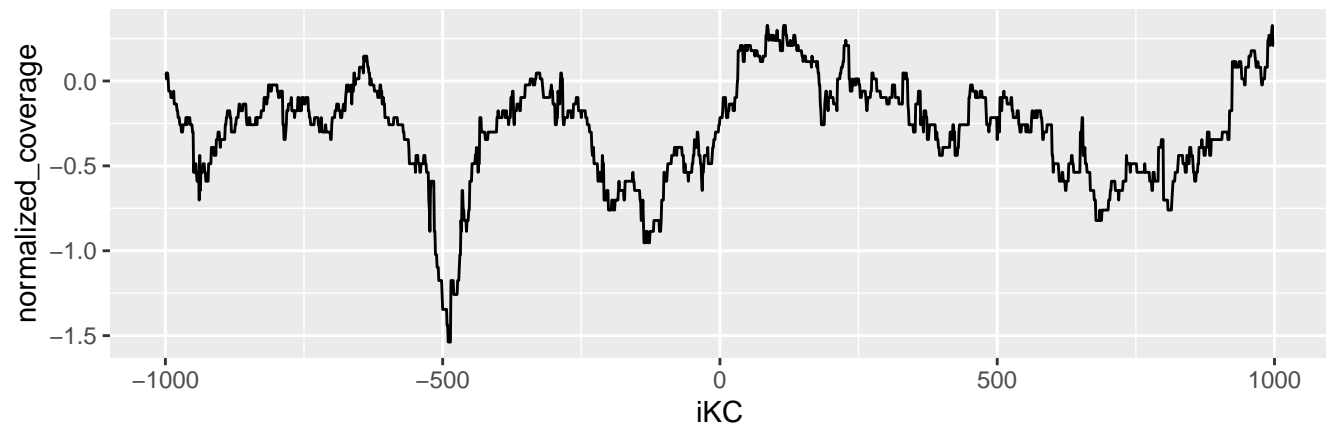

Supplement: Supplementary file 6 — Supplementary Data 3 [file 41467_2024_49400_MOESM6_ESM.zip › Supplementary Data 3/57_offtarget_sites/CO1-131_2KB/patient3_1.chr12_6075570_6075592.2KB.pdf]

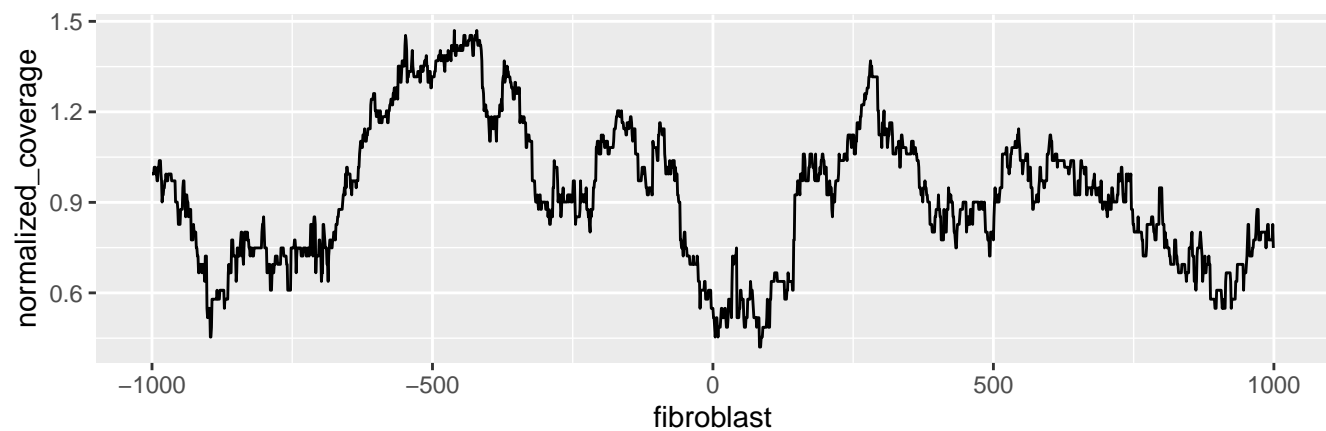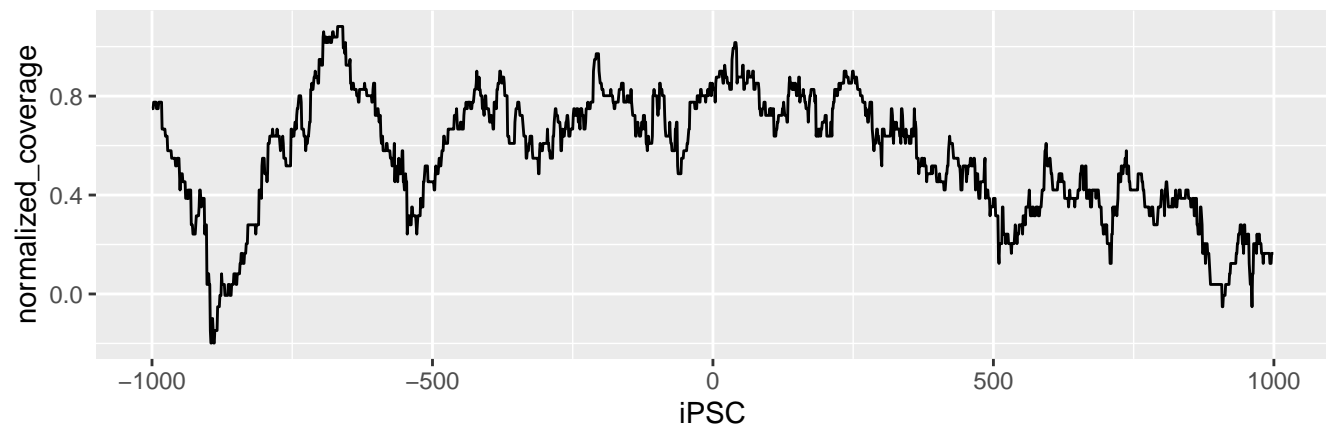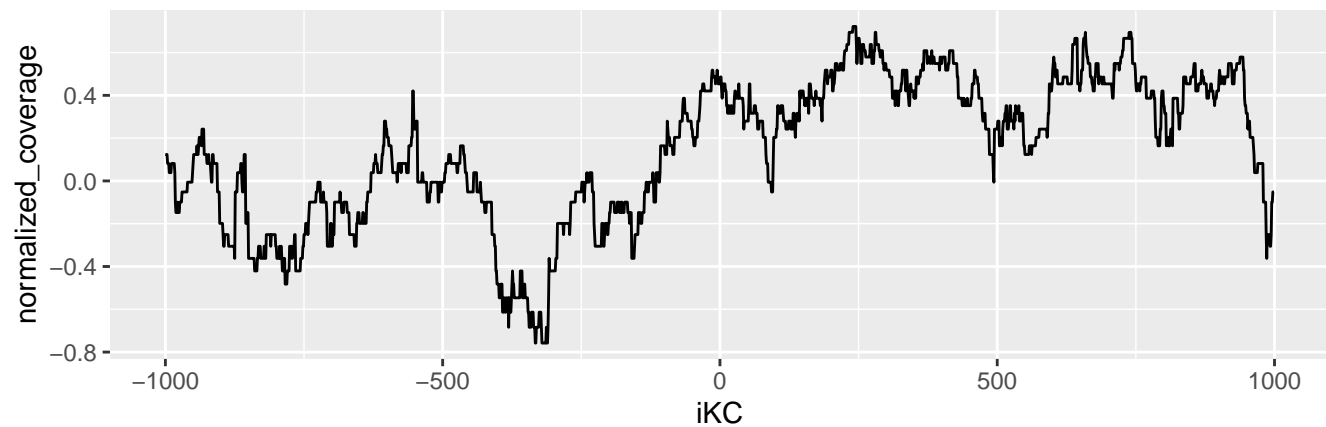

Supplement: Supplementary file 6 — Supplementary Data 3 [file 41467_2024_49400_MOESM6_ESM.zip › Supplementary Data 3/57_offtarget_sites/CO1-131_2KB/patient3_1.chr14_100299494_100299516.2KB.pdf]

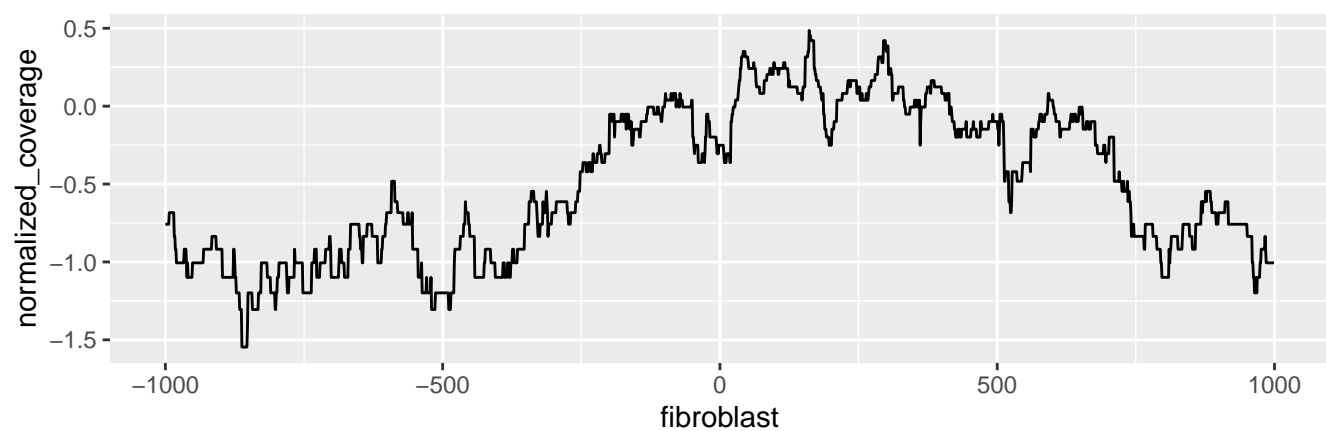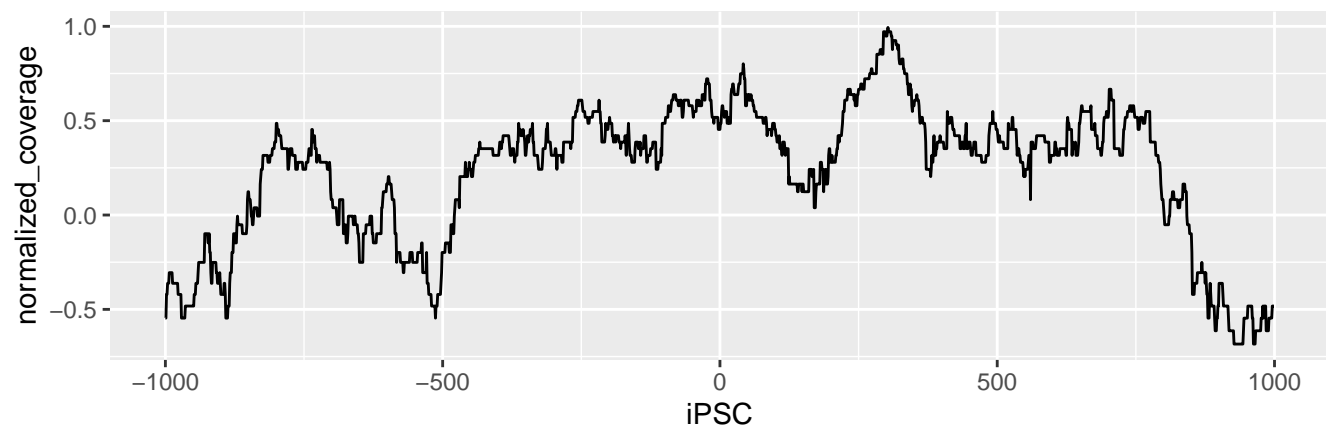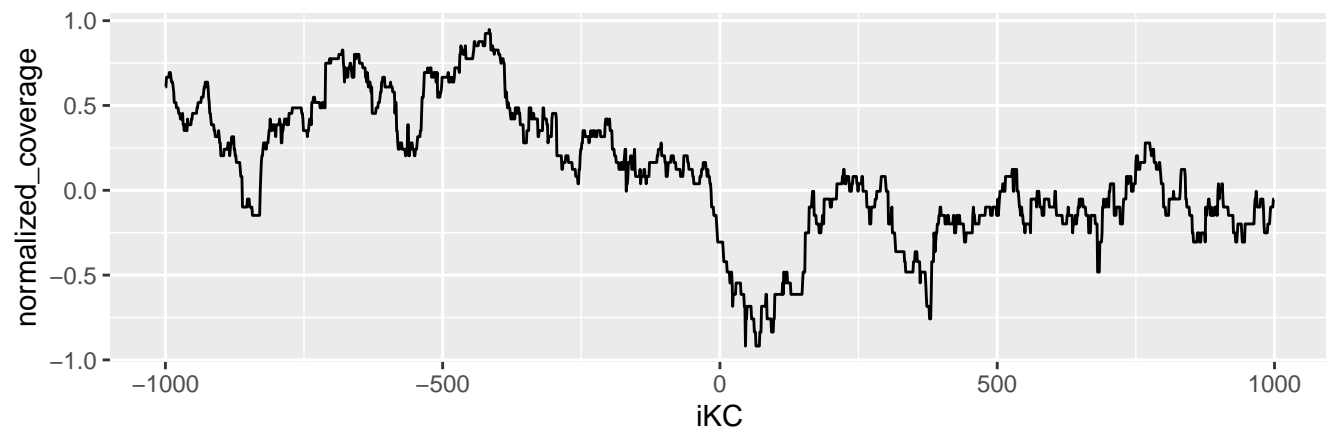

Supplement: Supplementary file 6 — Supplementary Data 3 [file 41467_2024_49400_MOESM6_ESM.zip › Supplementary Data 3/57_offtarget_sites/CO1-131_2KB/patient3_1.chr14_47984104_47984126.2KB.pdf]

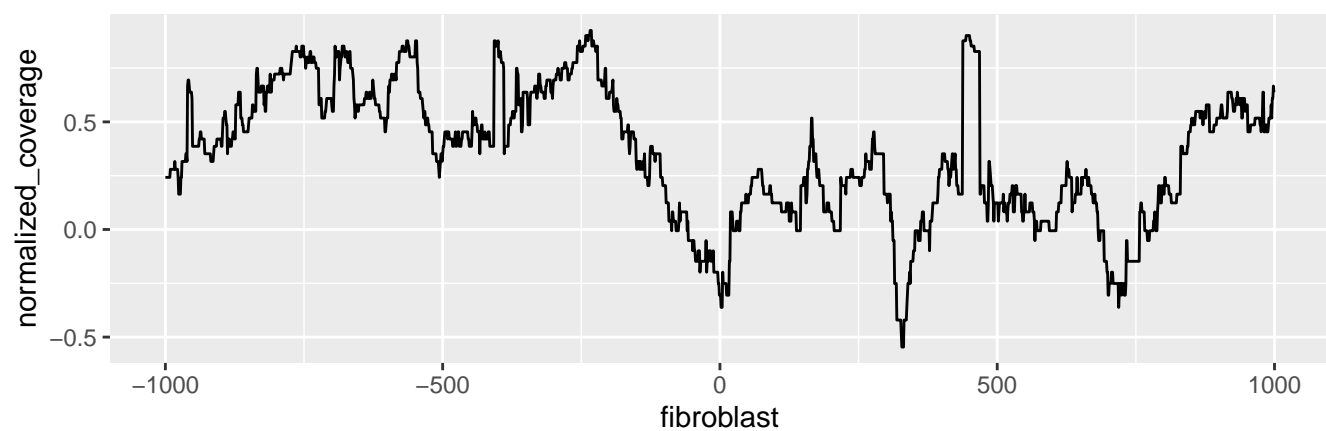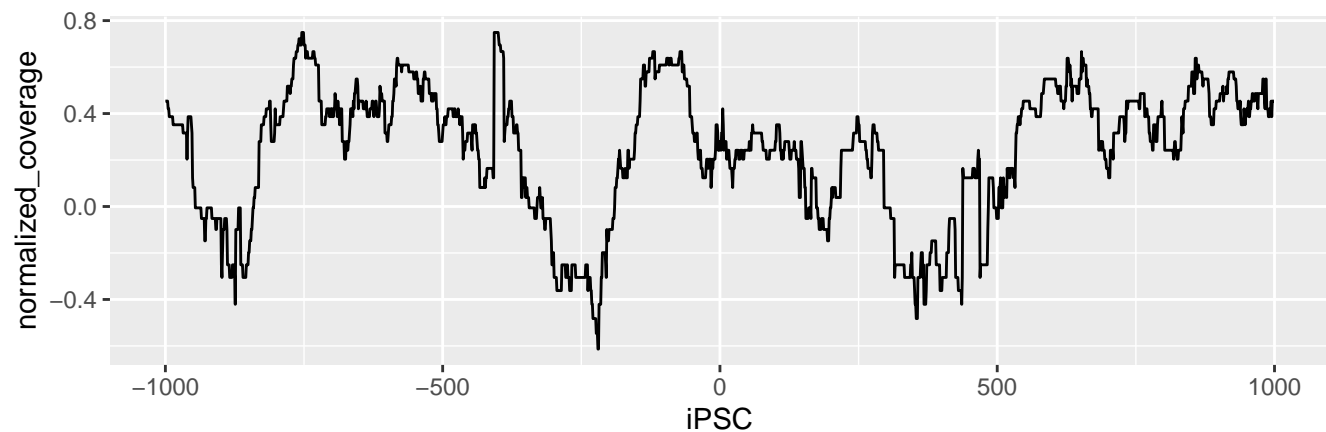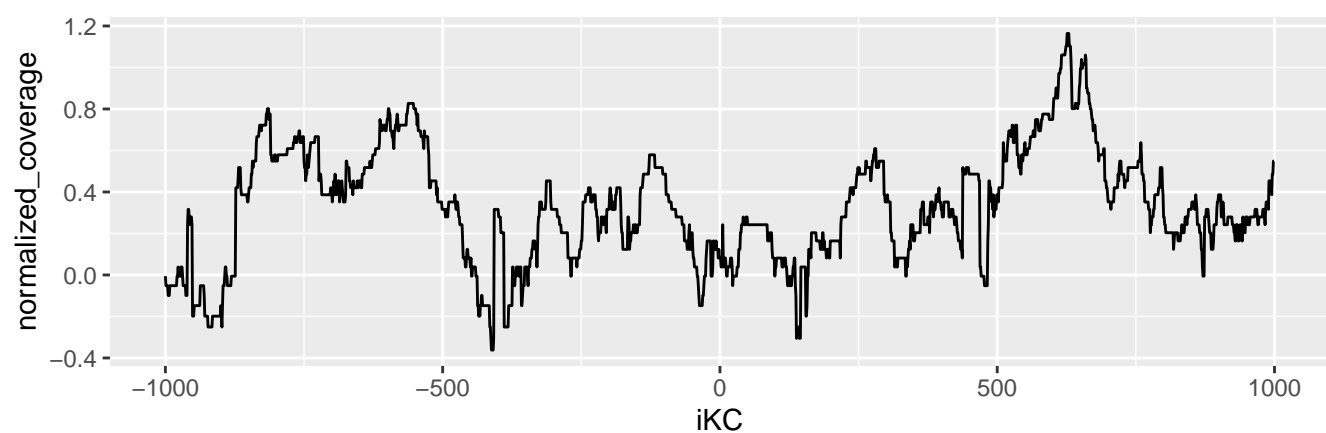

Supplement: Supplementary file 6 — Supplementary Data 3 [file 41467_2024_49400_MOESM6_ESM.zip › Supplementary Data 3/57_offtarget_sites/CO1-131_2KB/patient3_1.chr14_76670168_76670190.2KB.pdf]

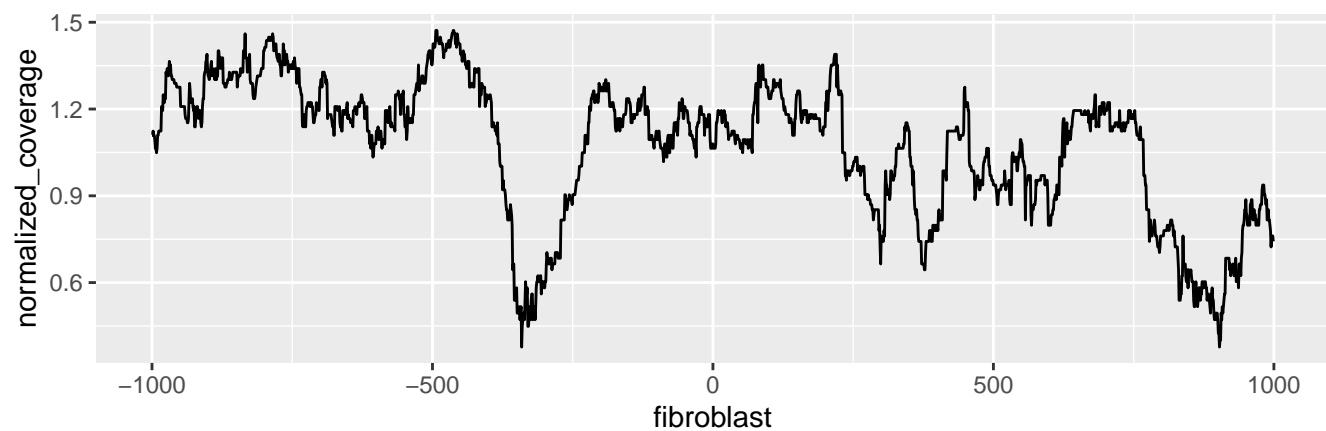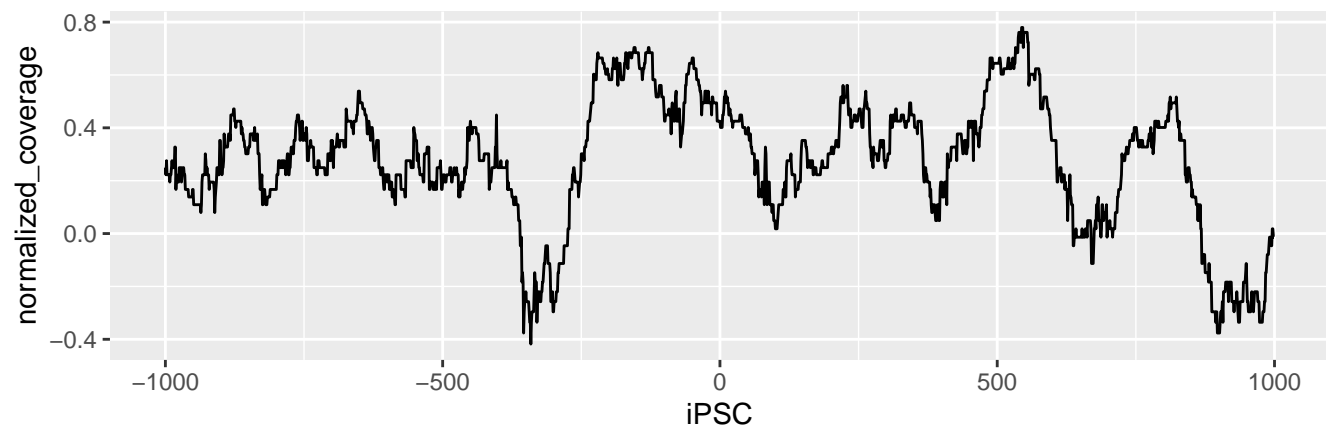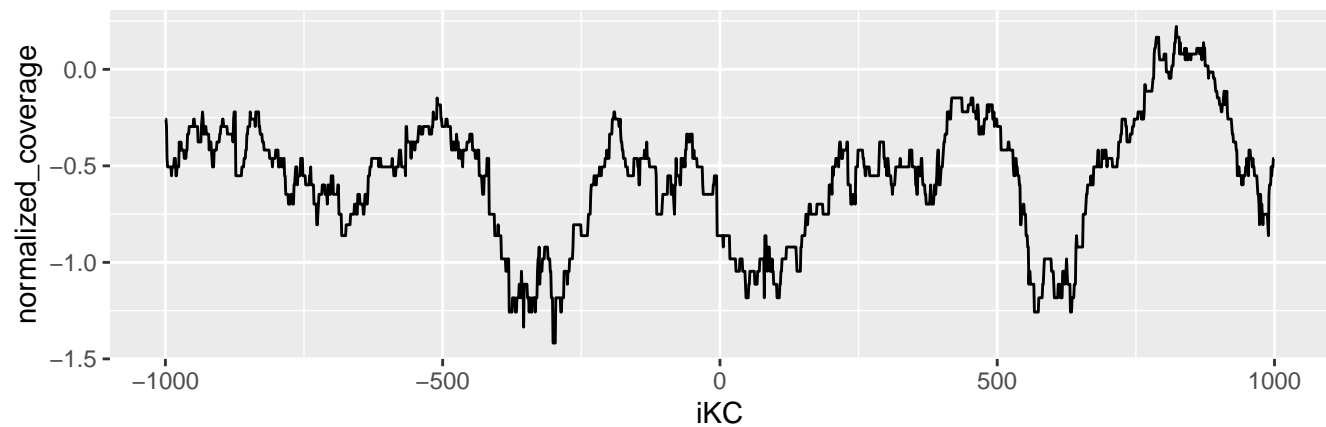

Supplement: Supplementary file 6 — Supplementary Data 3 [file 41467_2024_49400_MOESM6_ESM.zip › Supplementary Data 3/57_offtarget_sites/CO1-131_2KB/patient3_1.chr16_1306676_1306698.2KB.pdf]

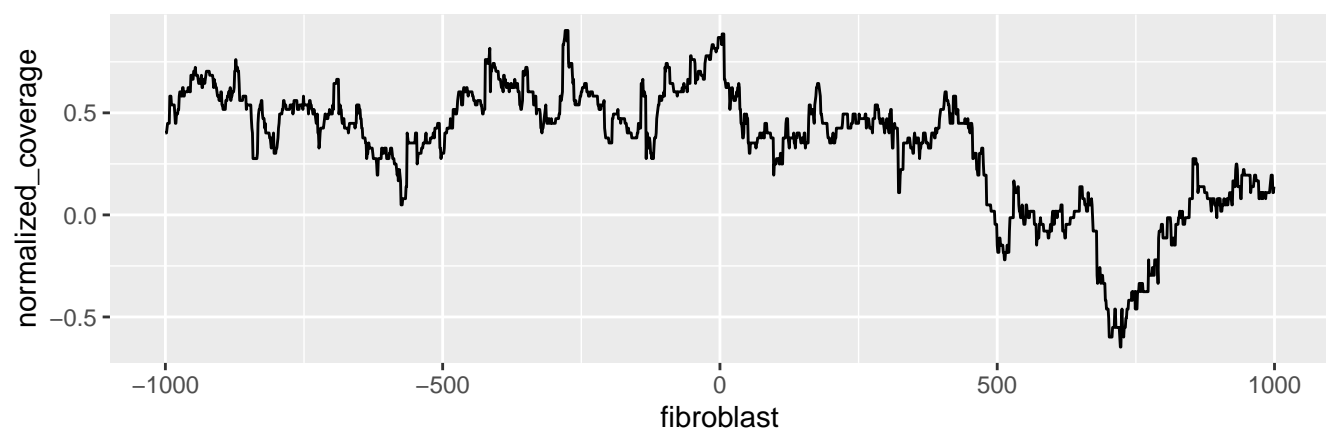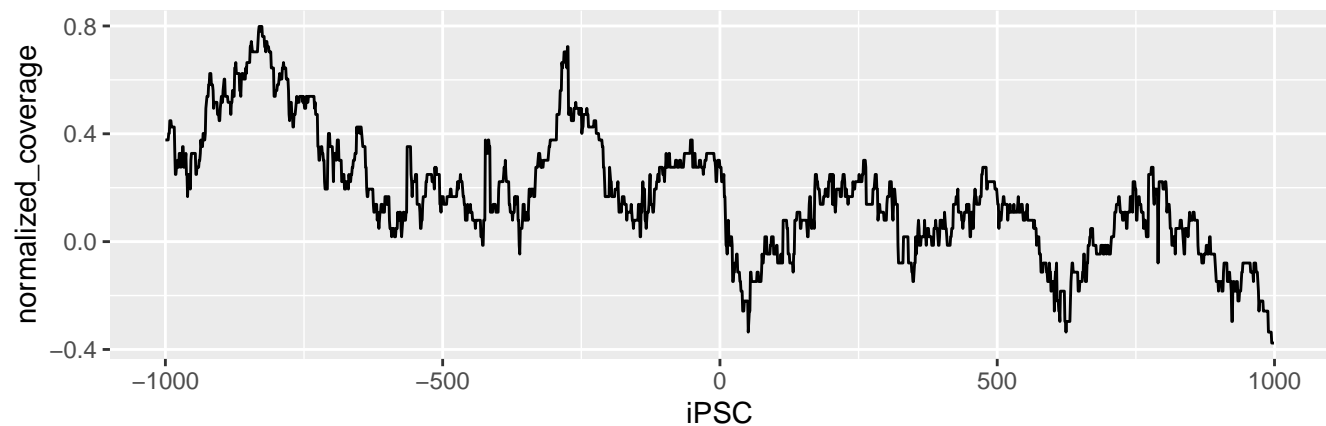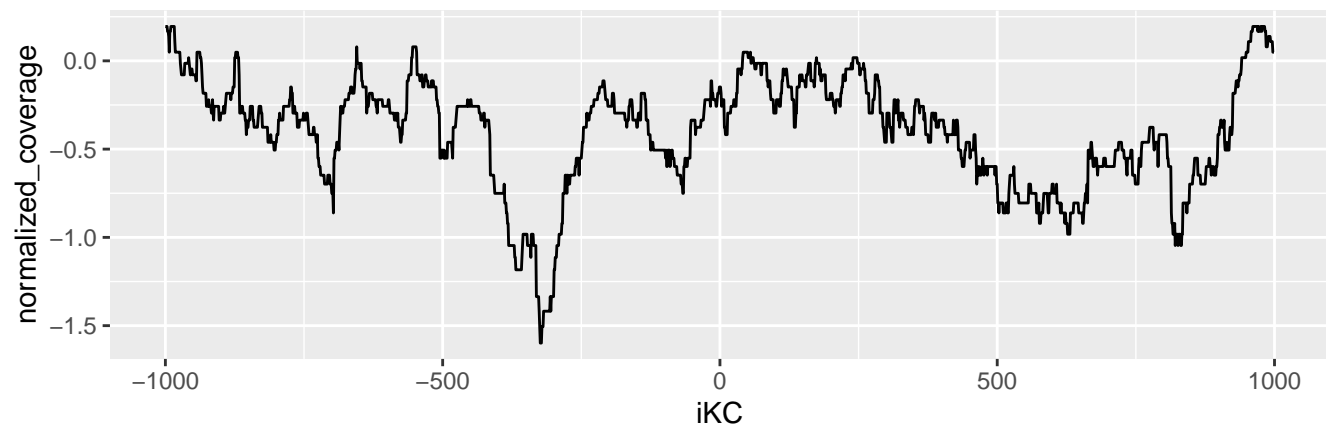

Supplement: Supplementary file 6 — Supplementary Data 3 [file 41467_2024_49400_MOESM6_ESM.zip › Supplementary Data 3/57_offtarget_sites/CO1-131_2KB/patient3_1.chr16_22142360_22142382.2KB.pdf]

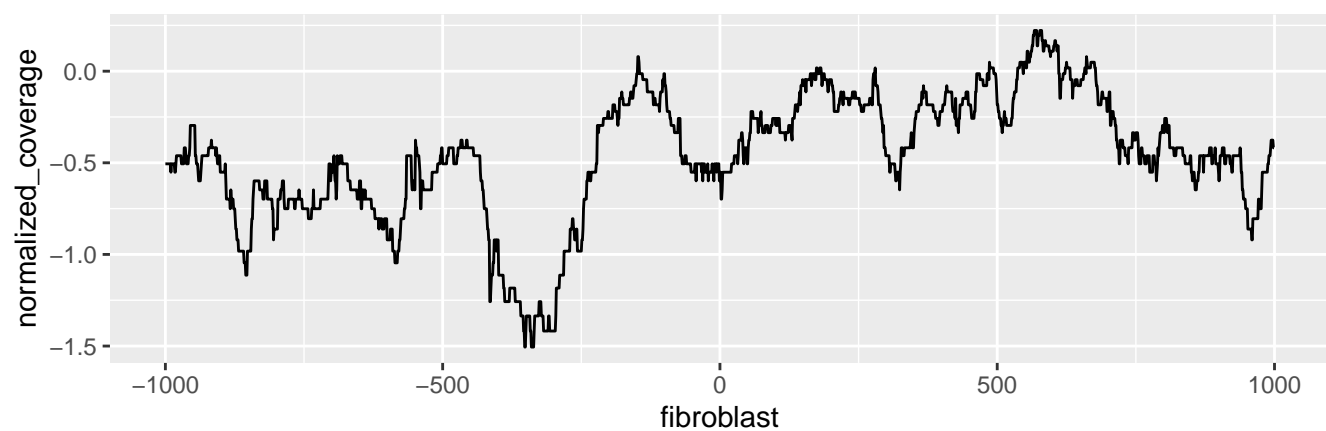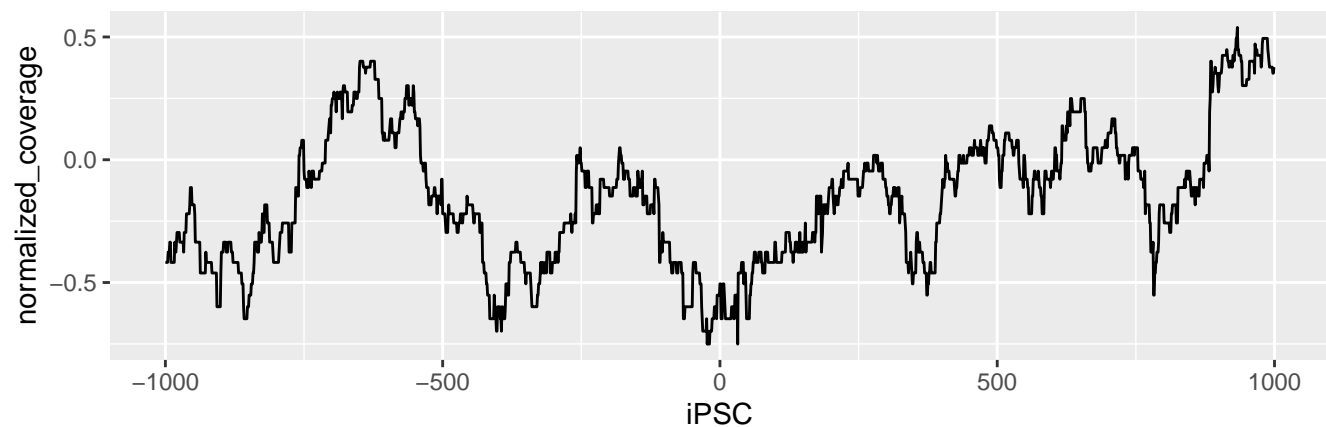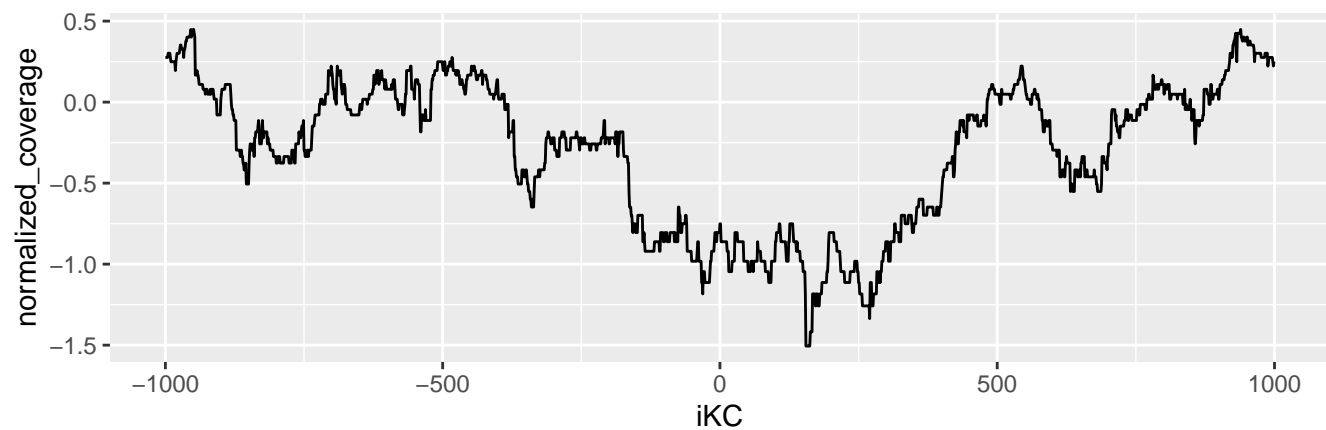

Supplement: Supplementary file 6 — Supplementary Data 3 [file 41467_2024_49400_MOESM6_ESM.zip › Supplementary Data 3/57_offtarget_sites/CO1-131_2KB/patient3_1.chr16_26135954_26135976.2KB.pdf]

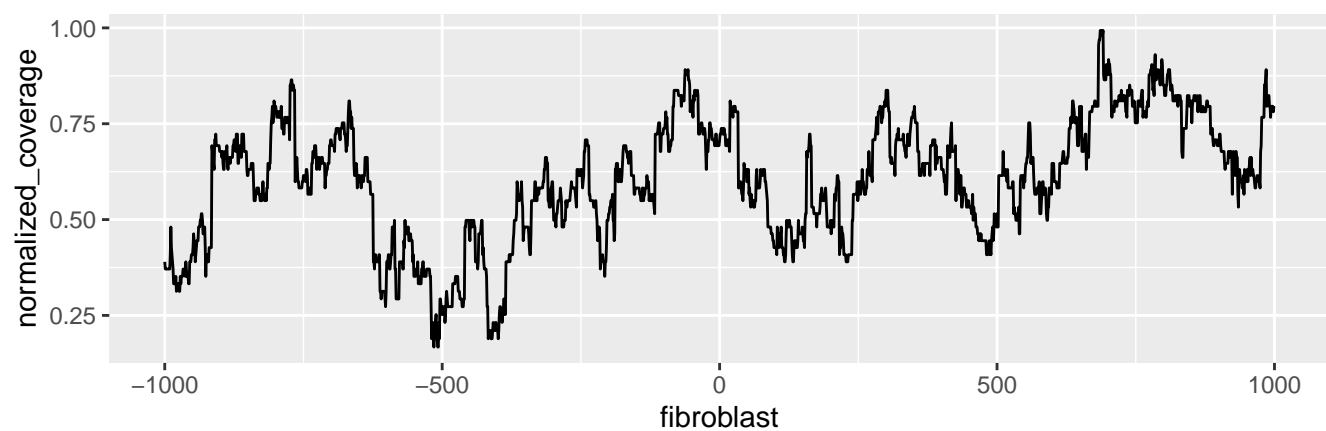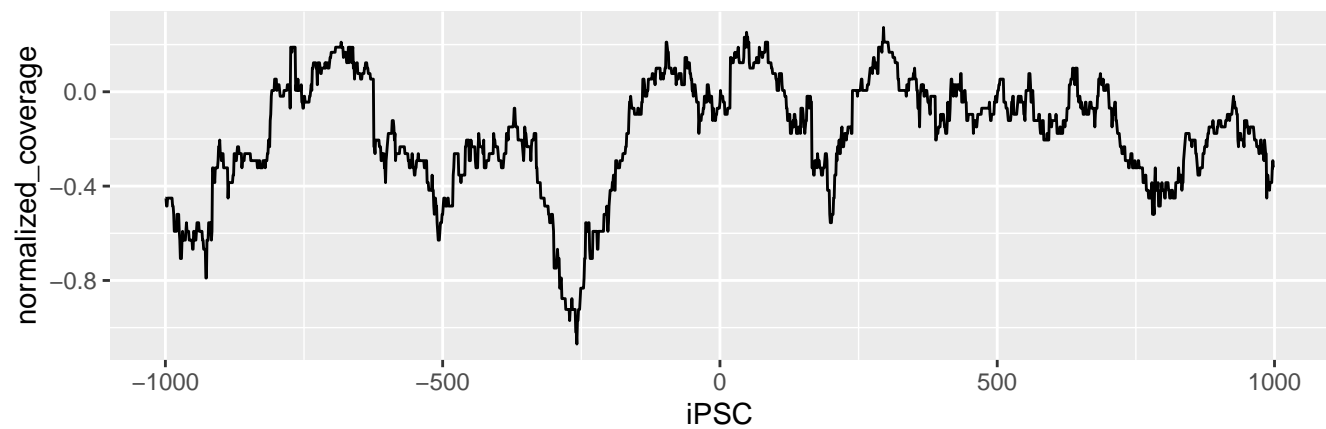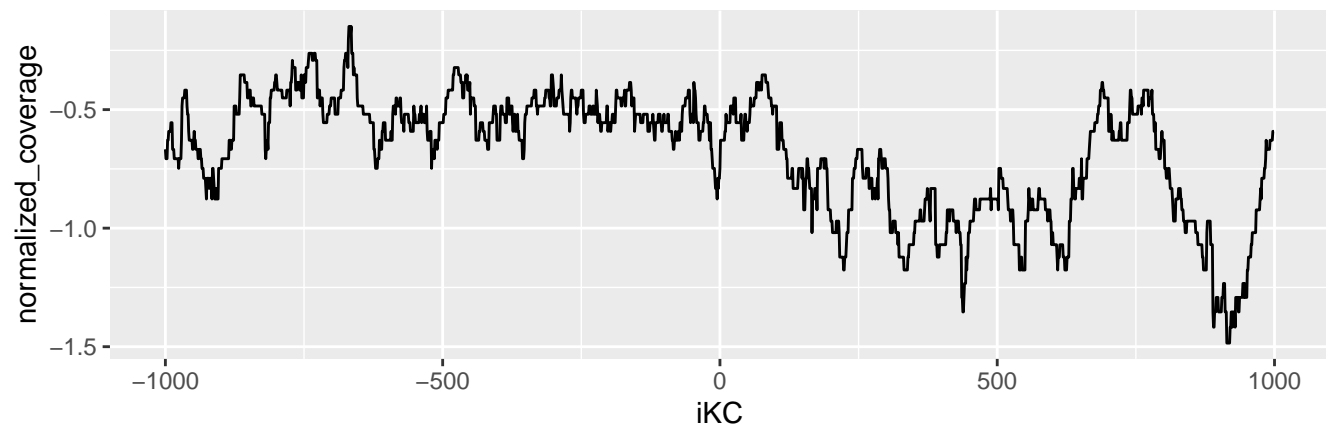

Supplement: Supplementary file 6 — Supplementary Data 3 [file 41467_2024_49400_MOESM6_ESM.zip › Supplementary Data 3/57_offtarget_sites/CO1-131_2KB/patient3_1.chr17_49845081_49845103.2KB.pdf]

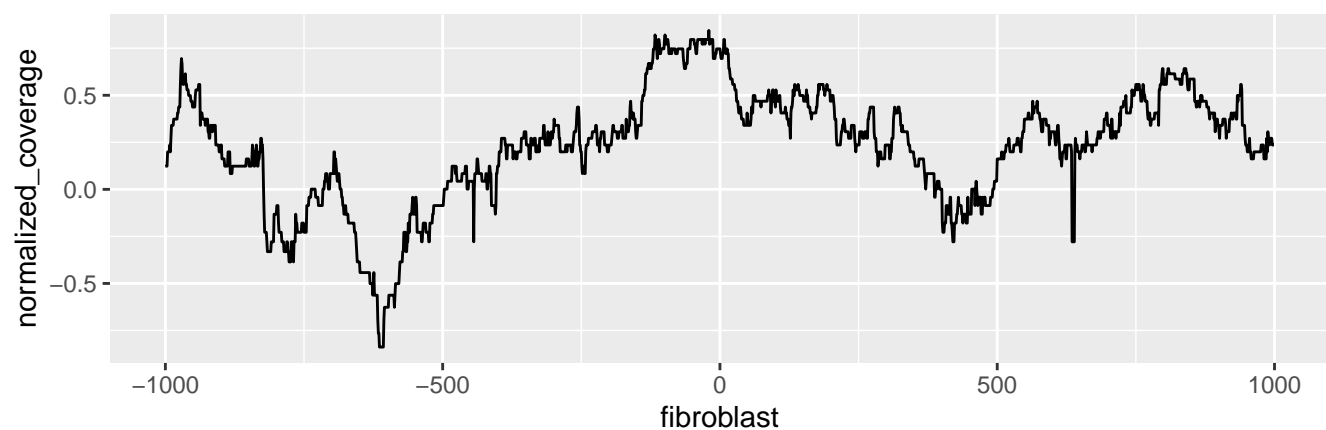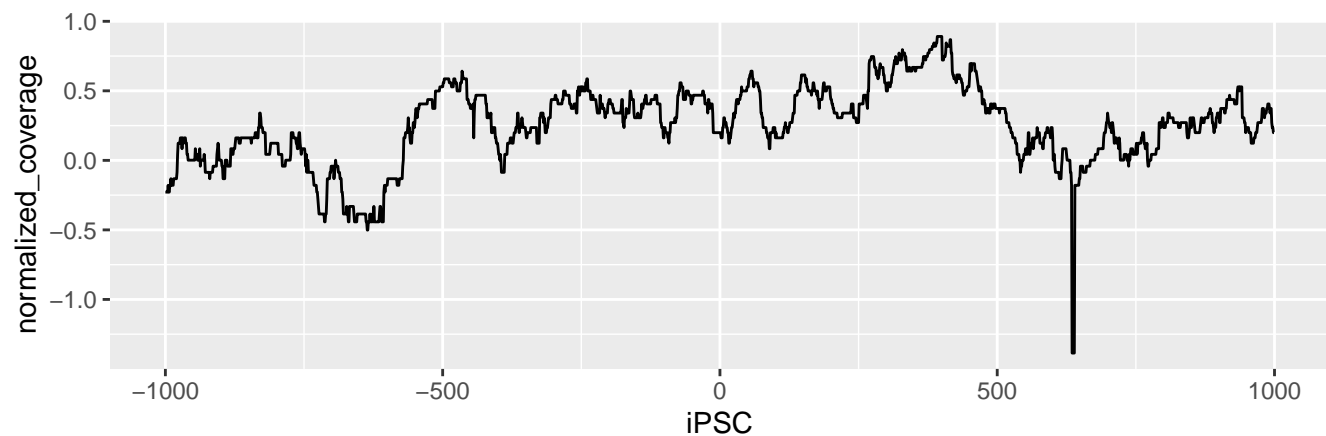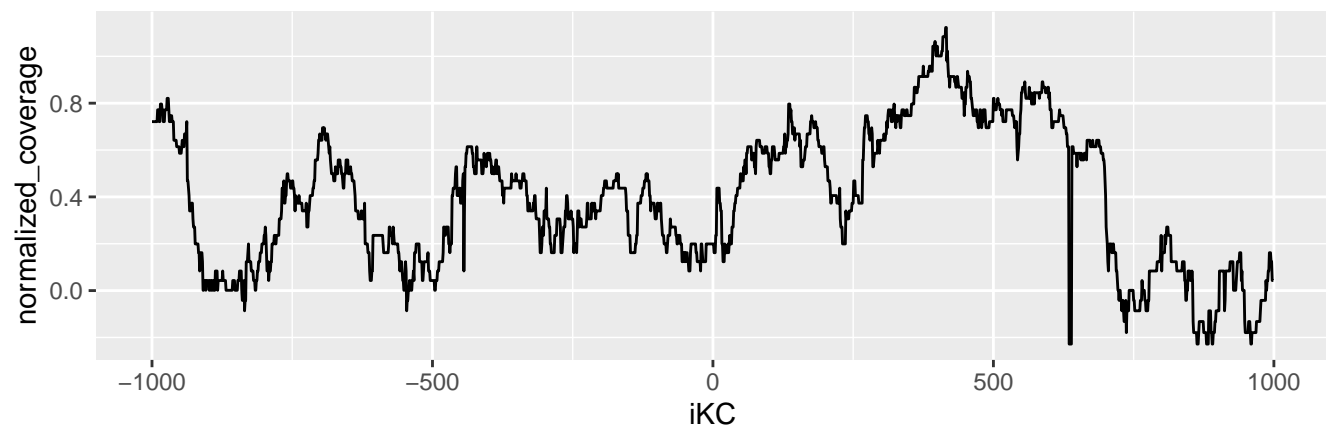

Supplement: Supplementary file 6 — Supplementary Data 3 [file 41467_2024_49400_MOESM6_ESM.zip › Supplementary Data 3/57_offtarget_sites/CO1-131_2KB/patient3_1.chr18_35288320_35288342.2KB.pdf]

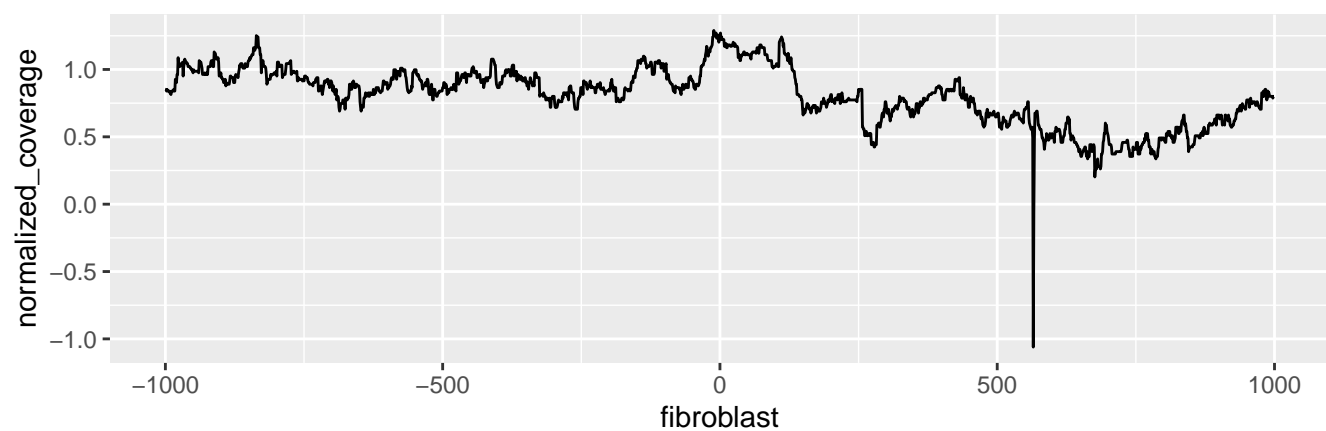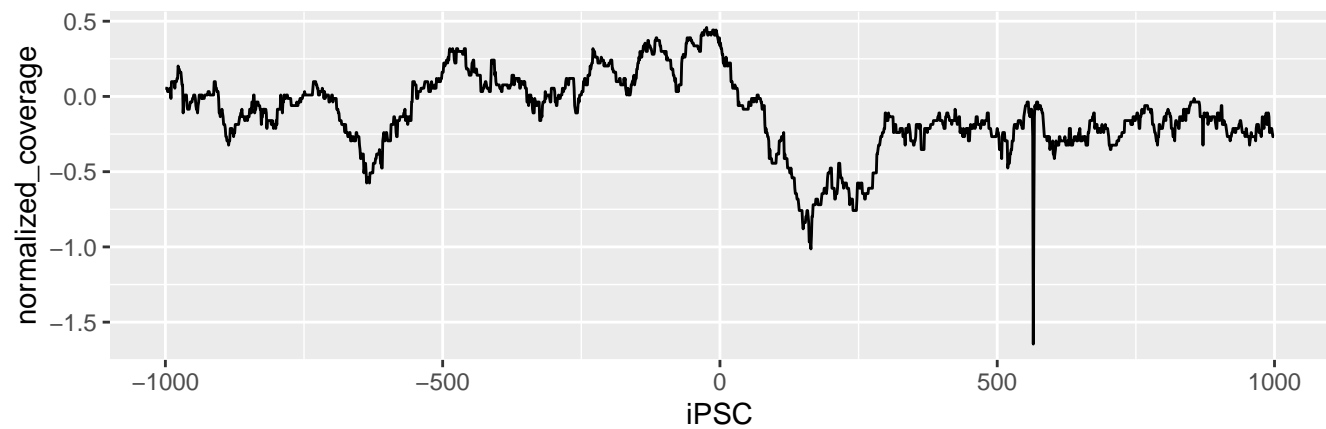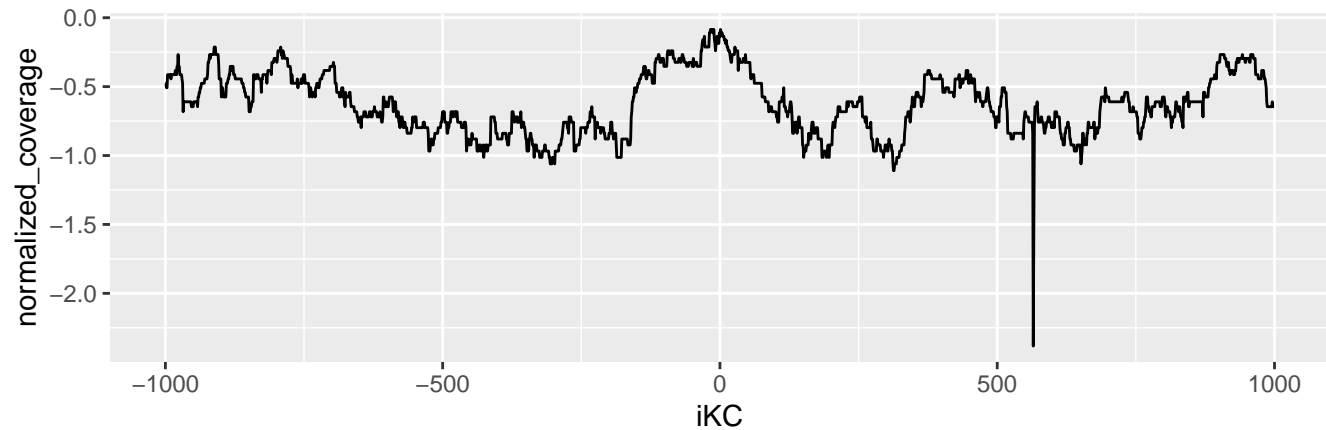

Supplement: Supplementary file 6 — Supplementary Data 3 [file 41467_2024_49400_MOESM6_ESM.zip › Supplementary Data 3/57_offtarget_sites/CO1-131_2KB/patient3_1.chr19_1730409_1730431.2KB.pdf]

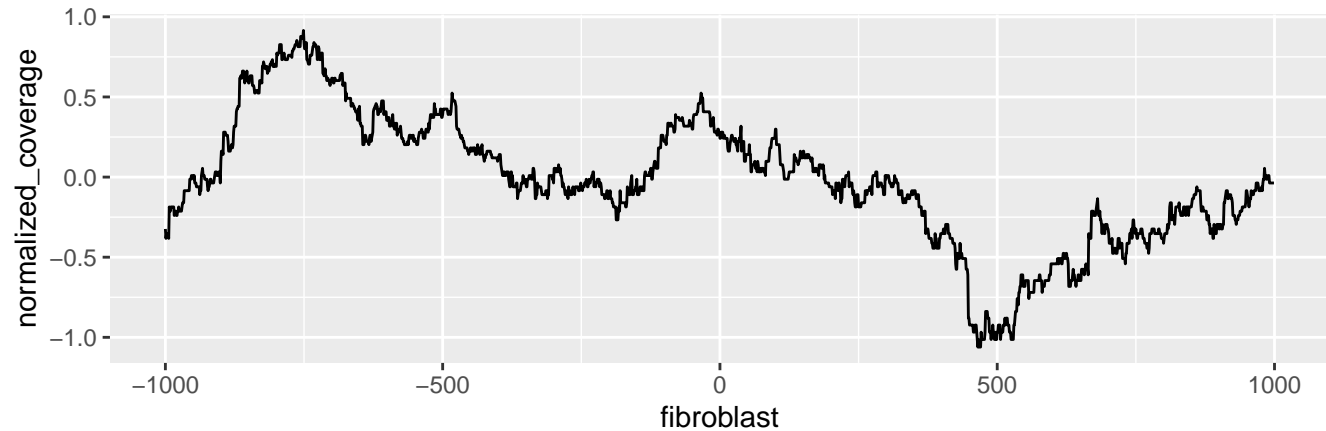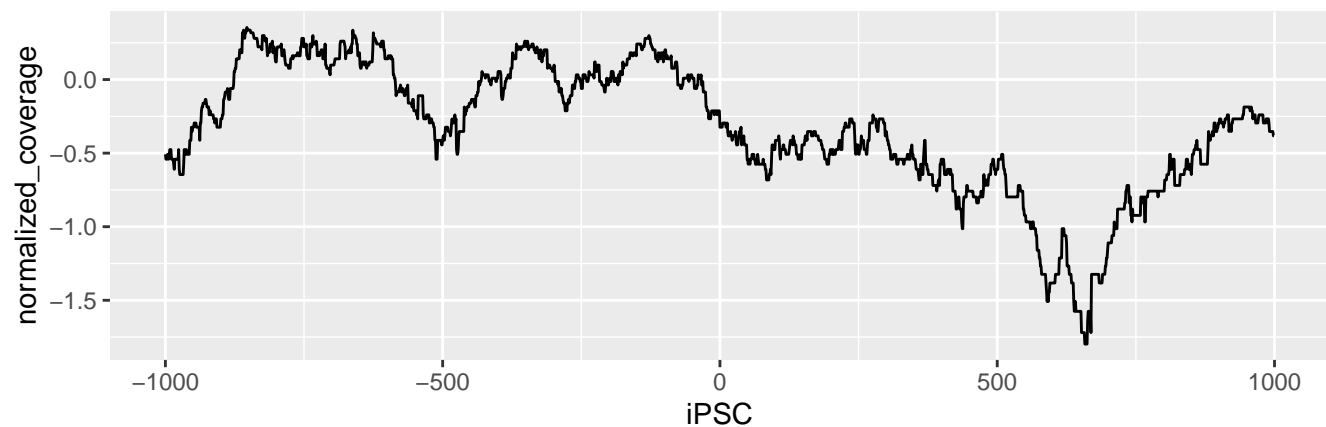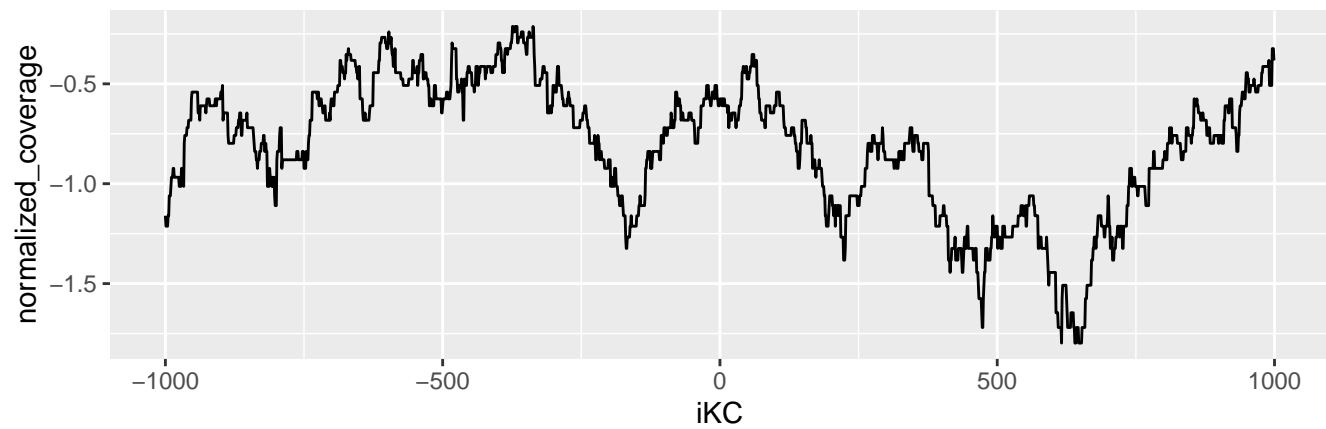

Supplement: Supplementary file 6 — Supplementary Data 3 [file 41467_2024_49400_MOESM6_ESM.zip › Supplementary Data 3/57_offtarget_sites/CO1-131_2KB/patient3_1.chr19_35142505_35142527.2KB.pdf]

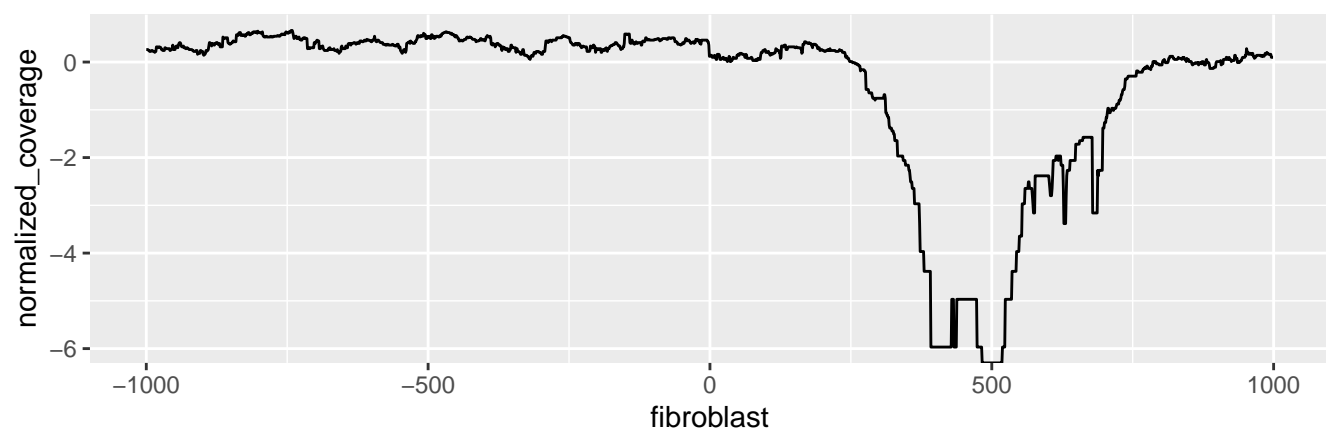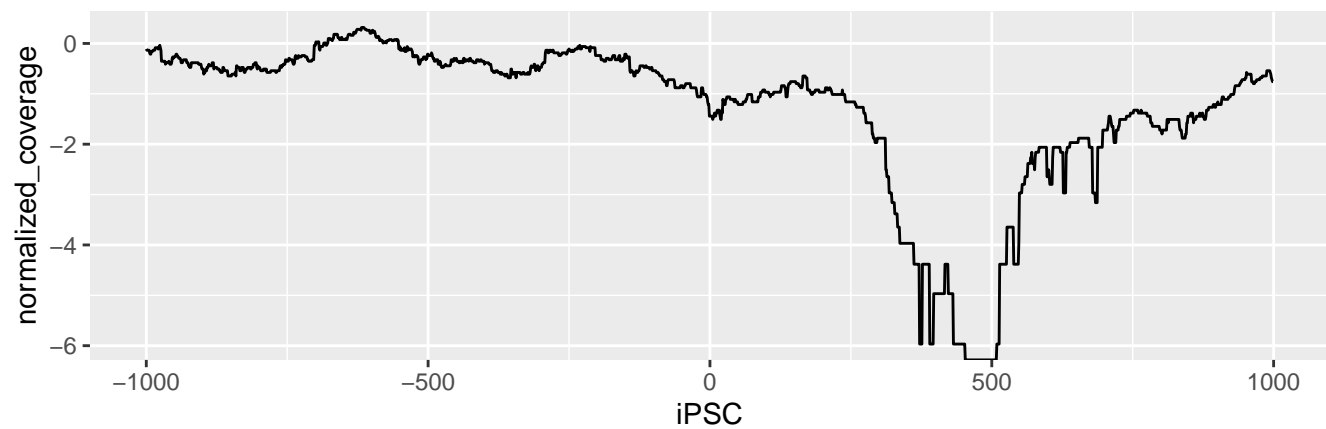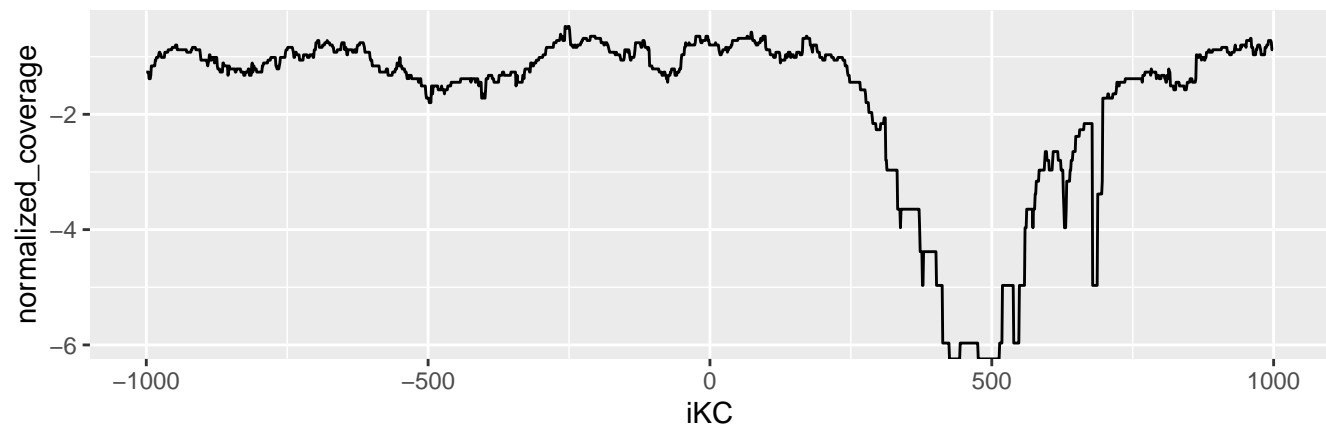

Supplement: Supplementary file 6 — Supplementary Data 3 [file 41467_2024_49400_MOESM6_ESM.zip › Supplementary Data 3/57_offtarget_sites/CO1-131_2KB/patient3_1.chr19_38735330_38735352.2KB.pdf]

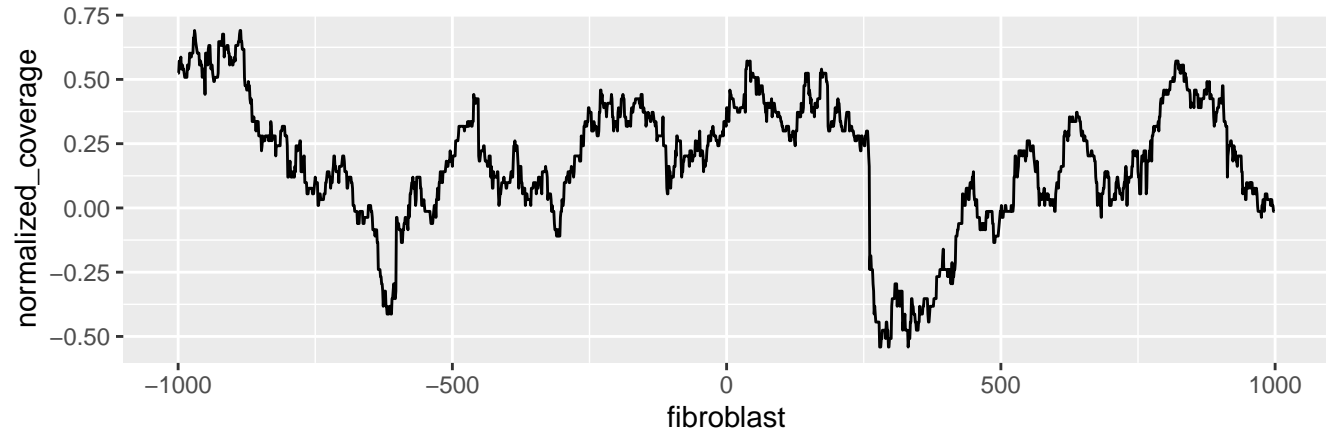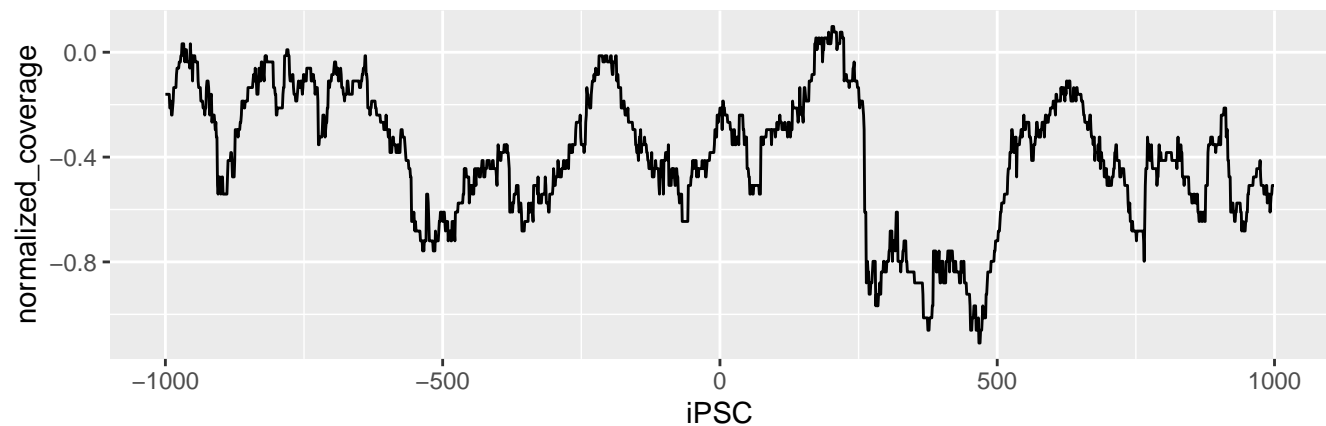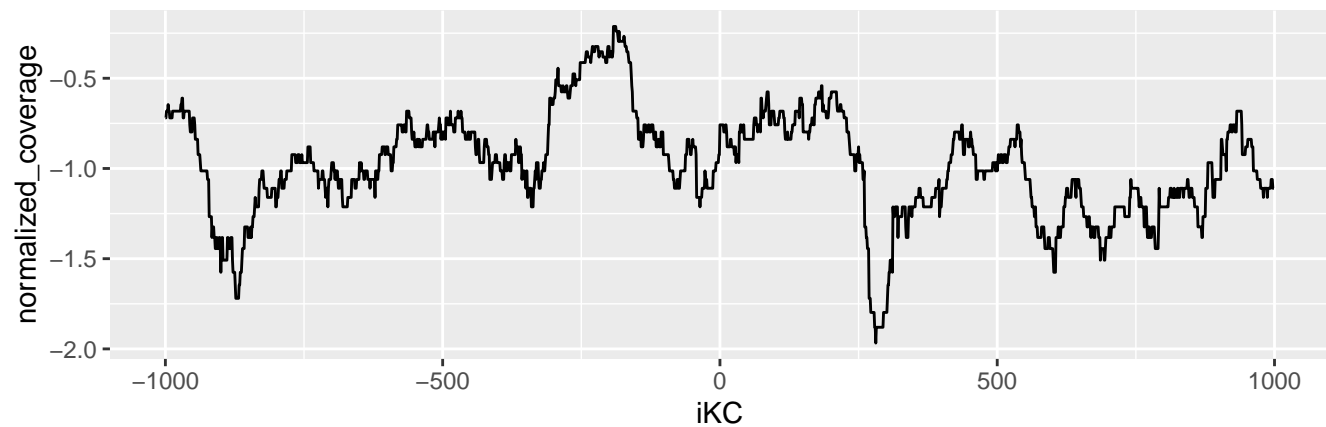

Supplement: Supplementary file 6 — Supplementary Data 3 [file 41467_2024_49400_MOESM6_ESM.zip › Supplementary Data 3/57_offtarget_sites/CO1-131_2KB/patient3_1.chr19_39173786_39173808.2KB.pdf]

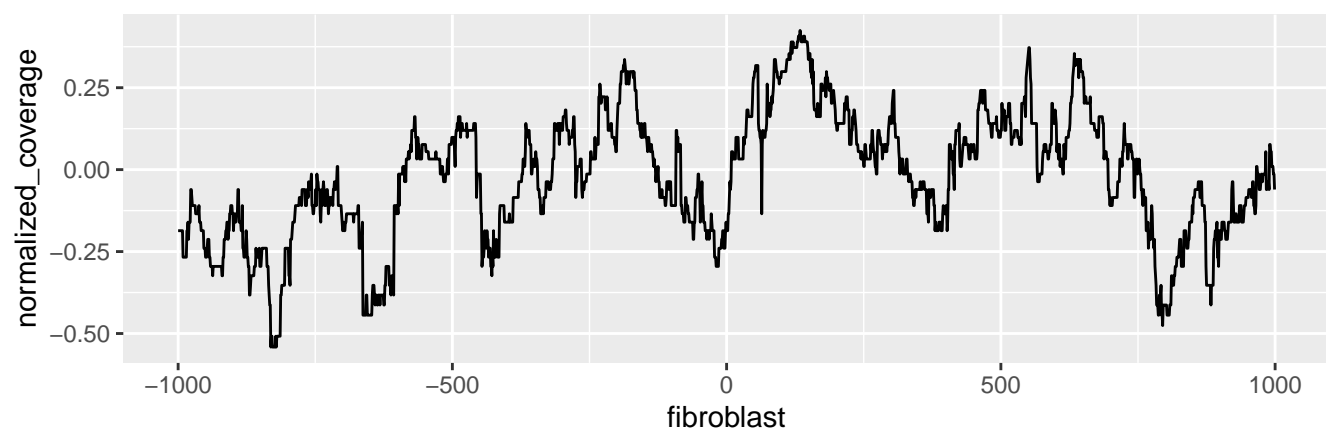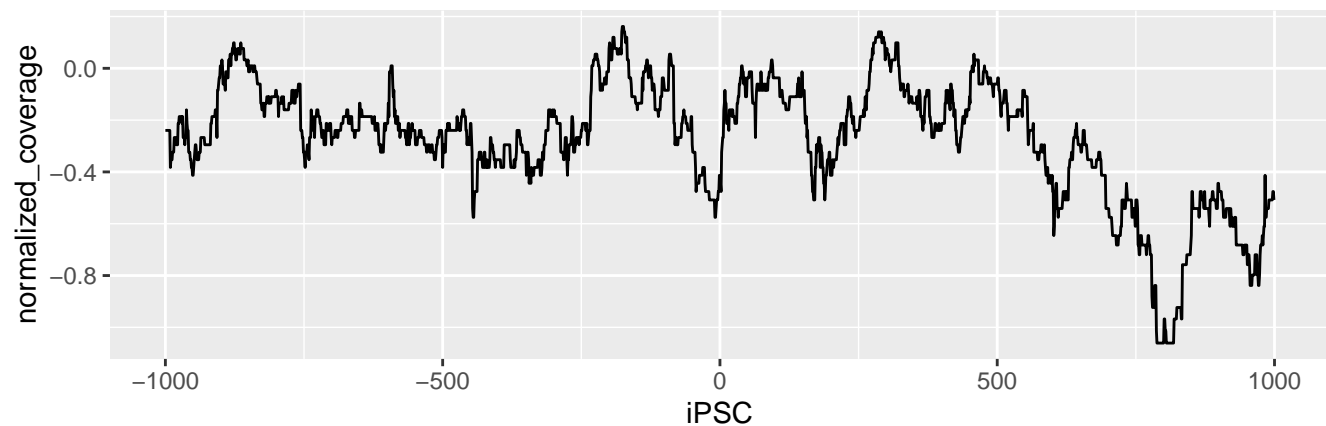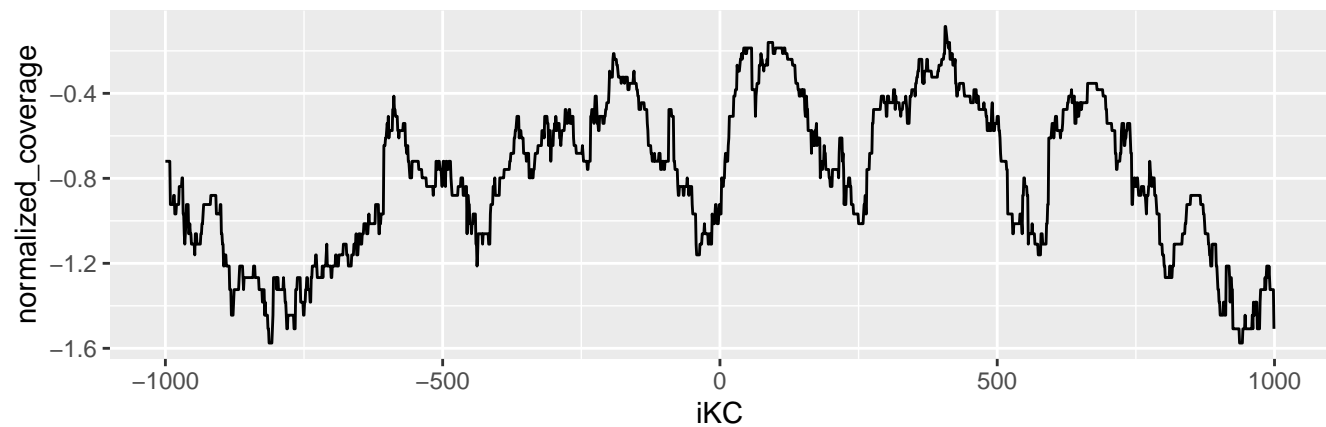

Supplement: Supplementary file 6 — Supplementary Data 3 [file 41467_2024_49400_MOESM6_ESM.zip › Supplementary Data 3/57_offtarget_sites/CO1-131_2KB/patient3_1.chr19_52431793_52431815.2KB.pdf]

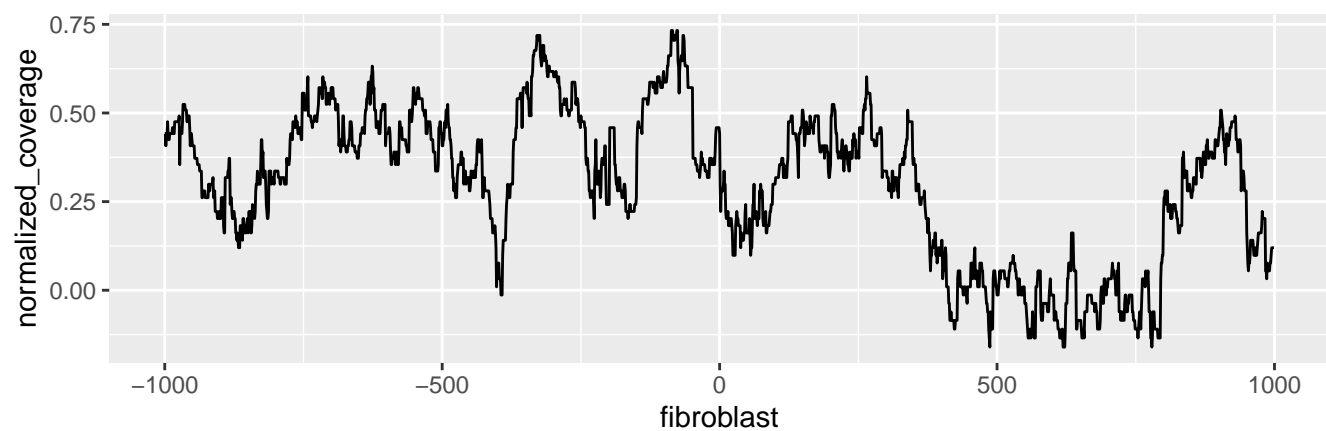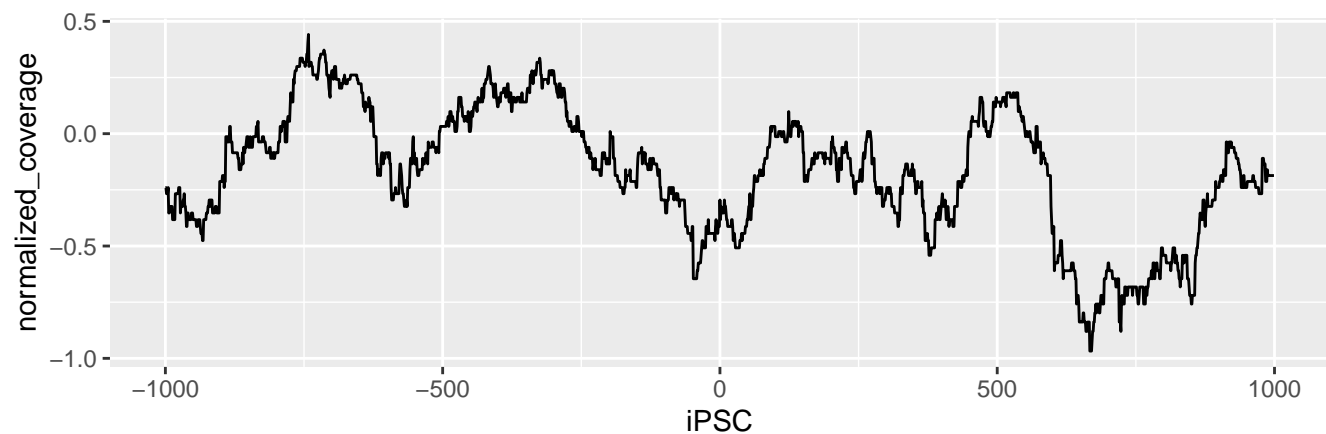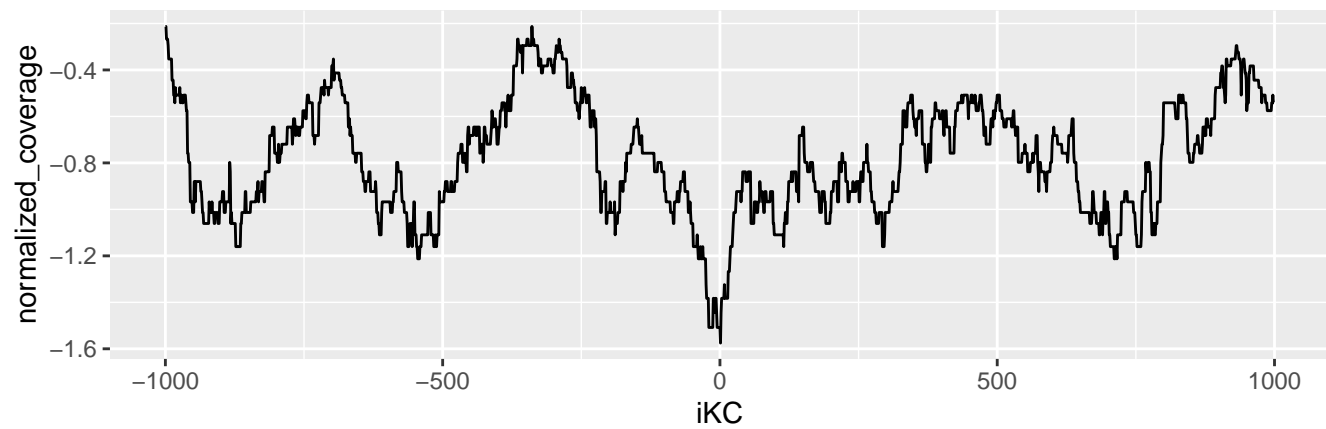

Supplement: Supplementary file 6 — Supplementary Data 3 [file 41467_2024_49400_MOESM6_ESM.zip › Supplementary Data 3/57_offtarget_sites/CO1-131_2KB/patient3_1.chr19_7933017_7933039.2KB.pdf]

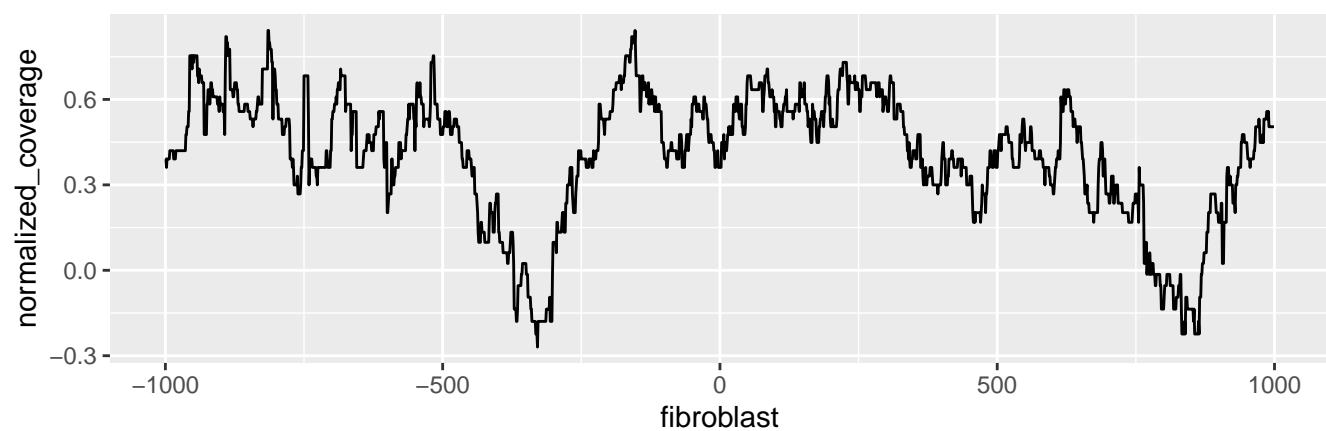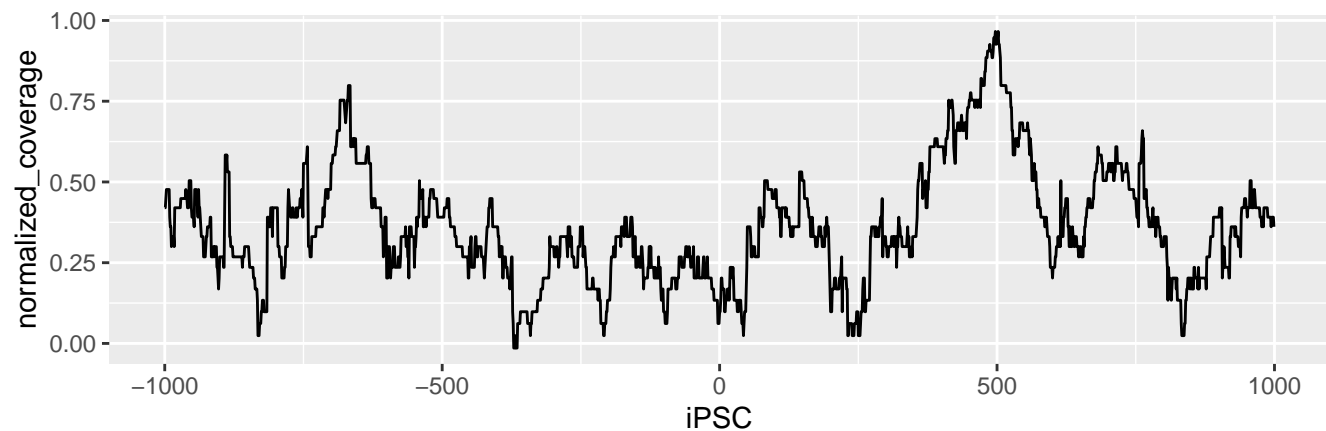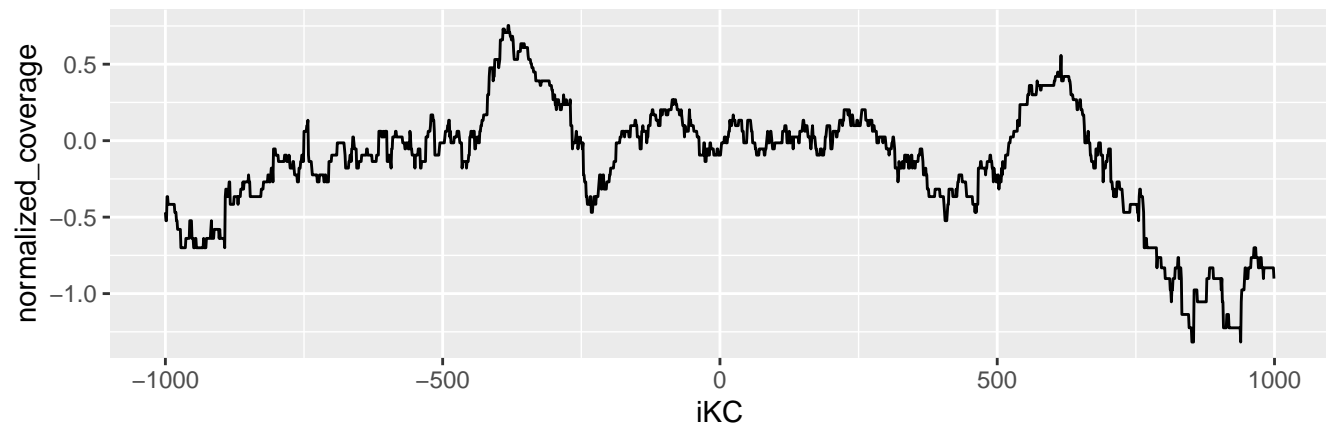

Supplement: Supplementary file 6 — Supplementary Data 3 [file 41467_2024_49400_MOESM6_ESM.zip › Supplementary Data 3/57_offtarget_sites/CO1-131_2KB/patient3_1.chr2_120339100_120339122.2KB.pdf]

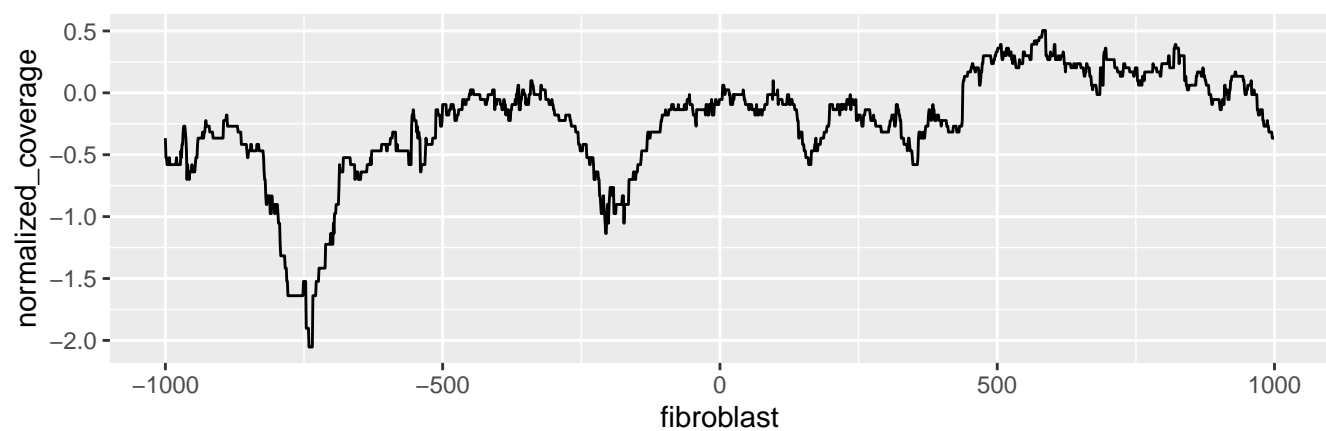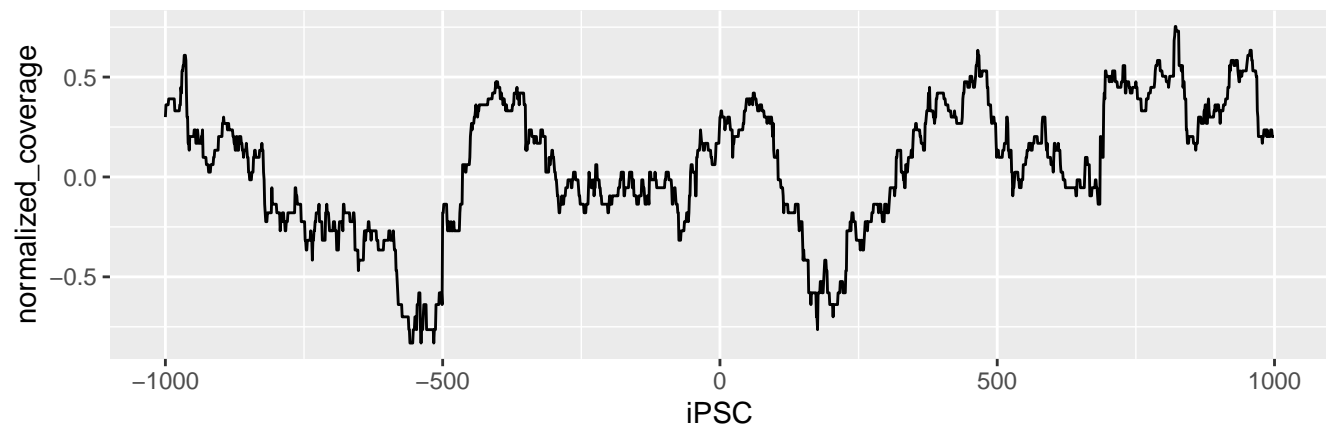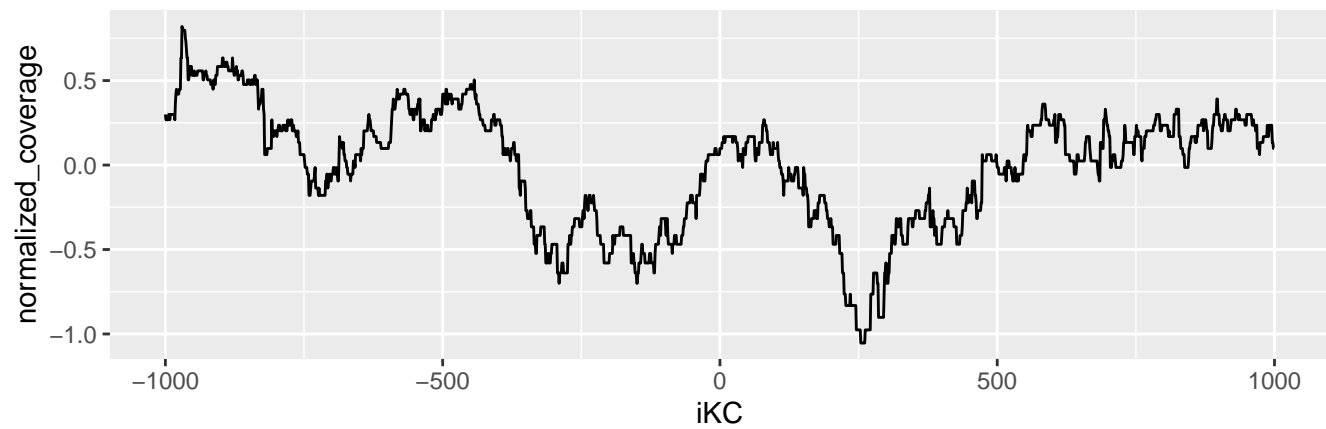

Supplement: Supplementary file 6 — Supplementary Data 3 [file 41467_2024_49400_MOESM6_ESM.zip › Supplementary Data 3/57_offtarget_sites/CO1-131_2KB/patient3_1.chr2_13678023_13678045.2KB.pdf]

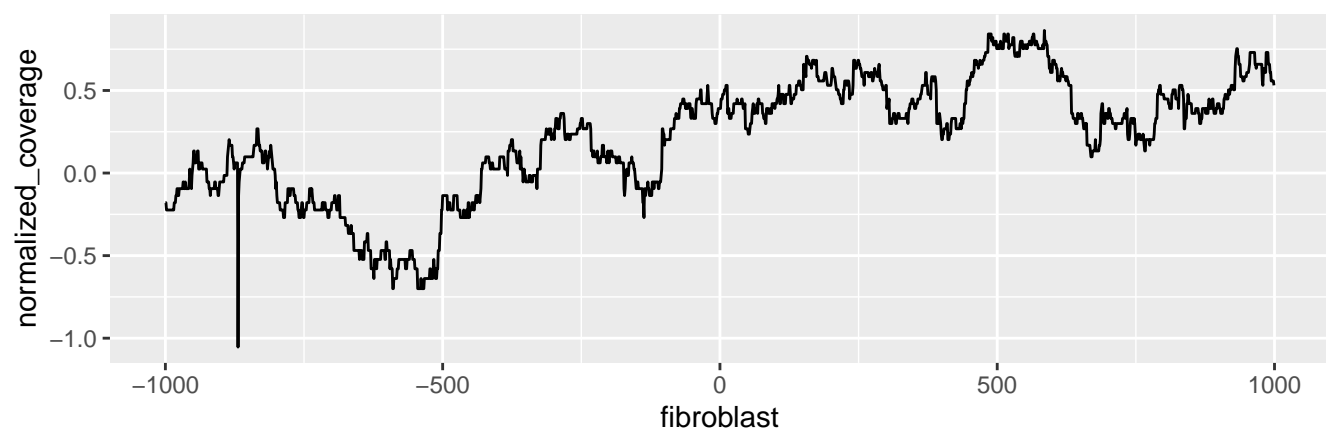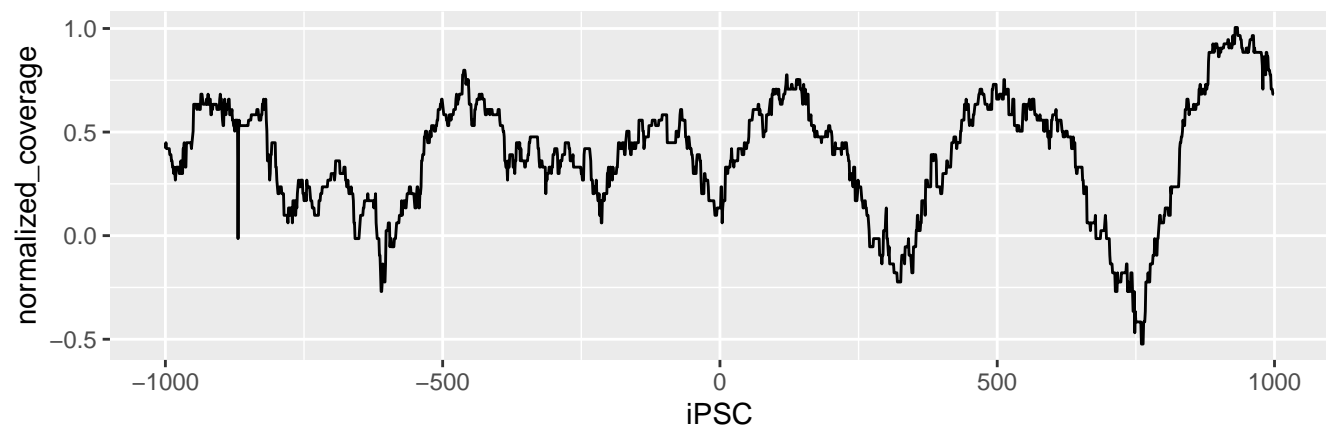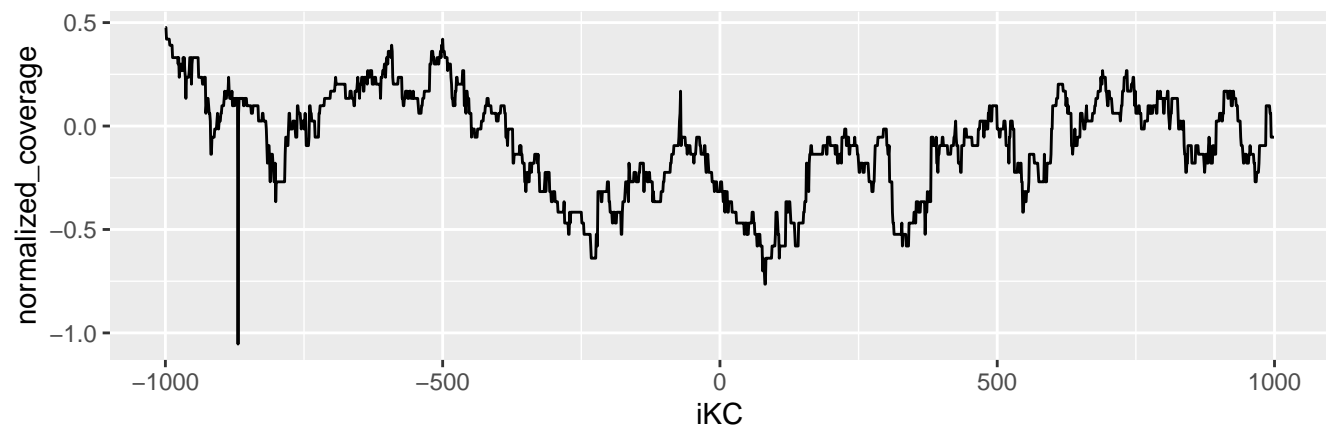

Supplement: Supplementary file 6 — Supplementary Data 3 [file 41467_2024_49400_MOESM6_ESM.zip › Supplementary Data 3/57_offtarget_sites/CO1-131_2KB/patient3_1.chr2_1625392_1625414.2KB.pdf]

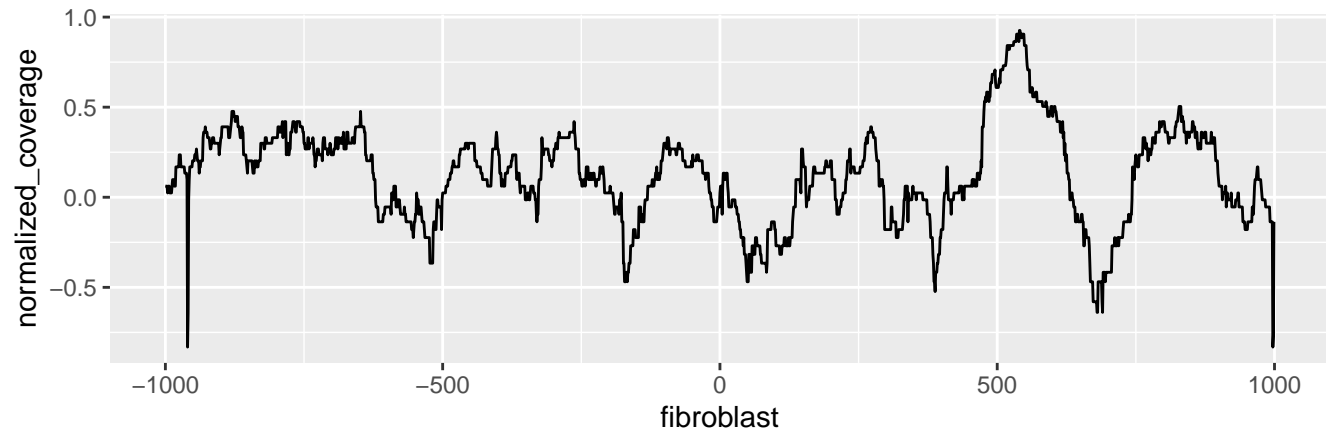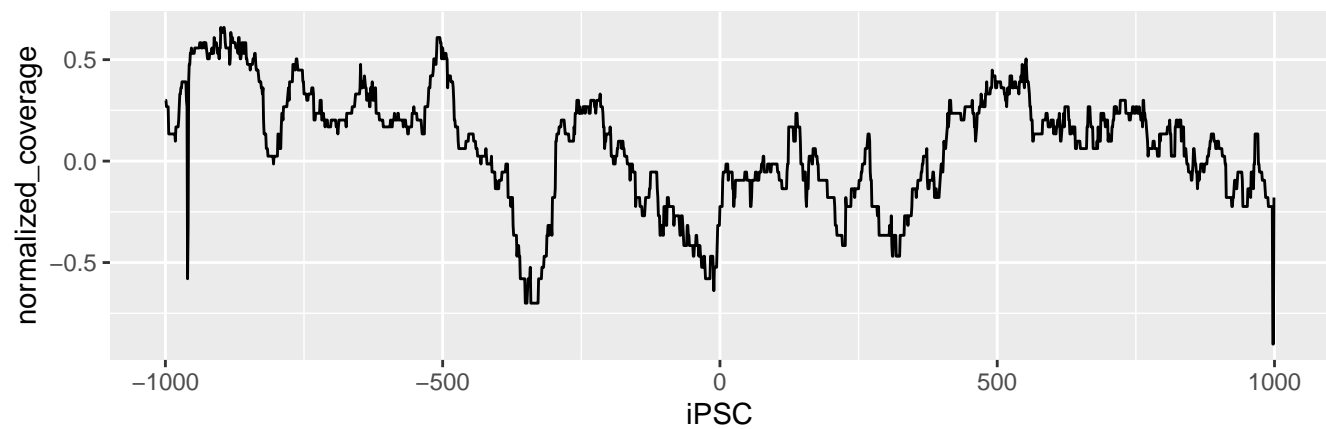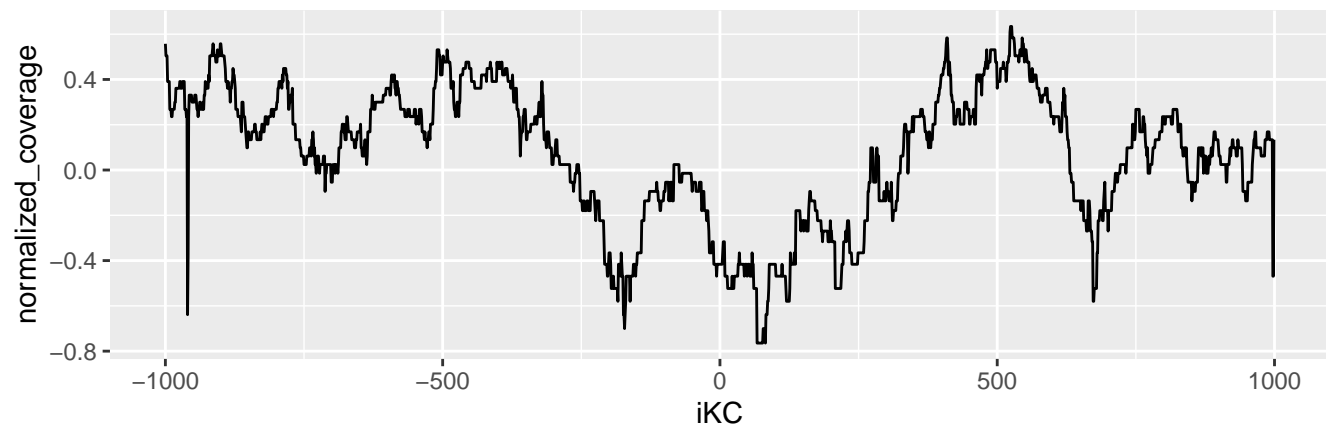

Supplement: Supplementary file 6 — Supplementary Data 3 [file 41467_2024_49400_MOESM6_ESM.zip › Supplementary Data 3/57_offtarget_sites/CO1-131_2KB/patient3_1.chr2_62262033_62262055.2KB.pdf]

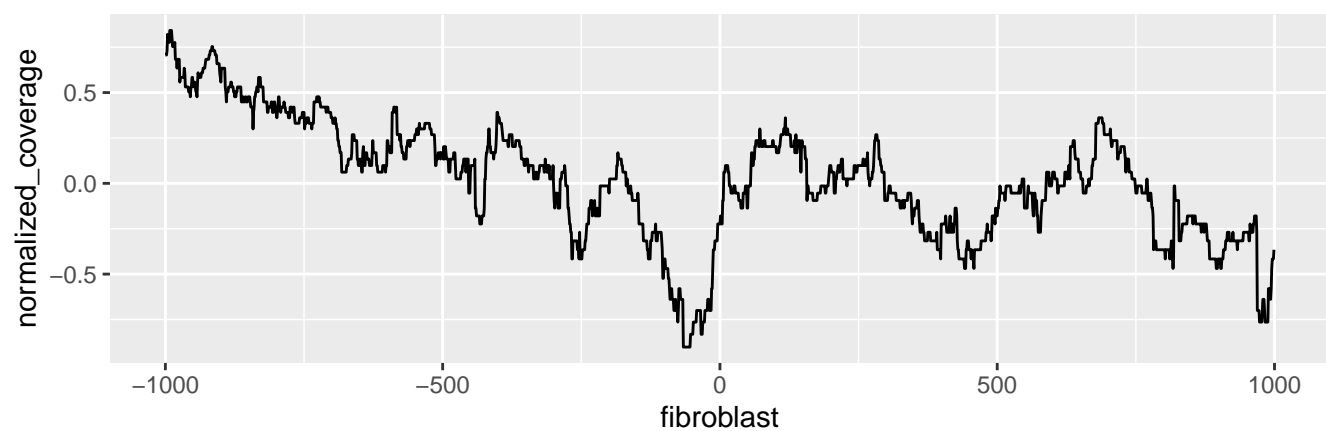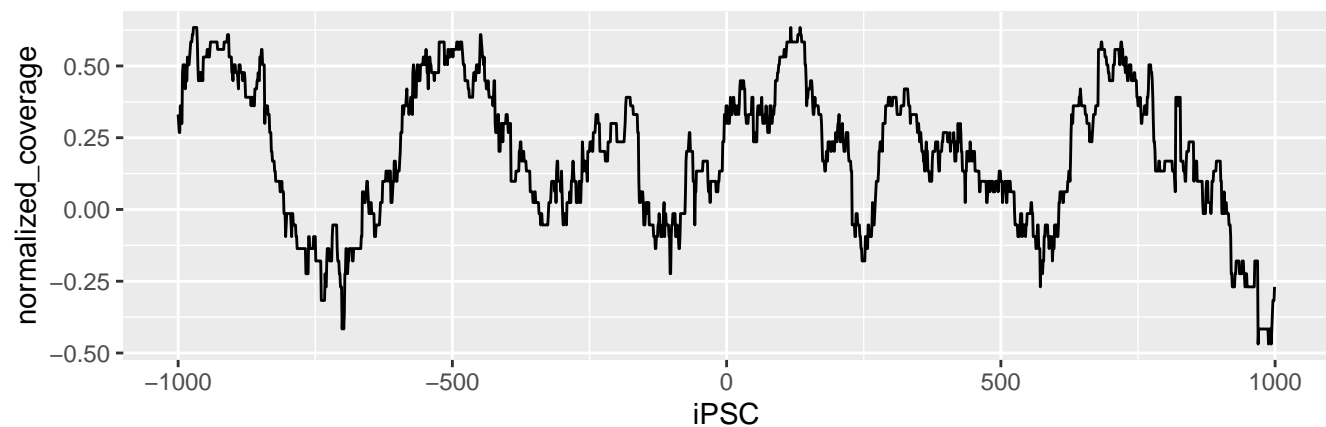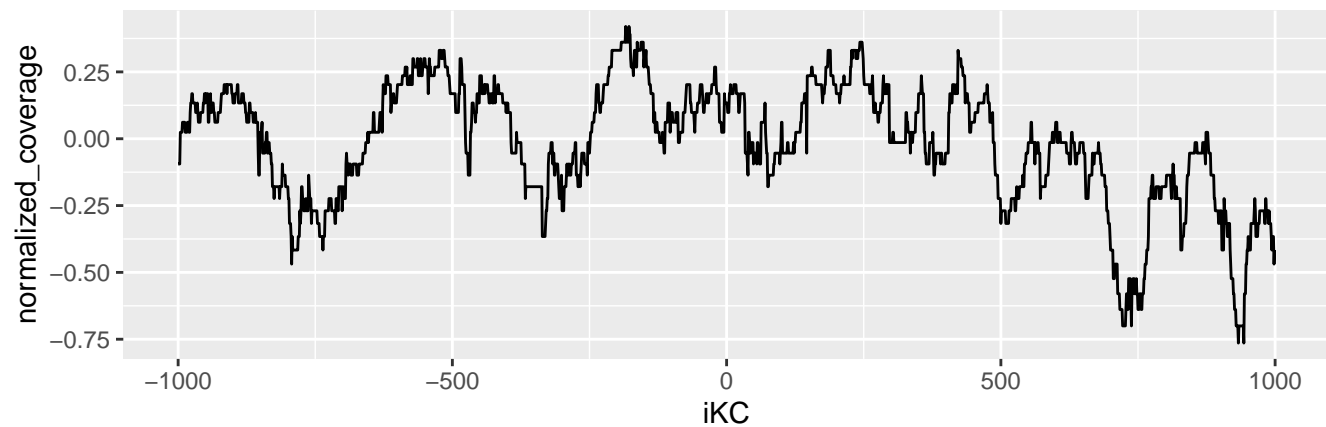

Supplement: Supplementary file 6 — Supplementary Data 3 [file 41467_2024_49400_MOESM6_ESM.zip › Supplementary Data 3/57_offtarget_sites/CO1-131_2KB/patient3_1.chr2_88349353_88349375.2KB.pdf]

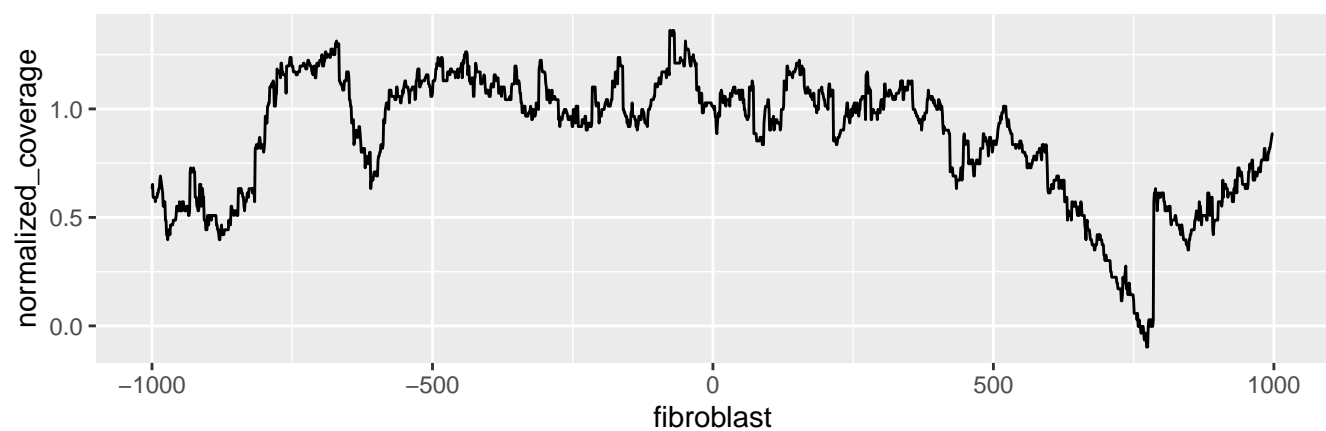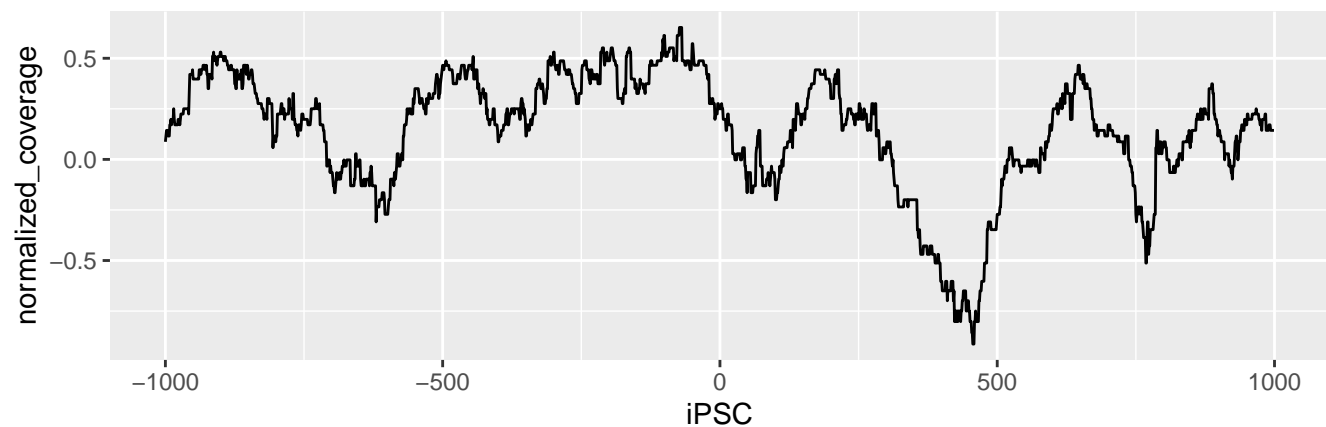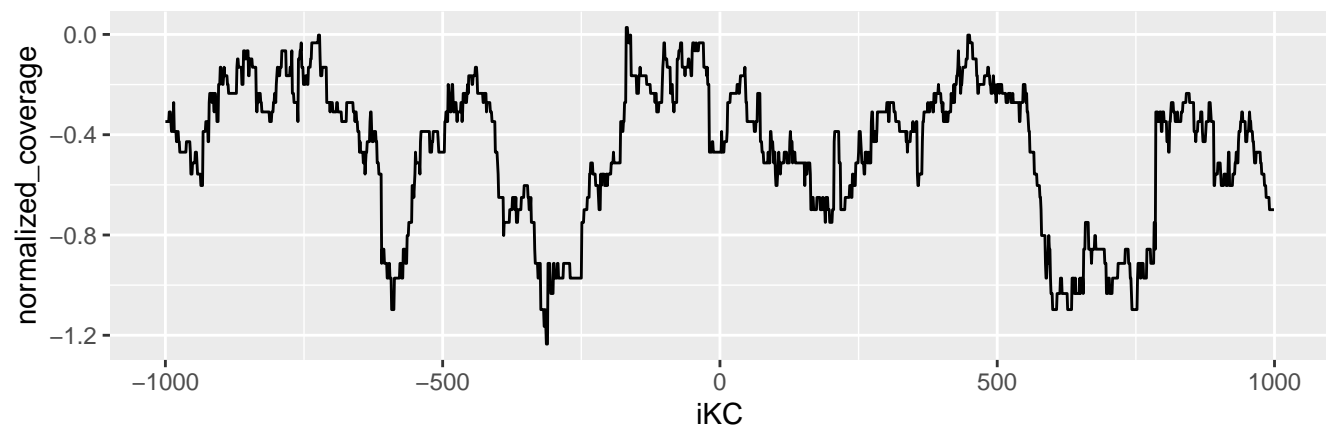

Supplement: Supplementary file 6 — Supplementary Data 3 [file 41467_2024_49400_MOESM6_ESM.zip › Supplementary Data 3/57_offtarget_sites/CO1-131_2KB/patient3_1.chr20_63292355_63292377.2KB.pdf]

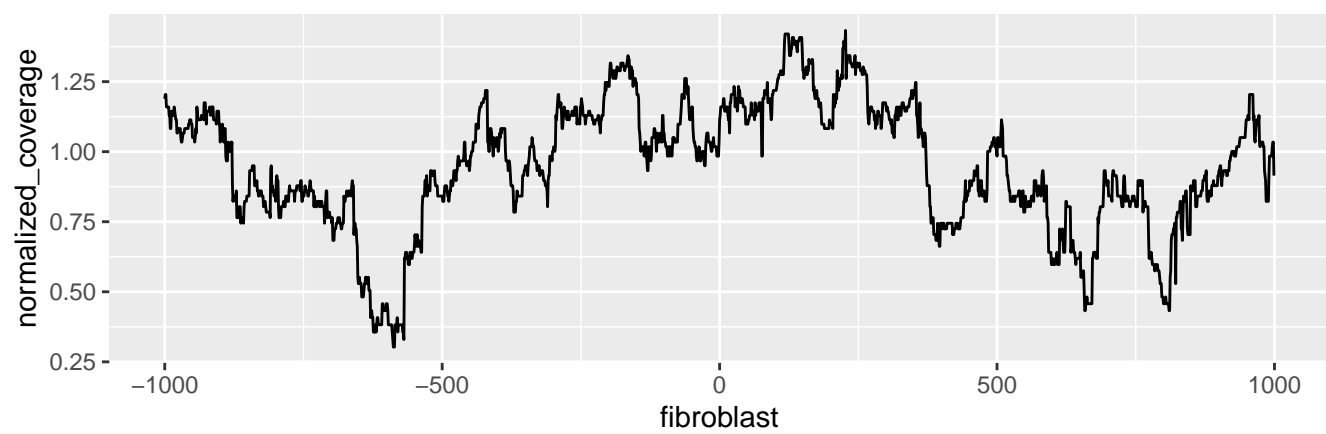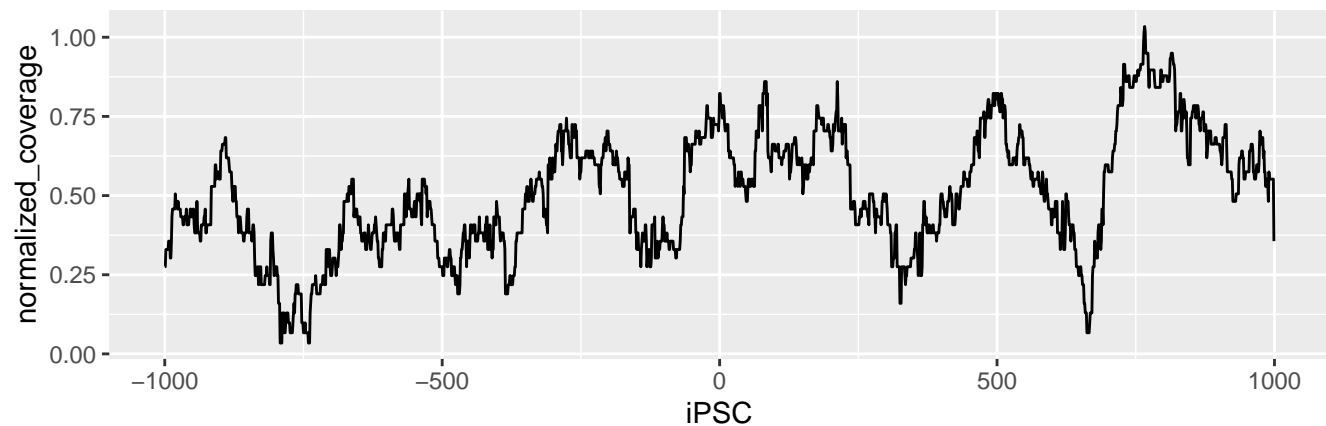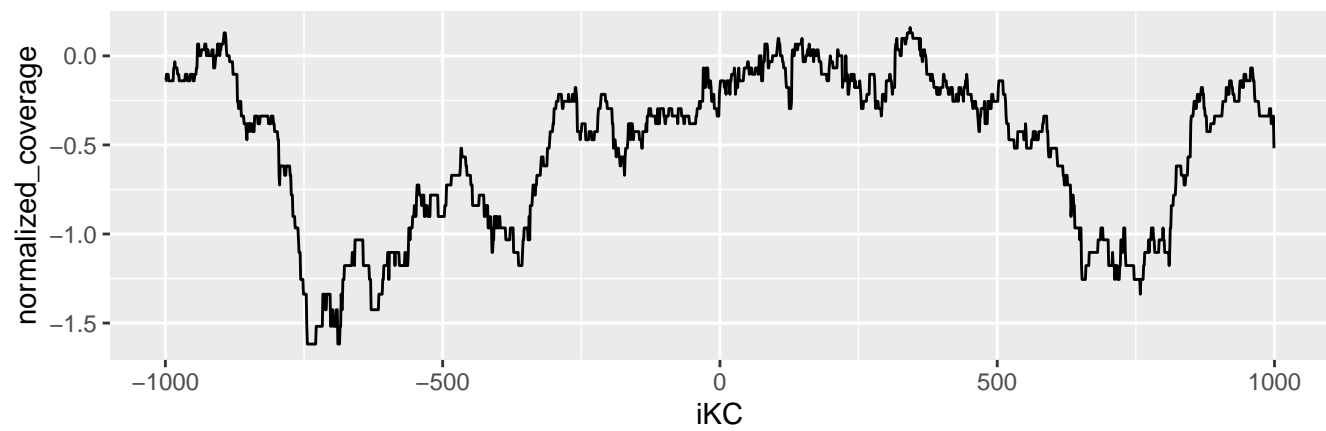

Supplement: Supplementary file 6 — Supplementary Data 3 [file 41467_2024_49400_MOESM6_ESM.zip › Supplementary Data 3/57_offtarget_sites/CO1-131_2KB/patient3_1.chr21_44957643_44957665.2KB.pdf]

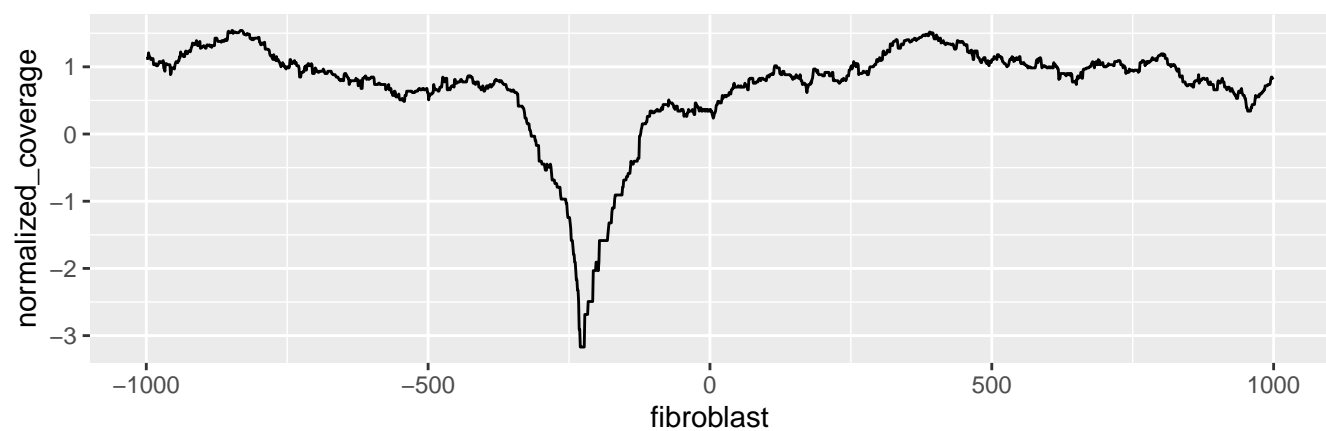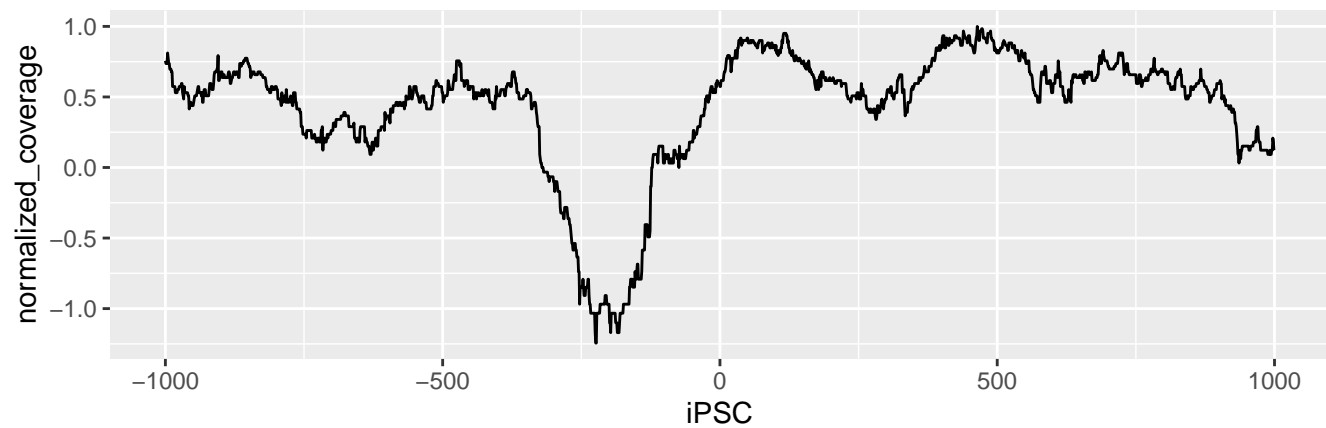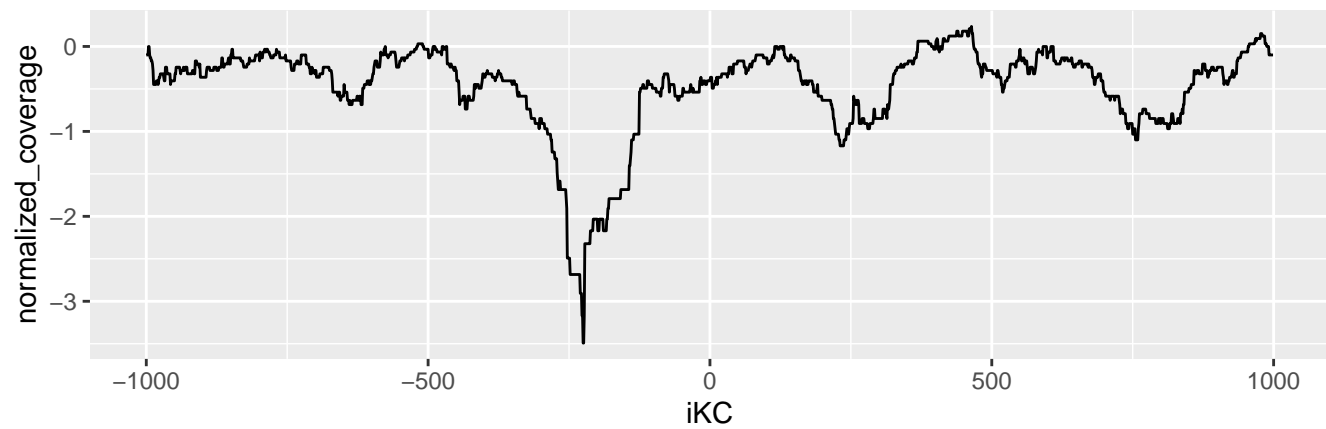

Supplement: Supplementary file 6 — Supplementary Data 3 [file 41467_2024_49400_MOESM6_ESM.zip › Supplementary Data 3/57_offtarget_sites/CO1-131_2KB/patient3_1.chr22_21956299_21956321.2KB.pdf]

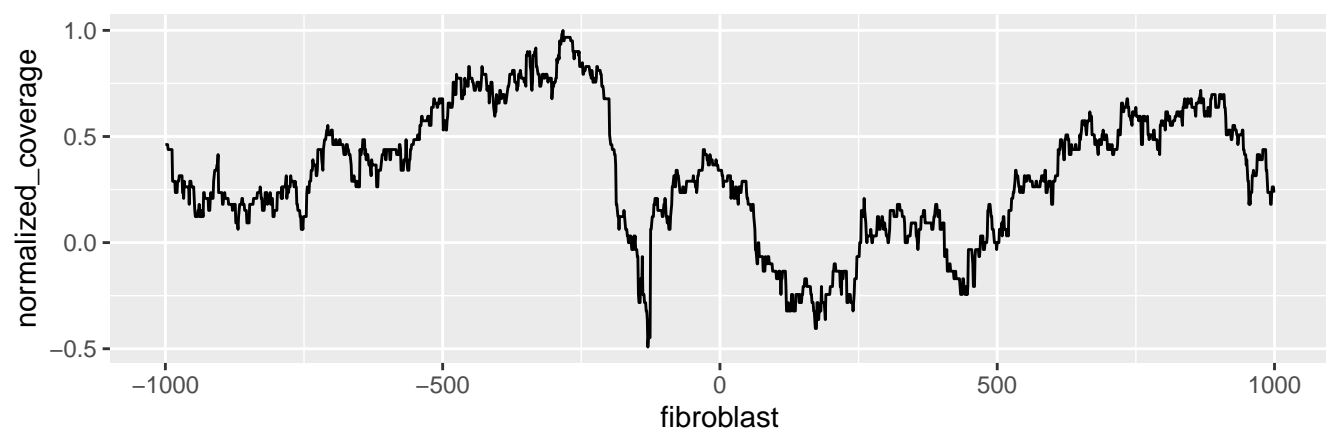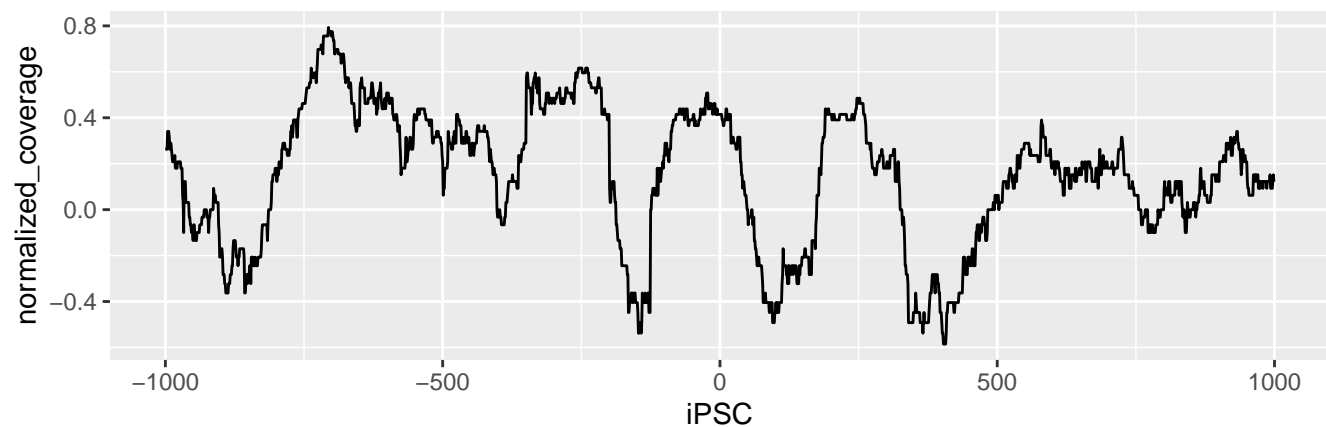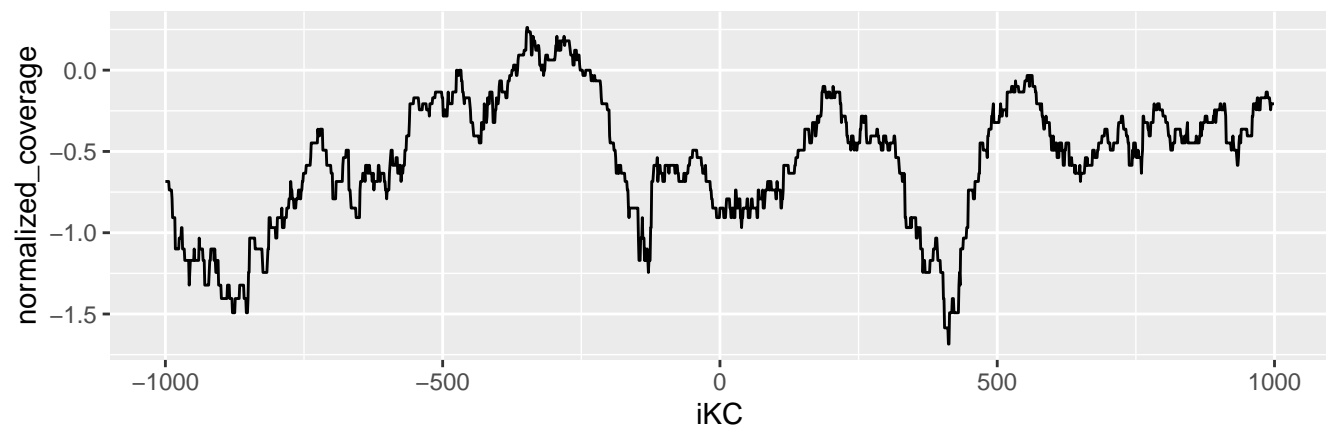

Supplement: Supplementary file 6 — Supplementary Data 3 [file 41467_2024_49400_MOESM6_ESM.zip › Supplementary Data 3/57_offtarget_sites/CO1-131_2KB/patient3_1.chr22_22222371_22222393.2KB.pdf]

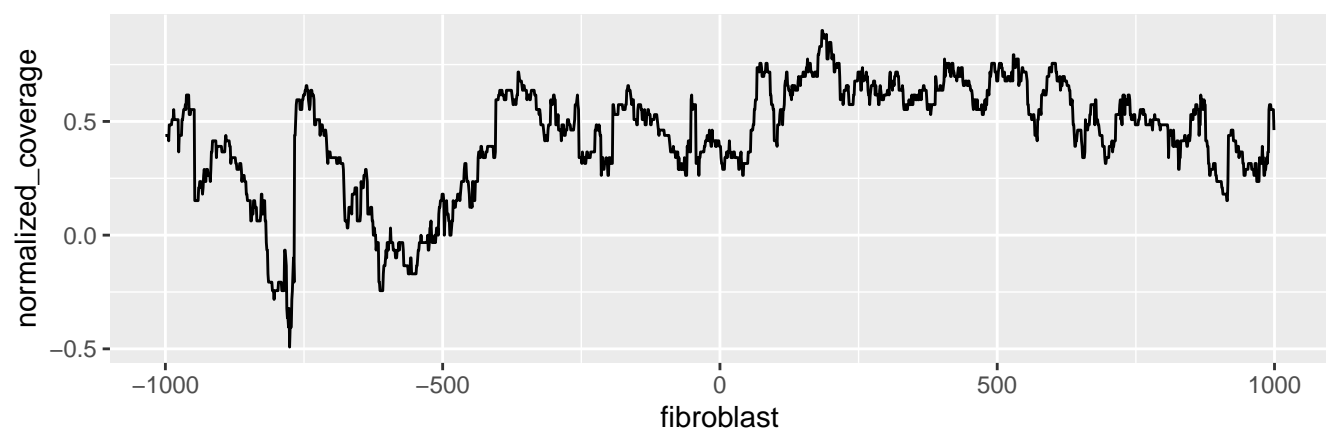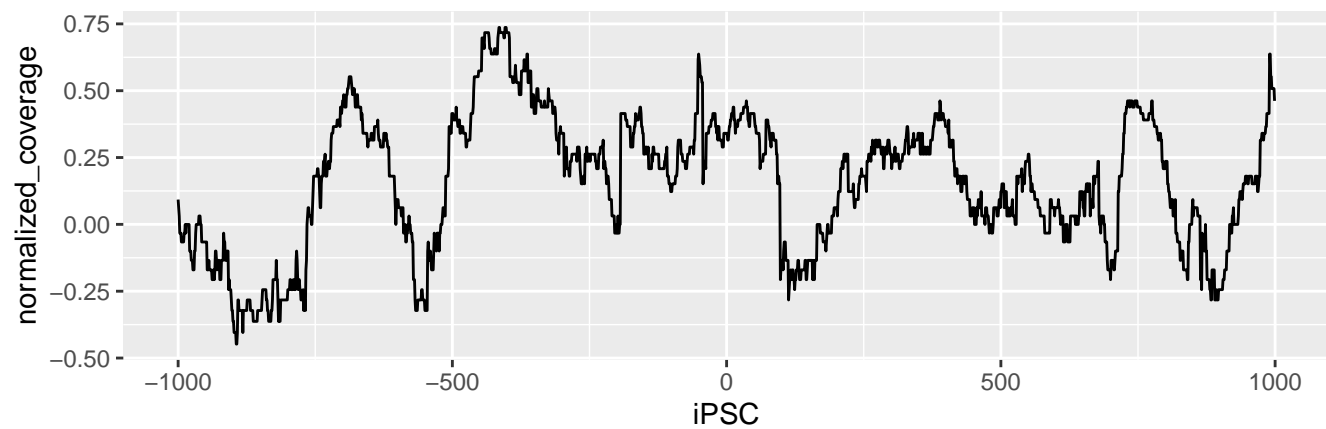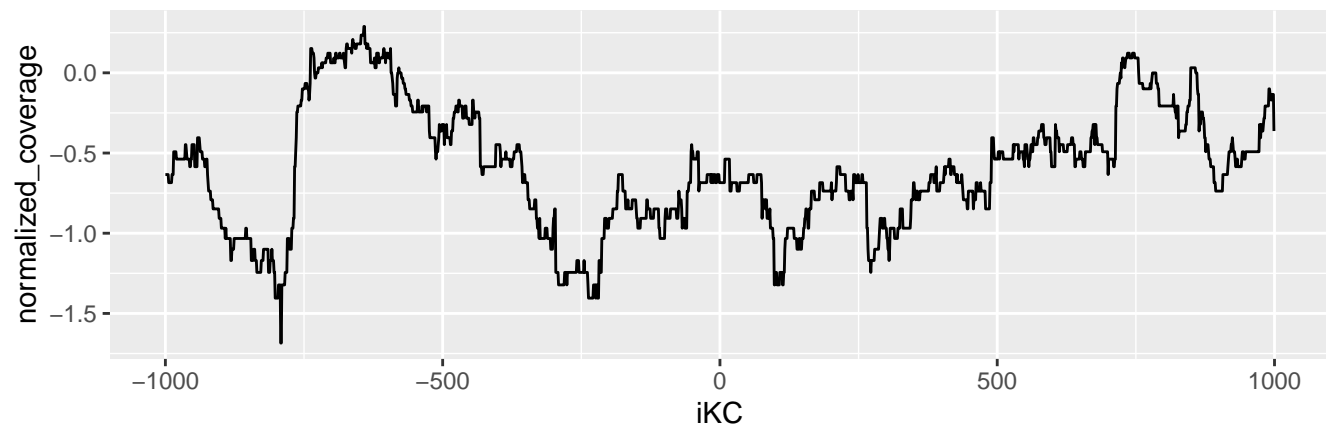

Supplement: Supplementary file 6 — Supplementary Data 3 [file 41467_2024_49400_MOESM6_ESM.zip › Supplementary Data 3/57_offtarget_sites/CO1-131_2KB/patient3_1.chr22_39714438_39714460.2KB.pdf]

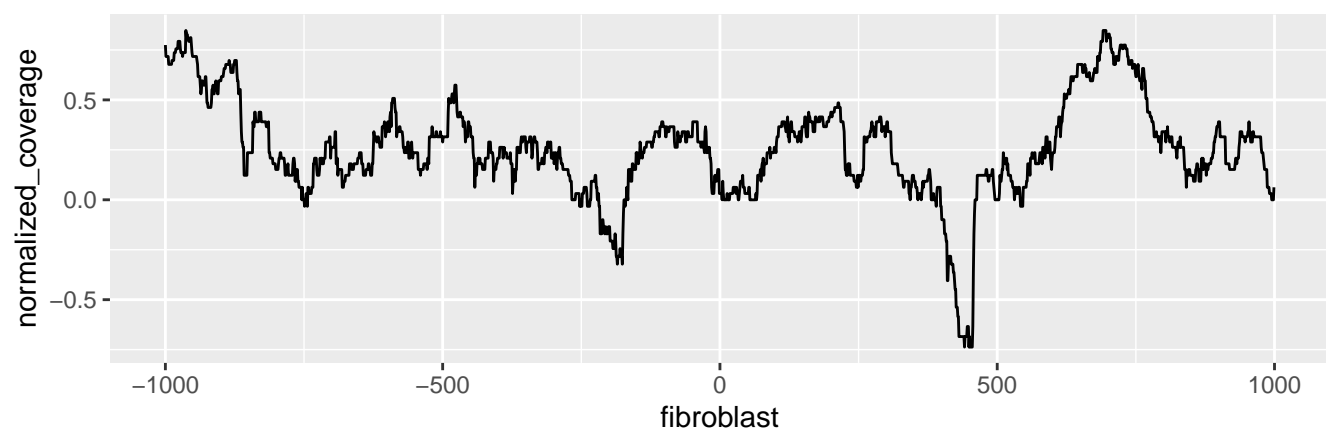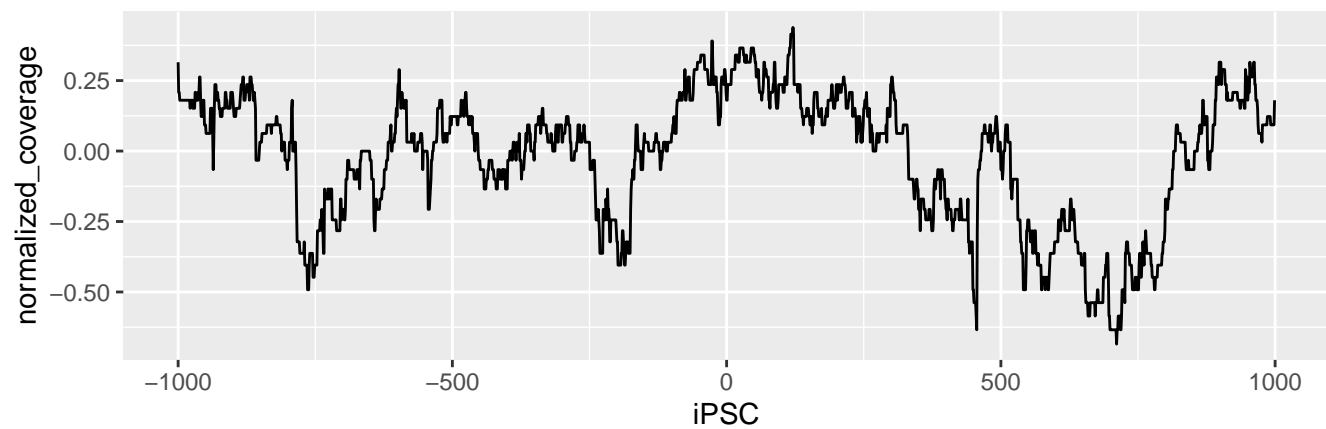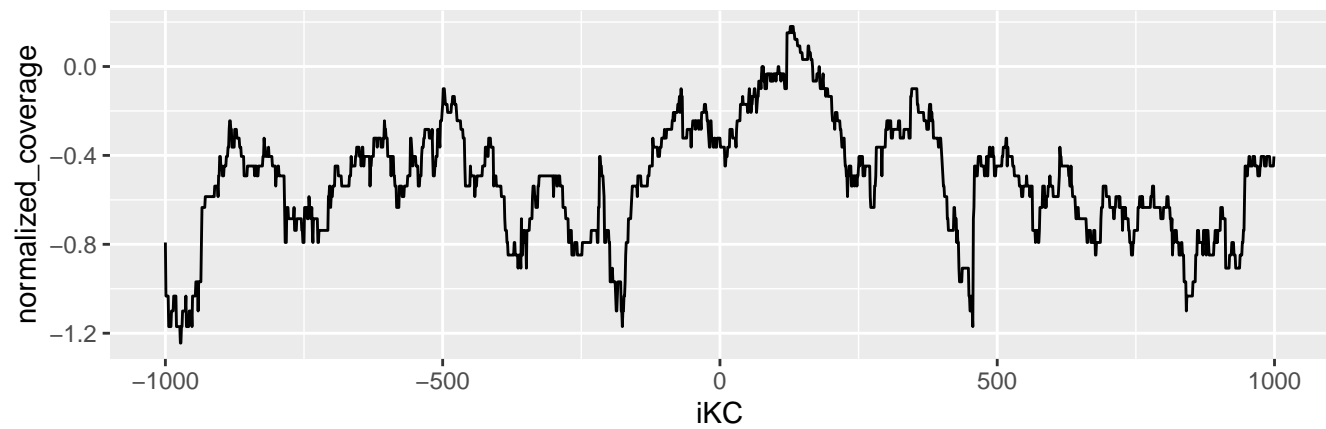

Supplement: Supplementary file 6 — Supplementary Data 3 [file 41467_2024_49400_MOESM6_ESM.zip › Supplementary Data 3/57_offtarget_sites/CO1-131_2KB/patient3_1.chr22_44431399_44431421.2KB.pdf]

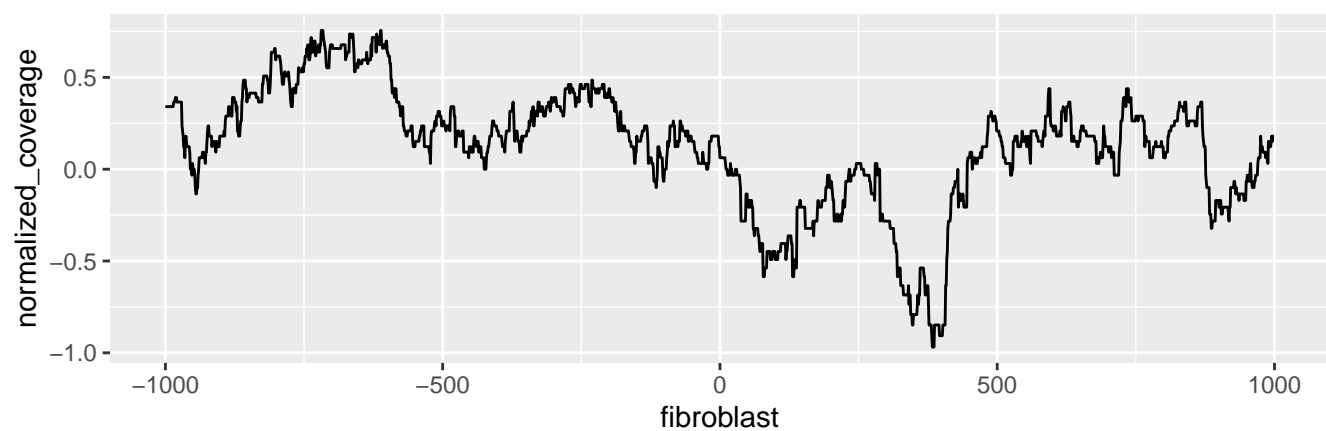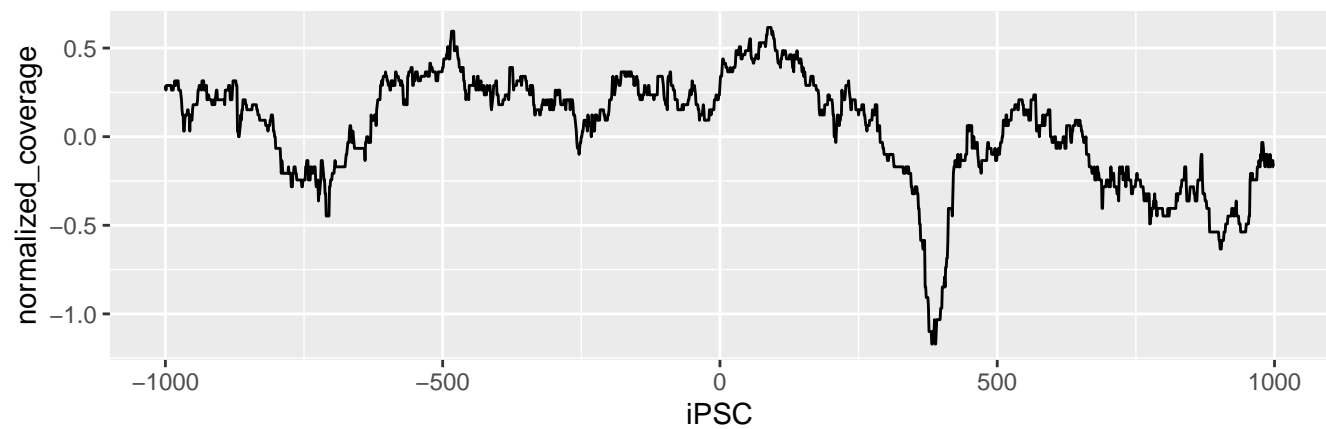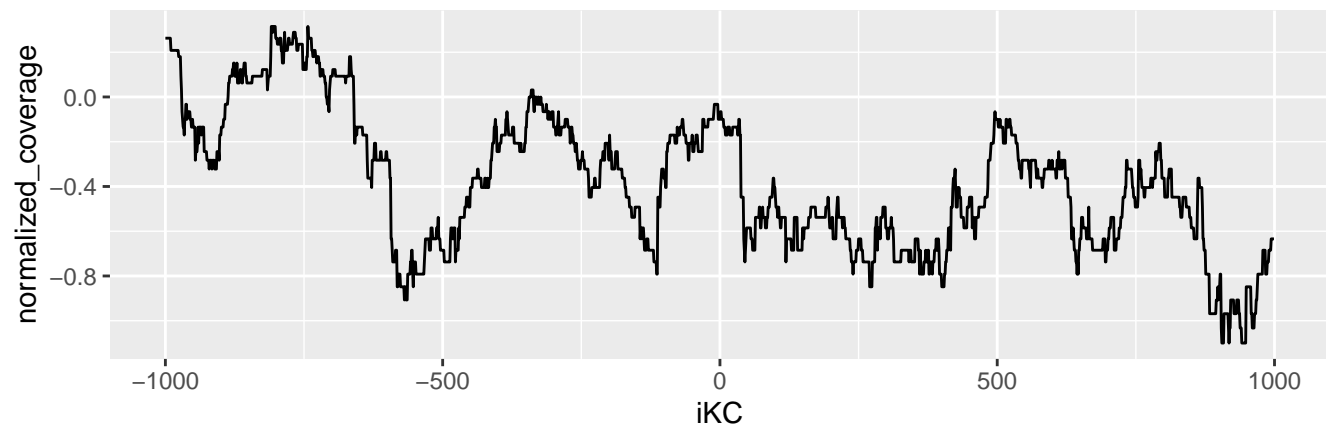

Supplement: Supplementary file 6 — Supplementary Data 3 [file 41467_2024_49400_MOESM6_ESM.zip › Supplementary Data 3/57_offtarget_sites/CO1-131_2KB/patient3_1.chr22_49848657_49848679.2KB.pdf]

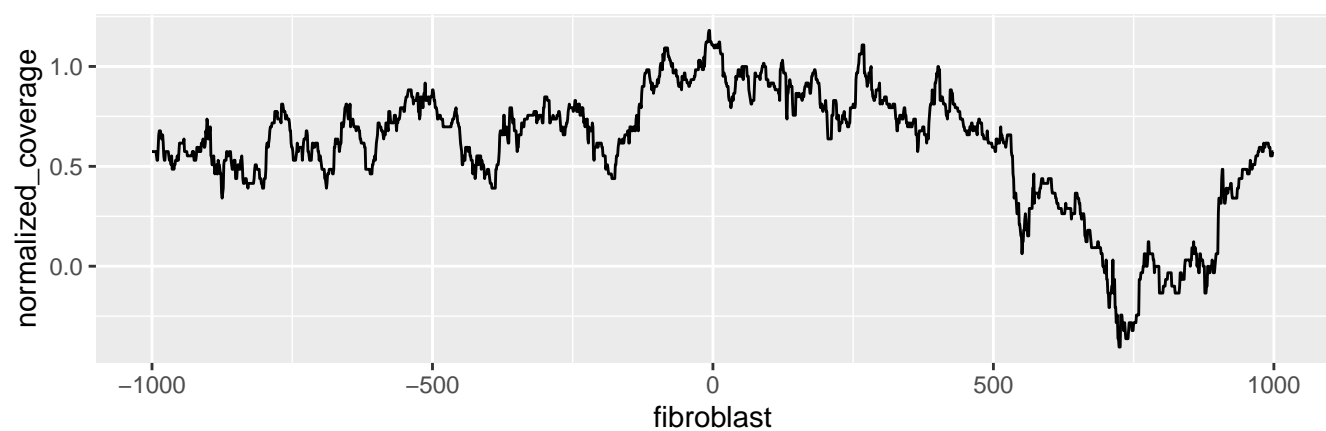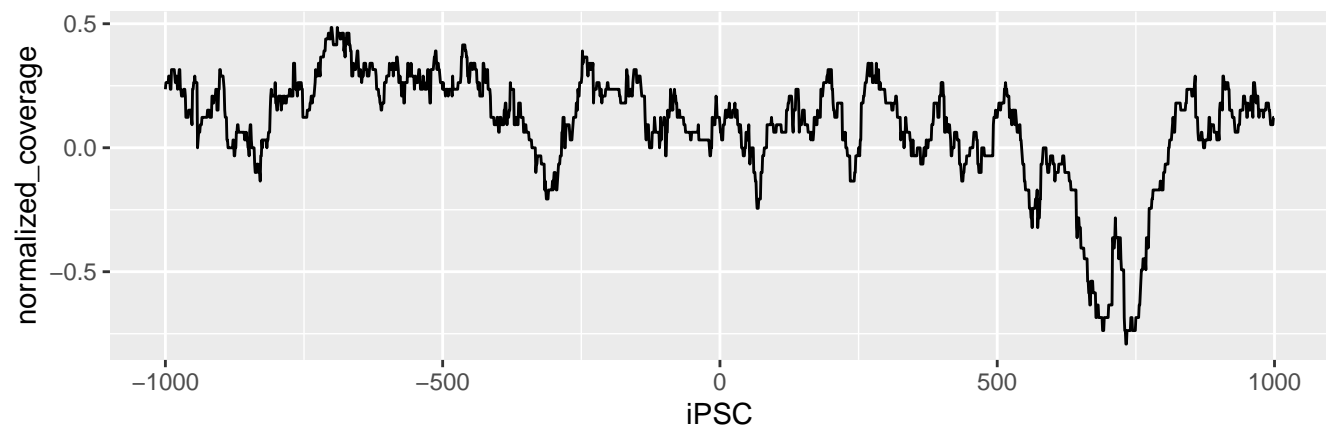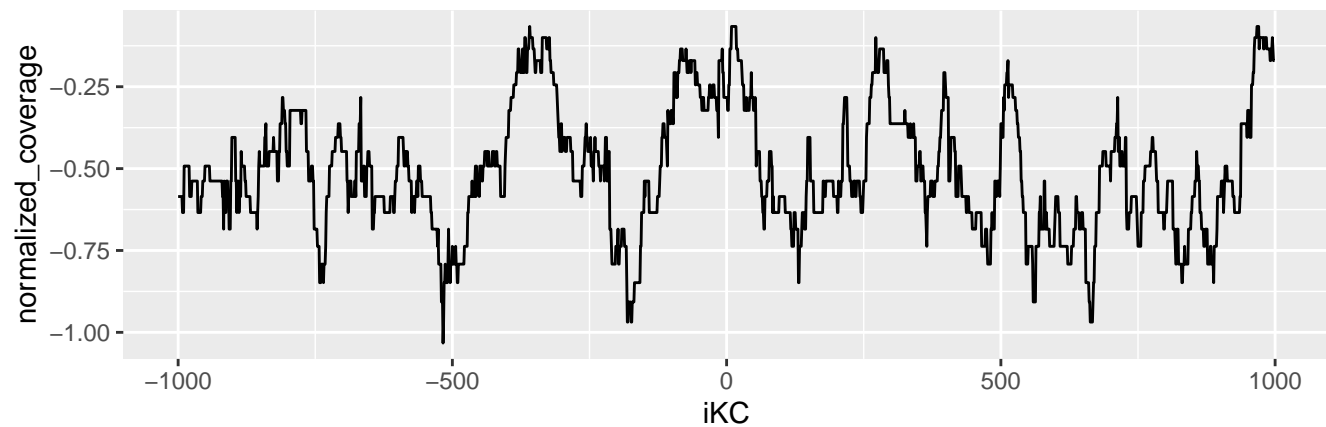

Supplement: Supplementary file 6 — Supplementary Data 3 [file 41467_2024_49400_MOESM6_ESM.zip › Supplementary Data 3/57_offtarget_sites/CO1-131_2KB/patient3_1.chr22_49858552_49858574.2KB.pdf]

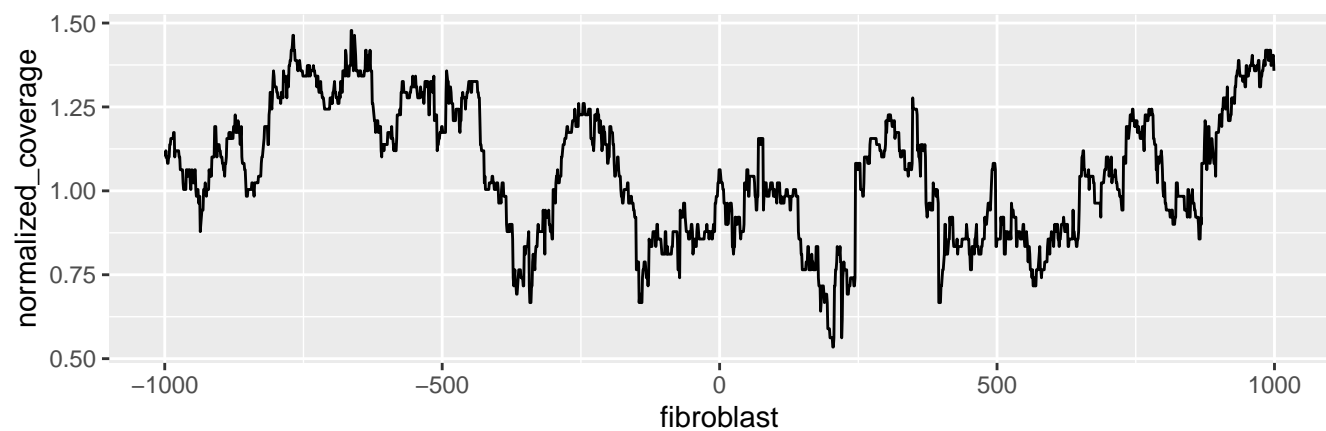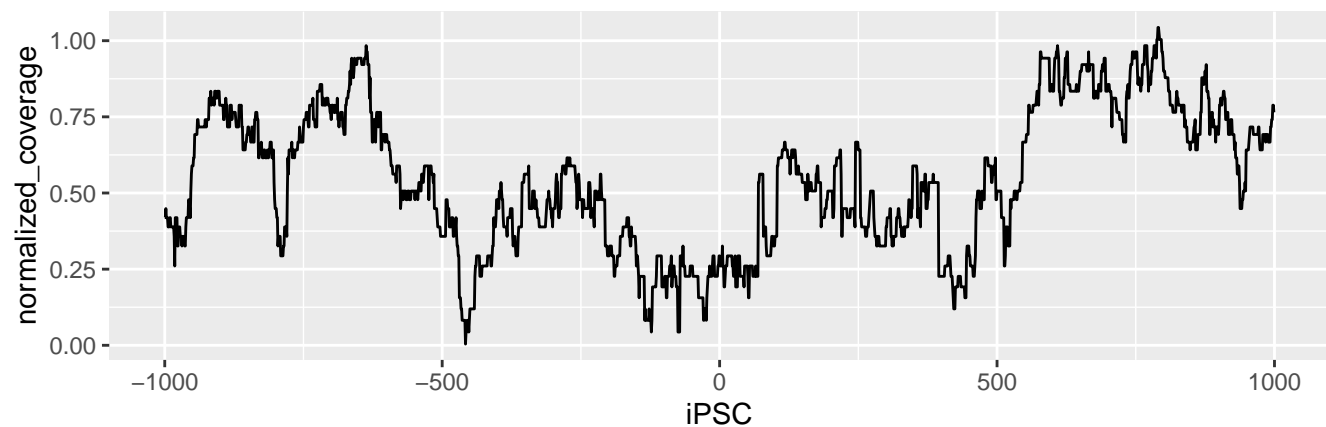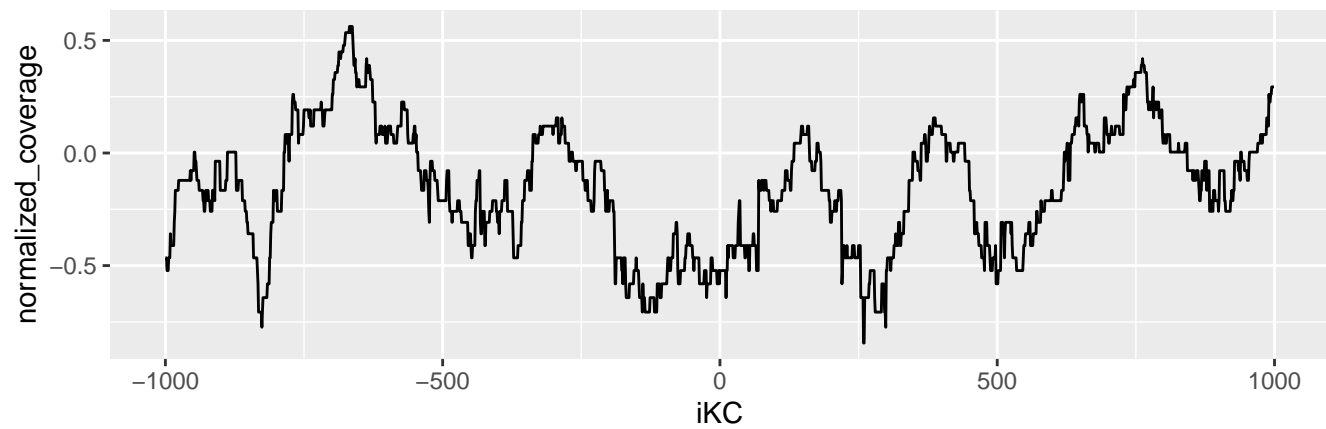

Supplement: Supplementary file 6 — Supplementary Data 3 [file 41467_2024_49400_MOESM6_ESM.zip › Supplementary Data 3/57_offtarget_sites/CO1-131_2KB/patient3_1.chr3_127031465_127031487.2KB.pdf]

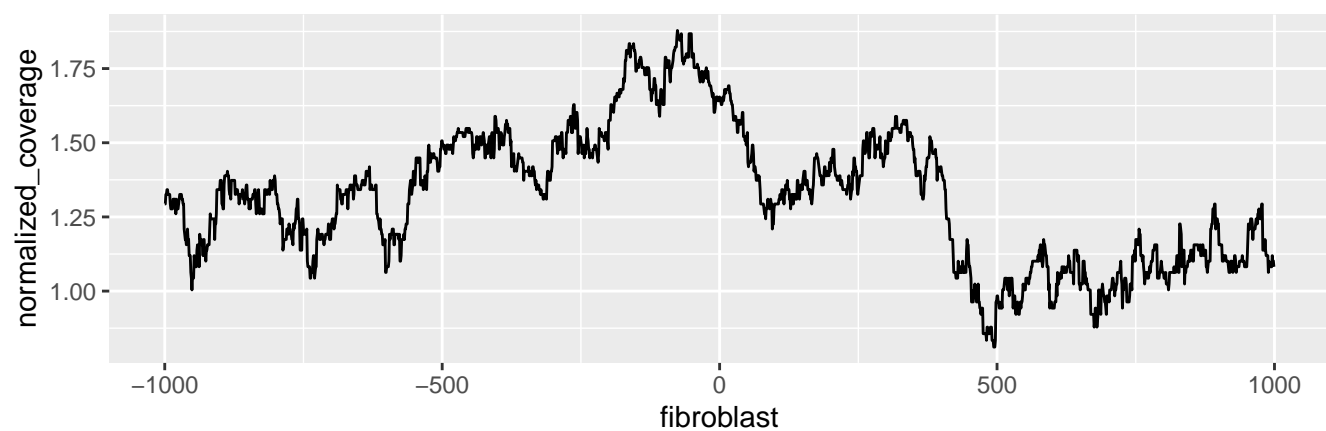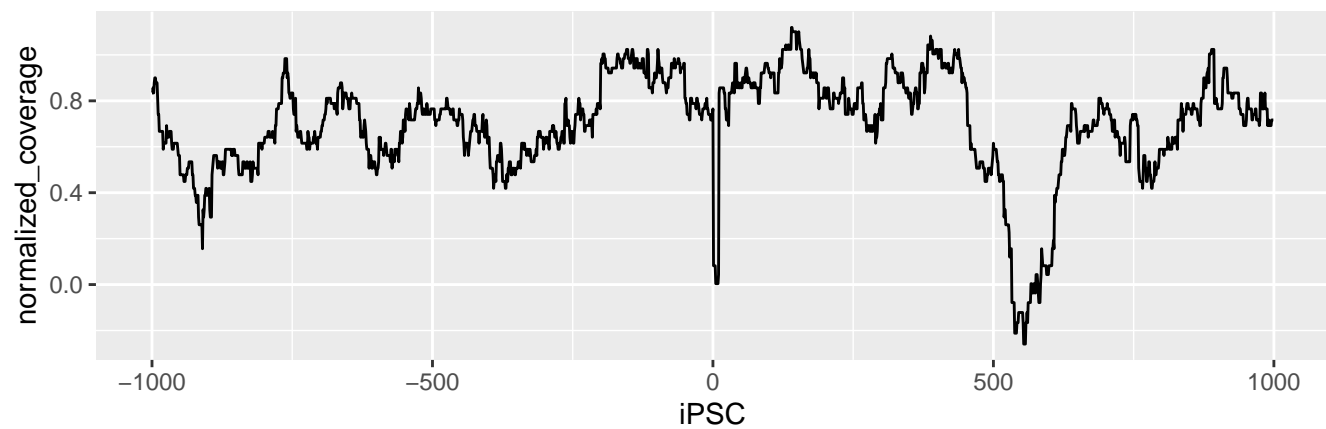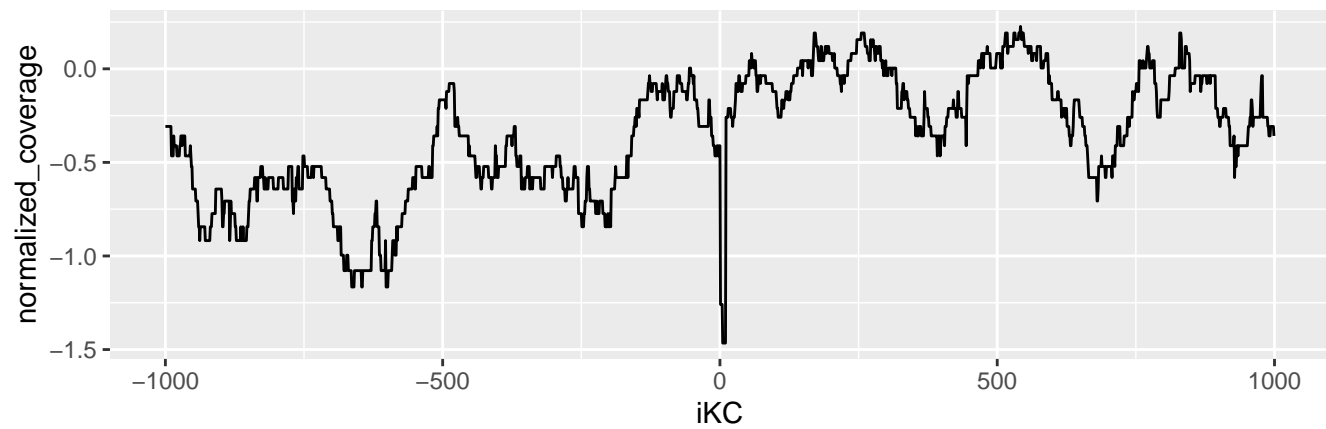

Supplement: Supplementary file 6 — Supplementary Data 3 [file 41467_2024_49400_MOESM6_ESM.zip › Supplementary Data 3/57_offtarget_sites/CO1-131_2KB/patient3_1.chr3_48570118_48570140.2KB.pdf]

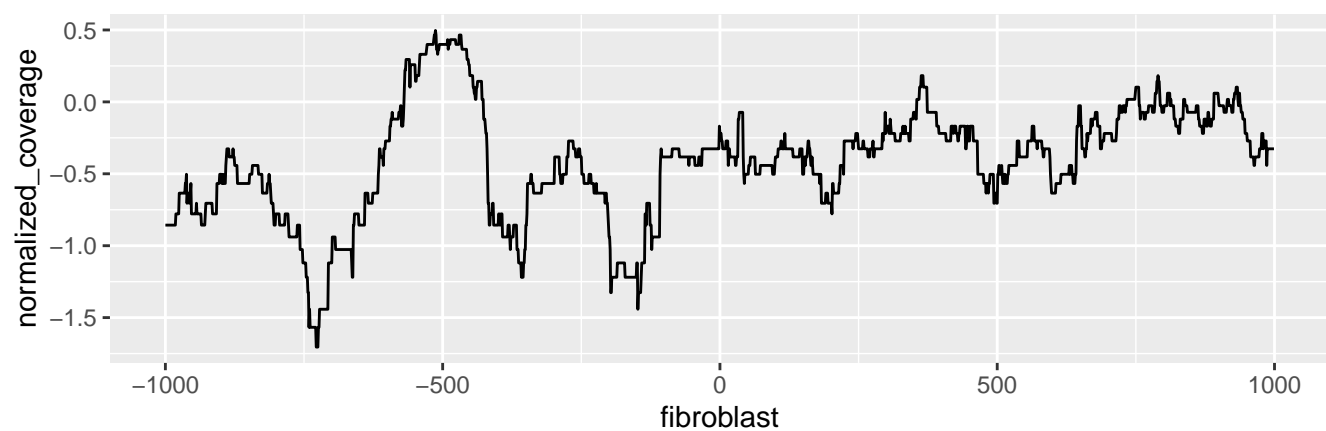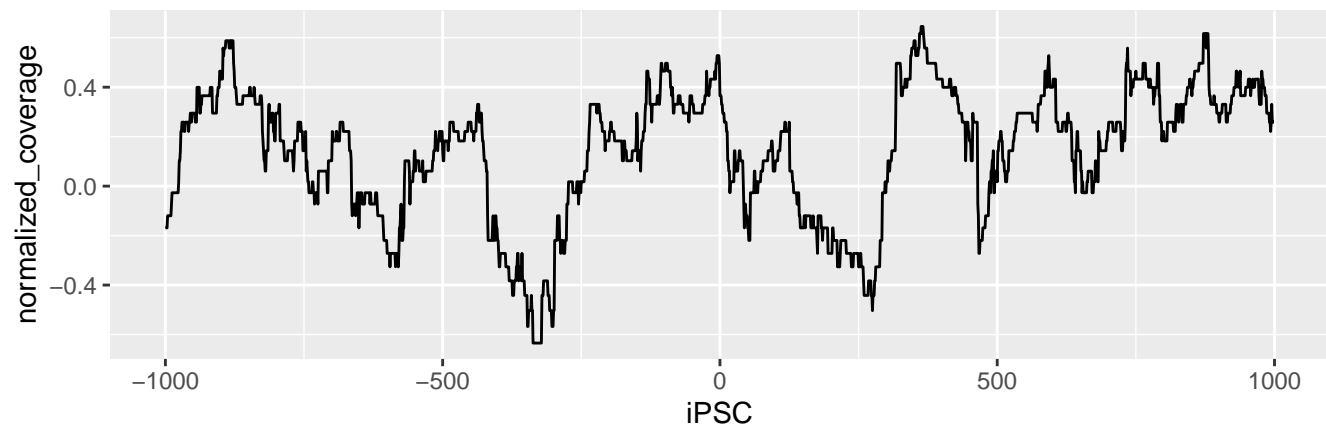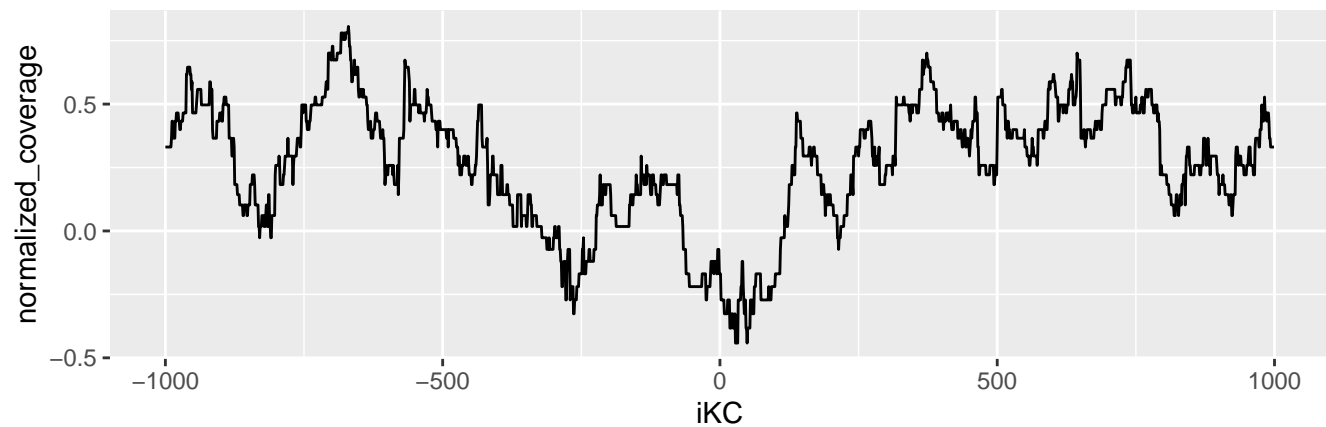

Supplement: Supplementary file 6 — Supplementary Data 3 [file 41467_2024_49400_MOESM6_ESM.zip › Supplementary Data 3/57_offtarget_sites/CO1-131_2KB/patient3_1.chr4_34873979_34874001.2KB.pdf]

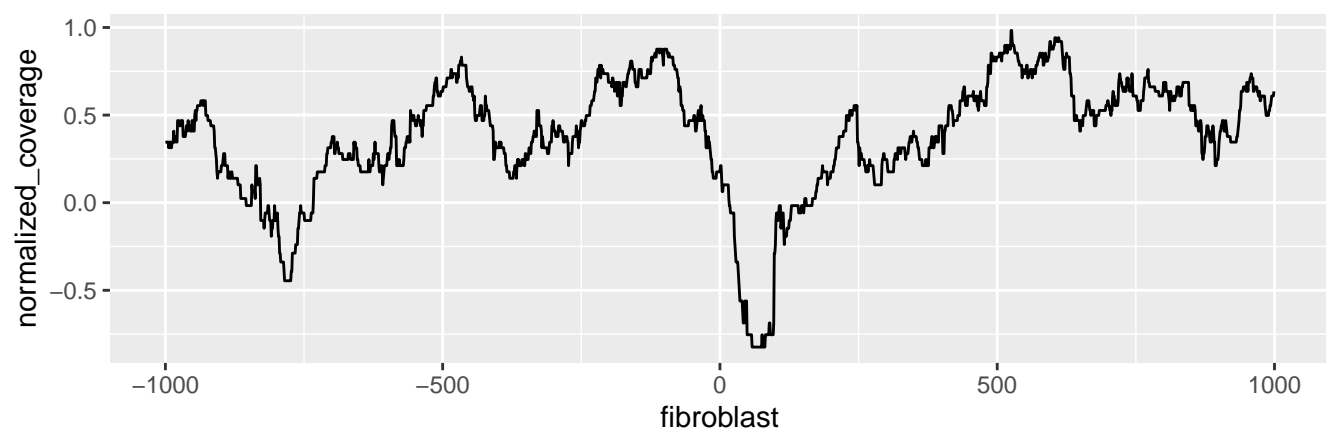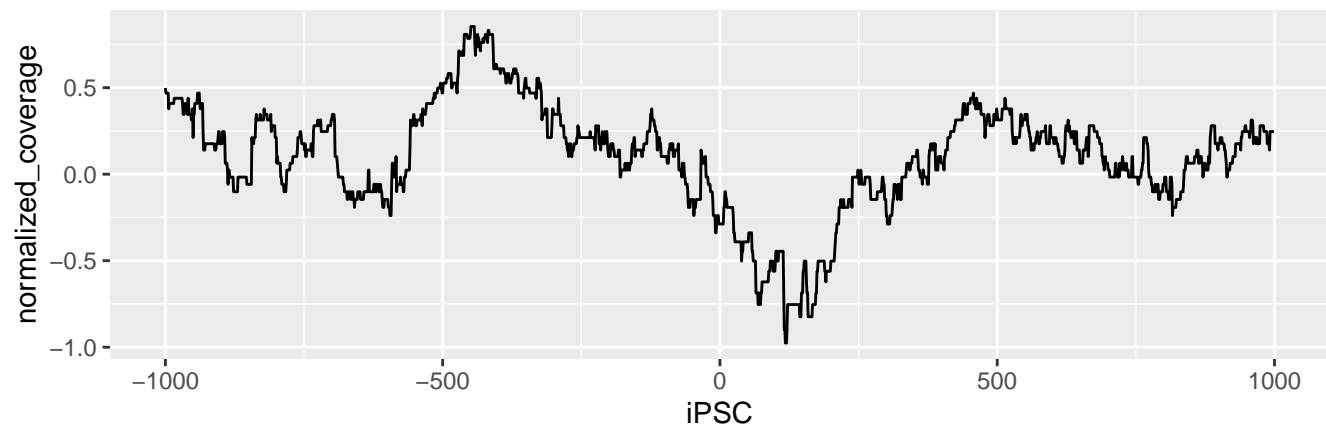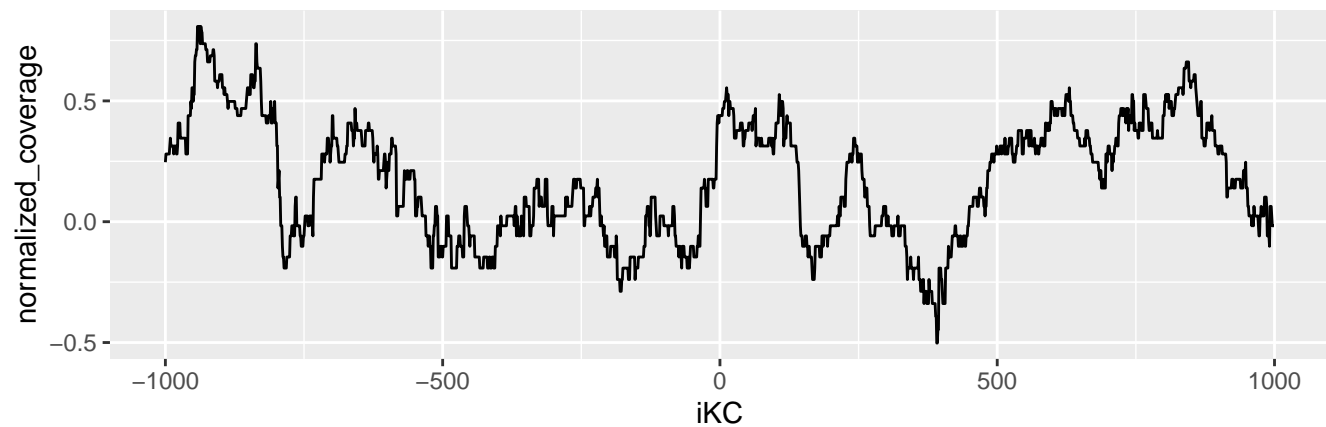

Supplement: Supplementary file 6 — Supplementary Data 3 [file 41467_2024_49400_MOESM6_ESM.zip › Supplementary Data 3/57_offtarget_sites/CO1-131_2KB/patient3_1.chr6_148779037_148779059.2KB.pdf]

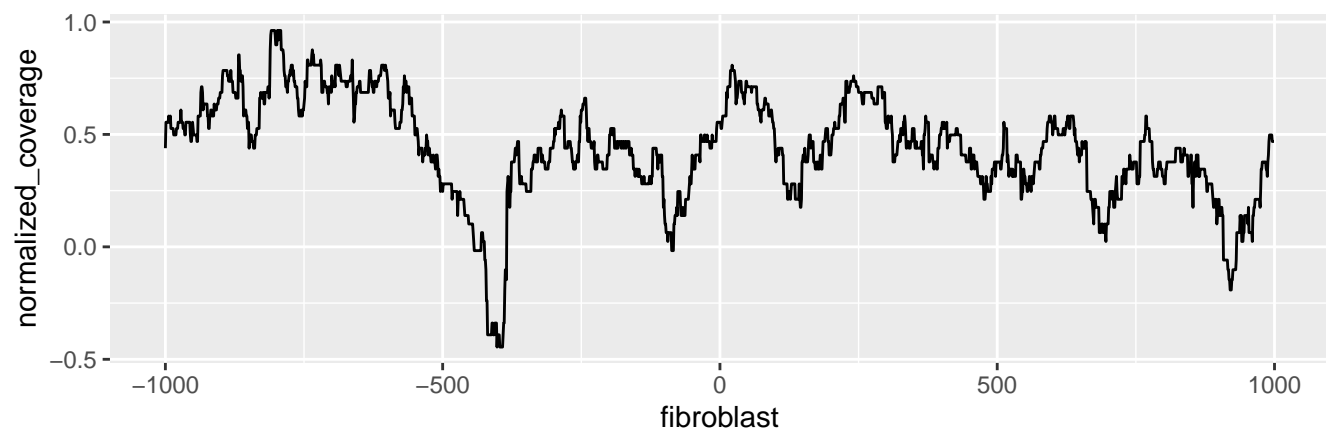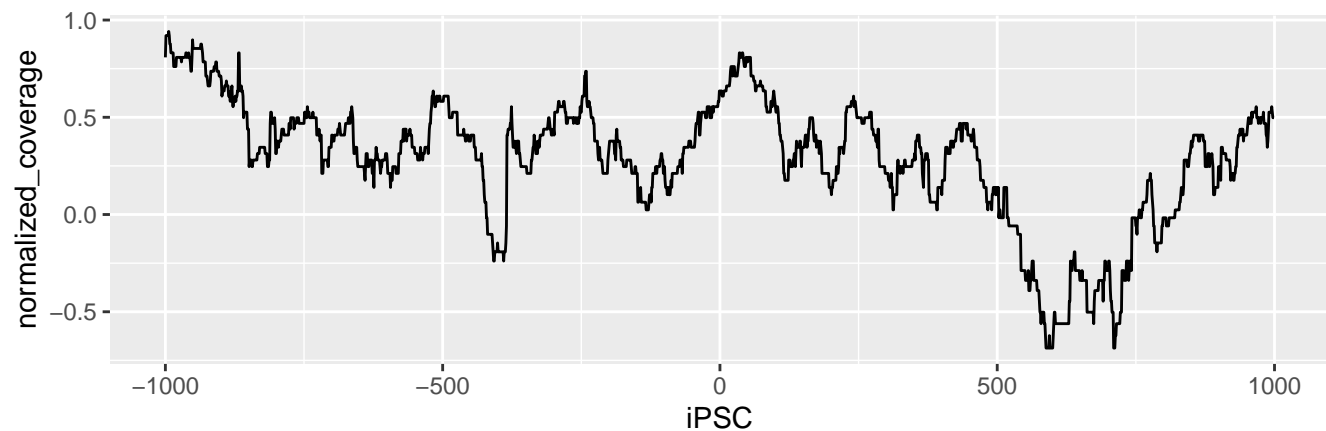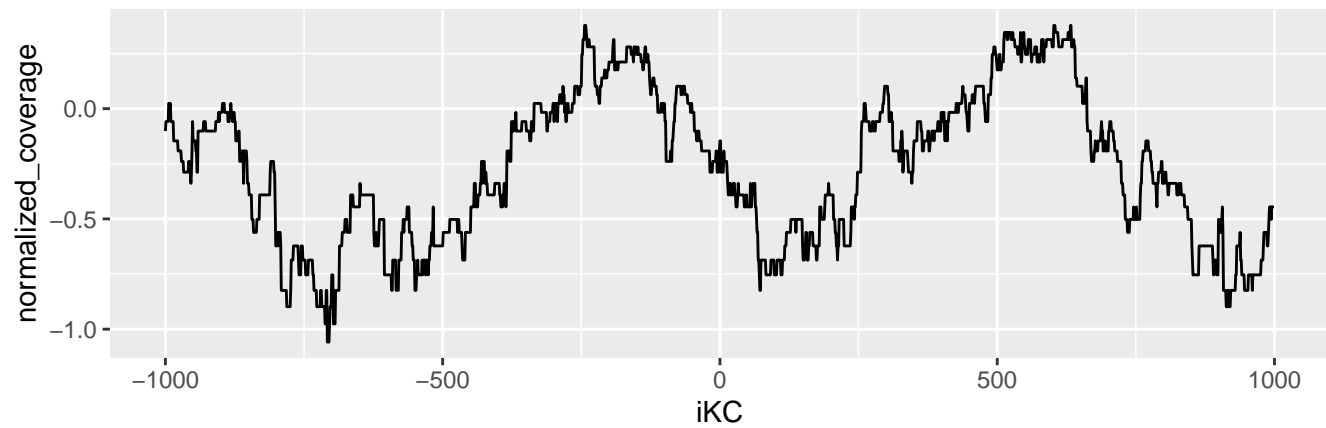

Supplement: Supplementary file 6 — Supplementary Data 3 [file 41467_2024_49400_MOESM6_ESM.zip › Supplementary Data 3/57_offtarget_sites/CO1-131_2KB/patient3_1.chr6_151241153_151241175.2KB.pdf]

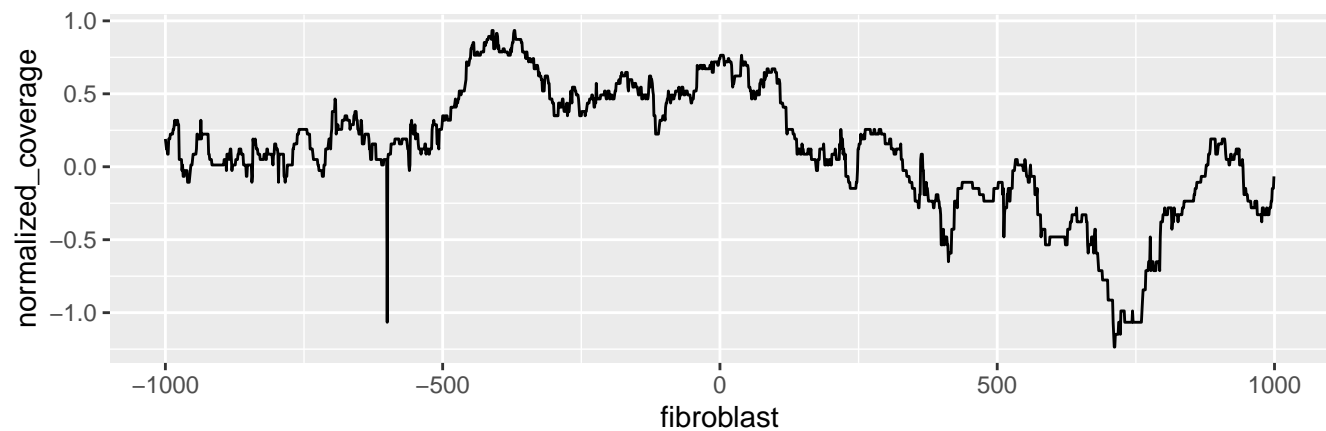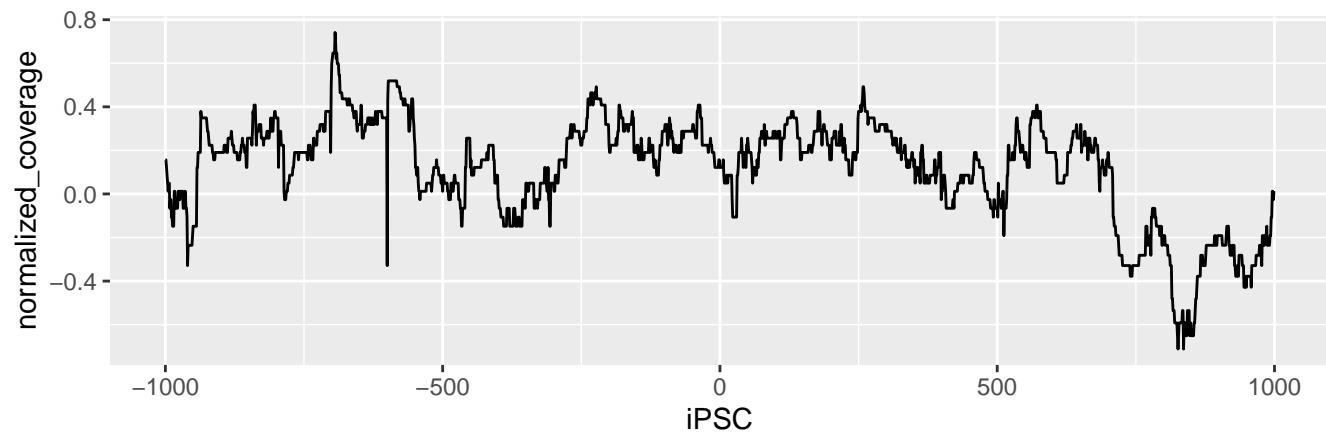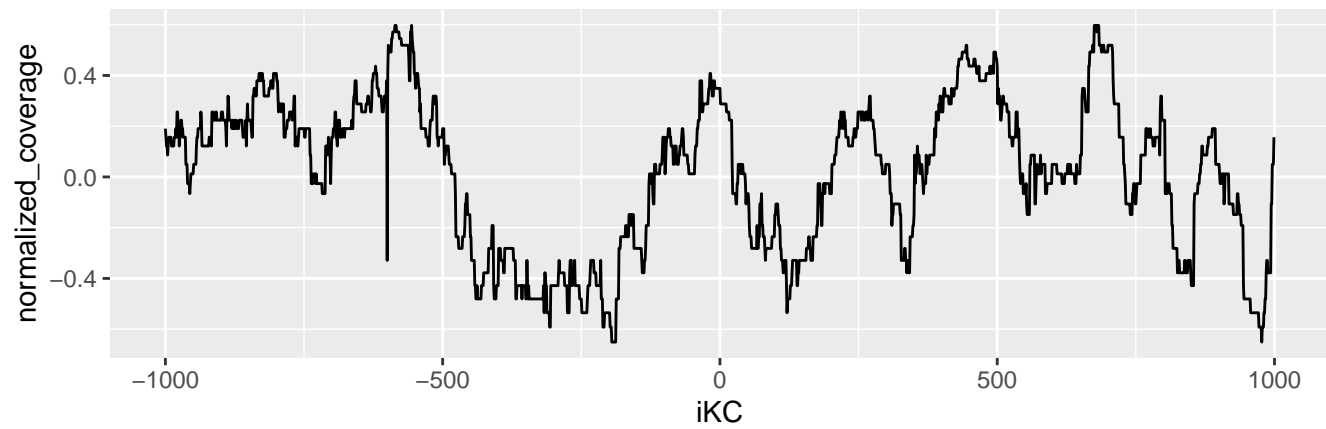

Supplement: Supplementary file 6 — Supplementary Data 3 [file 41467_2024_49400_MOESM6_ESM.zip › Supplementary Data 3/57_offtarget_sites/CO1-131_2KB/patient3_1.chr7_7567231_7567253.2KB.pdf]

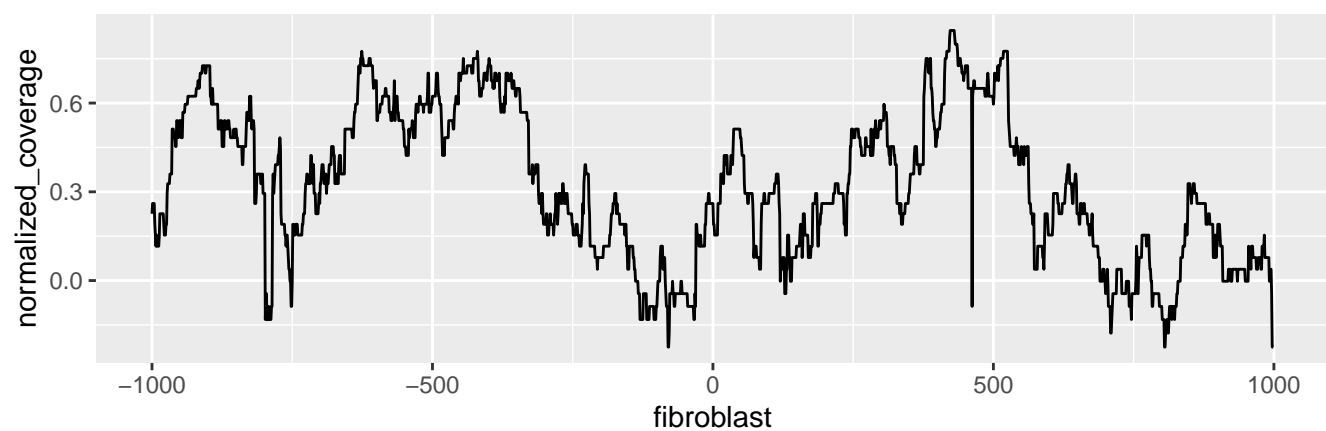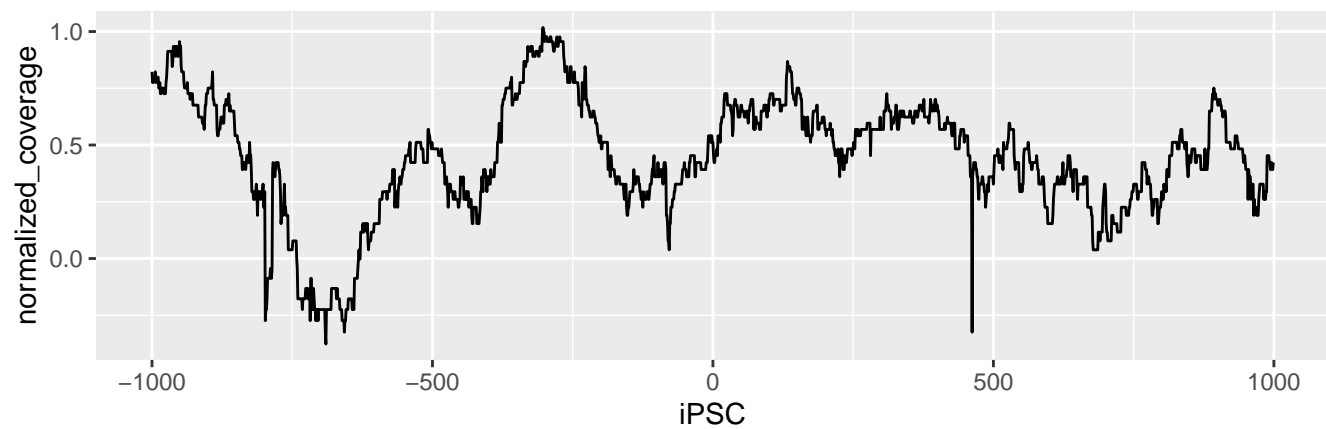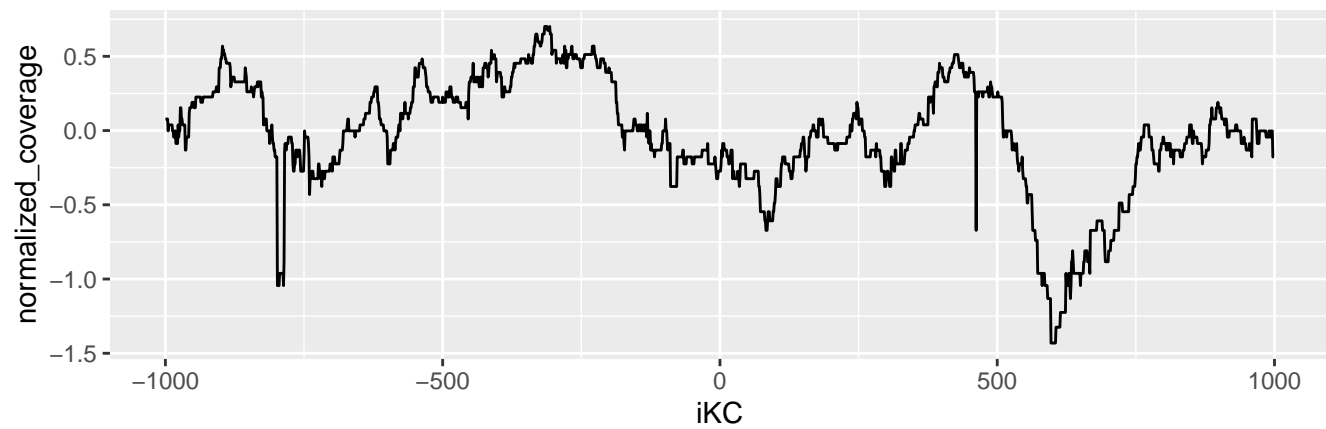

Supplement: Supplementary file 6 — Supplementary Data 3 [file 41467_2024_49400_MOESM6_ESM.zip › Supplementary Data 3/57_offtarget_sites/CO1-131_2KB/patient3_1.chr8_7036812_7036834.2KB.pdf]

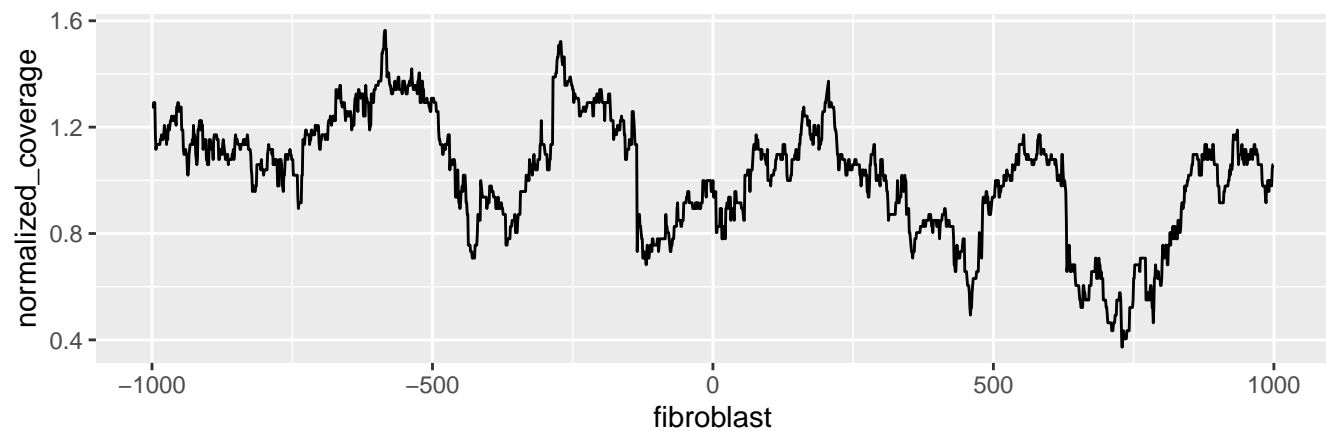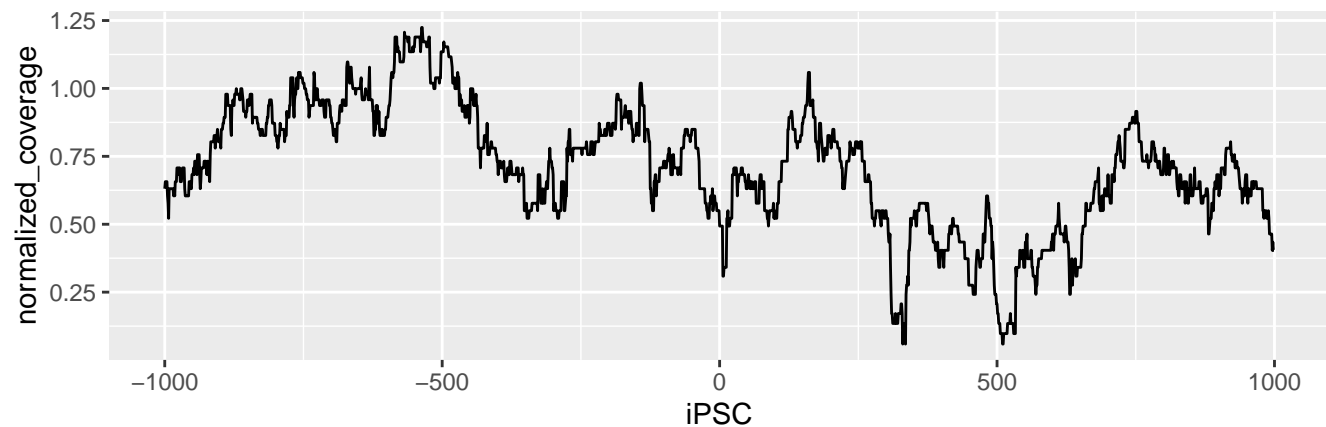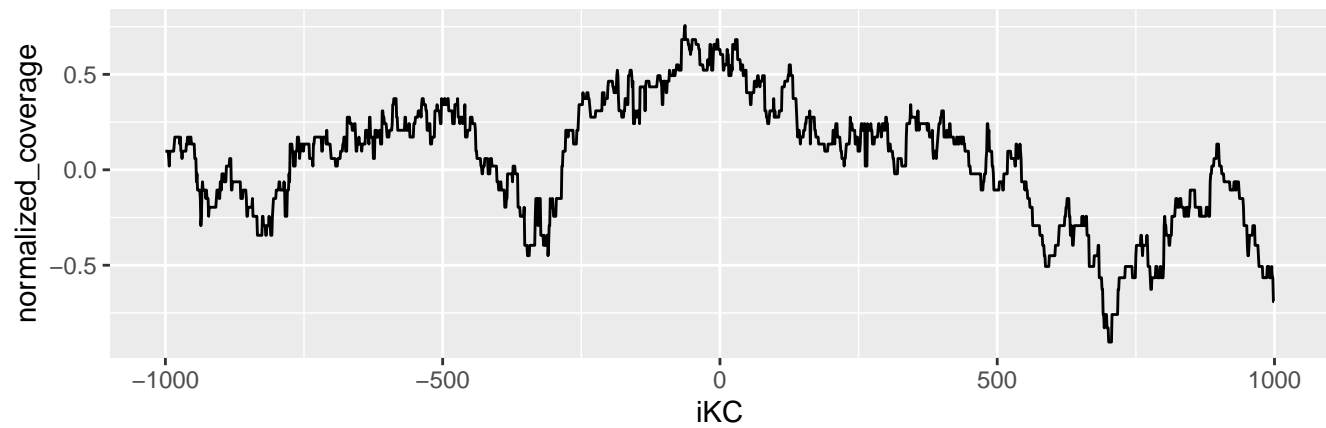

Supplement: Supplementary file 6 — Supplementary Data 3 [file 41467_2024_49400_MOESM6_ESM.zip › Supplementary Data 3/57_offtarget_sites/CO1-131_2KB/patient3_1.chr9_135086067_135086089.2KB.pdf]

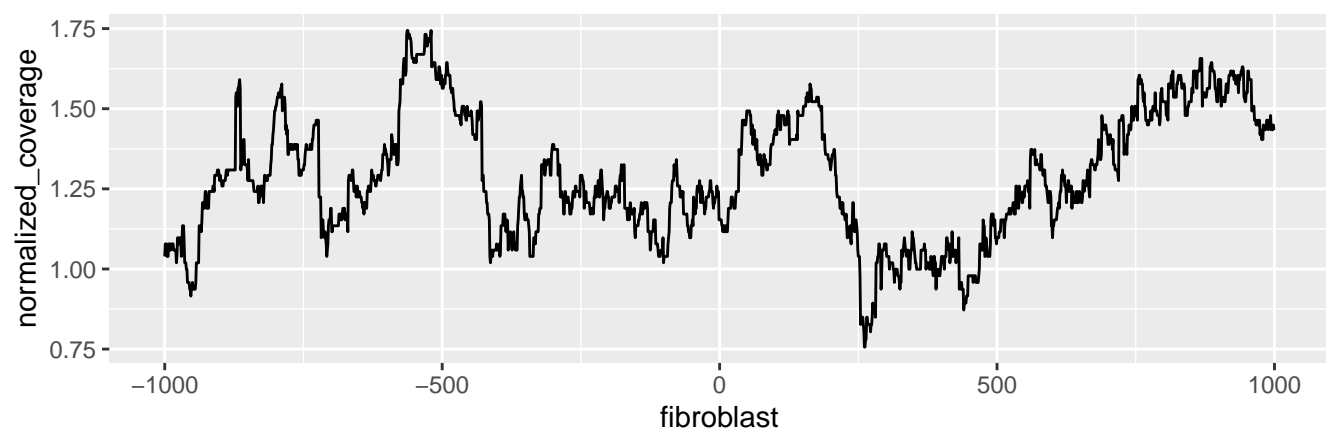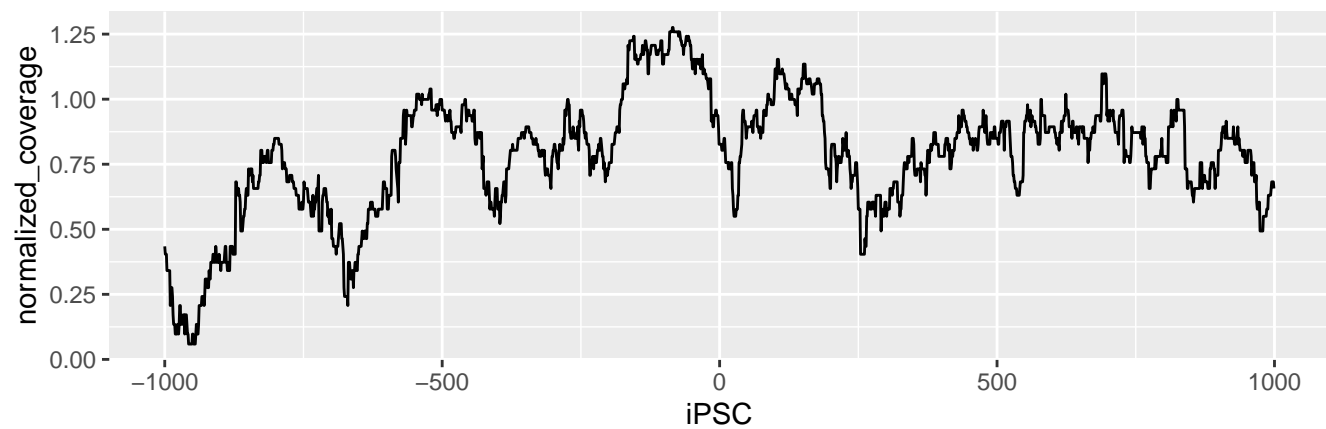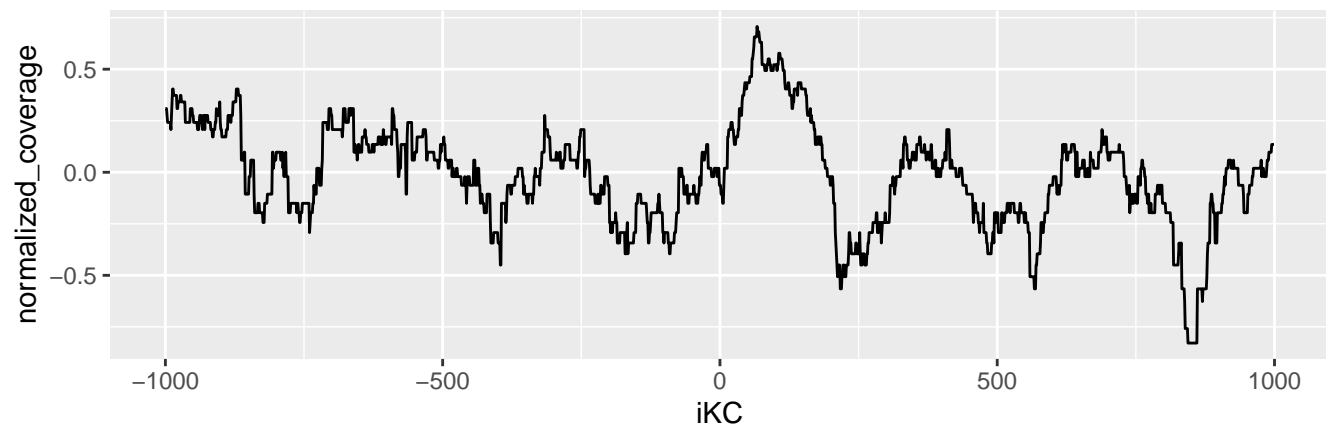

Supplement: Supplementary file 6 — Supplementary Data 3 [file 41467_2024_49400_MOESM6_ESM.zip › Supplementary Data 3/57_offtarget_sites/CO1-131_2KB/patient3_1.chr9_136509758_136509780.2KB.pdf]

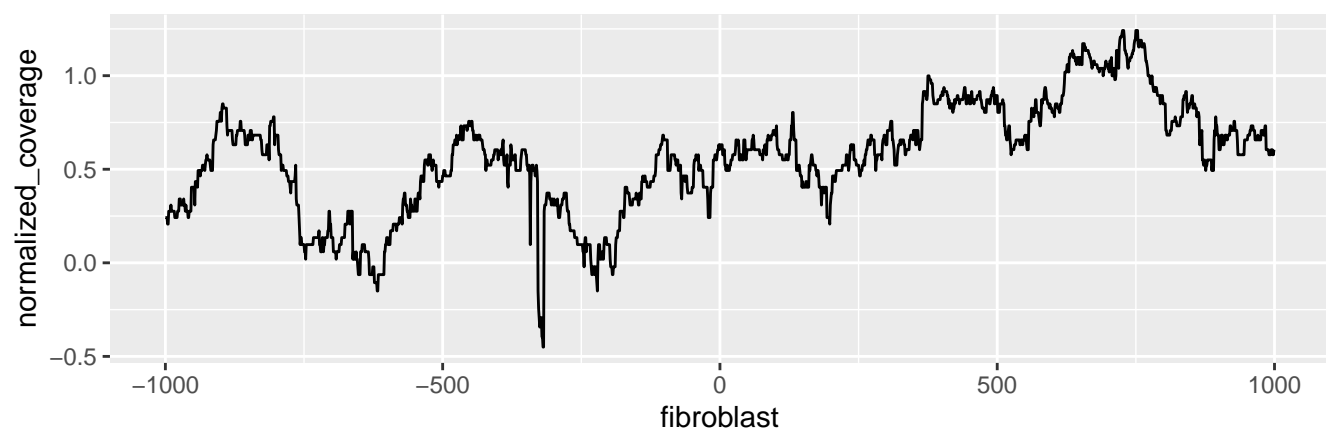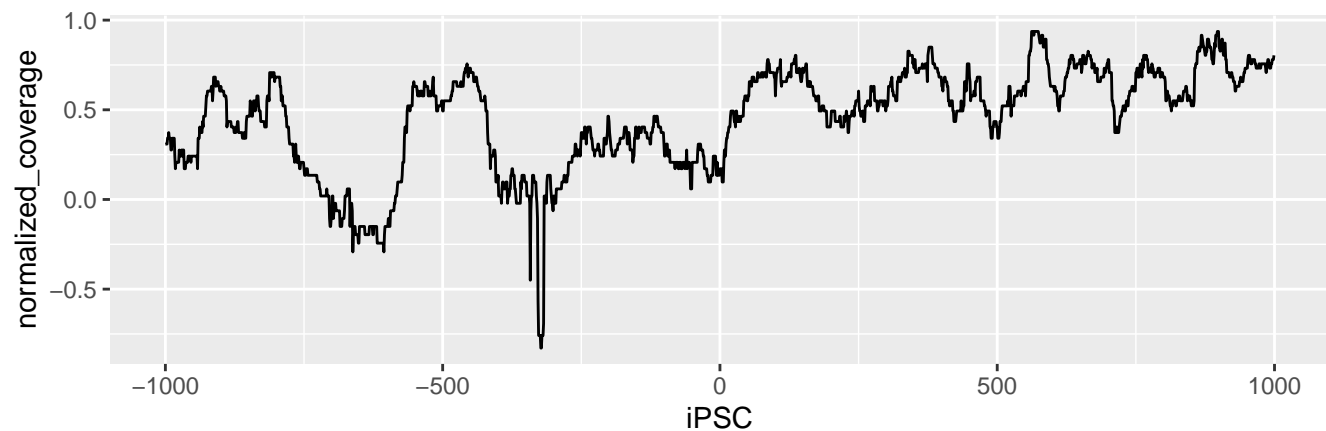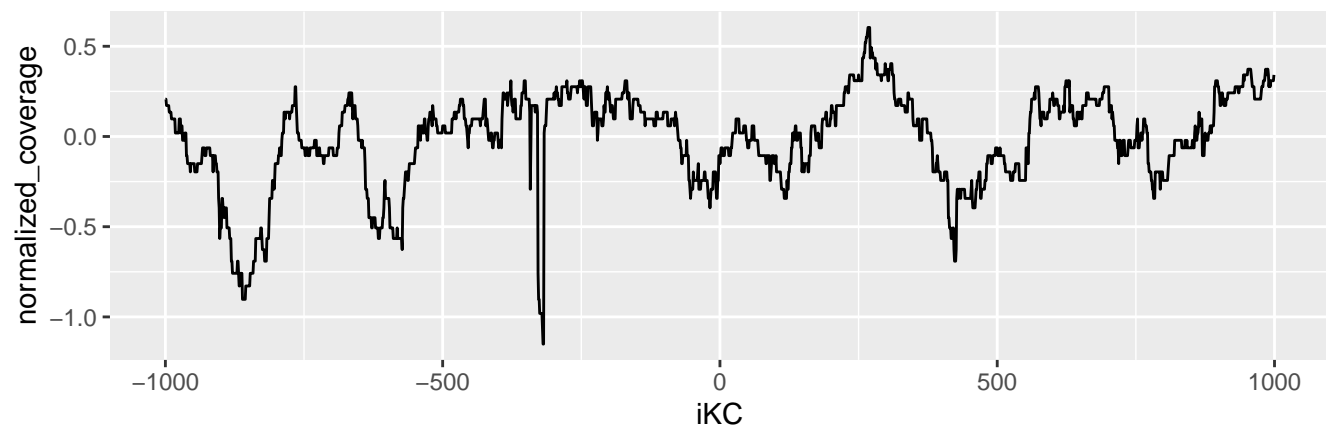

Supplement: Supplementary file 6 — Supplementary Data 3 [file 41467_2024_49400_MOESM6_ESM.zip › Supplementary Data 3/57_offtarget_sites/CO1-131_2KB/patient3_1.chr9_36989005_36989027.2KB.pdf]

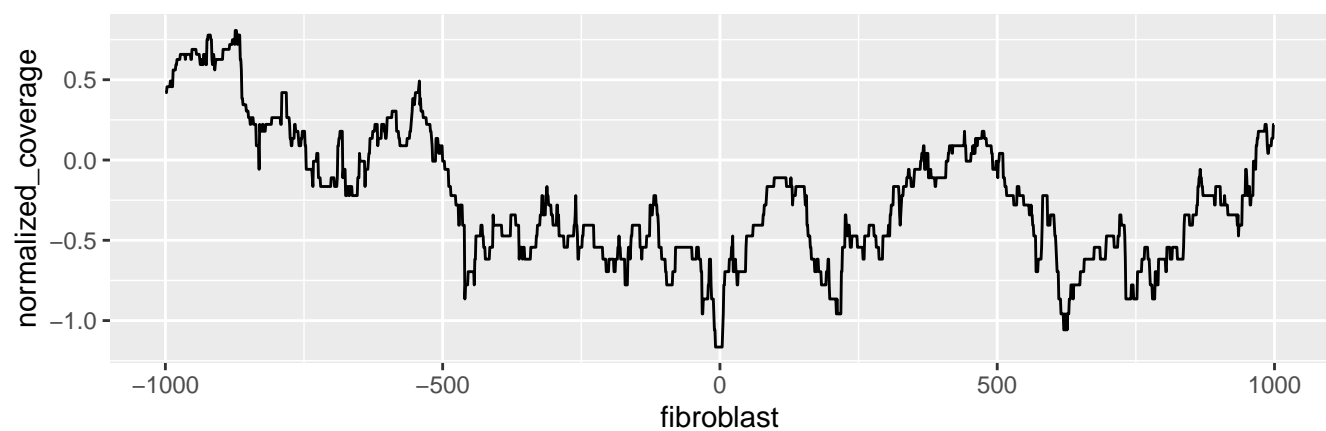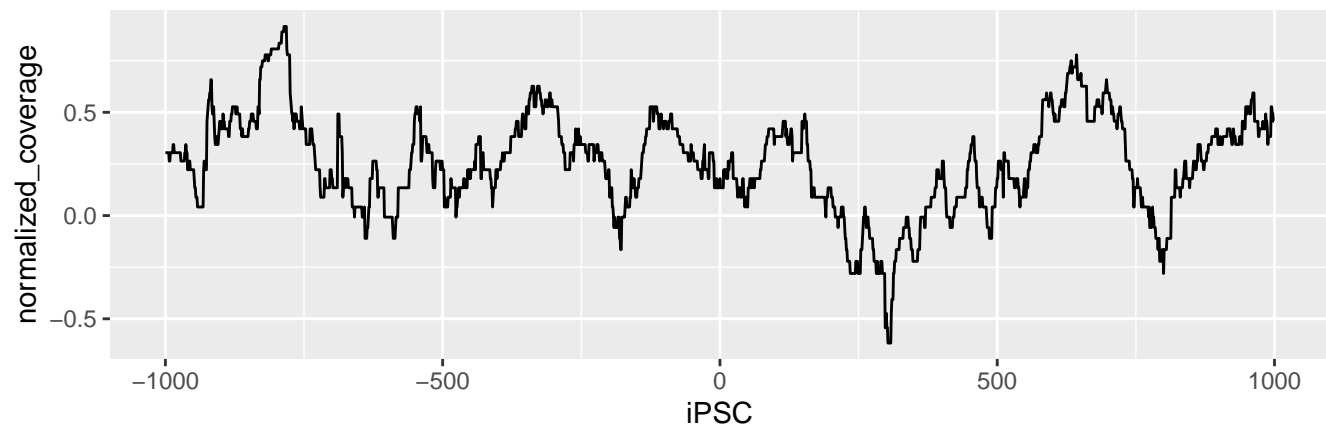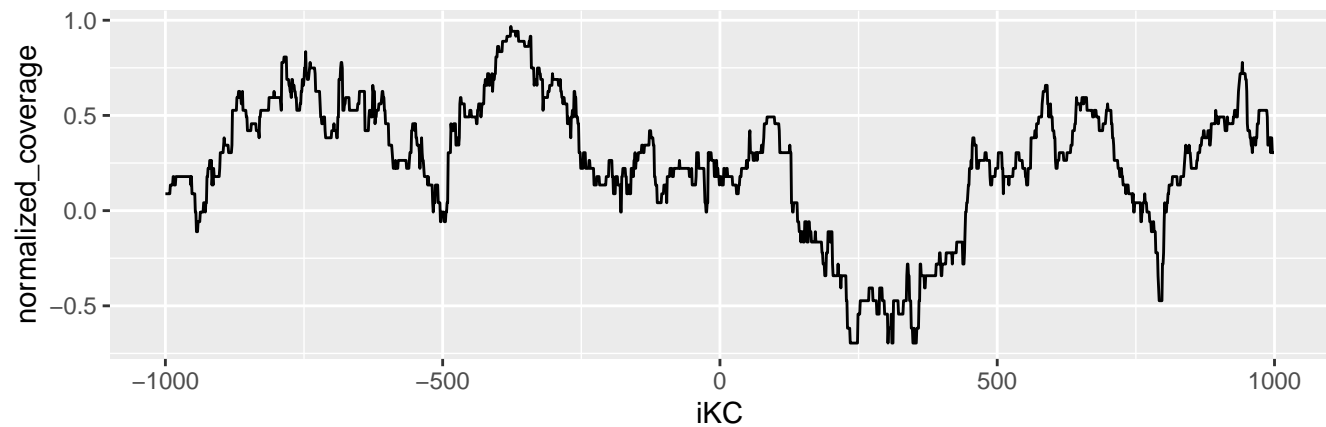

Supplement: Supplementary file 6 — Supplementary Data 3 [file 41467_2024_49400_MOESM6_ESM.zip › Supplementary Data 3/57_offtarget_sites/CO1-131_2KB/patient3_1.chrX_104827480_104827502.2KB.pdf]

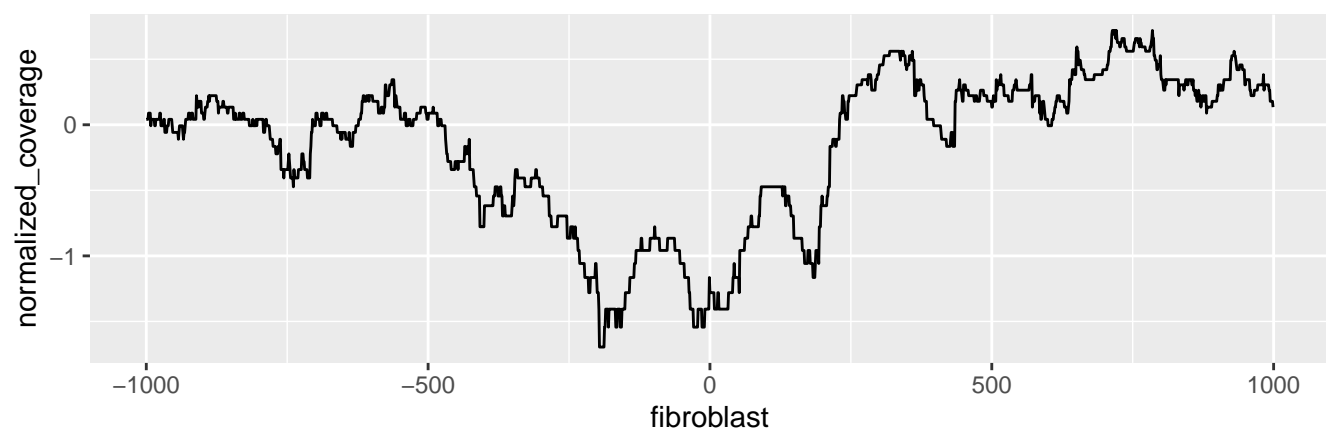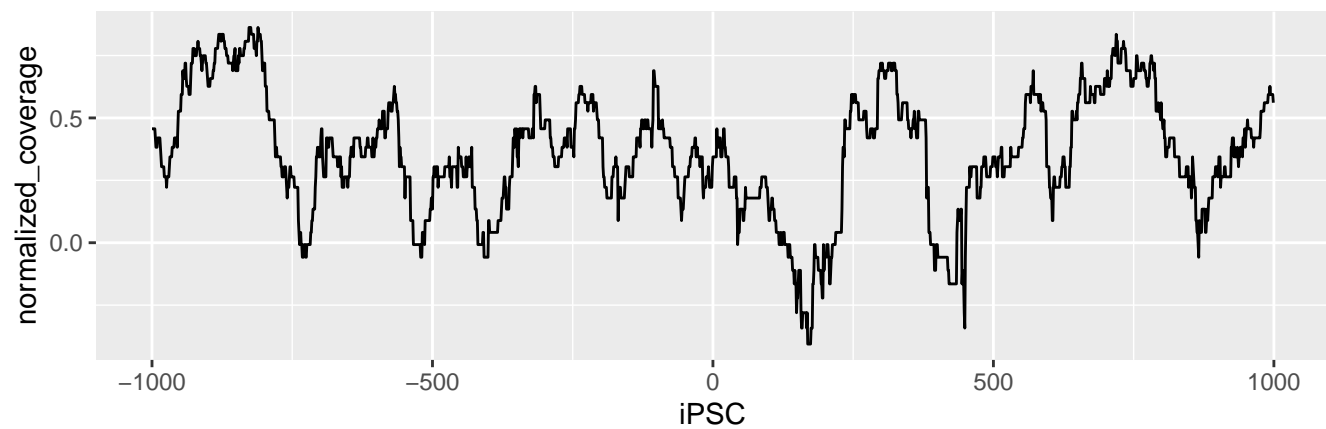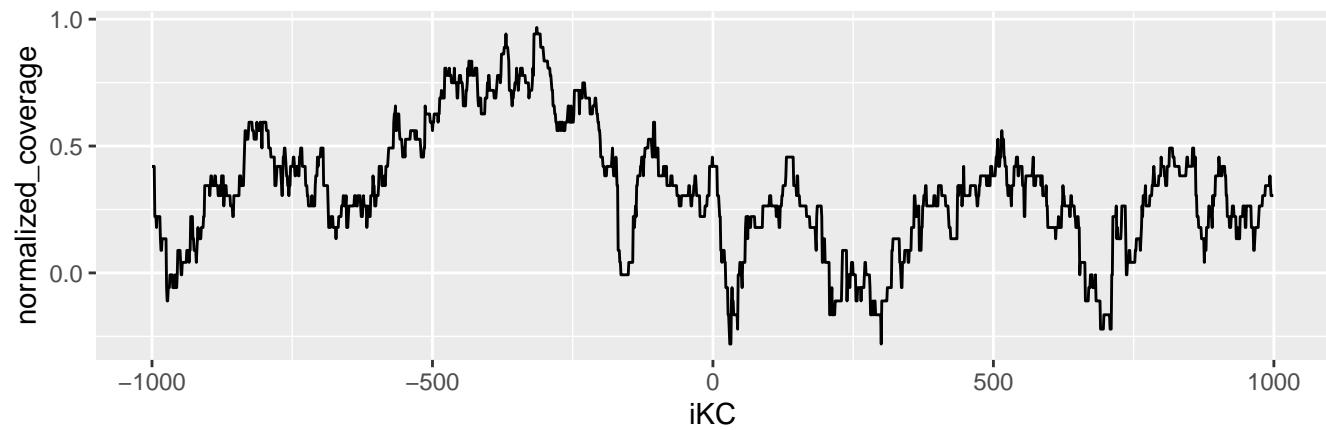

Supplement: Supplementary file 6 — Supplementary Data 3 [file 41467_2024_49400_MOESM6_ESM.zip › Supplementary Data 3/57_offtarget_sites/CO1-131_2KB/patient3_1.chrX_22868452_22868474.2KB.pdf]

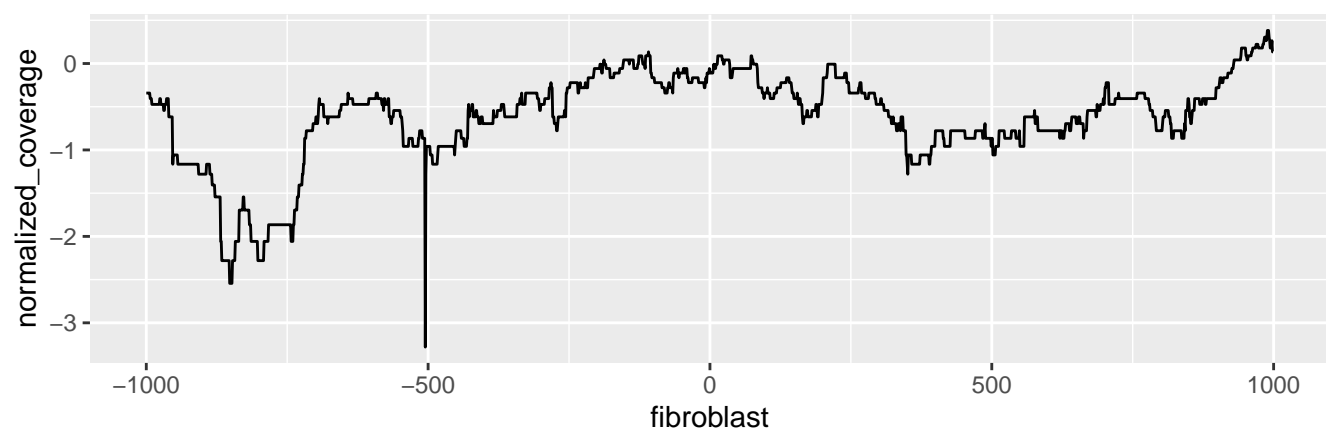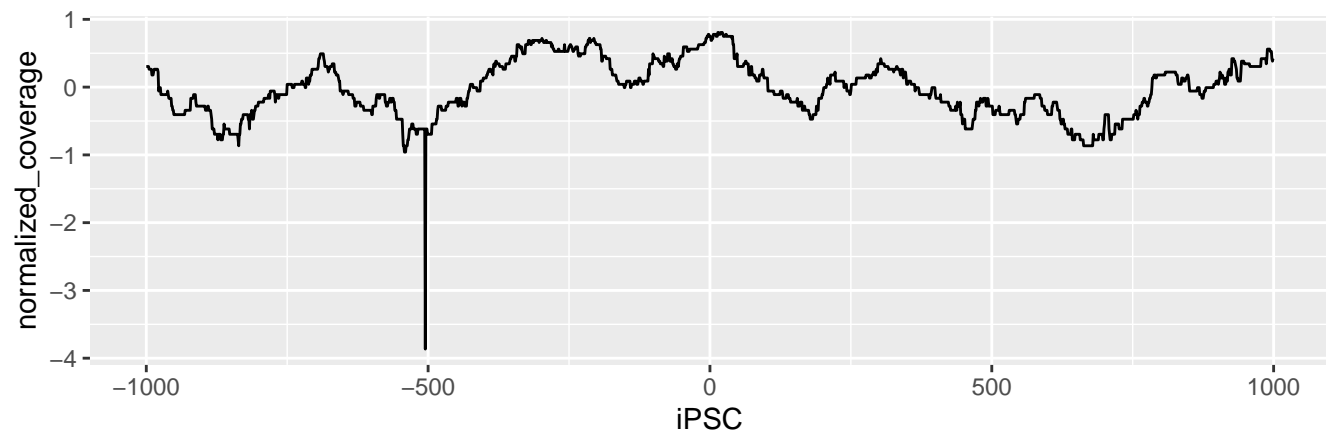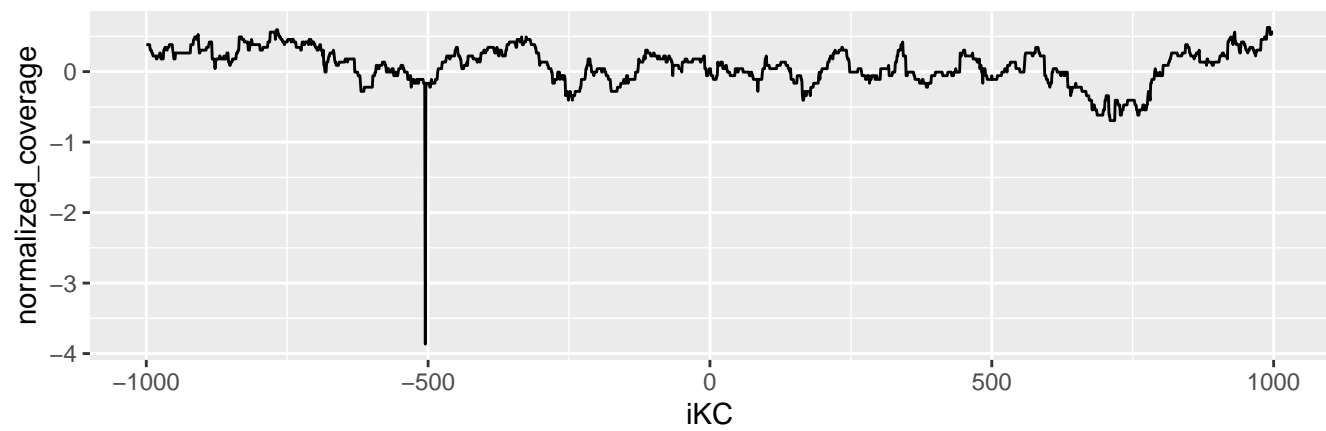

Supplement: Supplementary file 6 — Supplementary Data 3 [file 41467_2024_49400_MOESM6_ESM.zip › Supplementary Data 3/57_offtarget_sites/CO1-131_2KB/patient3_1.chrX_34794493_34794515.2KB.pdf]

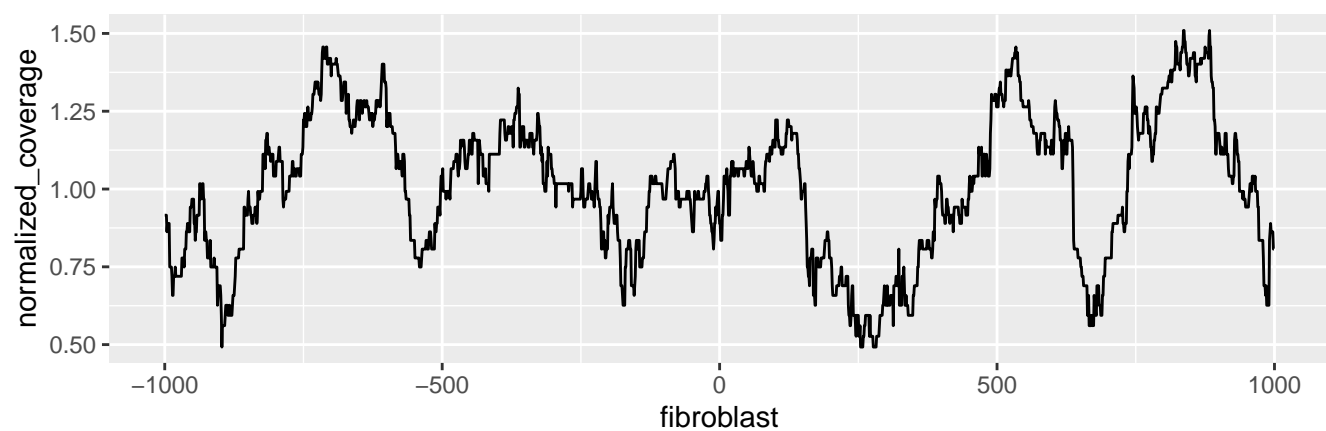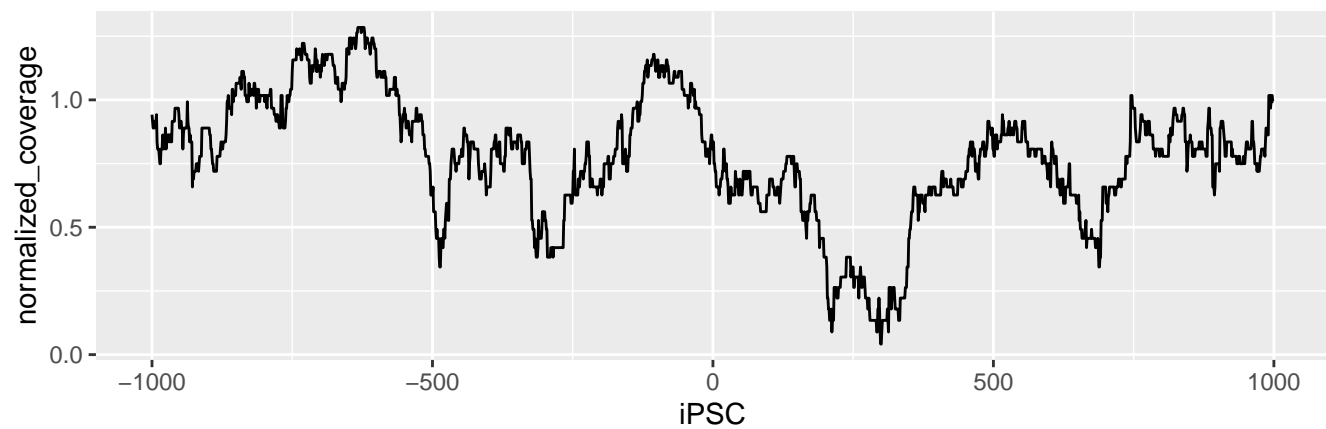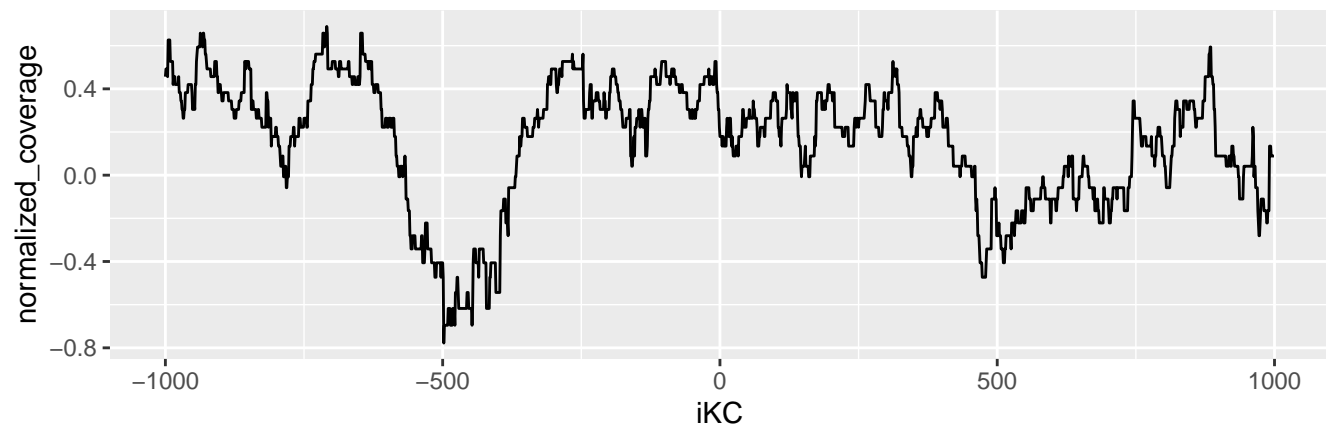

Supplement: Supplementary file 6 — Supplementary Data 3 [file 41467_2024_49400_MOESM6_ESM.zip › Supplementary Data 3/57_offtarget_sites/CO1-131_2KB/patient3_1.chrX_47178057_47178079.2KB.pdf]

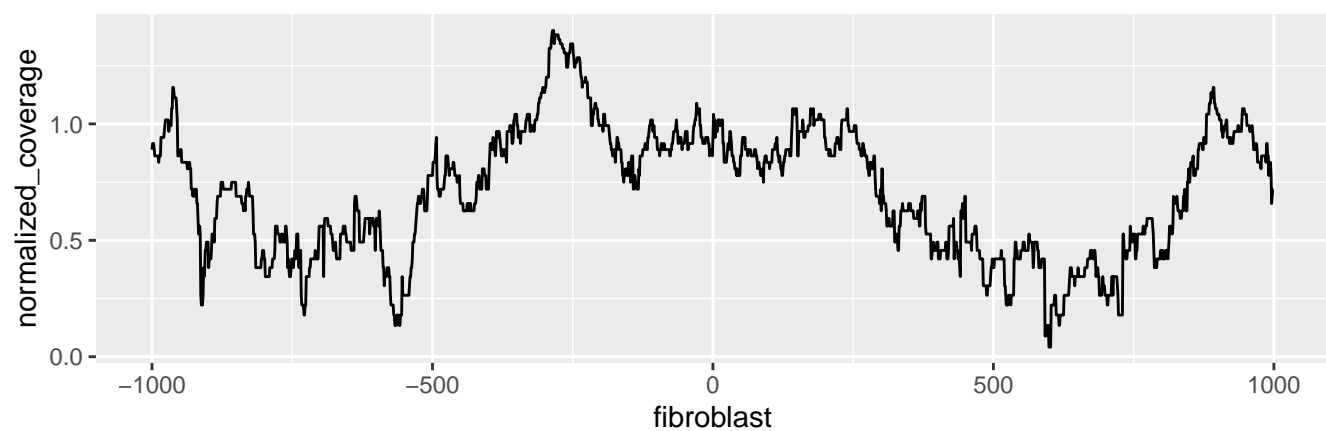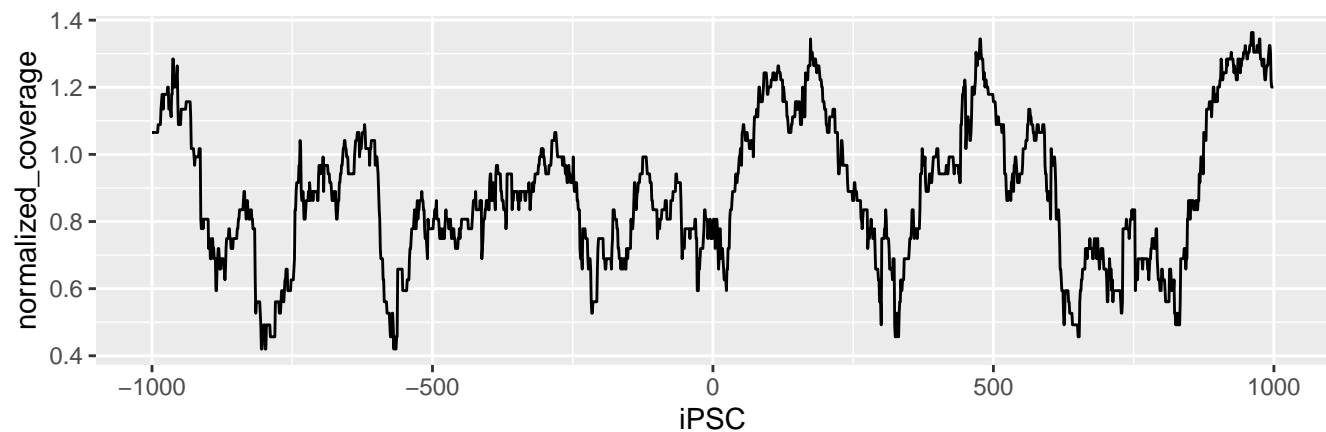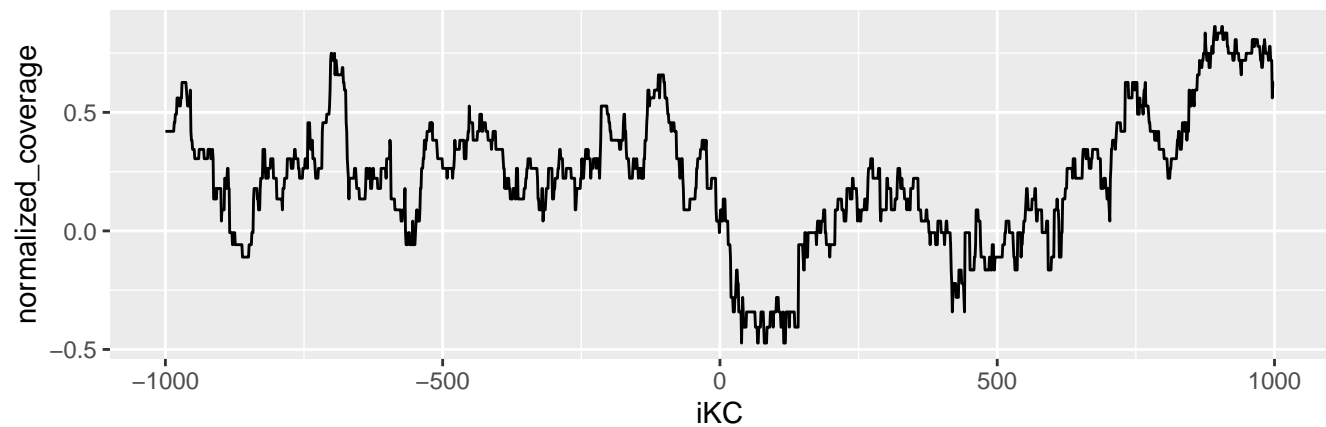

Supplement: Supplementary file 6 — Supplementary Data 3 [file 41467_2024_49400_MOESM6_ESM.zip › Supplementary Data 3/57_offtarget_sites/CO1-131_2KB/patient3_1.chrX_69297624_69297646.2KB.pdf]

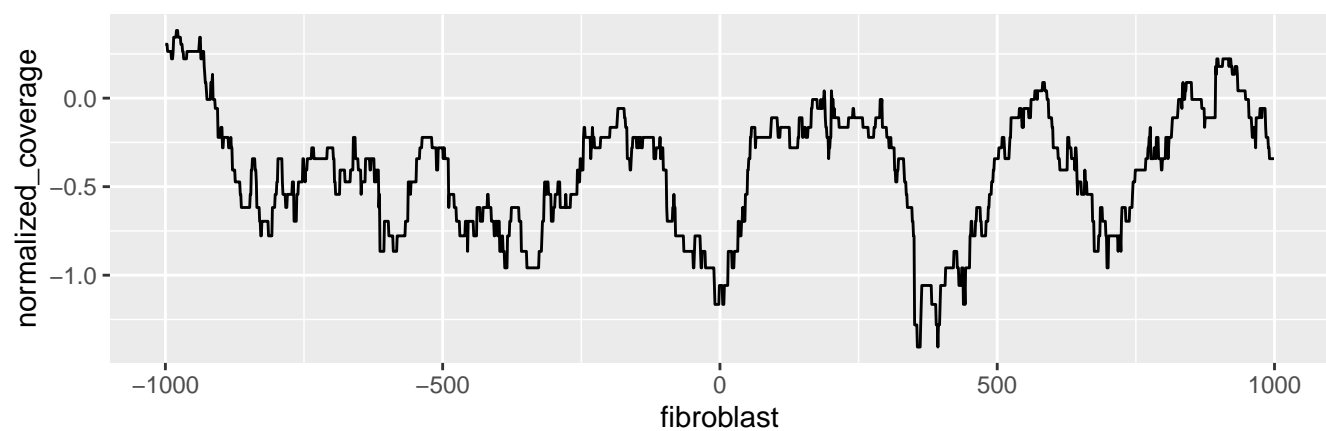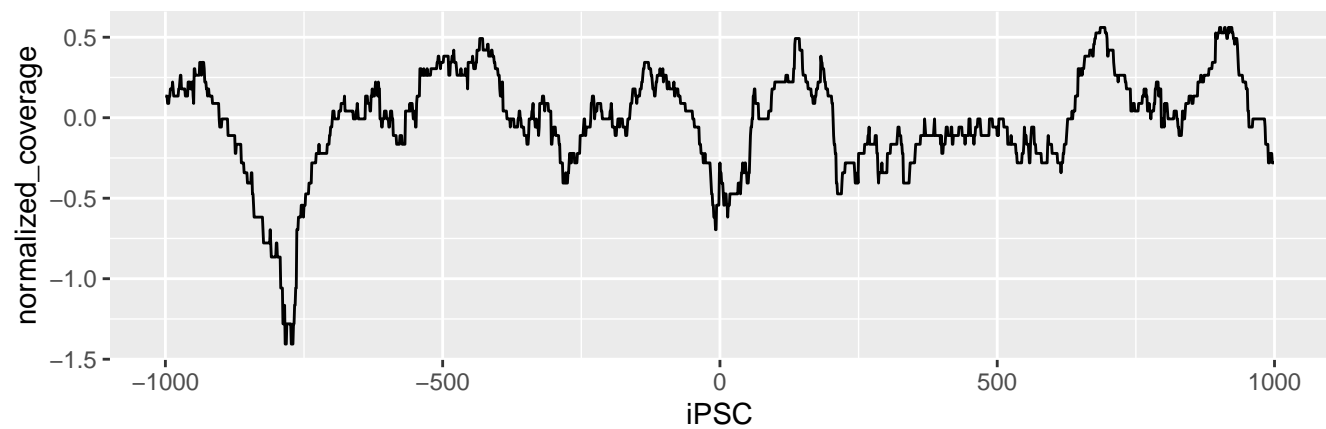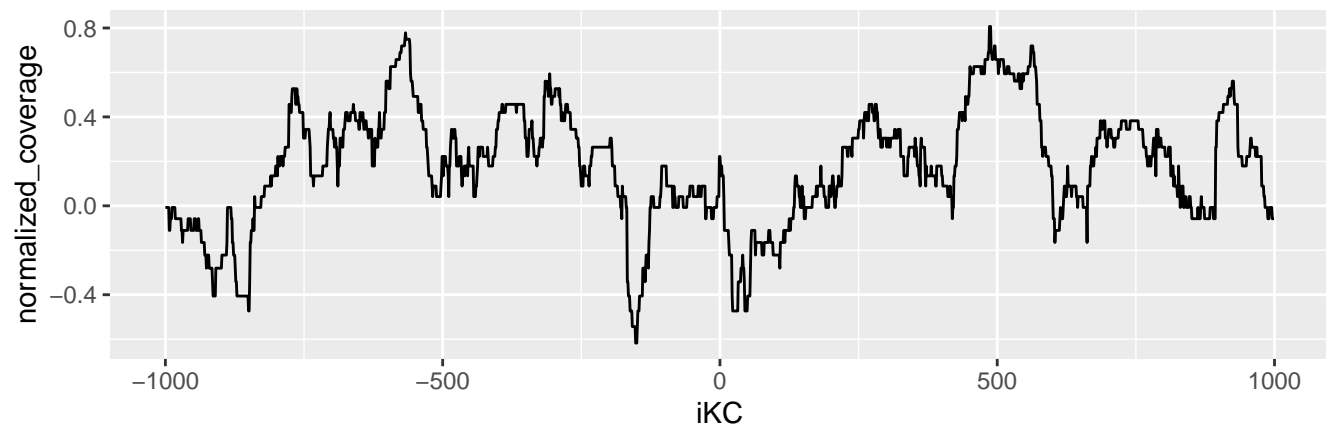

Supplement: Supplementary file 6 — Supplementary Data 3 [file 41467_2024_49400_MOESM6_ESM.zip › Supplementary Data 3/57_offtarget_sites/CO1-131_2KB/patient3_1.chrX_88696315_88696337.2KB.pdf]

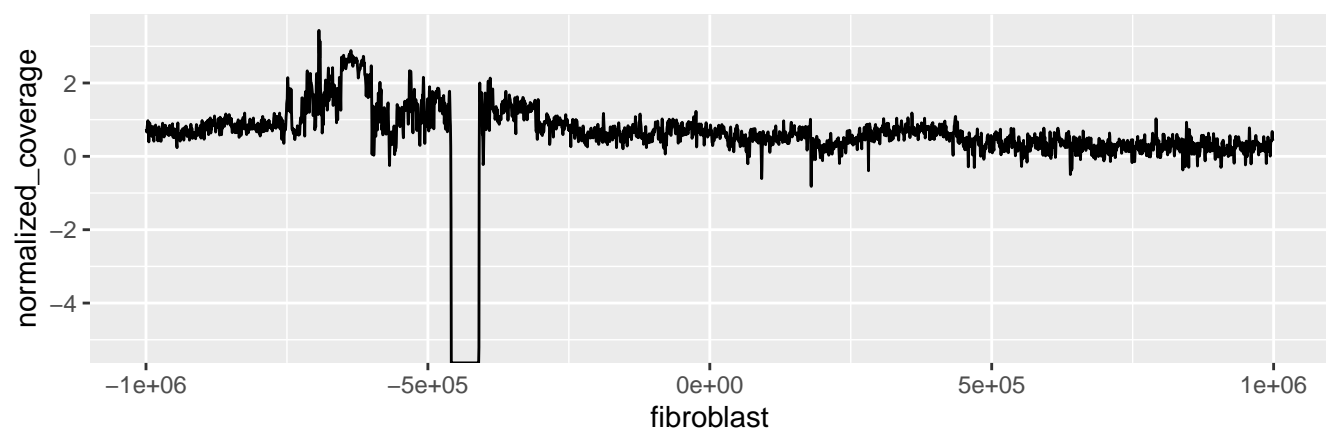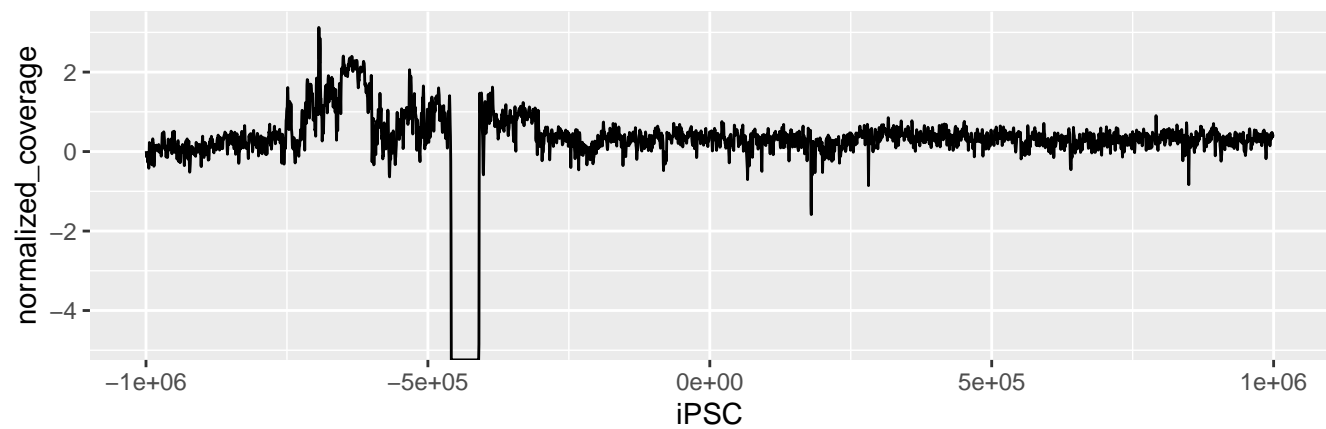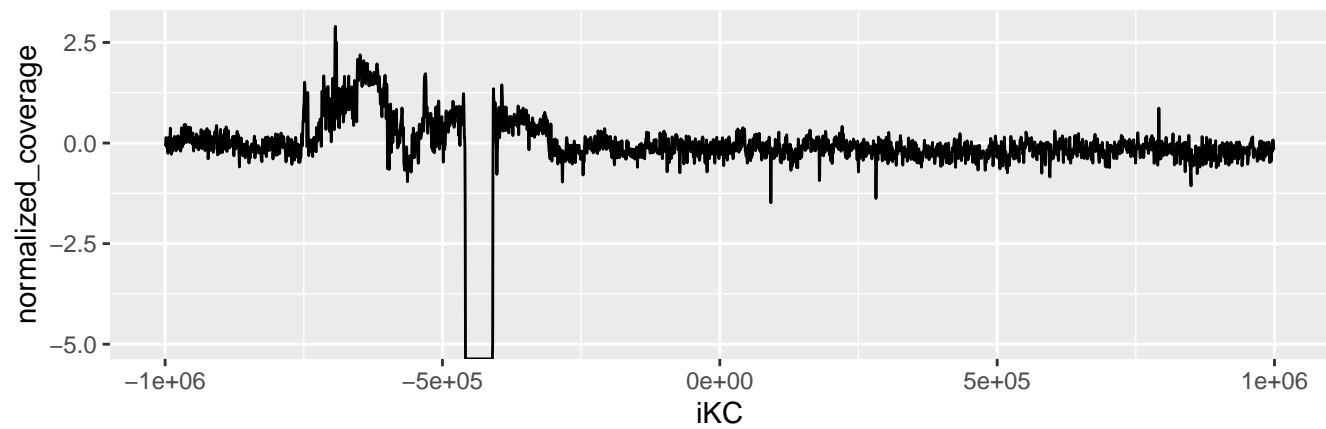

Supplement: Supplementary file 6 — Supplementary Data 3 [file 41467_2024_49400_MOESM6_ESM.zip › Supplementary Data 3/57_offtarget_sites/CO1-131_2MB/patient3_1.chr1_17258328_17258350.2MB.pdf]

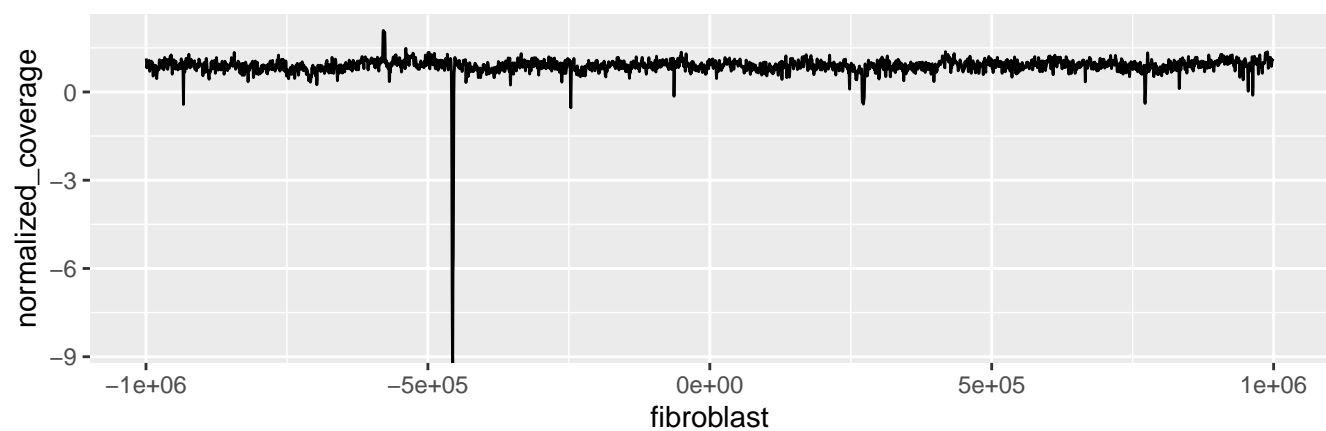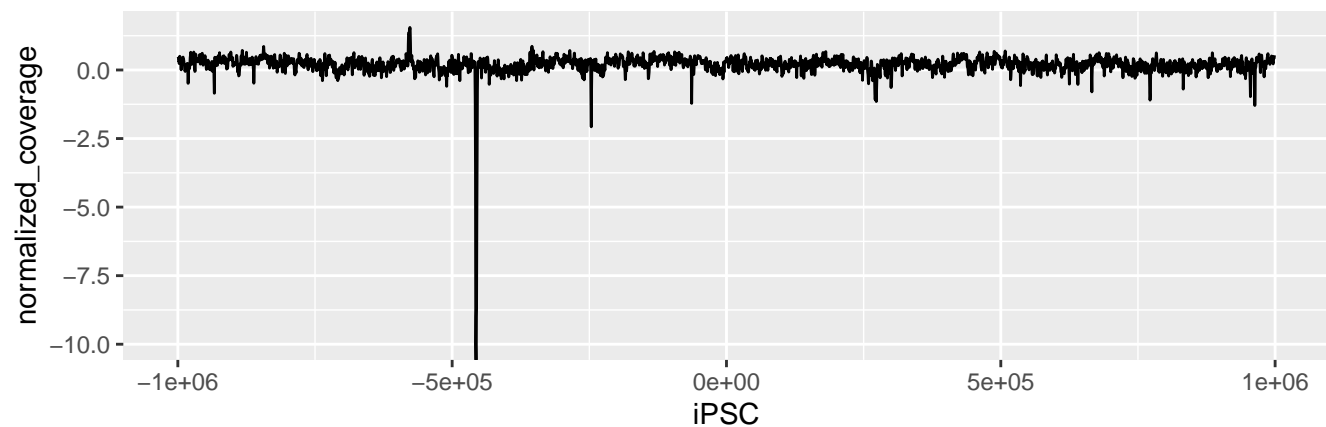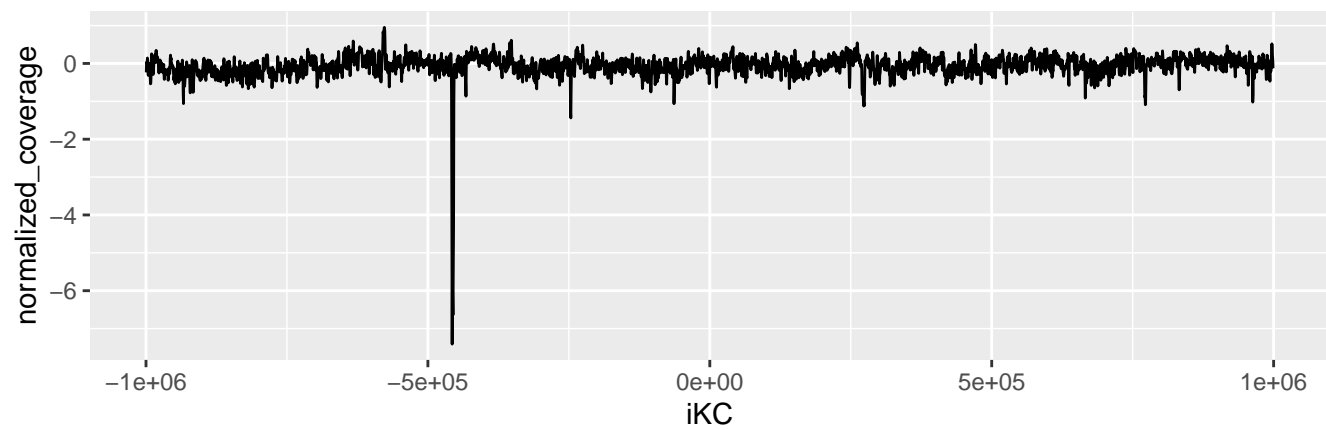

Supplement: Supplementary file 6 — Supplementary Data 3 [file 41467_2024_49400_MOESM6_ESM.zip › Supplementary Data 3/57_offtarget_sites/CO1-131_2MB/patient3_1.chr1_25863728_25863750.2MB.pdf]

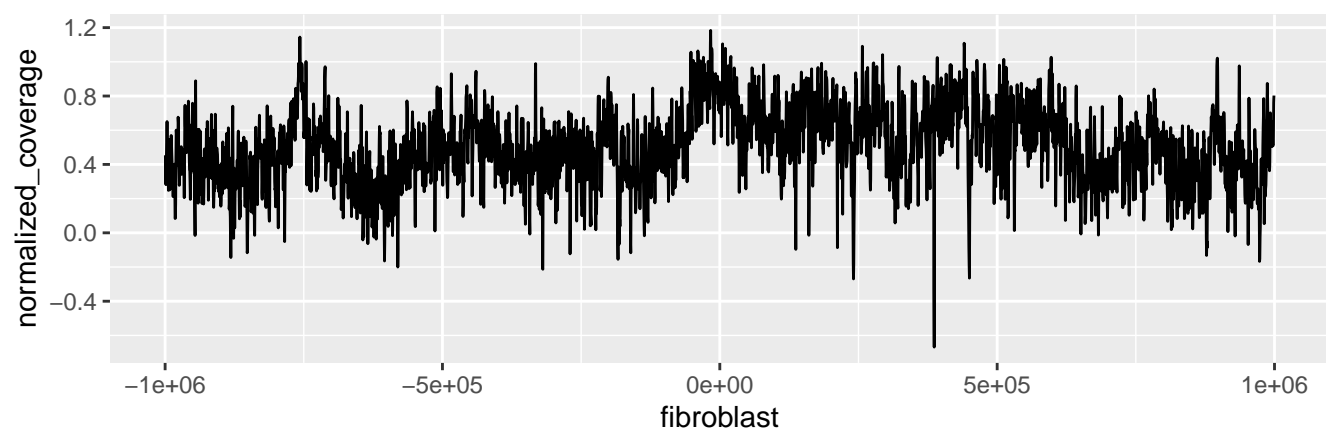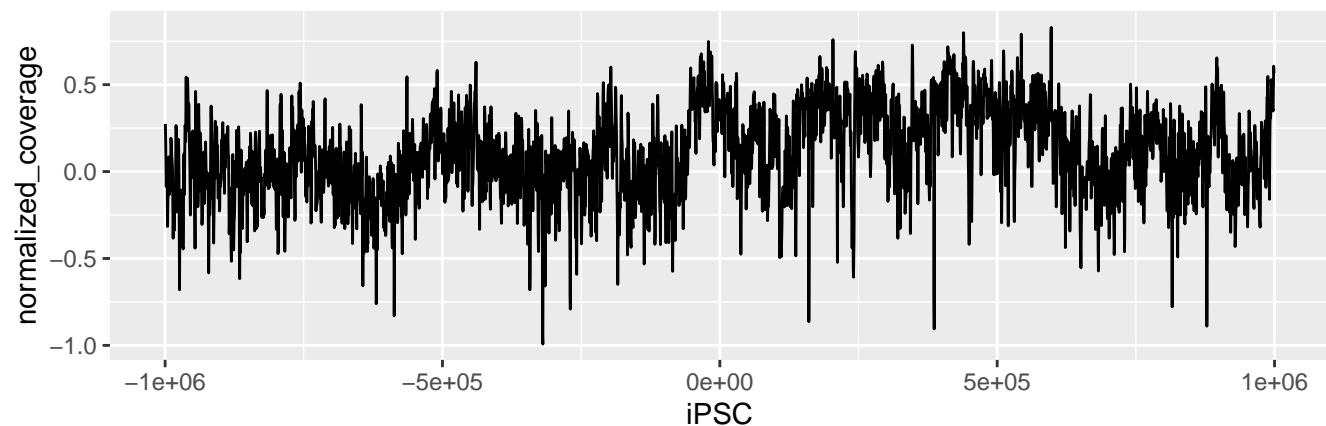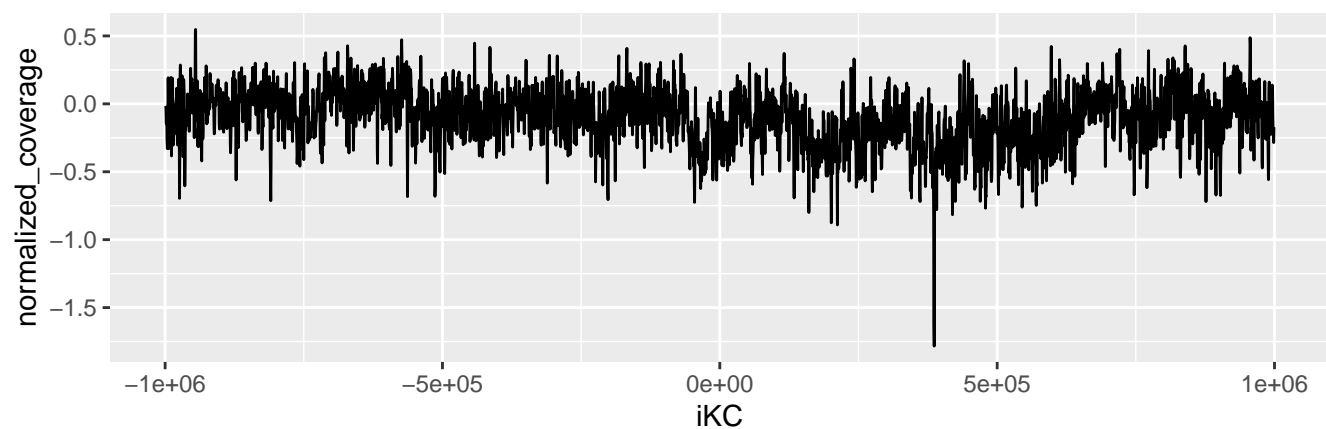

Supplement: Supplementary file 6 — Supplementary Data 3 [file 41467_2024_49400_MOESM6_ESM.zip › Supplementary Data 3/57_offtarget_sites/CO1-131_2MB/patient3_1.chr1_53116163_53116185.2MB.pdf]

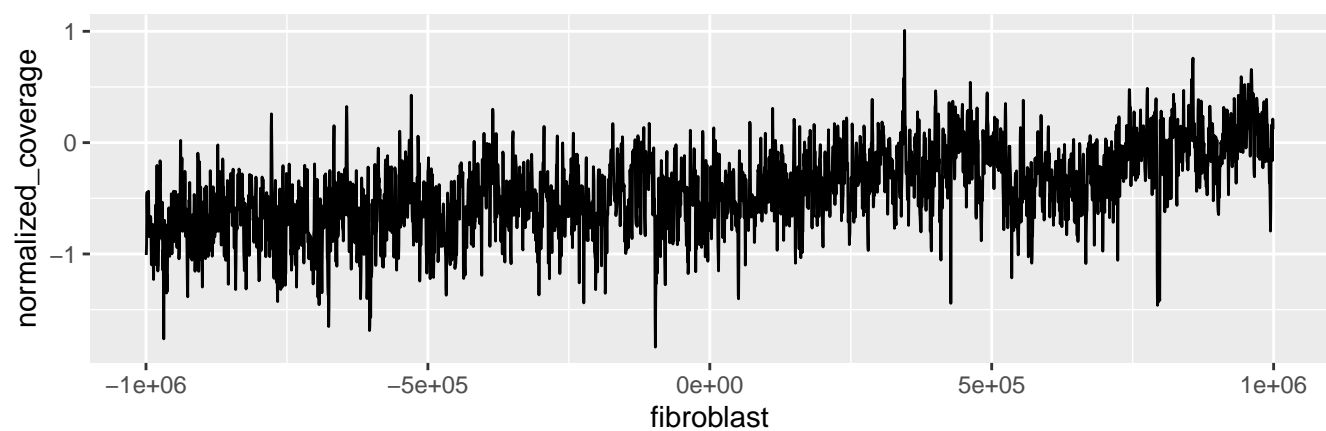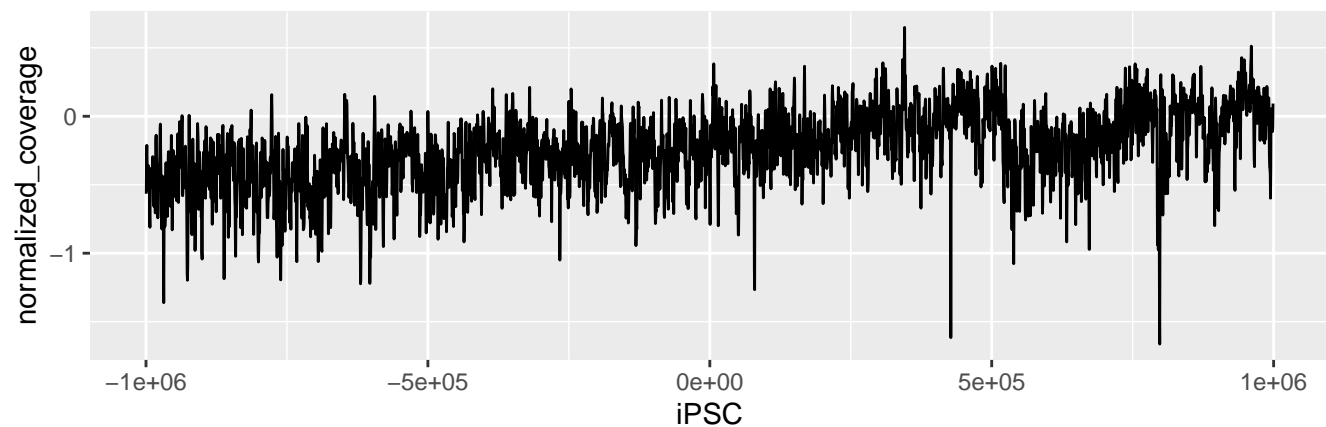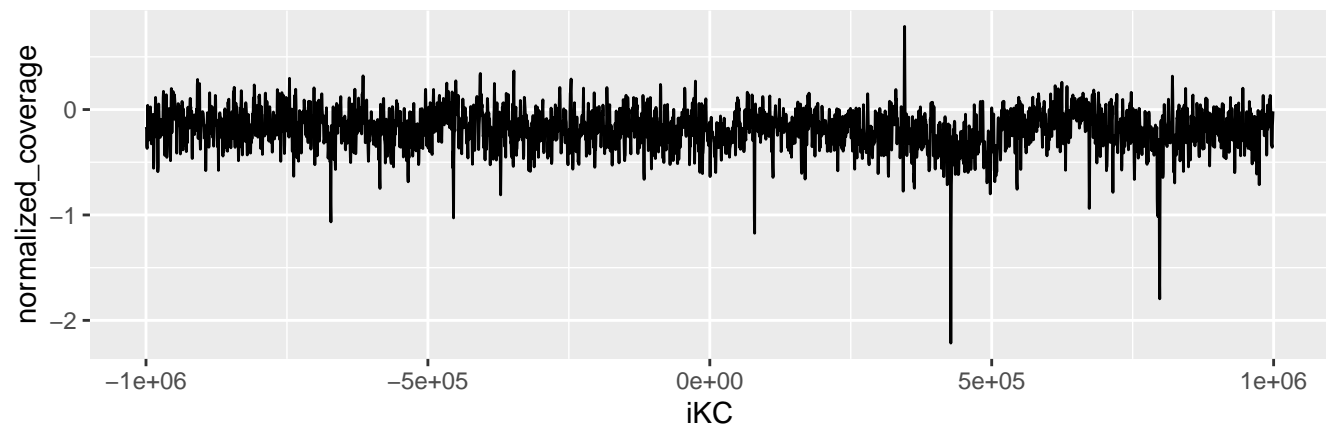

Supplement: Supplementary file 6 — Supplementary Data 3 [file 41467_2024_49400_MOESM6_ESM.zip › Supplementary Data 3/57_offtarget_sites/CO1-131_2MB/patient3_1.chr10_10938785_10938807.2MB.pdf]

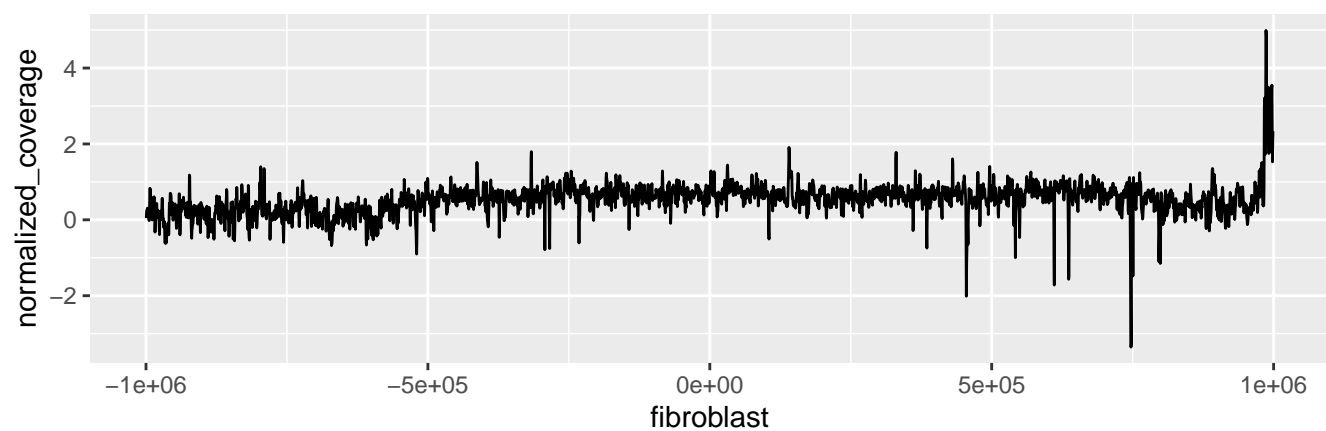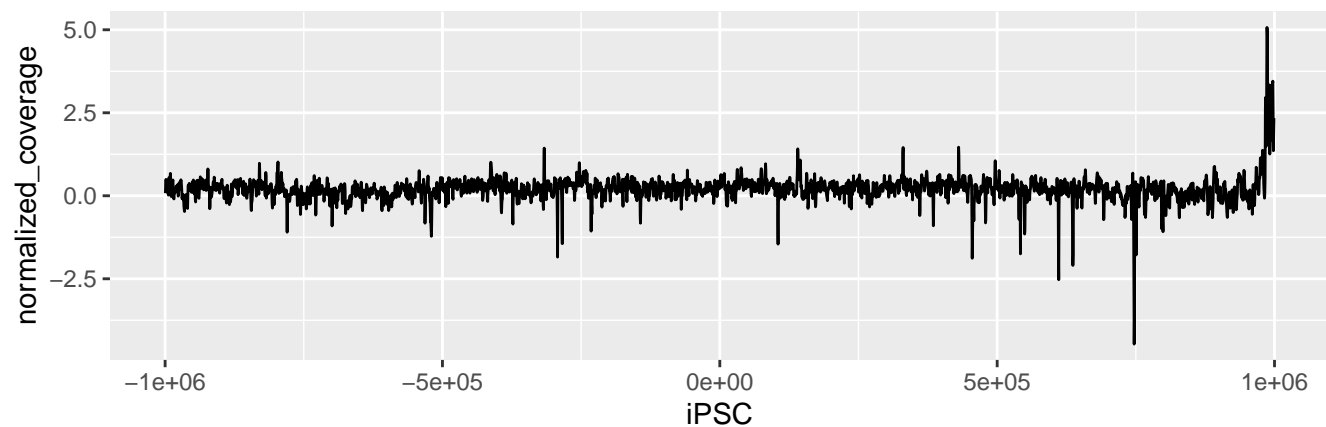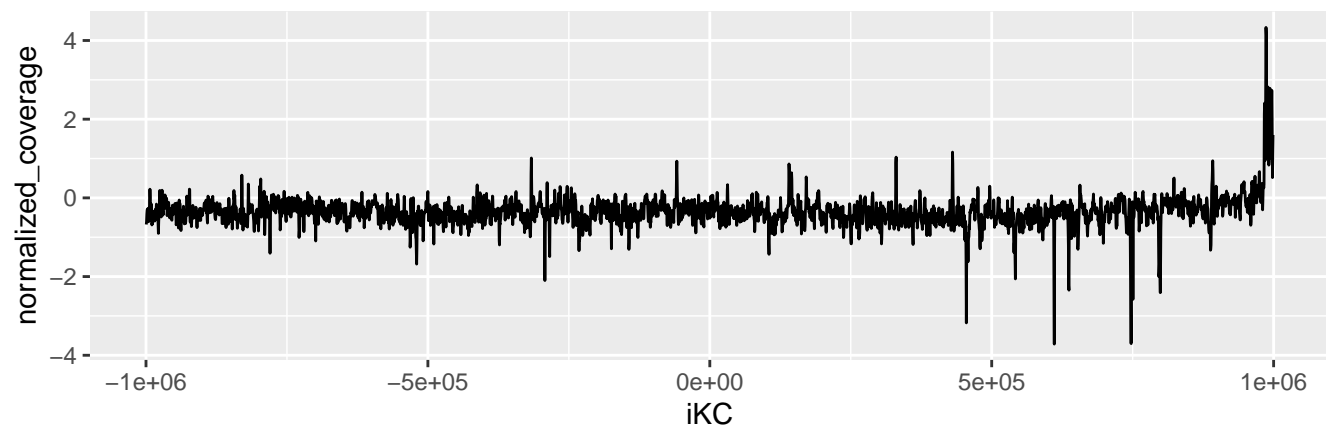

Supplement: Supplementary file 6 — Supplementary Data 3 [file 41467_2024_49400_MOESM6_ESM.zip › Supplementary Data 3/57_offtarget_sites/CO1-131_2MB/patient3_1.chr10_132681079_132681101.2MB.pdf]

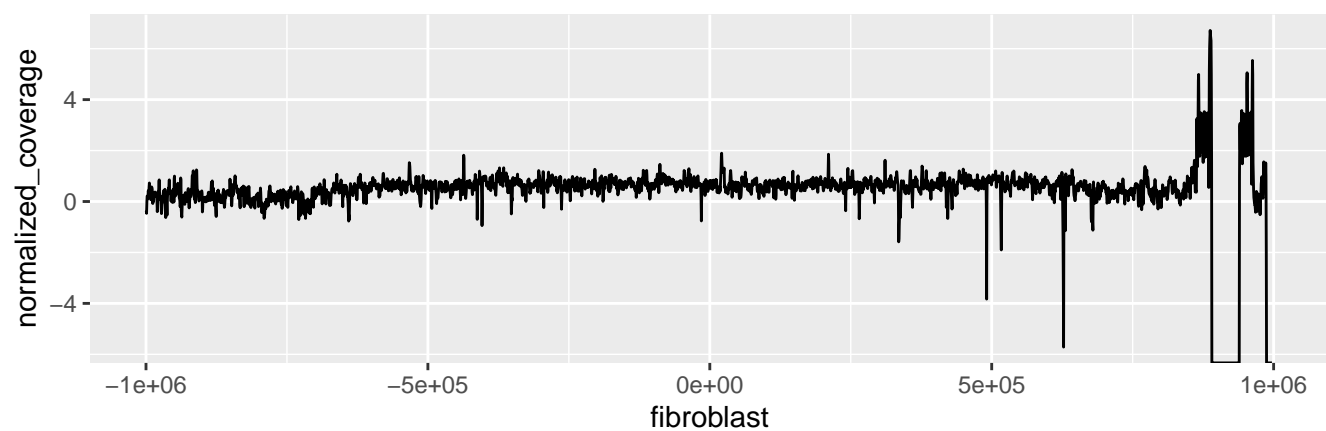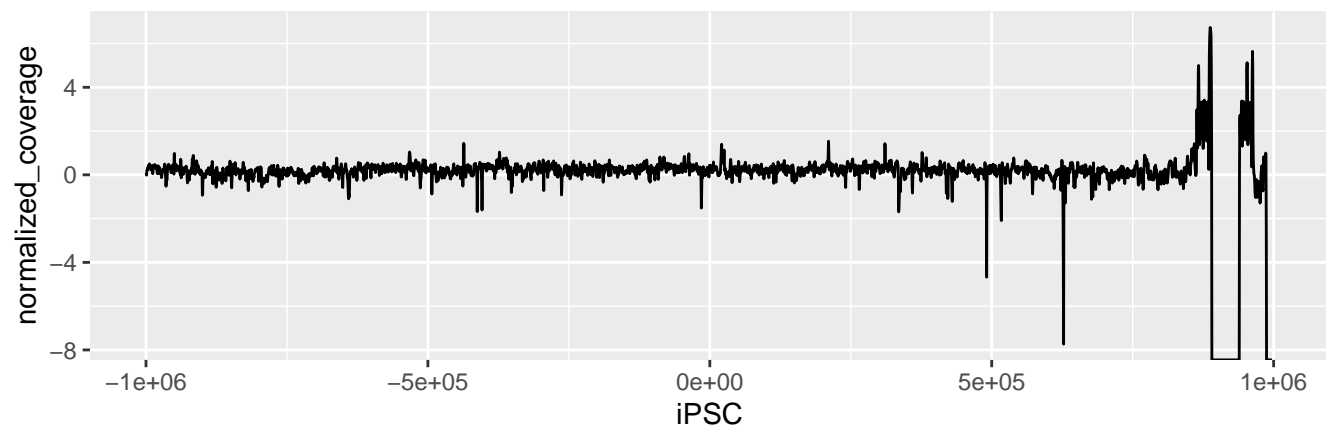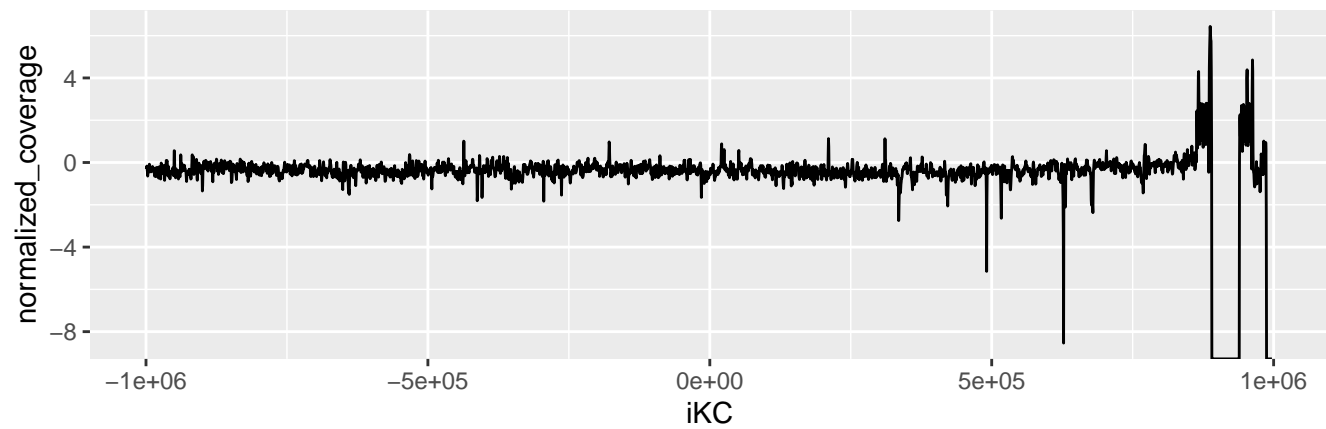

Supplement: Supplementary file 6 — Supplementary Data 3 [file 41467_2024_49400_MOESM6_ESM.zip › Supplementary Data 3/57_offtarget_sites/CO1-131_2MB/patient3_1.chr10_132800880_132800902.2MB.pdf]

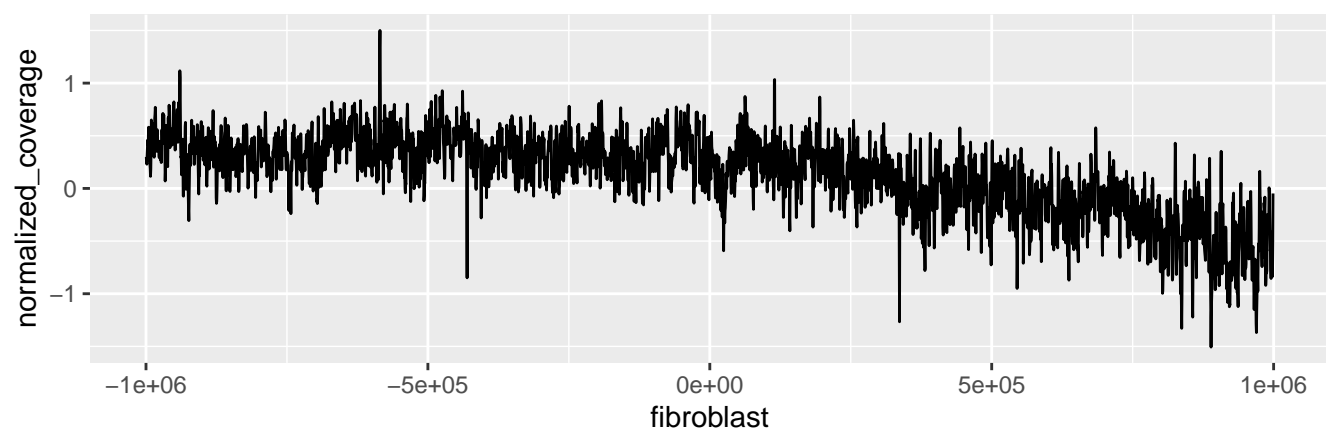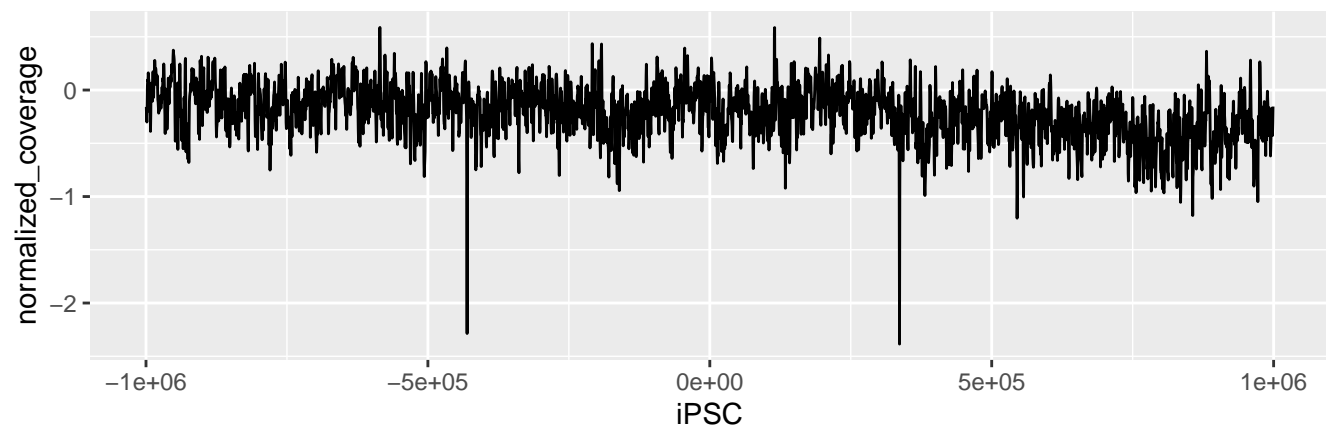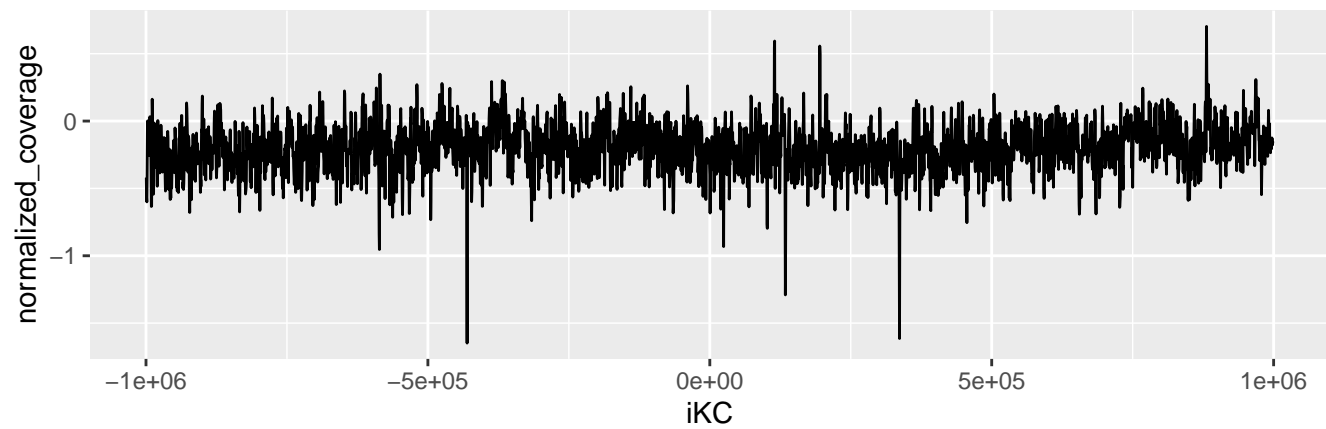

Supplement: Supplementary file 6 — Supplementary Data 3 [file 41467_2024_49400_MOESM6_ESM.zip › Supplementary Data 3/57_offtarget_sites/CO1-131_2MB/patient3_1.chr10_15034690_15034712.2MB.pdf]

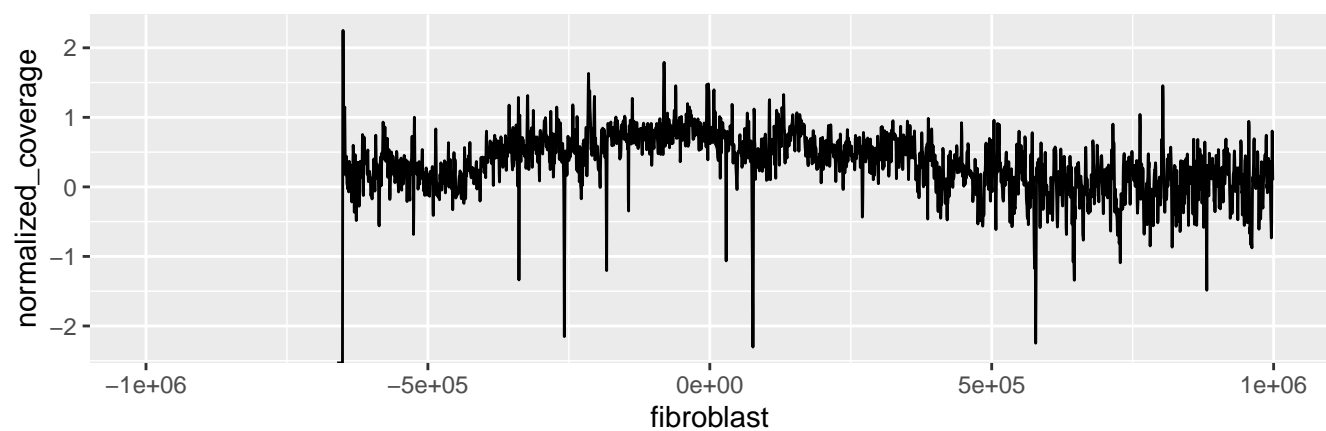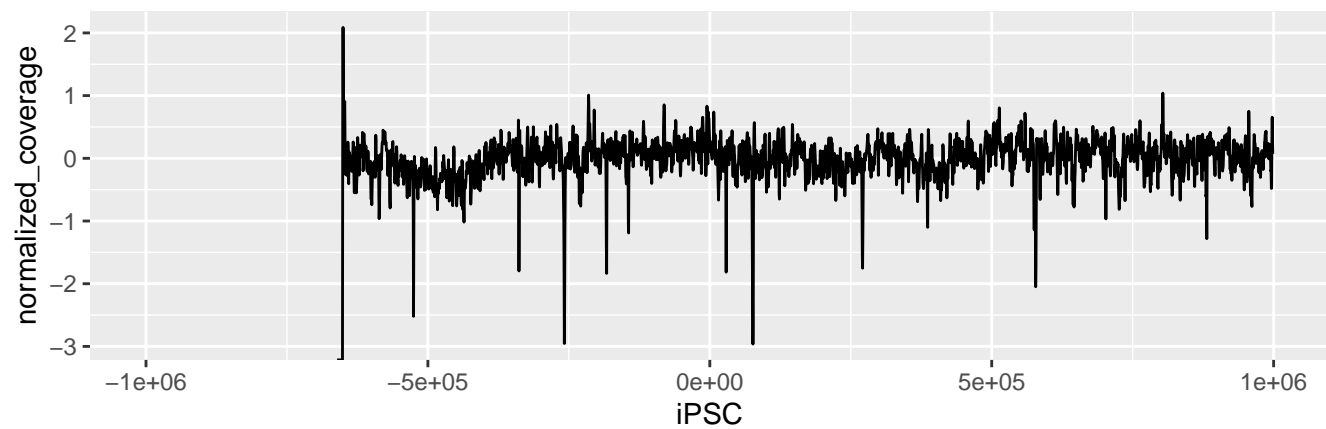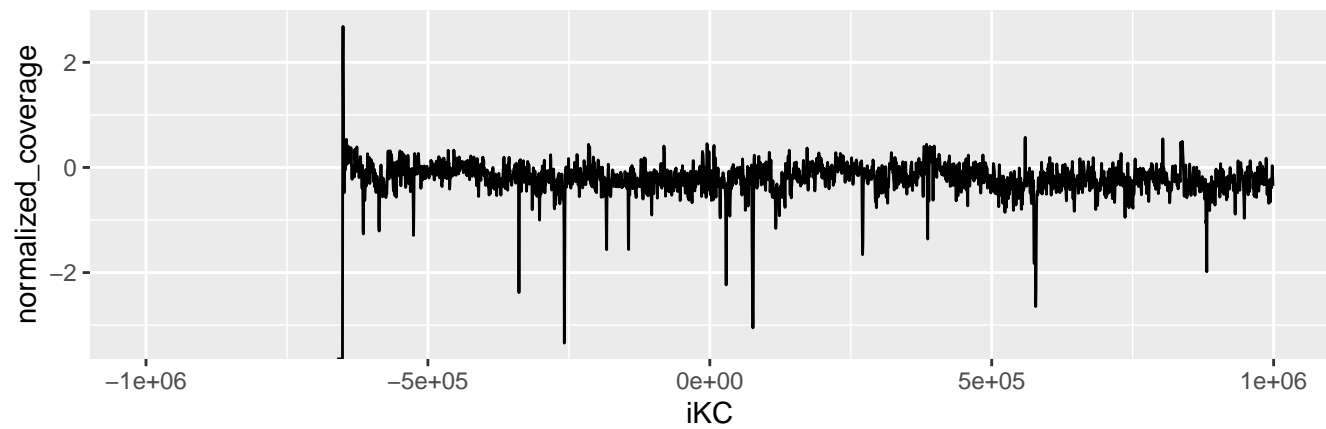

Supplement: Supplementary file 6 — Supplementary Data 3 [file 41467_2024_49400_MOESM6_ESM.zip › Supplementary Data 3/57_offtarget_sites/CO1-131_2MB/patient3_1.chr10_660796_660818.2MB.pdf]

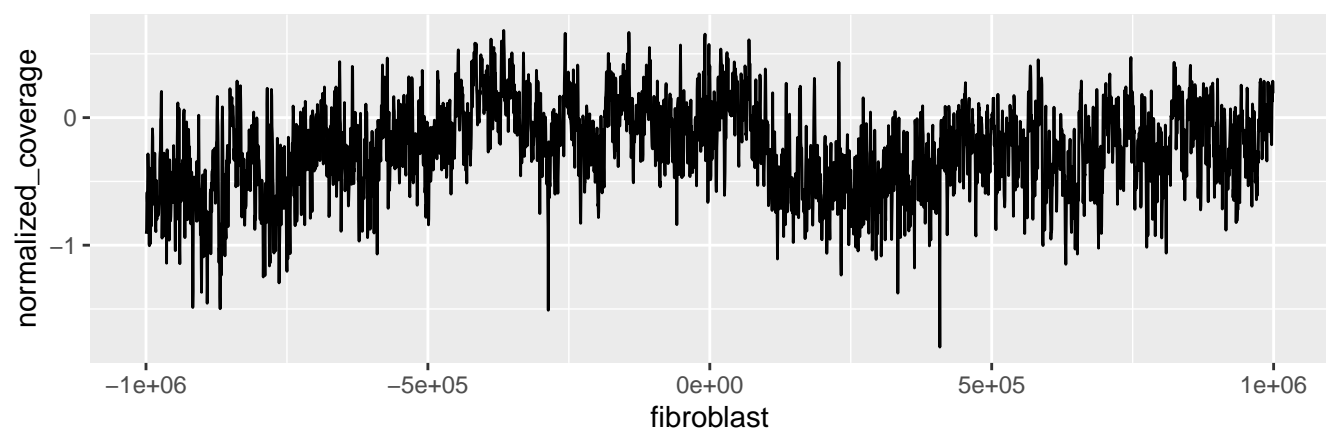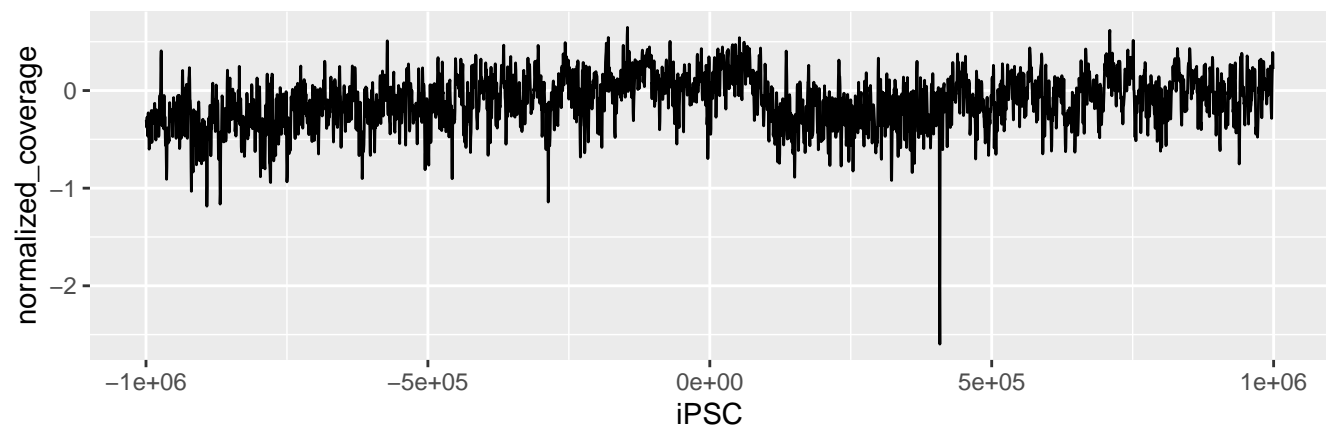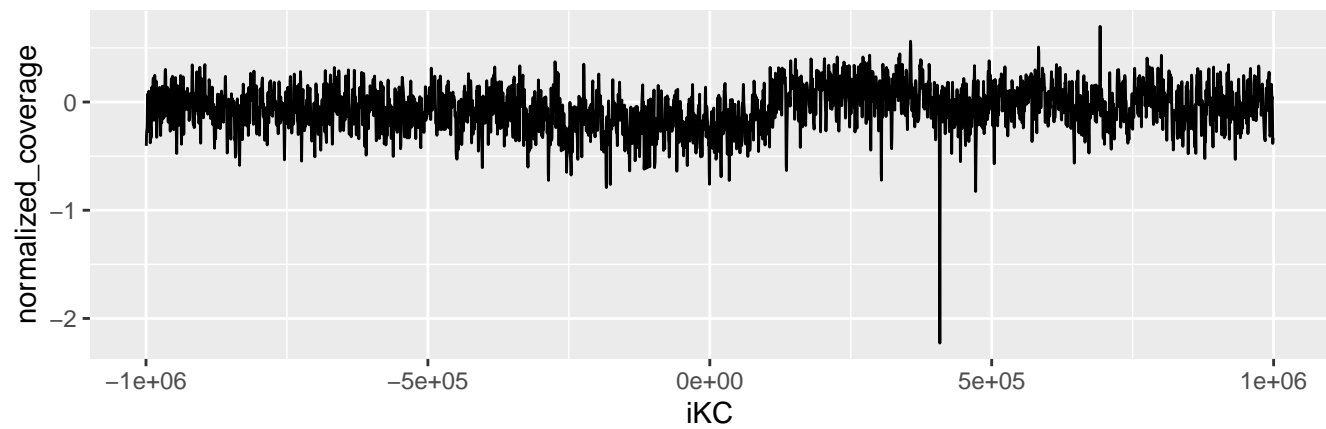

Supplement: Supplementary file 6 — Supplementary Data 3 [file 41467_2024_49400_MOESM6_ESM.zip › Supplementary Data 3/57_offtarget_sites/CO1-131_2MB/patient3_1.chr11_128871452_128871474.2MB.pdf]

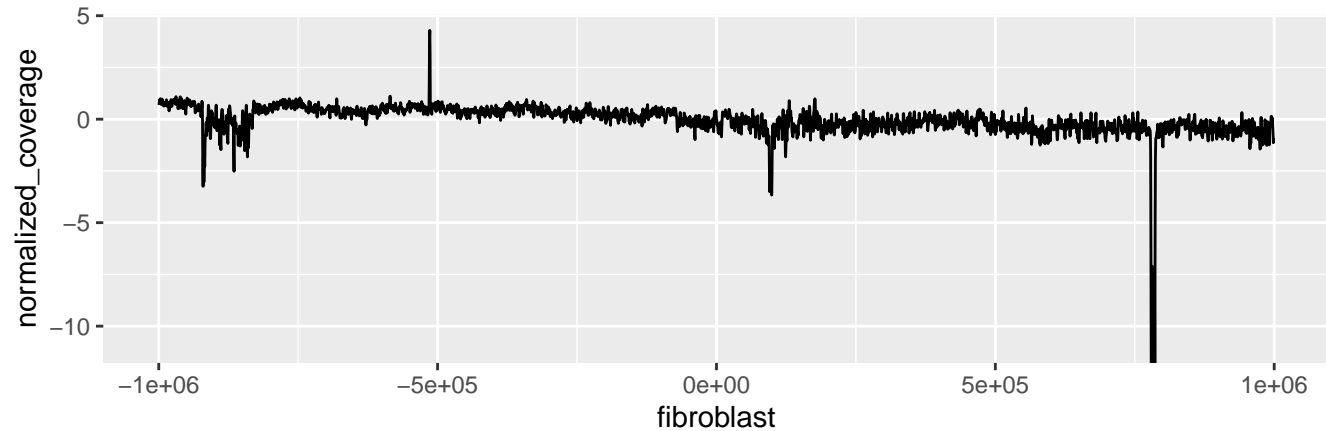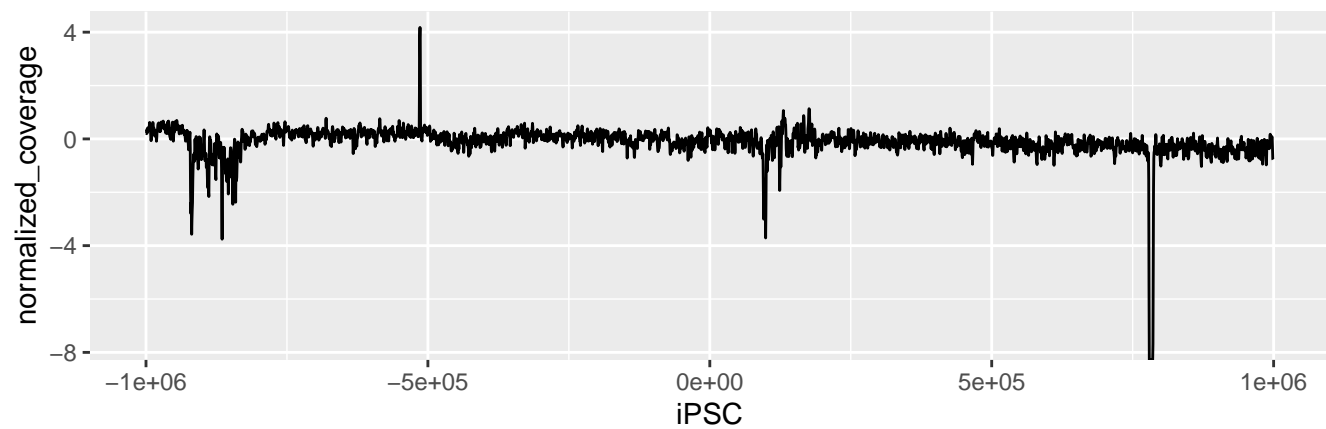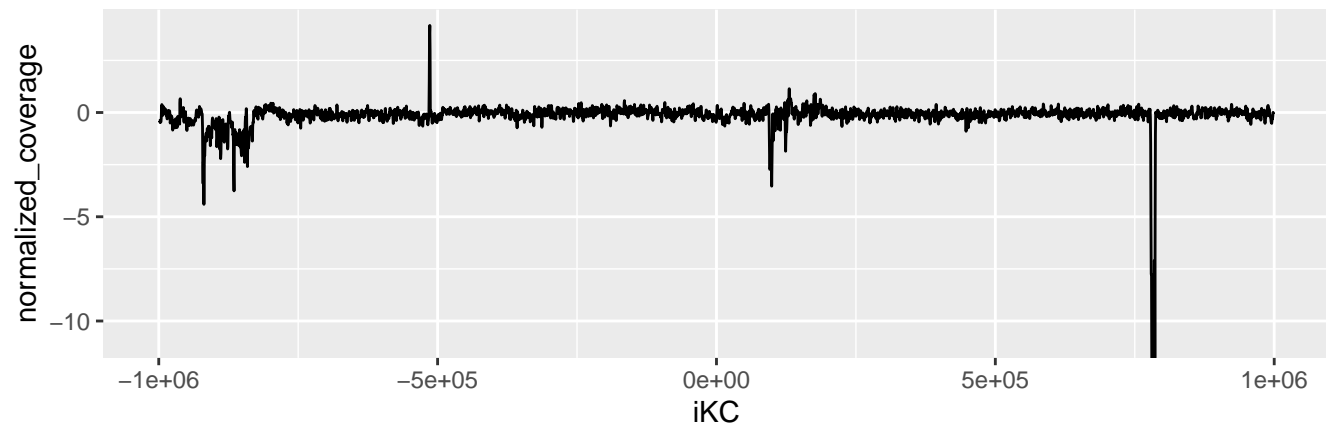

Supplement: Supplementary file 6 — Supplementary Data 3 [file 41467_2024_49400_MOESM6_ESM.zip › Supplementary Data 3/57_offtarget_sites/CO1-131_2MB/patient3_1.chr11_4168526_4168548.2MB.pdf]

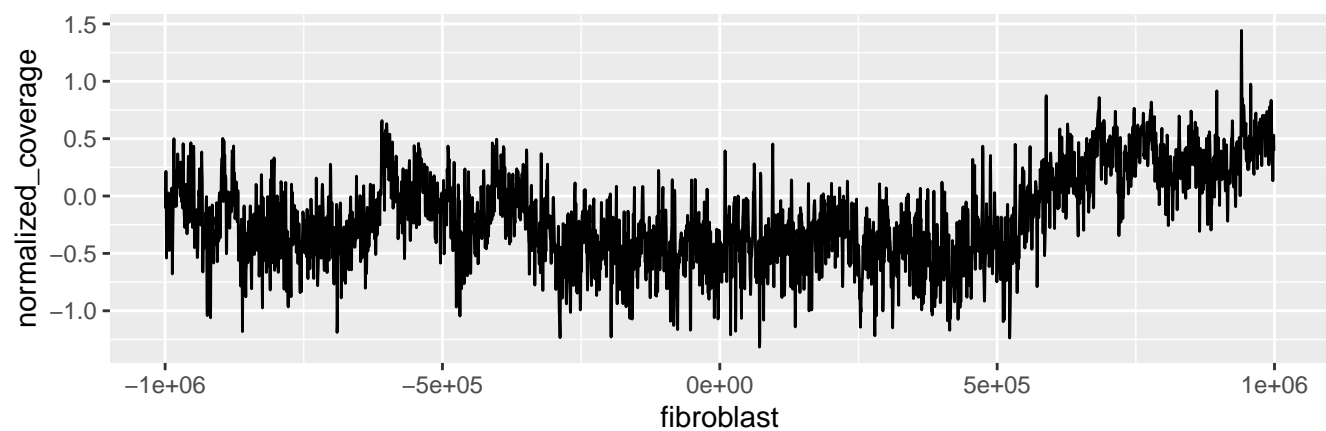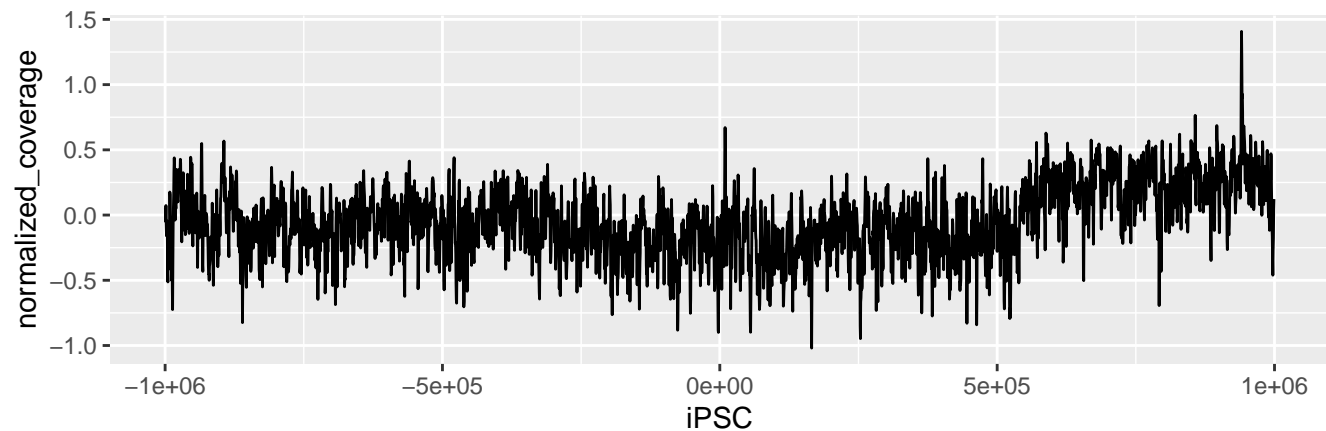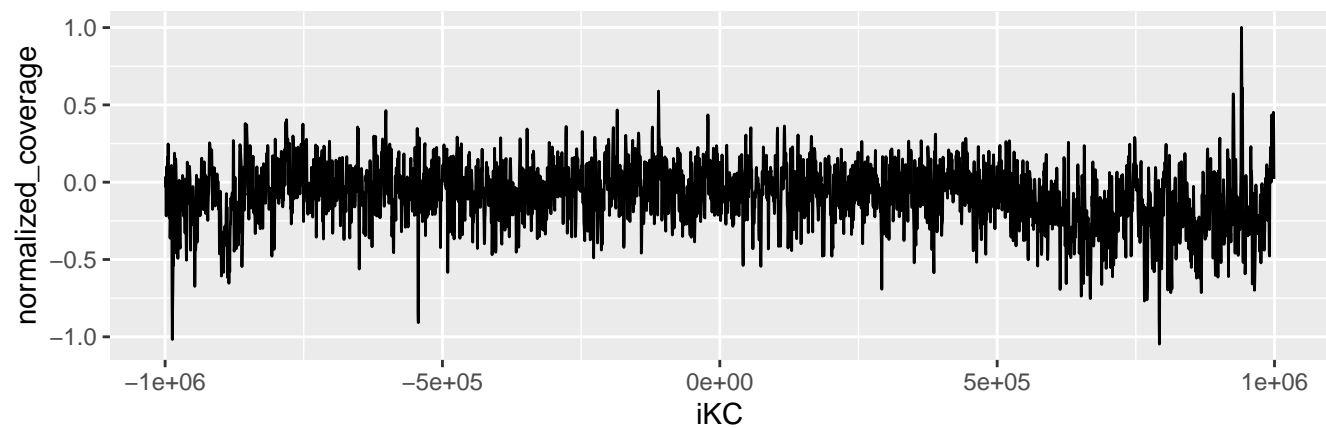

Supplement: Supplementary file 6 — Supplementary Data 3 [file 41467_2024_49400_MOESM6_ESM.zip › Supplementary Data 3/57_offtarget_sites/CO1-131_2MB/patient3_1.chr11_60159330_60159352.2MB.pdf]

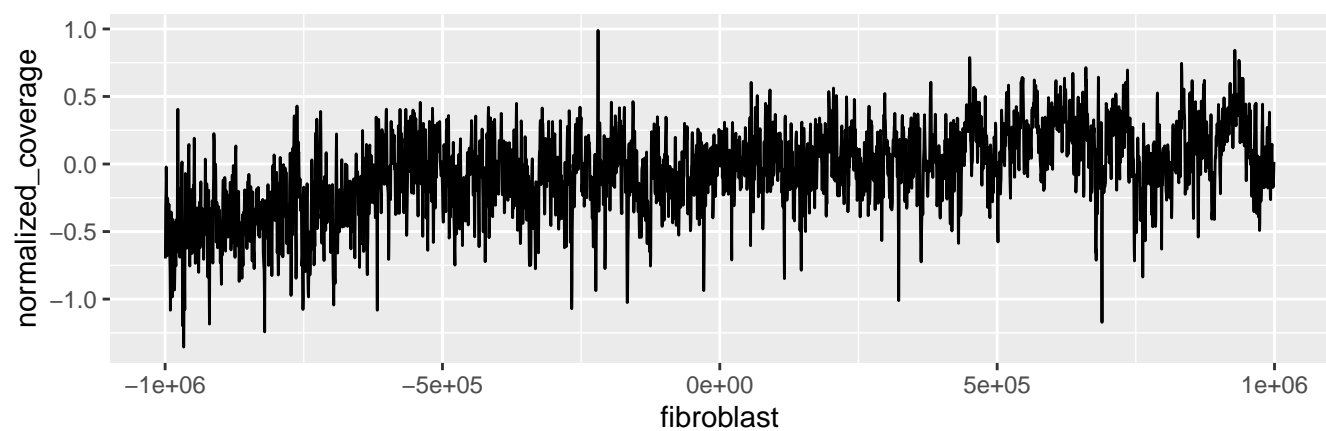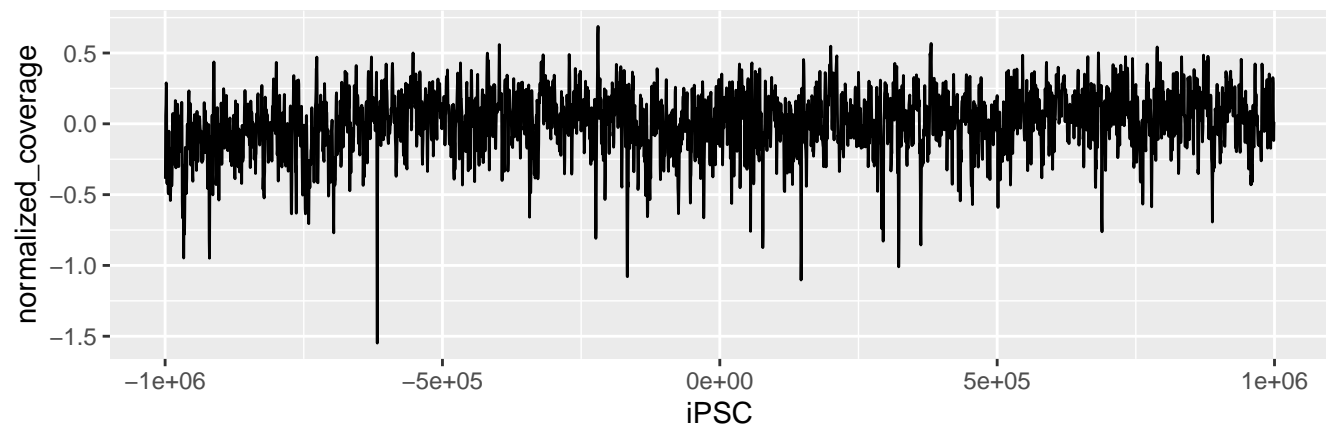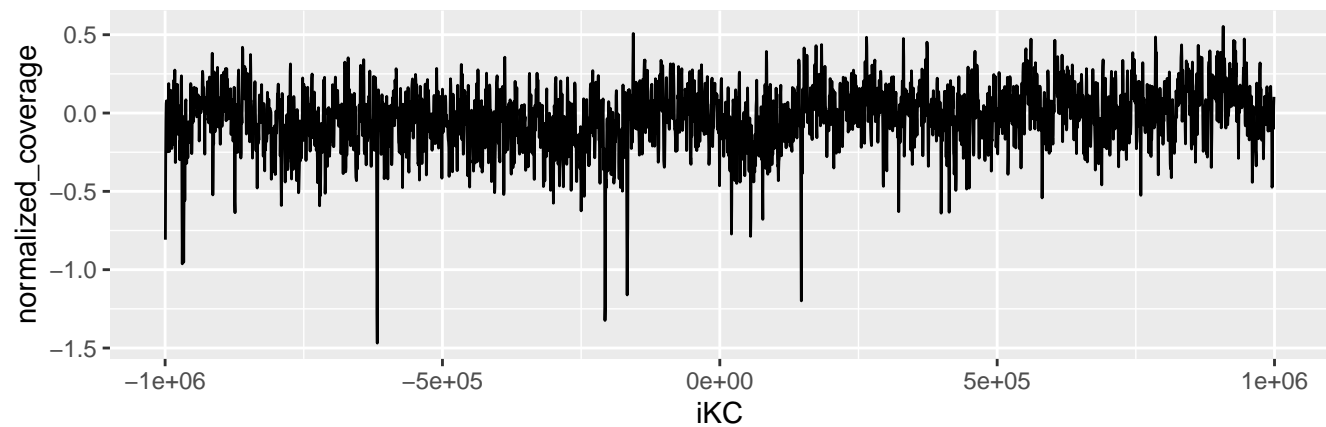

Supplement: Supplementary file 6 — Supplementary Data 3 [file 41467_2024_49400_MOESM6_ESM.zip › Supplementary Data 3/57_offtarget_sites/CO1-131_2MB/patient3_1.chr12_12267463_12267485.2MB.pdf]

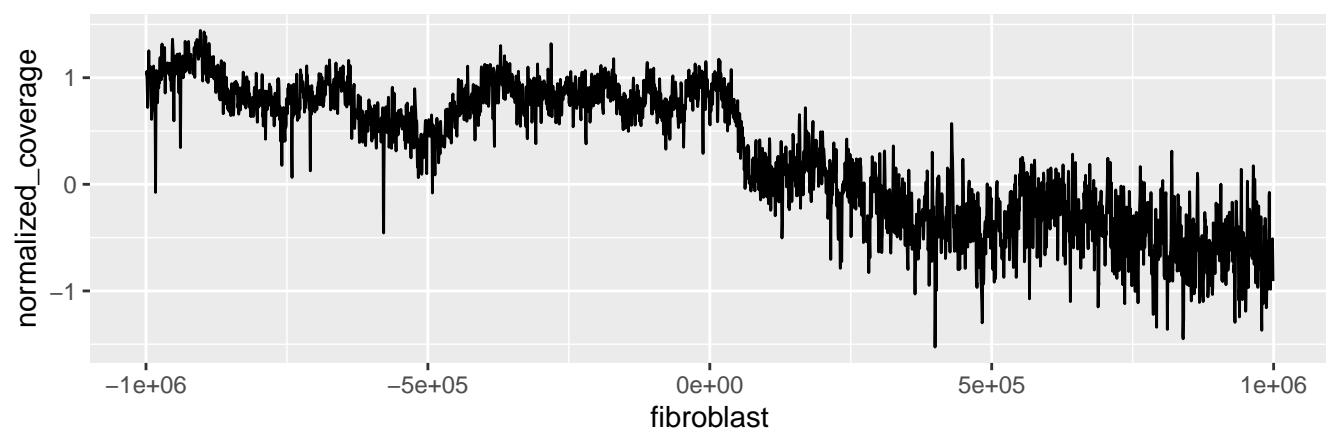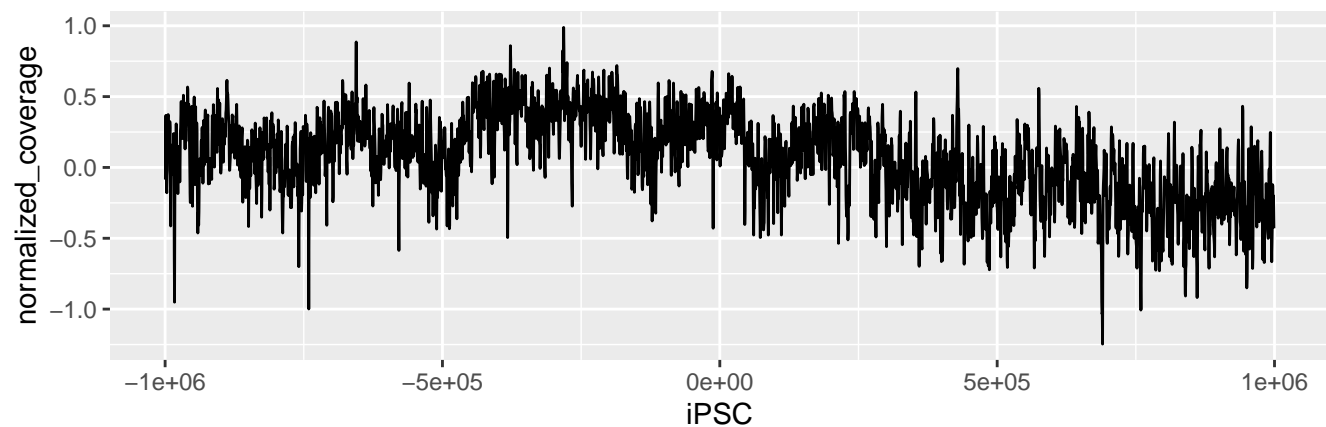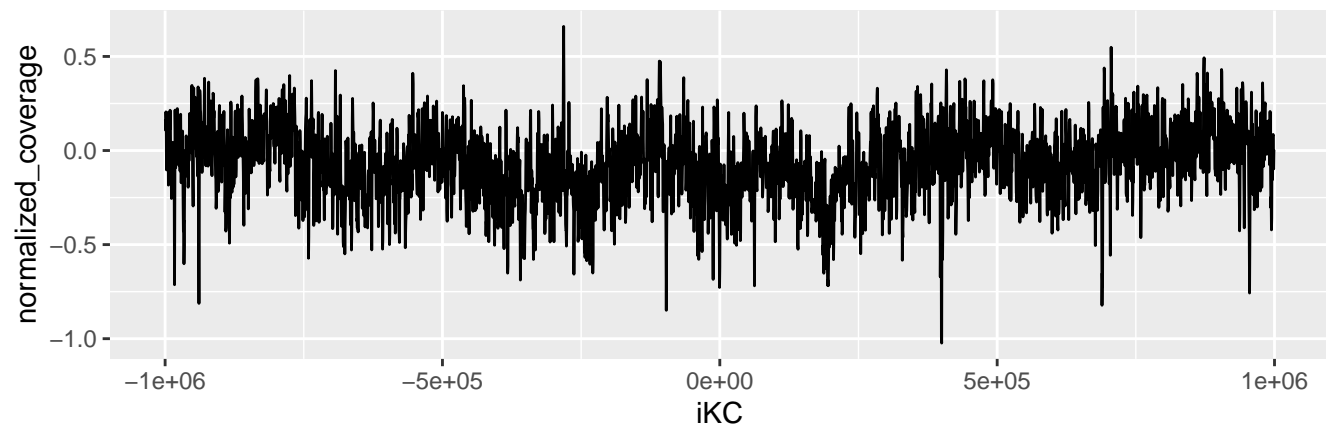

Supplement: Supplementary file 6 — Supplementary Data 3 [file 41467_2024_49400_MOESM6_ESM.zip › Supplementary Data 3/57_offtarget_sites/CO1-131_2MB/patient3_1.chr12_54391229_54391251.2MB.pdf]

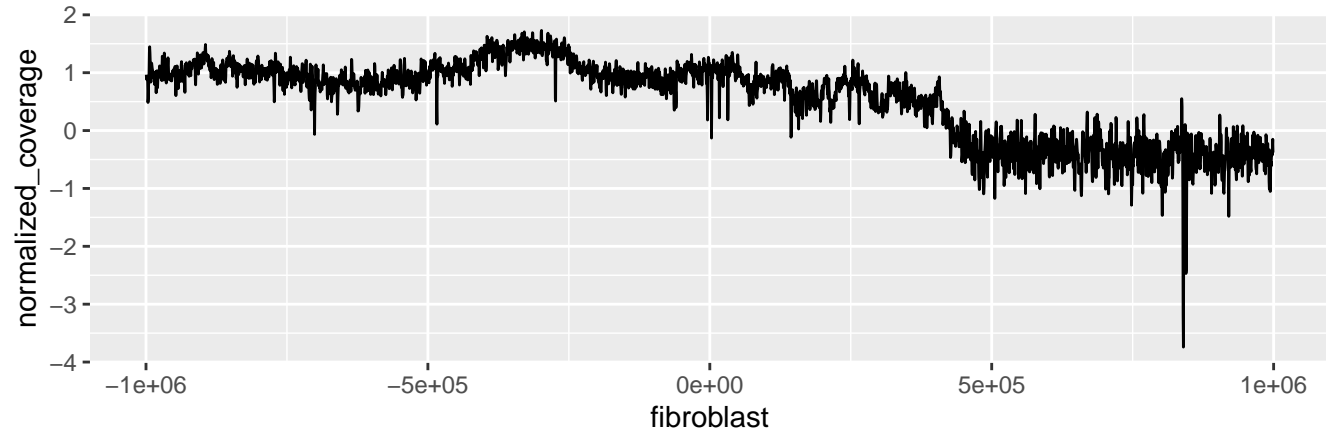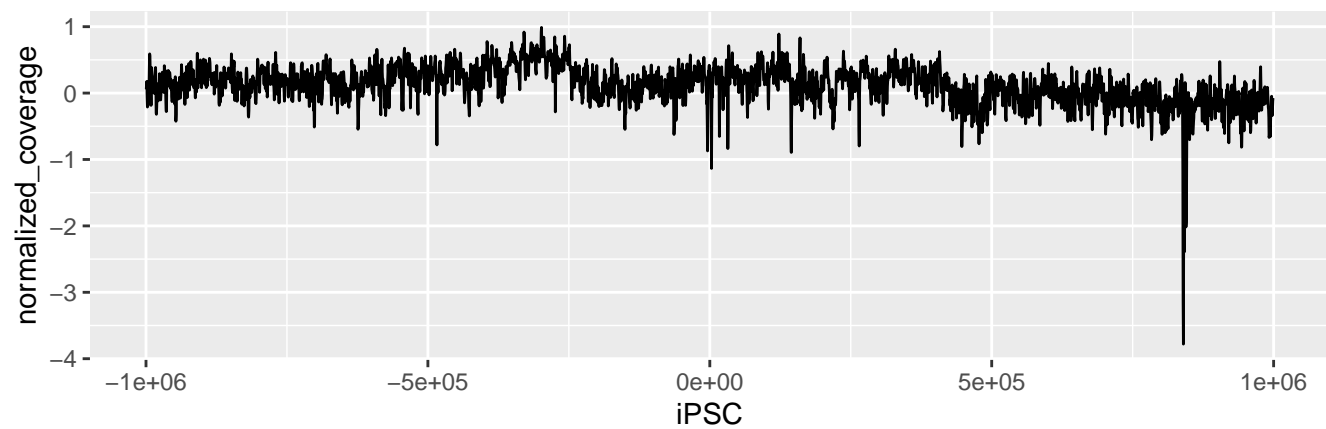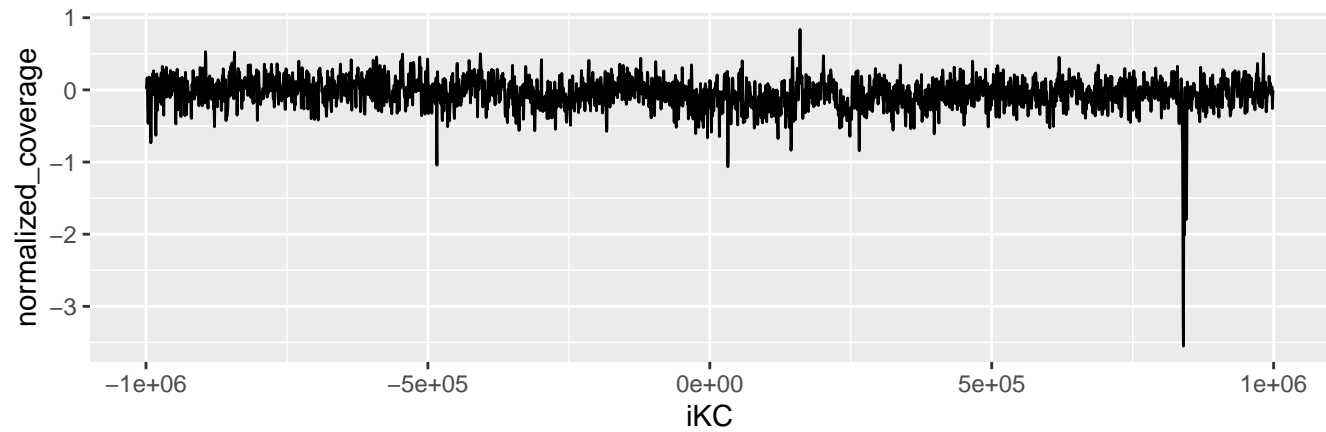

Supplement: Supplementary file 6 — Supplementary Data 3 [file 41467_2024_49400_MOESM6_ESM.zip › Supplementary Data 3/57_offtarget_sites/CO1-131_2MB/patient3_1.chr12_57490628_57490650.2MB.pdf]

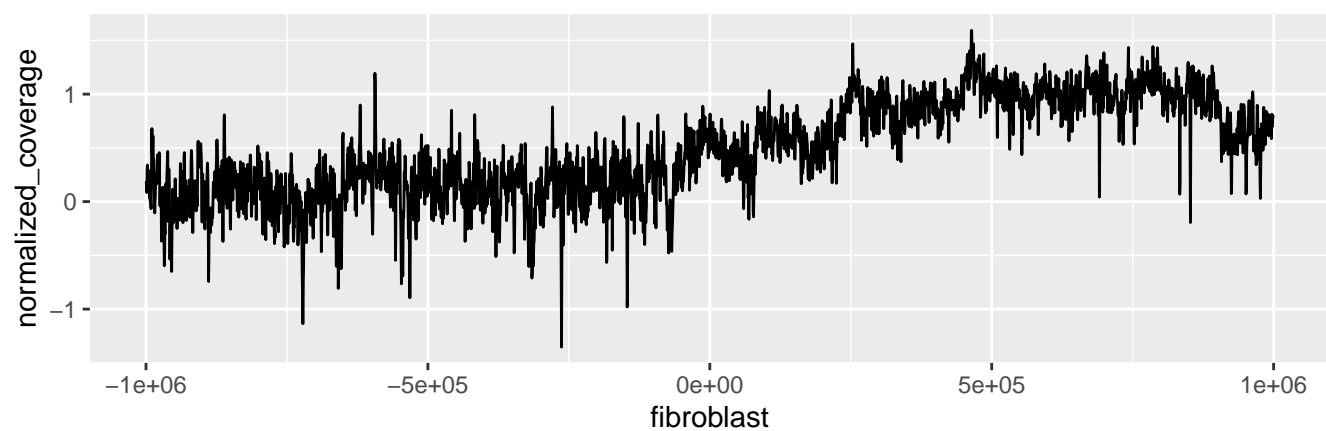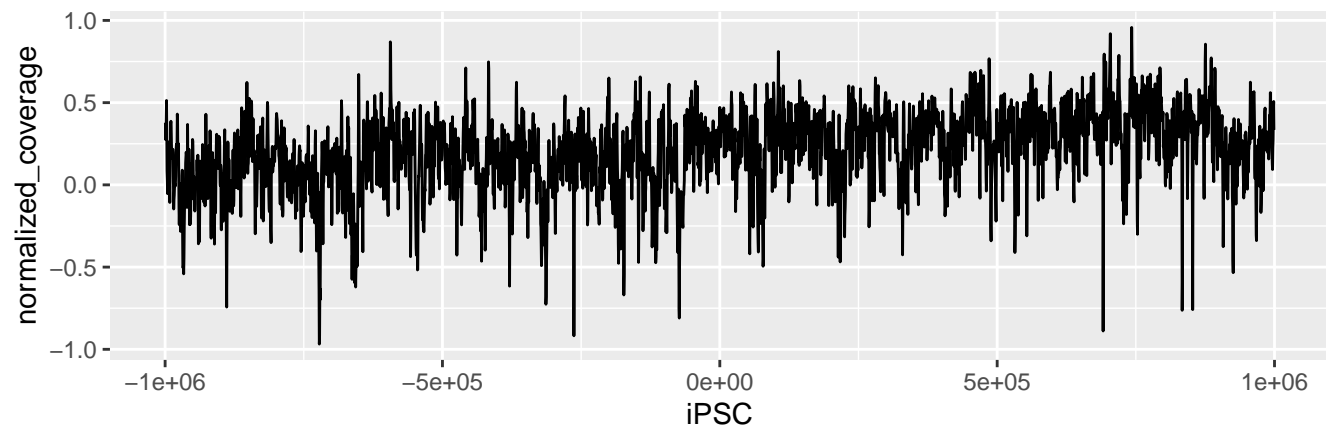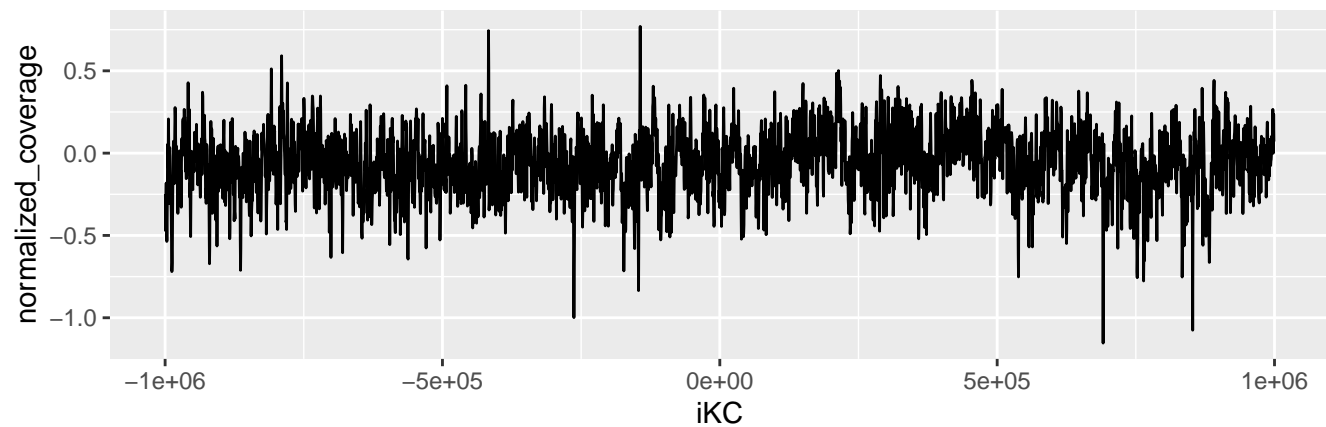

Supplement: Supplementary file 6 — Supplementary Data 3 [file 41467_2024_49400_MOESM6_ESM.zip › Supplementary Data 3/57_offtarget_sites/CO1-131_2MB/patient3_1.chr12_6075570_6075592.2MB.pdf]

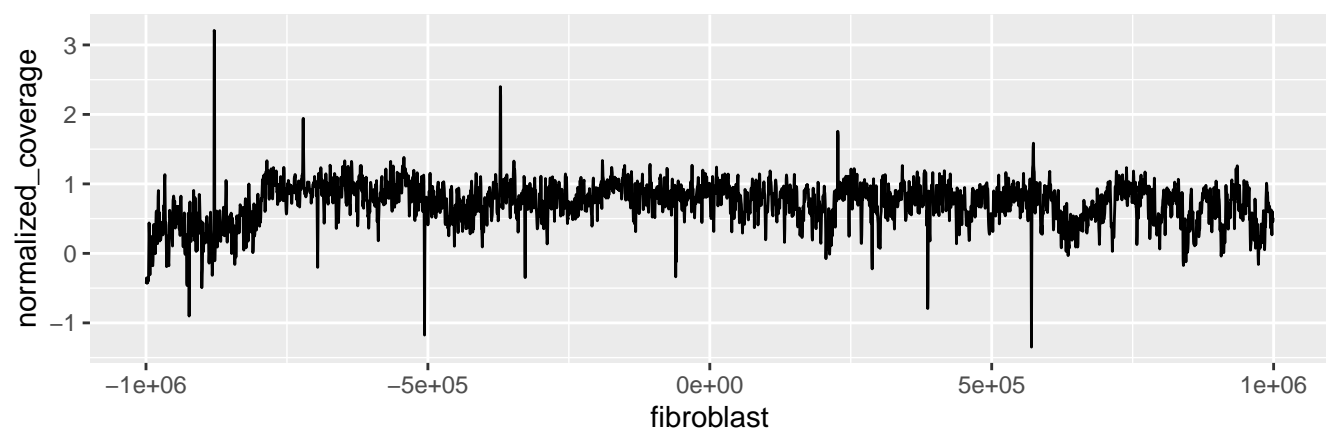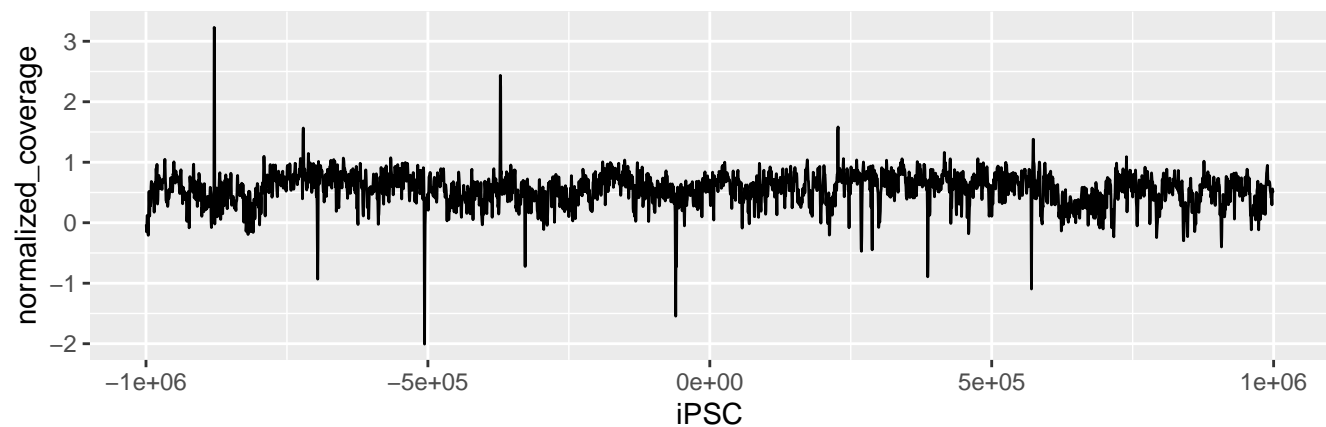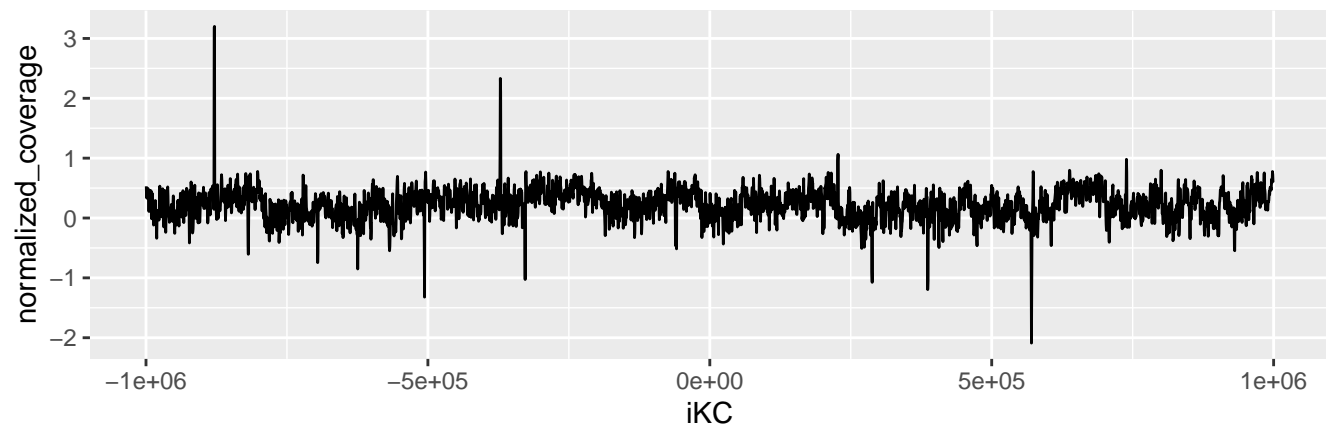

Supplement: Supplementary file 6 — Supplementary Data 3 [file 41467_2024_49400_MOESM6_ESM.zip › Supplementary Data 3/57_offtarget_sites/CO1-131_2MB/patient3_1.chr14_100299494_100299516.2MB.pdf]

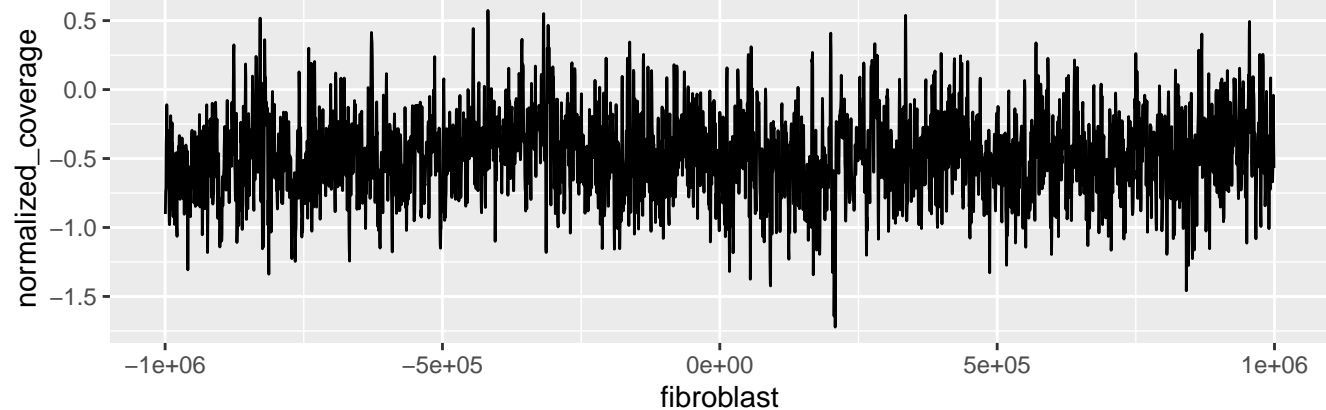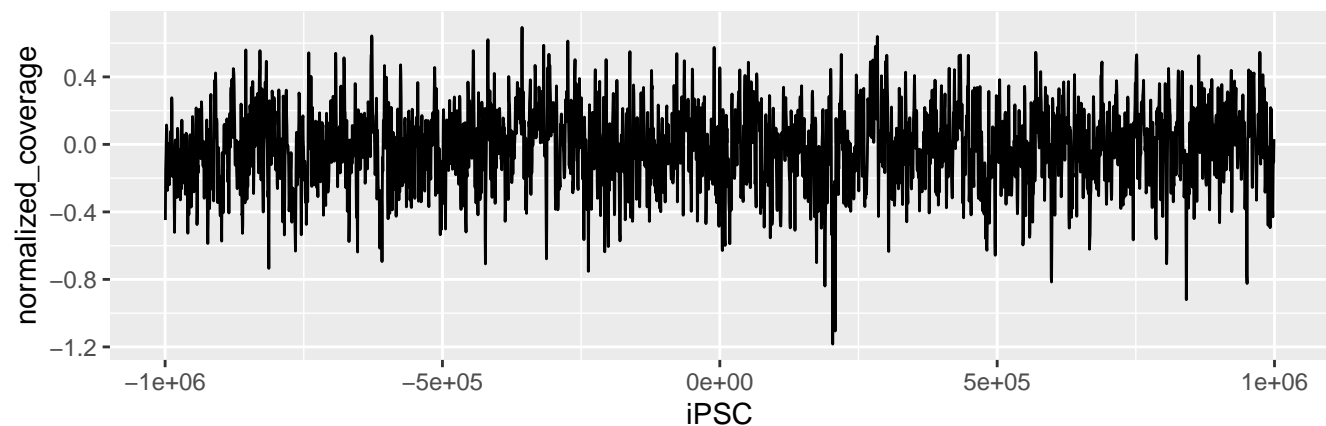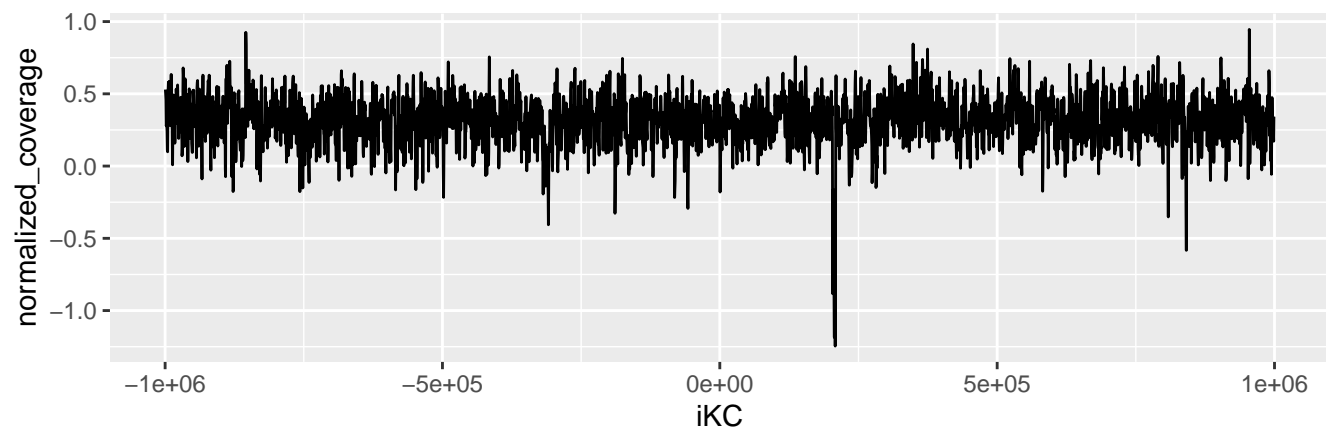

Supplement: Supplementary file 6 — Supplementary Data 3 [file 41467_2024_49400_MOESM6_ESM.zip › Supplementary Data 3/57_offtarget_sites/CO1-131_2MB/patient3_1.chr14_47984104_47984126.2MB.pdf]

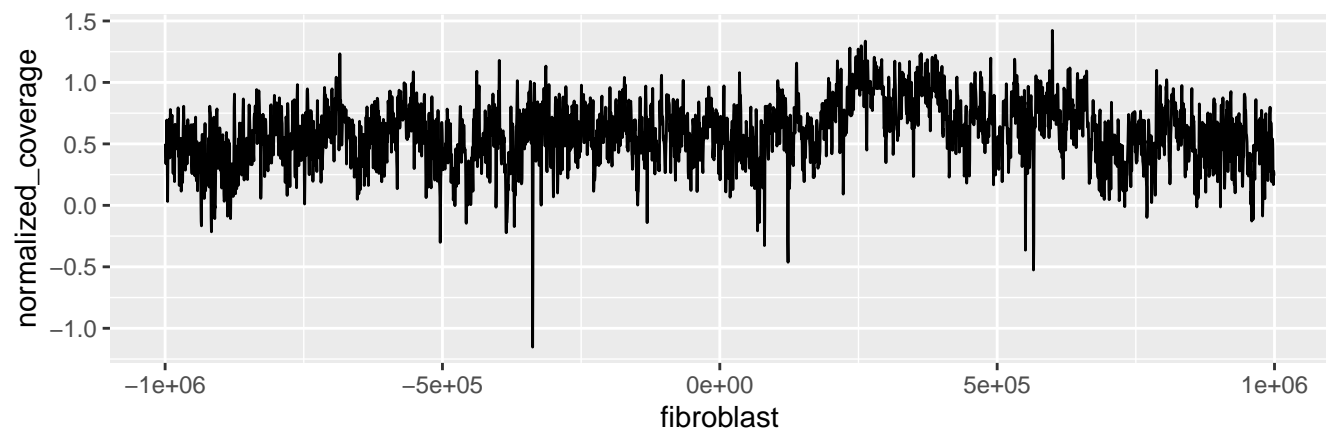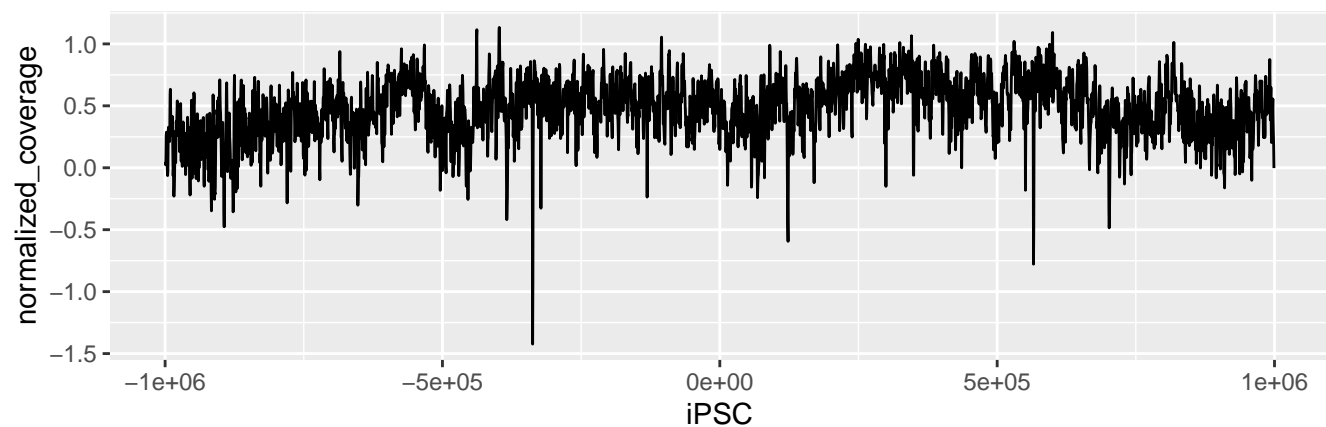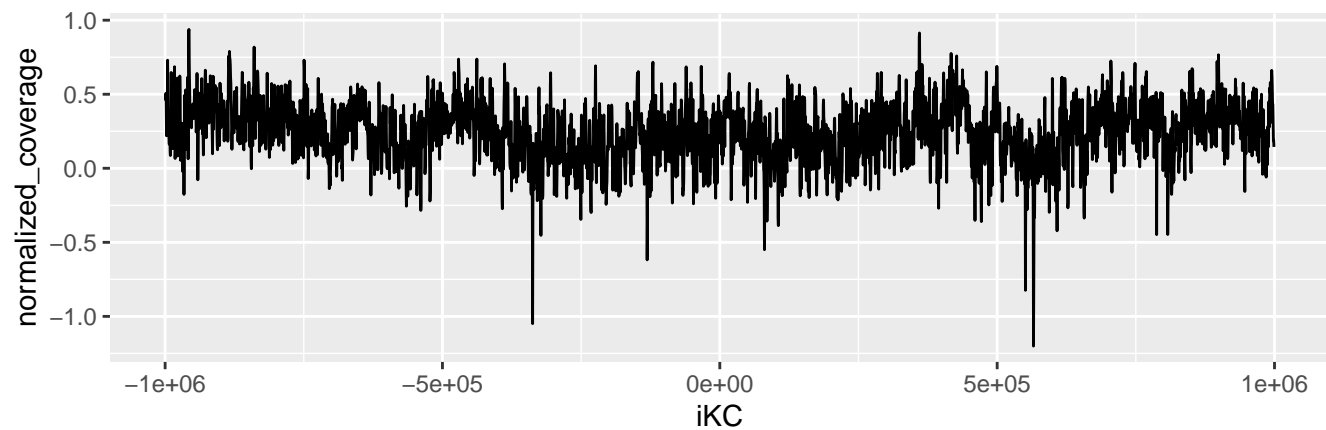

Supplement: Supplementary file 6 — Supplementary Data 3 [file 41467_2024_49400_MOESM6_ESM.zip › Supplementary Data 3/57_offtarget_sites/CO1-131_2MB/patient3_1.chr14_76670168_76670190.2MB.pdf]

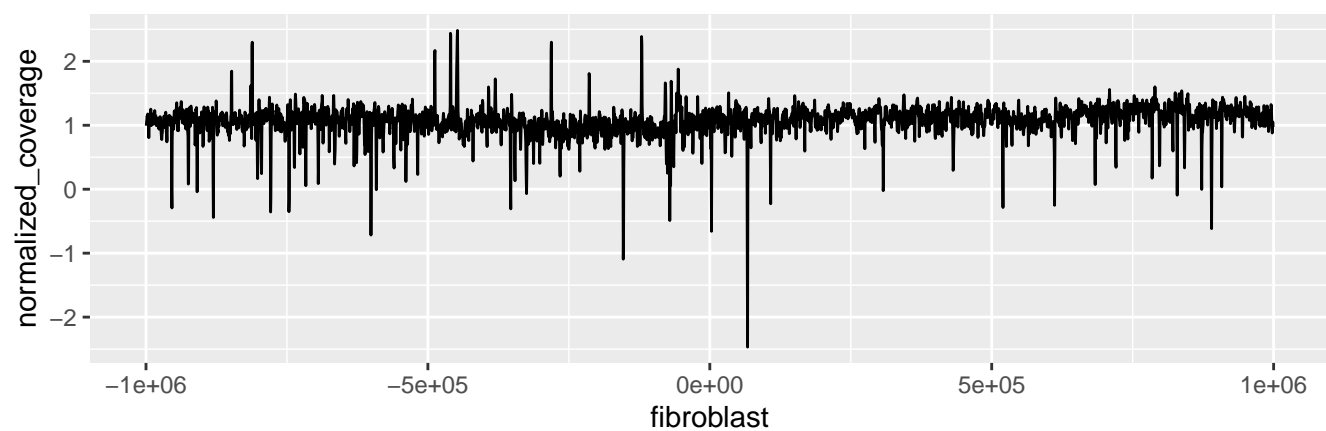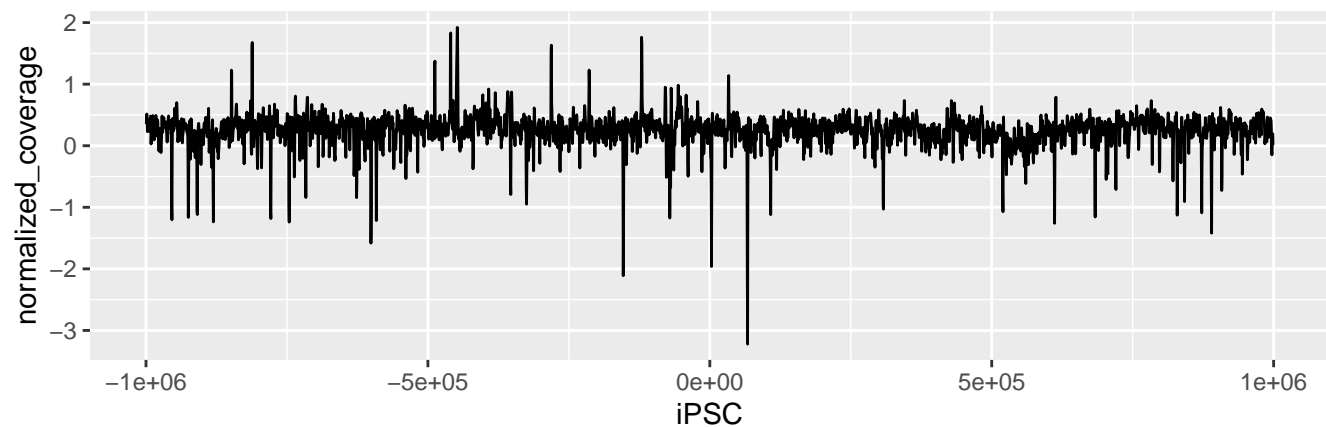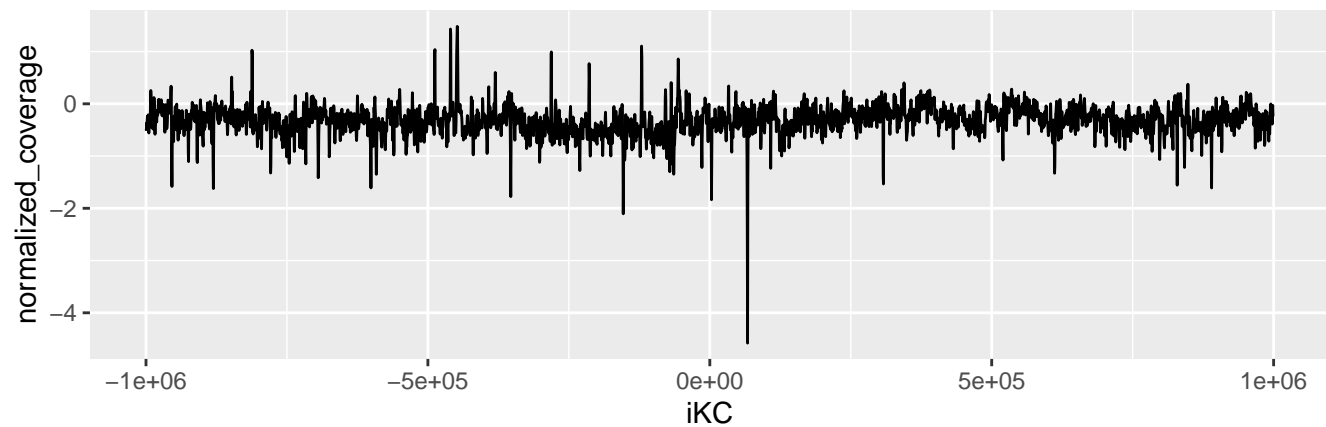

Supplement: Supplementary file 6 — Supplementary Data 3 [file 41467_2024_49400_MOESM6_ESM.zip › Supplementary Data 3/57_offtarget_sites/CO1-131_2MB/patient3_1.chr16_1306676_1306698.2MB.pdf]

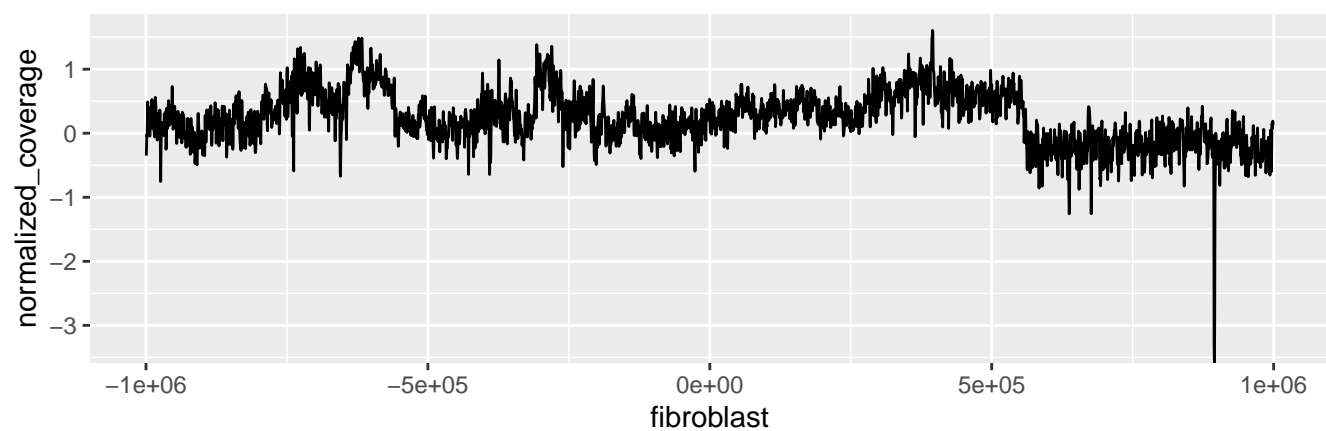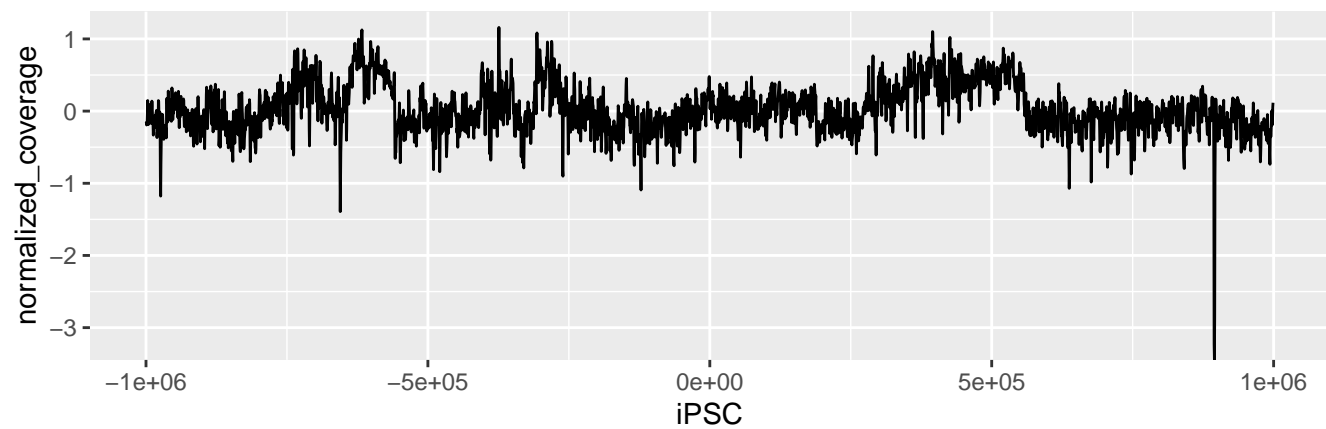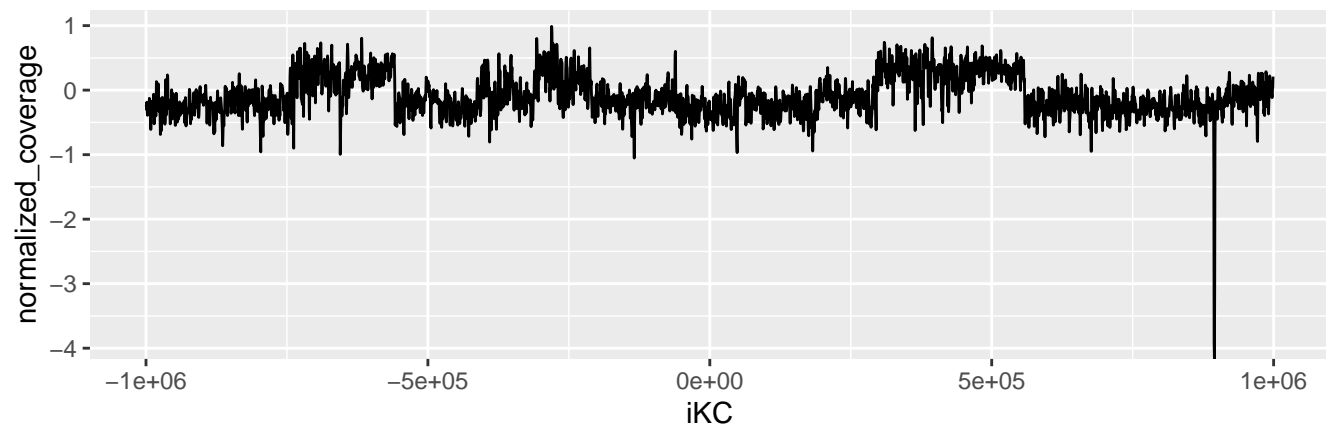

Supplement: Supplementary file 6 — Supplementary Data 3 [file 41467_2024_49400_MOESM6_ESM.zip › Supplementary Data 3/57_offtarget_sites/CO1-131_2MB/patient3_1.chr16_22142360_22142382.2MB.pdf]

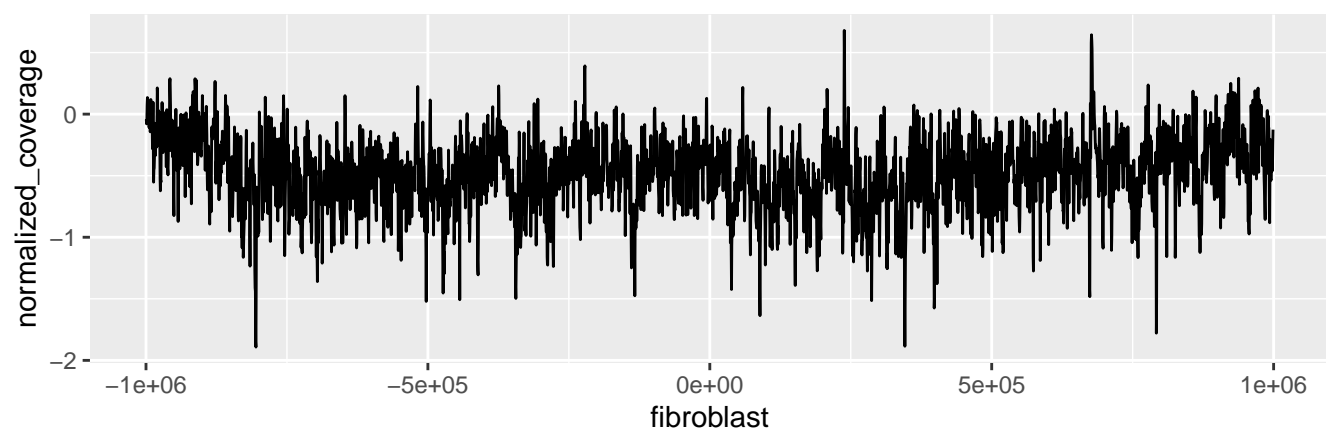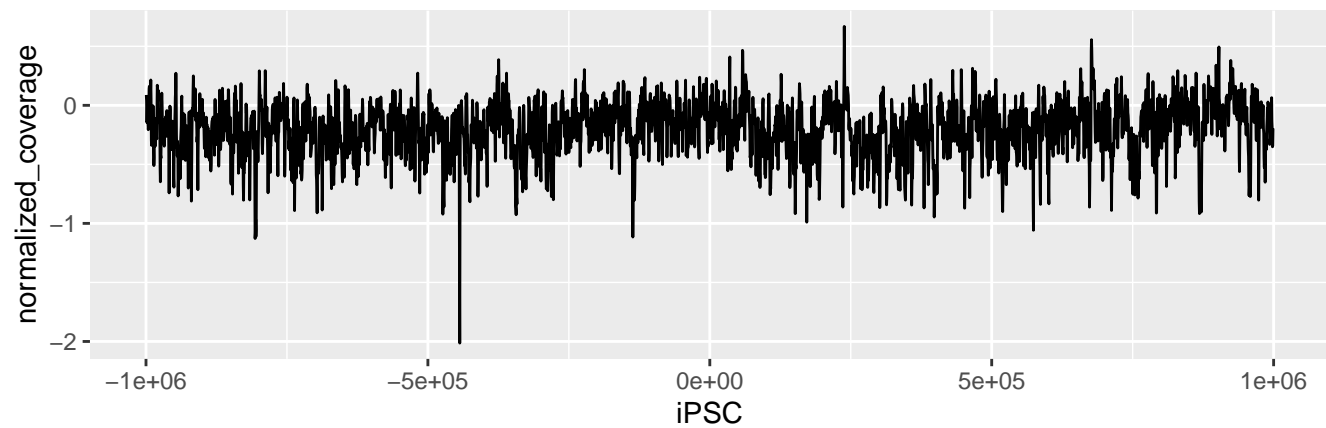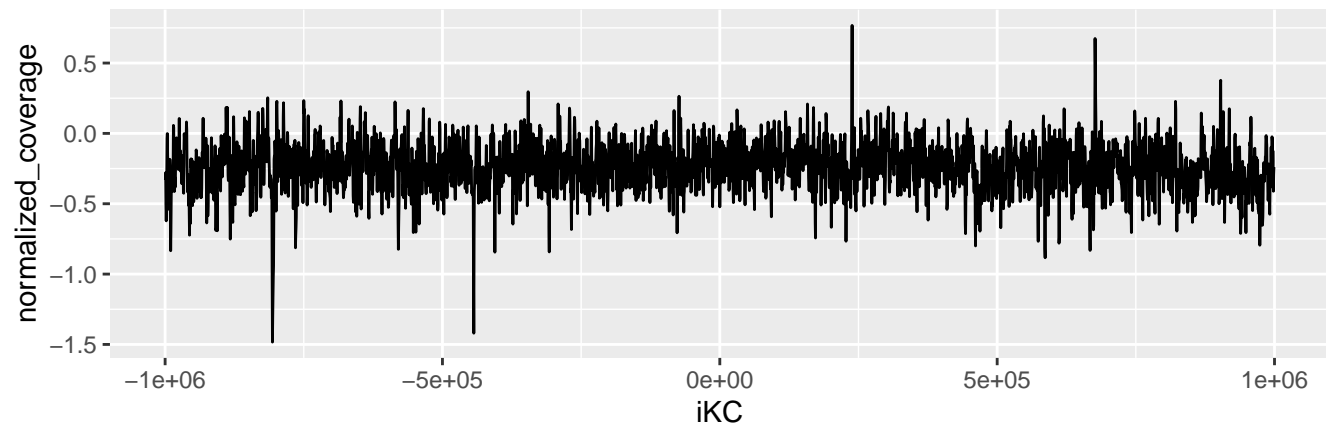

Supplement: Supplementary file 6 — Supplementary Data 3 [file 41467_2024_49400_MOESM6_ESM.zip › Supplementary Data 3/57_offtarget_sites/CO1-131_2MB/patient3_1.chr16_26135954_26135976.2MB.pdf]

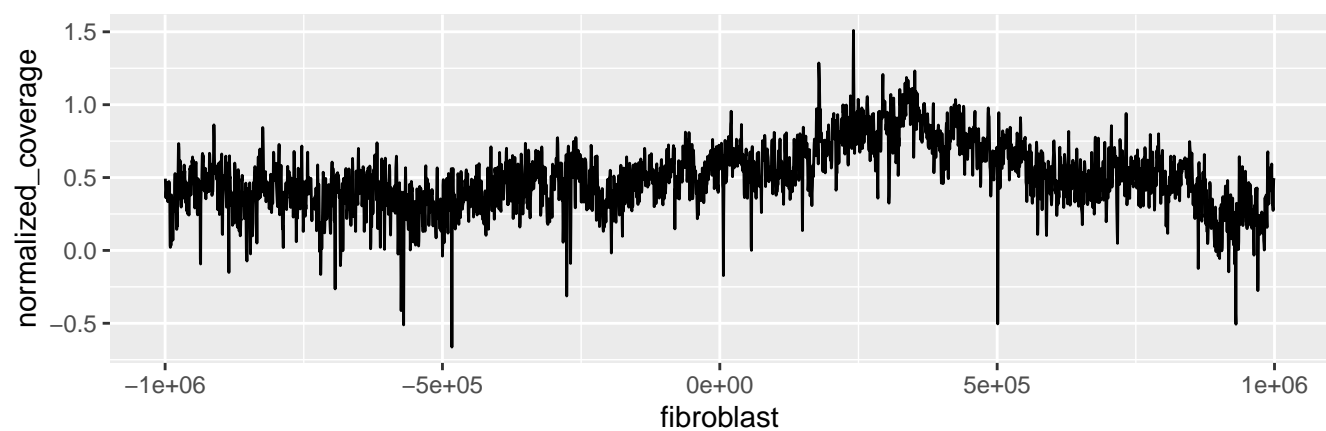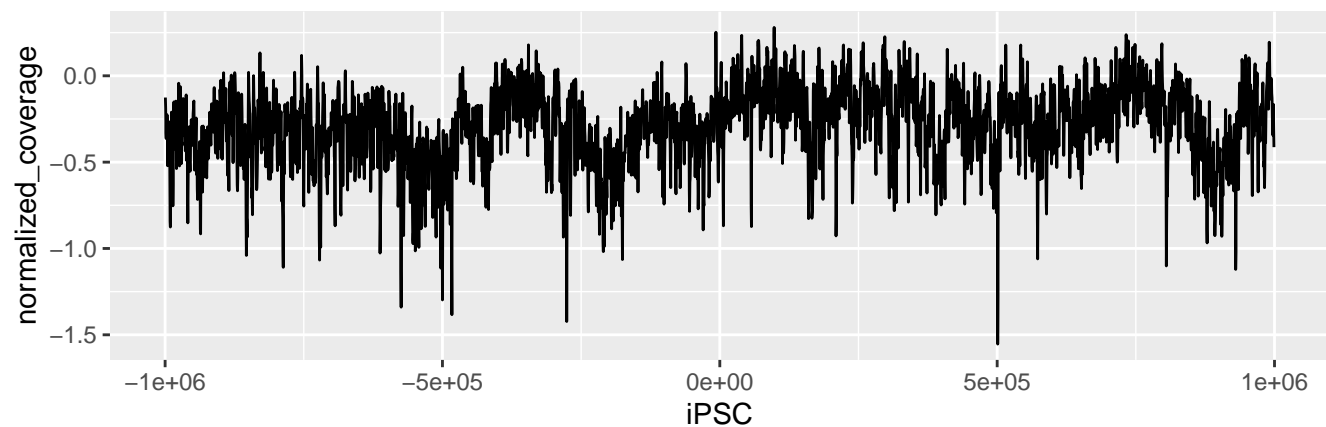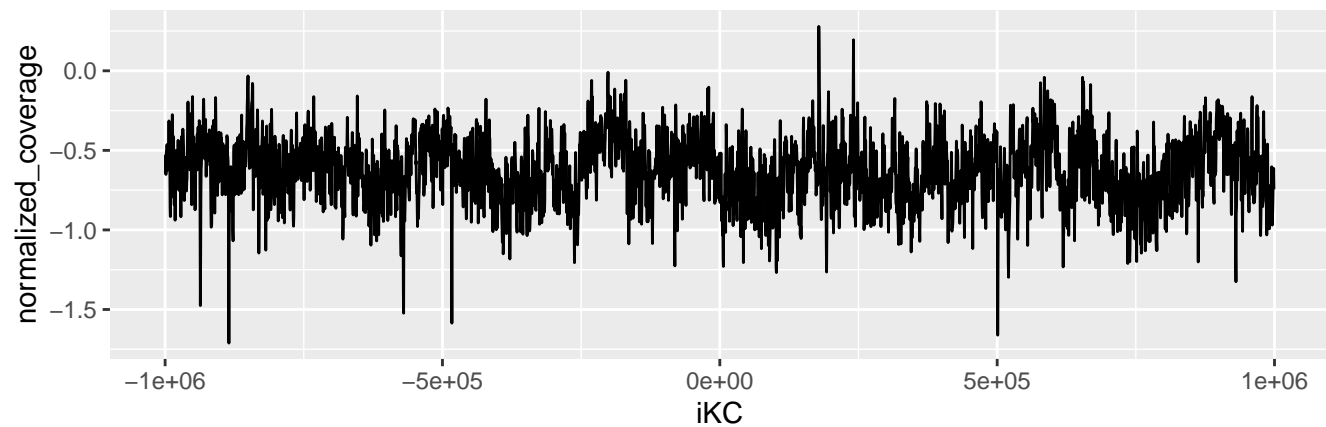

Supplement: Supplementary file 6 — Supplementary Data 3 [file 41467_2024_49400_MOESM6_ESM.zip › Supplementary Data 3/57_offtarget_sites/CO1-131_2MB/patient3_1.chr17_49845081_49845103.2MB.pdf]

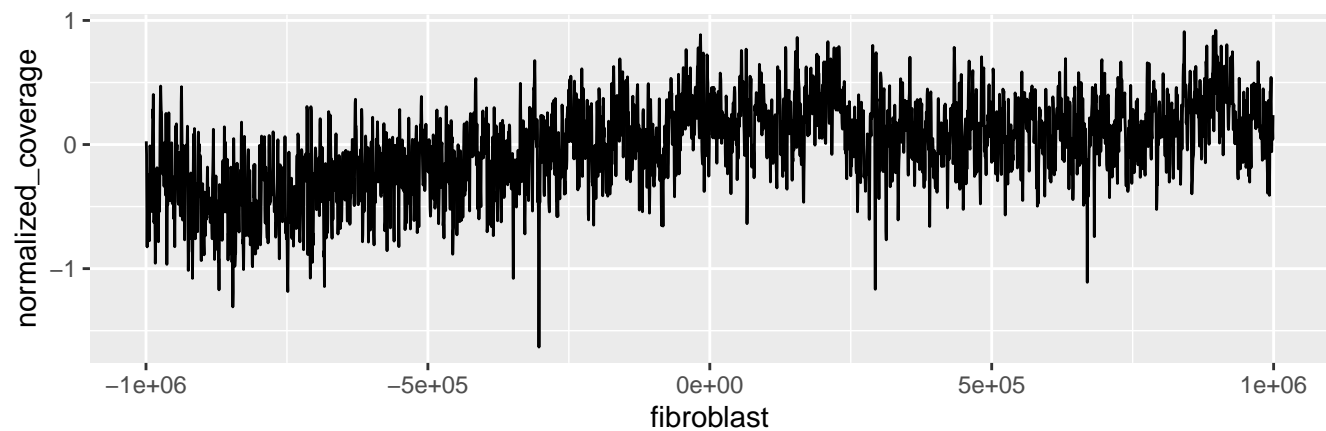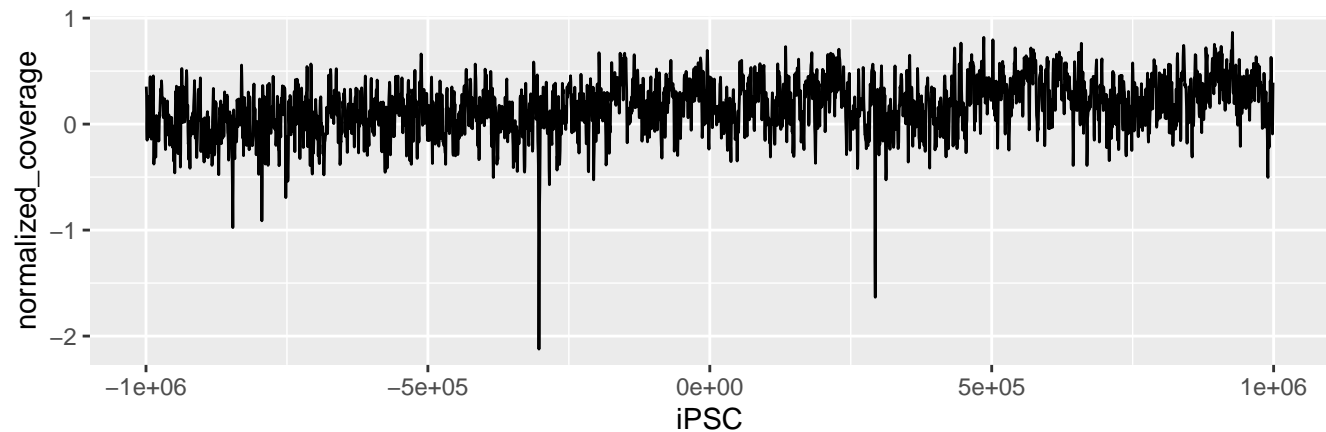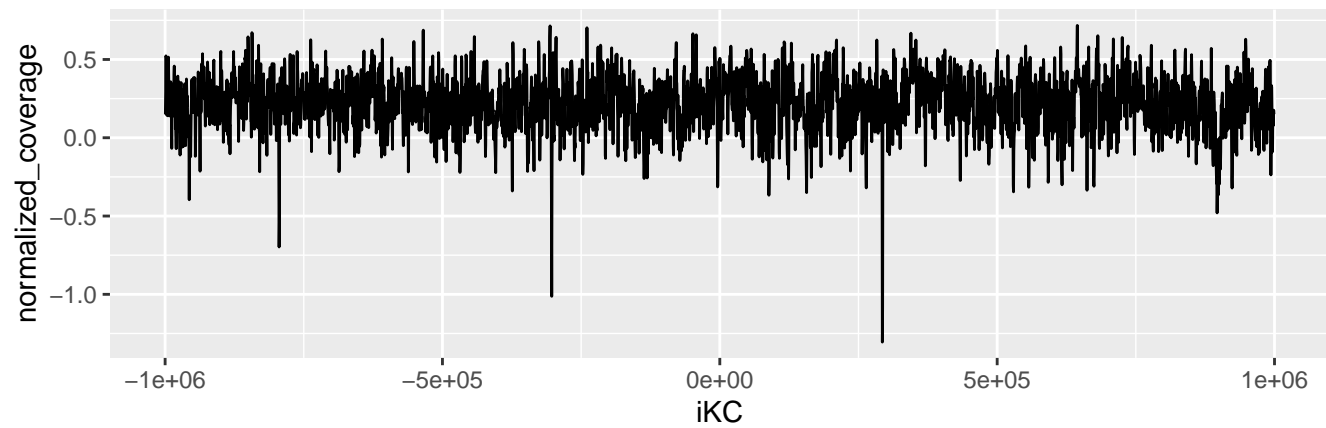

Supplement: Supplementary file 6 — Supplementary Data 3 [file 41467_2024_49400_MOESM6_ESM.zip › Supplementary Data 3/57_offtarget_sites/CO1-131_2MB/patient3_1.chr18_35288320_35288342.2MB.pdf]

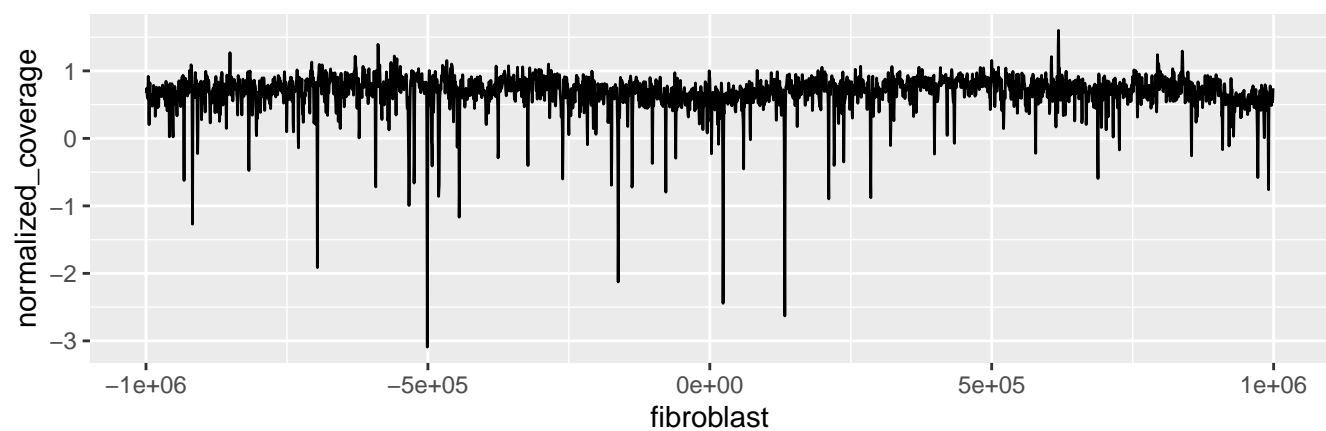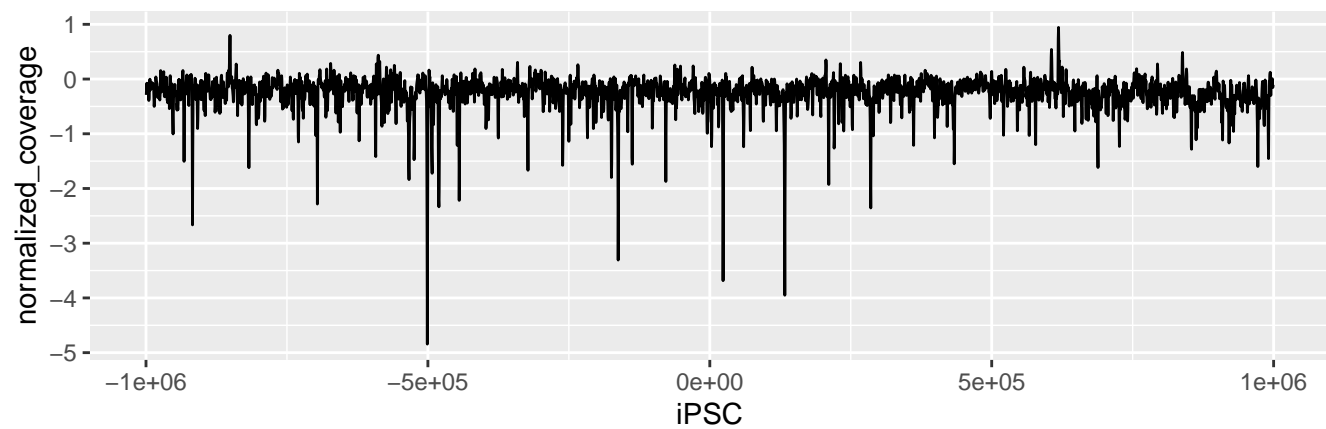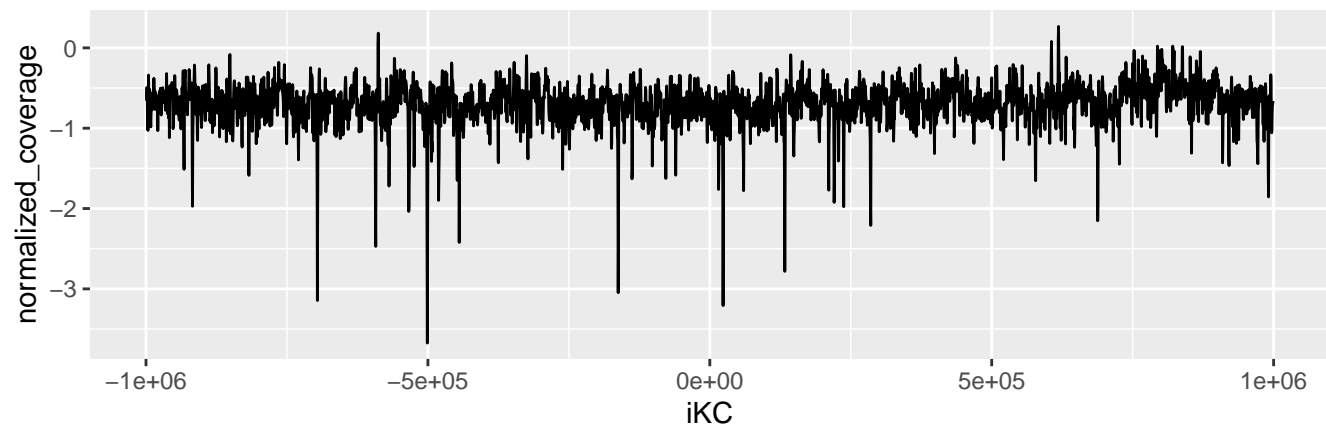

Supplement: Supplementary file 6 — Supplementary Data 3 [file 41467_2024_49400_MOESM6_ESM.zip › Supplementary Data 3/57_offtarget_sites/CO1-131_2MB/patient3_1.chr19_1730409_1730431.2MB.pdf]

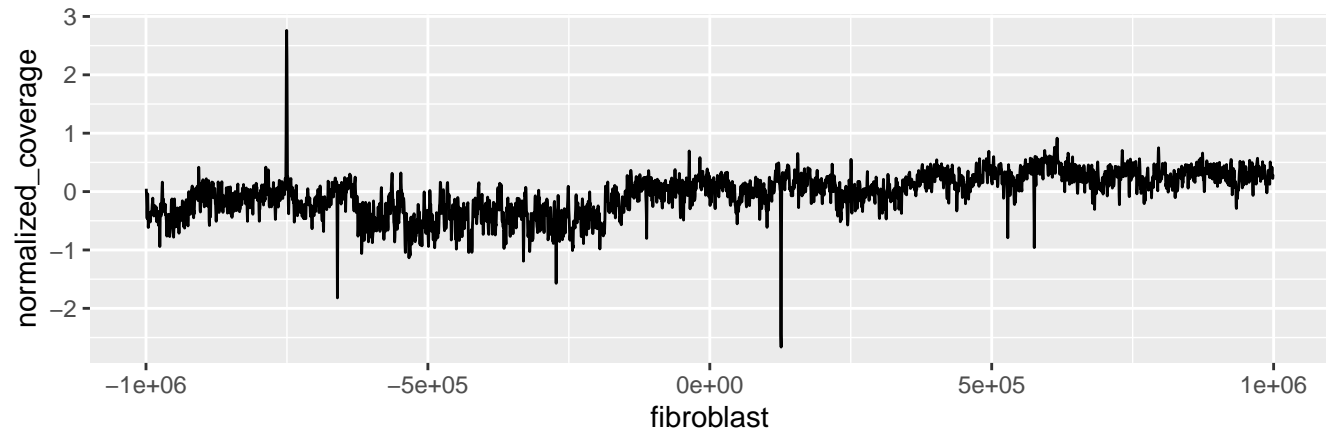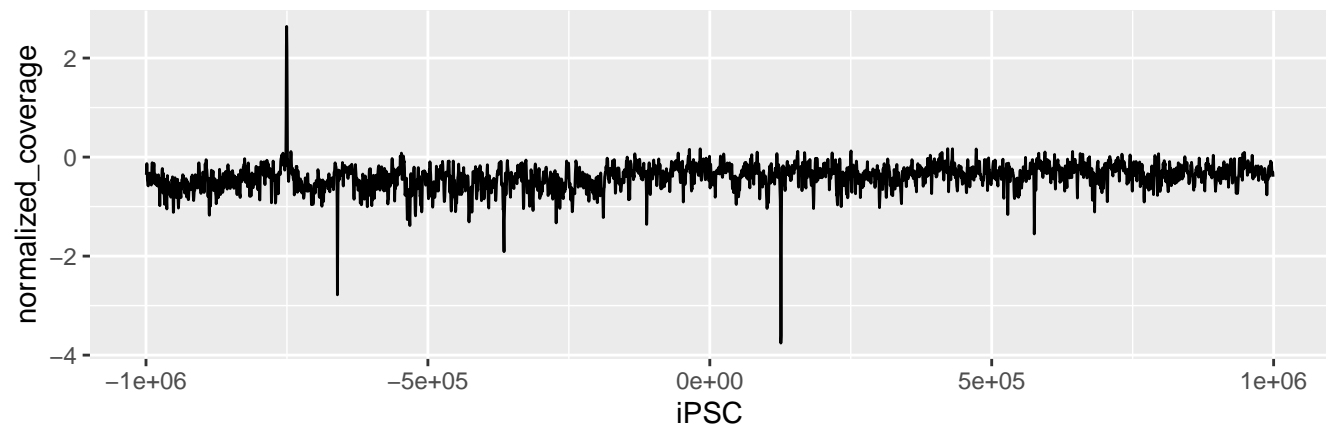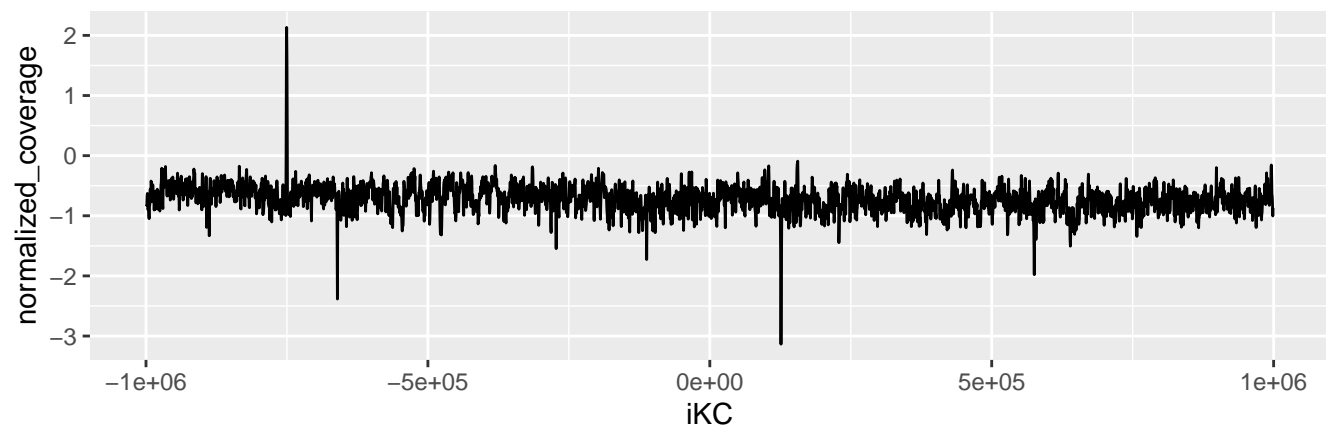

Supplement: Supplementary file 6 — Supplementary Data 3 [file 41467_2024_49400_MOESM6_ESM.zip › Supplementary Data 3/57_offtarget_sites/CO1-131_2MB/patient3_1.chr19_35142505_35142527.2MB.pdf]

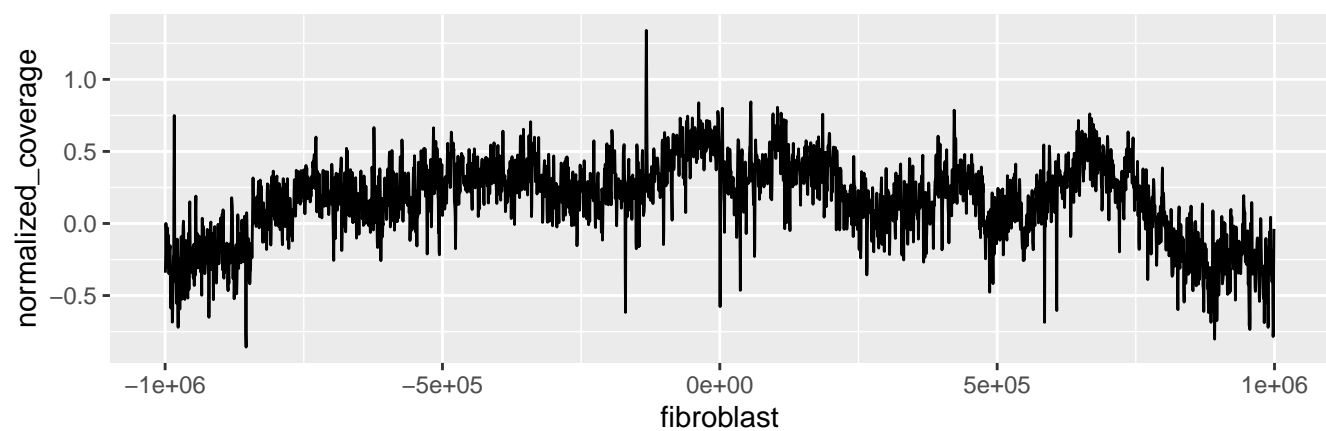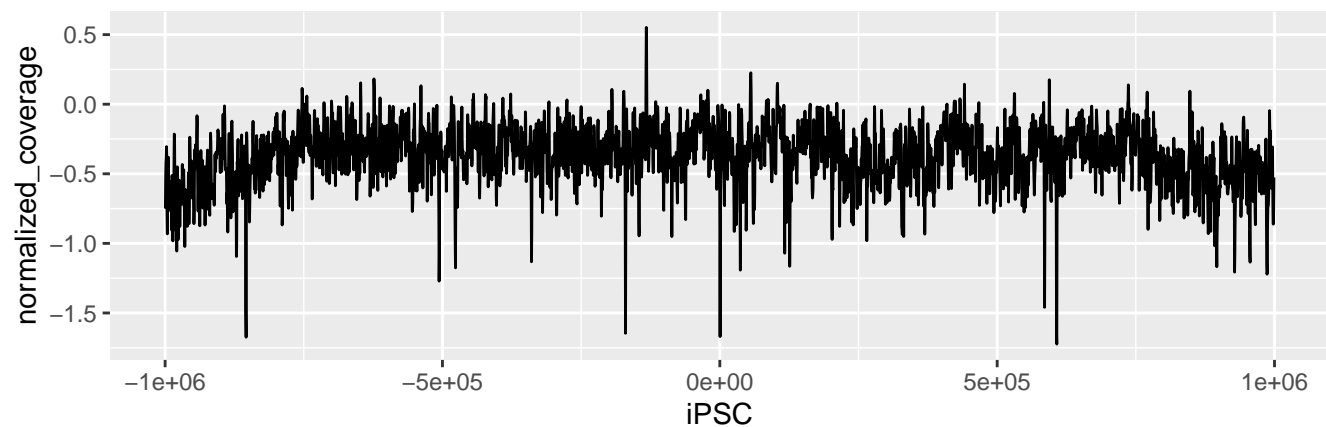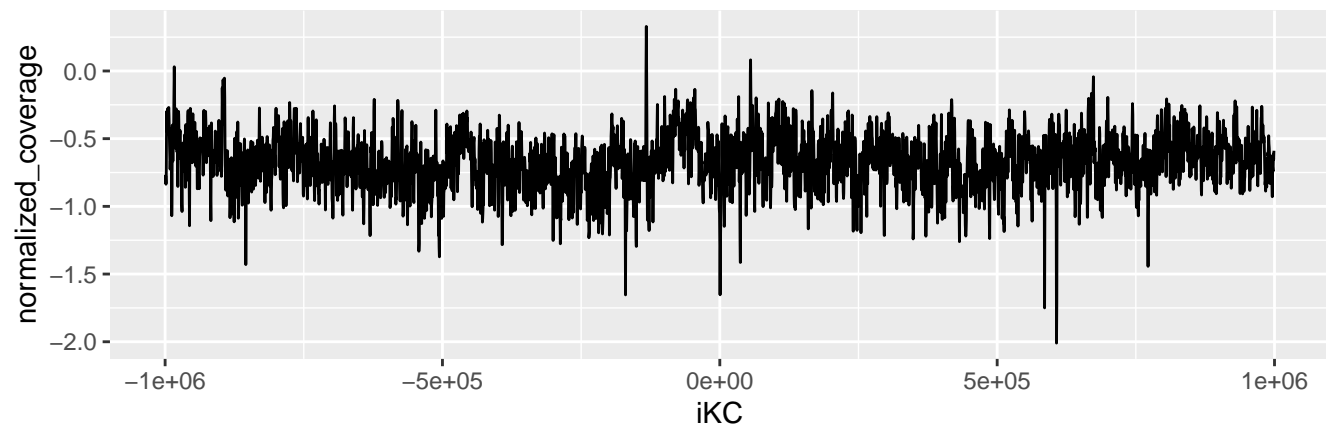

Supplement: Supplementary file 6 — Supplementary Data 3 [file 41467_2024_49400_MOESM6_ESM.zip › Supplementary Data 3/57_offtarget_sites/CO1-131_2MB/patient3_1.chr19_38735330_38735352.2MB.pdf]
