# Supplementary material for: A scalable and cGMP-compatible autologous organotypic cell therapy for Dystrophic Epidermolysis Bullosa
Source: Nat Commun. 2024 Jul 11;15:5834. doi: 10.1038/s41467-024-49400-z (PMC11239819; doi:10.1038/s41467-024-49400-z)
Supplement: Supplementary file 7 — Reporting Summary [file 41467_2024_49400_MOESM7_ESM.pdf]

nature portfolio

Corresponding author(s): Drs. Marius Wernig (wernig@stanford.edu) and Anthony E. Oro (oro@stanford.edu)

Last updated by author(s): Apr 5, 2024

Reporting Summary

Nature Portfolio wishes to improve the reproducibility of the work that we publish. This form provides structure for consistency and transparency in reporting. For further information on Nature Portfolio policies, see our Editorial Policies and the Editorial Policy Checklist.

Statistics

For all statistical analyses, confirm that the following items are present in the figure legend, table legend, main text, or Methods section.

n/a	Confirmed	
☐	☒	The exact sample size (*n*) for each experimental group/condition, given as a discrete number and unit of measurement
☐	☒	A statement on whether measurements were taken from distinct samples or whether the same sample was measured repeatedly
☐	☒	The statistical test(s) used AND whether they are one- or two-sided<br>*Only common tests should be described solely by name; describe more complex techniques in the Methods section.*
☒	☐	A description of all covariates tested
☒	☐	A description of any assumptions or corrections, such as tests of normality and adjustment for multiple comparisons
☐	☒	A full description of the statistical parameters including central tendency (e.g. means) or other basic estimates (e.g. regression coefficient) AND variation (e.g. standard deviation) or associated estimates of uncertainty (e.g. confidence intervals)
☒	☐	For null hypothesis testing, the test statistic (e.g. *F*, *t*, *r*) with confidence intervals, effect sizes, degrees of freedom and *P* value noted<br>*Give P values as exact values whenever suitable.*
☒	☐	For Bayesian analysis, information on the choice of priors and Markov chain Monte Carlo settings
☒	☐	For hierarchical and complex designs, identification of the appropriate level for tests and full reporting of outcomes
☒	☐	Estimates of effect sizes (e.g. Cohen's *d*, Pearson's *r*), indicating how they were calculated

Our web collection on statistics for biologists contains articles on many of the points above.

Software and code

Policy information about availability of computer code

Data collection

Data from agarose gels were collected using a BioRad Molecular Imager Gel Doc XR+ imaging system with associated software, i.e. Image Lab 5.1.
FACS and flow cytometry data were acquired on a BD LSRII with associated BD FACSDiva v8.0.1 software.
Immunofluorescence microscopy data was collected using a Leica DMi8 with associated software, i.e. Leica Application Suite X 3.7.4.23463, a Carl Zeiss Axio Observer.Z1 inverted microscope with Axiovision software, or a Leica TCS SP5 confocal laser scanning microscope with associated software, i.e. Leica Application Suite for Advanced Fluorescence software v.2.7.9.
ddPCR data was collected using a BioRad QX200 system with associated software, i.e. QuantaSoft Version 1.7.4.0917.
TIDE data was collected using the online tool available at https://tide.nki.nl/
CIRSPR/CAS9 off target prediction was done using the online tool available at http://crispor.tefor.net/crispor.py.
scRNAseq data was collected using Illumina HiSeq and NextSeq System Suits.
The RNA-seq libraries were sequenced on Illumina Hiseq2000 or NextSeq sequencers.
RTPCR data was collected using Roche LightCycler 480 Software 1.5.1.
IVIS Optical Imaging system was used to detect the bioluminescence signal in the skin grafts made from RDEB-SCC cells expressing the luciferase gene.

Data analysis

Agarose gel TIFF files were processed in Adobe Photoshop CS6 (64bit).
FACS and flow cytometry data analysis was performed with FlowJo v10.6 software.
Immunofluorescence microscopy files were processed in Adobe Photoshop CS6 (64bit) or Adobe Illustrator v26.
ddPCR data was analyzed using QuantaSoft Analysis Pro Software Version 1.0.596 and Microsoft Office Excel. ddPCR plots were prepared

using Adobe Photoshop CS6 (64bit).
TIDE data was analyzed using the online tool available at https://tide.nki.nl/ and Microsoft Office Excel. TIDE plots were prepared using Adobe Photoshop CS6 (64bit).

Whole genome sequencing raw data provided by Novogene after sequencing via the NovaSeq 6000 S4 platform was analyzed using the following software: cutadapt (v2.3; EMBnet.journal 17, 10–12, 2011) in pair-end mode for trimming, BWA-MEM (v0.7.17-r1188; Bioinformatics 25, 1754–1760, 2009) for mapping, GATK4 (v.4.1.9.0; Genome Res. 20, 1297–1303, 2010) for sorting and recalibration of base quality scores (BQSRs). For BSQR, we applied below resources of known human variant sets to train the base quality model, as recommended by GATK best practice workflow (all files were downloaded from GATK resource bundle here: https://console.cloud.google.com/storage/browser/genomics-public-data/resources/broad/hg38/v0/):
1) hapmap_3.3.hg38.vcf.gz(.tbi)
2) Mills_and_1000G_gold_standard.indels.hg38.vcf.gz(.tbi)
3) Homo_sapiens_assembly38.dbsnp138.vcf(.tbi)
4) Homo_sapiens_assembly38.known_indels.vcf(.tbi)

Read pre-processing was performed using the embedded PICARD toolset in GATK. HaplotypeCaller (Genome Res. 20, 1297–1303, 2010; bioRxiv 201178, 2018, doi:10.1101/201178), Mutect2 (Nat. Biotechnol. 31, 213–219, 2013), Lofreq2 (Nucleic Acids Res. 40, 11189–11201, 2012) and Scalpel (Nat Protoc 11, 2529–2548, 2016) were used to call variants and ANNOVAR (Nucleic Acids Res. 38, e164, 2010) with the embedded refGene protocol for hg38 was used for functional annotation. We ran HaplotypeCaller with parameters "-ERC GVCF" to generate .gvcf files for each individual sample, followed by joint variant calling combining all 11 .bam files of 3 patients. The raw .vcf files were filtered off low-quality variants that didn't pass variant quality score recalibration (VQSR) using recommended external variant datasets for constructing separate models for SNPs and indels. As the filtered .vcf files contain unknown genotypes indicated as "./." in the GT field, we next filtered the variants with "vcftools –max-missing 0.5" and imputed the missing genotypes using BEAGLE (v18May20.d20). Mutect2 was ran on a per-individual basis combining all .bam files from each patient. The resulted .vcf files were filtered off low-quality variants using command "gatk FilterMutectCalls" with default parameters. Lofreq called SNP variants with the "call-parallel" mode in each sample .bam file separately. Same external variant files indicated in the read pre-processing section was applied here using the "-s" function. Due to computational speed and memory consumption concern of the algorithm, we split the genome into 2MB windows for running Scalpel indel callings parallelly on a HPC cluster. The splited .vcf files from all windows were then merged into a single .vcf file containing all indels in the sample.
Unsupervised K-means clustering was conducted using function "kmeans" in R package "stats".
GO enrichment analysis was performed via the online-tool at http://geneontology.org/
Homology search between variant sites and the sequence of sgRNA C4 (Fig 4D) was conducted using a custom script available at our GitHub repository: https://github.com/shli-embl/hg_wgs_variant_calling/
Sanger sequencing data provided by Elim Biopharm was analyzed using Applied Biosystems Sequence Scanner Software 2, Microsoft Office Word, sequence alignment software tools available at https://blast.ncbi.nlm.nih.gov/Blast.cgi, and Adobe Photoshop CS6 (64bit).
The luminescence intensity of the luciferase assay in regions of transplanted cells from each image was quantified via an automated software process in Living Image software. (Caliper (a PerkinElmer company), Columbia University Herbert Irving Comprehensive Cancer Center, New York, NY)
scRNAseq data was analyzed using Seurat v4.0.0, cellRanger v3.1.0, R v4.0.0, Tophat v2.1.1, samtools v1.8, Deseq2 1.28.1 and HOMER v4.11.1.
Bulk RNAseq was analyzed using TopHat 2.1.1 for alignment, Homer v4.11.1 for calling read counts and Deseq2 1.28.1 for differential analysis.
RTPCR data was analyzed using GraphPad Prism v8.2, 9.3 and 9.5.1.

For manuscripts utilizing custom algorithms or software that are central to the research but not yet described in published literature, software must be made available to editors and reviewers. We strongly encourage code deposition in a community repository (e.g. GitHub). See the Nature Portfolio guidelines for submitting code & software for further information.

Data

Policy information about availability of data

All manuscripts must include a data availability statement. This statement should provide the following information, where applicable:

- Accession codes, unique identifiers, or web links for publicly available datasets
- A description of any restrictions on data availability
- For clinical datasets or third party data, please ensure that the statement adheres to our policy

All data from this study will be made available for public access. Source data are provided with this paper. Patient data will remain de-identified. Bulk RNAseq, scRNAseq, next generation amplicon and whole genome sequencing data generated in this study have been deposited in the dbGaP database under accession code phs003271.v1, (http://www.ncbi.nlm.nih.gov/projects/gap/cgi-bin/study.cgi?study_id=phs003271.v1.p1). The bulk RNAseq, scRNAseq, next generation amplicon and whole genome sequencing data are available under restricted access via the dbGaP database, which ensures that only authorized researchers working with appropriate approvals can access datasets derived from human patients. Access can be obtained by following instructions available at http://www.ncbi.nlm.nih.gov/projects/gap/cgi-bin/study.cgi?study_id=phs003271.v1.p1.

Research involving human participants, their data, or biological material

Policy information about studies with human participants or human data. See also policy information about sex, gender (identity/presentation), and sexual orientation and race, ethnicity and racism.

Reporting on sex and gender	Patient material used in this study has been provided de-identified. Karyotyping revealed that of 4 patients whose cells were used in this study, 2 are genetically male and 2 are genetically female, with no other karyotypic abnormalities. This reflects a balanced distribution of gender for this study.
Reporting on race, ethnicity, or	Patient material used in this study has been provided de-identified. Thus, no report on race, ethnicity, or other socially

other socially relevant groupings	relevant groupings can be made.
Population characteristics	Patient material used in this study has been provided de-identified. Thus, no information about age and treatment categories is available. Genotypes causing RDEB are disclosed in Figure 1 and Supplementary Figure 5 and associated text. Whole genome sequencing data will be made available in a de-identified manner as described above. The only diagnosis information available is that patients whose material was used in this study suffer from severe RDEB.
Recruitment	To establish reproducibility of results, 3 individuals carrying the same RDEB-causing mutation of COL7A1 were picked from patient registries at Stanford University and University of Colorado. As the RDEB-causing mutation spectrum spans the entire 31kb COL7A1 gene, individuals carrying the same genetic pathogenicity are extremely rare. Thus, patient cohorts from 2 clinics had to be used to identify 3 individuals carrying the same mutation. To establish adaptability of our approach, 1 individual carrying another RDEB-causing mutation of COL7A1 was randomly chosen from the patient registry at Stanford University. All patients provided consent for de-identified use of their biological material.
Ethics oversight	Stanford University approved this study via SCRO protocol 691. Associated IRBs are: IRB-45005, IRB-22237 and IRB-22005.

Note that full information on the approval of the study protocol must also be provided in the manuscript.

Field-specific reporting

Please select the one below that is the best fit for your research. If you are not sure, read the appropriate sections before making your selection.

[x] Life sciences [ ] Behavioural & social sciences [ ] Ecological, evolutionary & environmental sciences

For a reference copy of the document with all sections, see nature.com/documents/nr-reporting-summary-flat.pdf

Life sciences study design

All studies must disclose on these points even when the disclosure is negative.

Sample size	Individuals carrying the same RDEB-causing mutation of COL7A1 are extremely rare. Nonetheless, this study succeeded in recruiting 3 individuals carrying the same genetic pathogenicity. Our approach was successfully applied to cells from all 3 participants, establishing reproducibility via 3 biological replicates. All 1-step edited/reprogrammed iPSC lines derived from these 3 individuals were analyzed for correction of the pathogenic mutation (i.e. sample size was determined by yield of reprogramming). For extensive and cost-intense downstream analysis and production of iSC grafts (e.g. whole genome sequencing, scRNAseq and grafting with associated efficacy, biodistribution and tumorgenicity studies), 1-2 iPSC cell lines were randomly chosen from each patient.
Data exclusions	No data was excluded from the study.
Replication	Our study uses material from 3 RDEB patients carrying the same pathogenic mutation. Results were reproducible with all 3 biological replicates. Adaptability of our approach for other RDEB-causing mutations was established via a fourth patient carrying a different mutation.
Randomization	Randomization is not relevant to this deterministic study as it is designed to repair specific pathogenic mutations of rare individuals in order to produced a defined therapeutic product.
Blinding	Blinding is not relevant to this deterministic study as it is designed to repair specific pathogenic mutations of rare individuals, producing an autologous therapeutic product.

Reporting for specific materials, systems and methods

We require information from authors about some types of materials, experimental systems and methods used in many studies. Here, indicate whether each material, system or method listed is relevant to your study. If you are not sure if a list item applies to your research, read the appropriate section before selecting a response.

Materials & experimental systems

n/a	Involved in the study	
[ ]	[x]	Antibodies
[ ]	[x]	Eukaryotic cell lines
[x]	[ ]	Palaeontology and archaeology
[ ]	[x]	Animals and other organisms
[x]	[ ]	Clinical data
[x]	[ ]	Dual use research of concern
[x]	[ ]	Plants

Methods

n/a	Involved in the study	
[x]	[ ]	ChIP-seq
[ ]	[x]	Flow cytometry
[x]	[ ]	MRI-based neuroimaging

Antibodies

Antibodies used	Antibody, Company, Catalog number, lot number, used dilution:

p63, Gene Tex, GTX102425, 41143, 1:100
K10, Covance, PRB-159P, D12CF00418, 1:500
K14, BioLegend, SIG-3476-100, B350648, 1:800
K14, Covance, PRB-155P, no lot number, 1:2000
FITC-K14, Millipore, CBL197F, 3022864, 1:100
K18, R&D, AF7619, CGRO0119031, 1:800
K18, Cell Signaling Technology, 4548, lot3, 1:400
K18, Novus, NB120-7797, no lot number, 1:100
Involucrin, abcam, ab27495, 855282, 1:100
ITGA6, Milteny, REA518, 5181228167, 1:50
ITGA6, BD, 555736, B289793, 1:100
ITGA6, Millipore, MAB1378, 2251733, 1:200
ITGB4, BD, 744150, 2018381, 1:100
COL7(LH7.2), Millipore, MAB1345, 3792153, 1:250
CD90, BD, 555595, 1340966, 1:100
Labeling Check Reagent-FITC, Miltenyi Biotec , 130-099-136, 5210408850, 1:50
Streptavidin, Life Technologies, S32357, 1985403, 1:500
Human Nuclear Antigen, Thermo Fisher, RBM5-346-P1, RBM5-346P230710, 1:200
Vimentin, Abcam, ab8979, 1017416-1, 1:100
CD104, Thermo Fisher, 14-1049-82, 2559387, 1:50
TRA-1-81, Sigma Aldrich , MAB4381, NG1853948, 1:1000
TRA-1-60, Sigma Aldrich , MAB4360, 3158074, 1:1000
NANOG, Abcam, ab21624, GR3198376-10, 1:2000

Validation

All used antibodies are commercially available and widely used by the field. Associated references can be found via the following RRIDs at https://antibodyregistry.org/. Antibody, Company, RRID:

p63, Gene Tex, AB_1952344
K10, Covance, AB_291580
K14, BioLegend, AB_2616962
K14, Covance, AB_292096
FITC-K14, Millipore, AB_93327 & AB_11212136
K18, Cell Signaling Technology, AB_2296725
K18 , Novus, AB_790942 & AB_2133302
Involucrin, abcam, AB_470995
ITGA6, Milteny, AB_2658565
ITGA6, BD, AB_396079 & AB_493637
ITGA6, Millipore, AB_11210794 & AB_2128317
ITGB4, BD, AB_2742035
COL7(LH7.2), Millipore, AB_11210494 & AB_2083257
CD90, BD, AB_395969
Labeling Check Reagent-FITC, Miltenyi Biotec , AB_2661565
Vimentin, Abcam, AB_306908
CD104, Thermo Fisher, AB_1210460
TRA-1-81, Sigma Aldrich , AB_177638
TRA-1-60, Sigma Aldrich , AB_2119183
NANOG, Abcam, AB_446437

Eukaryotic cell lines

Policy information about cell lines and Sex and Gender in Research

Cell line source(s)

De-identified primary patient fibroblasts were derived from skin biopsies as described and named after the anonymous patient ID (i.e. CO1, CO2, DEB125, DEB135). Sex was determined via karyotyping of primary fibroblast-derived iPS cell lines (CO1: female , CO2: female, DEB125: male , DEB135: male). RDEB patient-derived SCC cells have been described previously (Jackow J et al. Targeting the Jak/Signal Transducer and Activator of Transcription 3 Pathway with Ruxolitinib in a Mouse Model of Recessive Dystrophic Epidermolysis Bullosa-Squamous Cell Carcinoma. J Invest Dermatol 2021;141(4):942-946. DOI: 10.1016/j.jid.2020.08.022). WA09 (H9) hESCs cells were obtained from WiCell, Wisconsin. TERT-KCs (aka N/TERT-1) were gifted from Jim Rheinwald and Ray Konger at IU-Indianapolis. N/TERT-1 is a TERT-immortalized human keratinocyte line that was derived from the primary human newborn foreskin epidermal keratinocyte line named "strain N". It was made using a retroviral vector that also carried a hygromycin-resistance gene and was described previously (Dickson, Mark A., et al. "Human keratinocytes that express hTERT and also bypass a p16INK4a-enforced mechanism that limits life span become immortal yet retain normal growth and differentiation characteristics." Molecular and cellular biology 20.4 (2000): 1436-1447). Primary NHKs were derived from discarded and de-identified foreskin neonatal tissue from Stanford Hospital (sex was not determined for NHKs). WTC-11 iPS cells (also known as UCSFi001-A and GM25256) are described at https://hpscreg.eu/cell-line/UCSFi001-A and are from a male donor; DSP iPS cells were obtained from Coriell and are described here https://www.coriell.org/0/Sections/Search/Sample_Detail.aspx?Ref=AICS-0017&Product=iPSC.

Authentication

De-identified primary patient fibroblasts and thereof derived iPS cell lines were authenticated via confirming their pathogenic mutation, or the edit of the pathogenic mutation, through sequencing analysis. Additional authentication of patient-derived

	iPS cell lines included assays ensuring clonality, pluripotency, and chromosomal and genomic stability (see paper). Please see above references for authentication of other used cell lines.
Mycoplasma contamination	All cell lines tested negative for mycoplasma contamination.
Commonly misidentified lines (See ICLAC register)	No commonly misidentified cell lines were used in this study. TERT-KCs are also known as N/TERT-1 cells. WTC-11 iPS cells are also known as UCSFi001-A and GM25256. DSP iPS cells are also known as AICS-0017 or MONO-ALLELIC mEGFP-TAGGED DSP WTC iPSC LINE (TAG AT C-TERM).

Animals and other research organisms

Policy information about studies involving animals; ARRIVE guidelines recommended for reporting animal research, and Sex and Gender in Research

Laboratory animals	NOD-NSG mice were used for grafting, tumorgenicity and biodistribution assays. Teratoma assay used following mice: jax.org/strain/017708. Age of mice was not recorded for this study.
Wild animals	The study did not involve wild animals.
Reporting on sex	Sex-based analysis has not been performed on used laboratory mice. Our patient cohort included a balanced distribution of male and female participants and results were reproducible, indicating no influence of gender.
Field-collected samples	The study did not involve samples collected from the field.
Ethics oversight	Stanford University approved this study via SCRO protocol 691. Associated IRBs are: IRB-45005, IRB-22237 and IRB-22005.

Note that full information on the approval of the study protocol must also be provided in the manuscript.

Plants

Seed stocks	No plants were involved in the study.
Novel plant genotypes	No plants were involved in the study.
Authentication	No plants were involved in the study.

Flow Cytometry

Plots

Confirm that:

- [x] The axis labels state the marker and fluorochrome used (e.g. CD4-FITC).
- [x] The axis scales are clearly visible. Include numbers along axes only for bottom left plot of group (a 'group' is an analysis of identical markers).
- [x] All plots are contour plots with outliers or pseudocolor plots.
- [x] A numerical value for number of cells or percentage (with statistics) is provided.

Methodology

Sample preparation	Dissociated cells were washed with FACS buffer (2% BSA Cat# 130-091-376 containing 1μM ROCK inhibitor). After wash steps, cells were fixed and permeabilized with (eBioscience™ Intracellular Fixation & Permeabilization Buffer Set, Cat # 88-8824-00) then stained for antibodies of interest for 30 min at 4°C (FITC anti-K14, CBL197F from Millipore; PerCP anti-K18 (NB120-7797, Novus), ITGA6 (PE anti-CD49F BD Cat #555736), all at 1:100 dilution. Enriched iSCs were analyzed using Streptavidin Alexa Fluor™ 647 conjugate (Life Technologies Cat # S32357, 1:500 dilution) and FITC-Labeling Reagent (Miltenyi Biotec, Cat # 130-099-136, 1:50 dilution). Composition analysis of enriched iSCs was performed using anti-ITGB4 (BD Cat # 744150, 1:100 dilution) and anti-CD90 (BD Cat # 555595, 1:100 dilution).
Instrument	Data was acquired on a BD LSRII in the Stanford Shared FACS Facility with BD FACSDiva Software.
Software	Analysis was performed with BD FACSDiva and FlowJo software.
Cell population abundance	Abundance of post-sort target cell populations is described in the relevant Figures with associated text.

Gating strategy

Gating strategies are described in the manuscript. Using Flowjo analysis software to analyze the composition of the cell population, FSC-A/SSC-A gating was first used to identify cells from debris, then gated for singlets by FSC-W/FSC-H, followed by X and Y axis assignment of epitopes as indicated.

Figure 5E: Using Flowjo analysis software to analyze the composition of the cell population, FSC-A/SSC-A gating was first used to identify cells from debris, then gated for singlets by FSC-W/FSC-H, followed by X and Y axis assignment of ITGB4/CD104 and THY1/CD90. The ITGB4 + population identifies the keratinocytes while the ITGB4 -/ CD90 + identifies the fibroblast population. The boundary of the positive and negative contour plots was drawn based on both positive control keratinocyte cell lines and unstained controls.

Supplementary Figure 6G: Using Flowjo analysis software to analyze CliniMACS enrichment of induced keratinocytes, FSC-A/SSC-A gating was first used to identify cells from debris, then gated for singlets by FSC-W/FSC-H, followed by X and Y axis assignment of markers for double positive surface binding of the primary ITGA6/CD49F REA518 biotin antibody by Streptavidin 647 and Anti-biotin microbead that is labeled with a Labeling Check reagent 488. The boundary of the positive and negative gates was drawn based on both positive control keratinocyte cell lines and unstained controls.

☐ Tick this box to confirm that a figure exemplifying the gating strategy is provided in the Supplementary Information.
