# Supplementary figures and images for: A scalable and cGMP-compatible autologous organotypic cell therapy for Dystrophic Epidermolysis Bullosa (part 3 of 3)
Source: Nat Commun. 2024 Jul 11;15:5834. doi: 10.1038/s41467-024-49400-z (PMC11239819; doi:10.1038/s41467-024-49400-z)

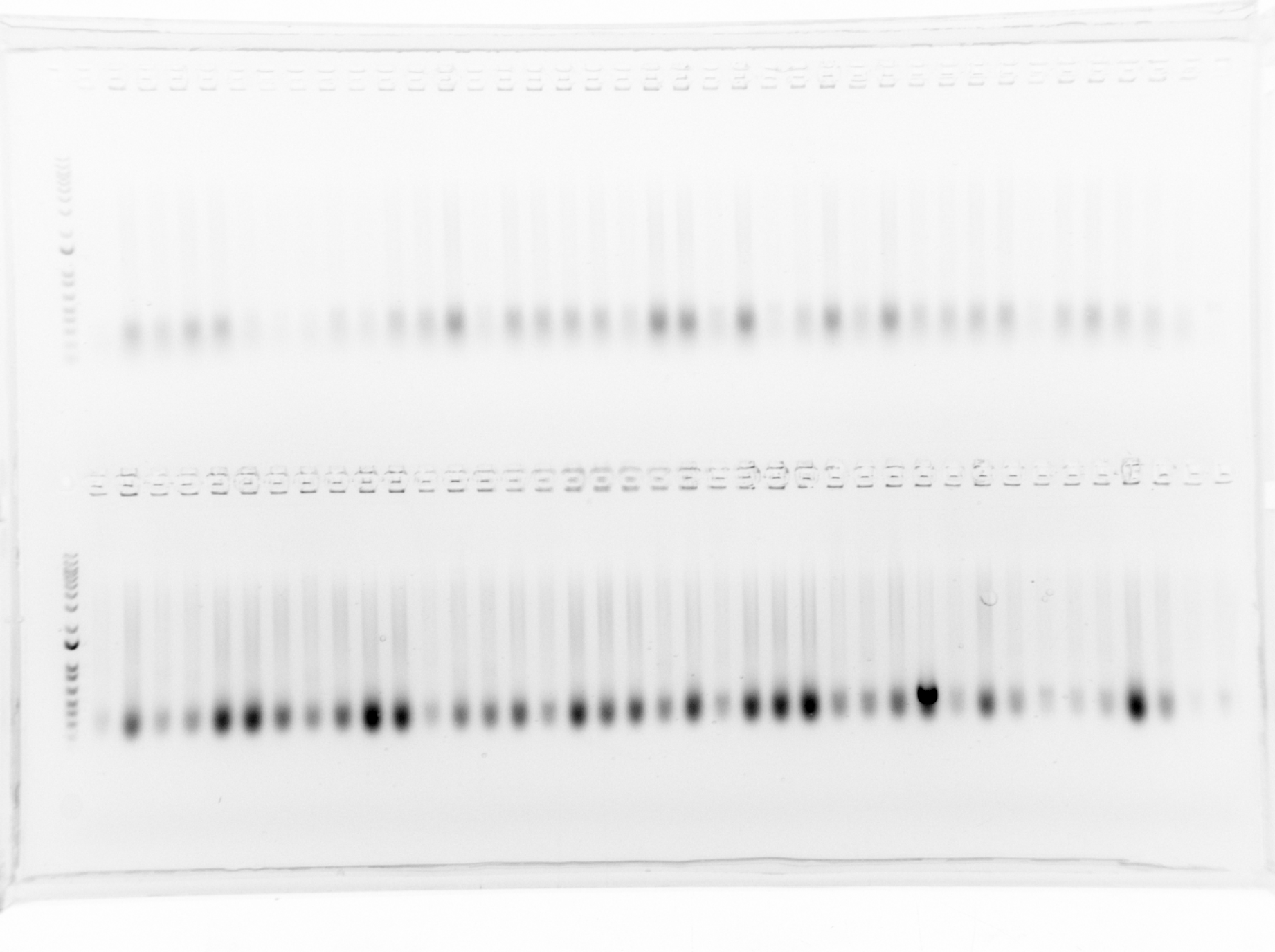

Supplement: Supplementary file 8 — Source Data [file 41467_2024_49400_MOESM8_ESM.zip › Source Data/Figure 1/Figure 1F/Figure 1F bottom.tif]

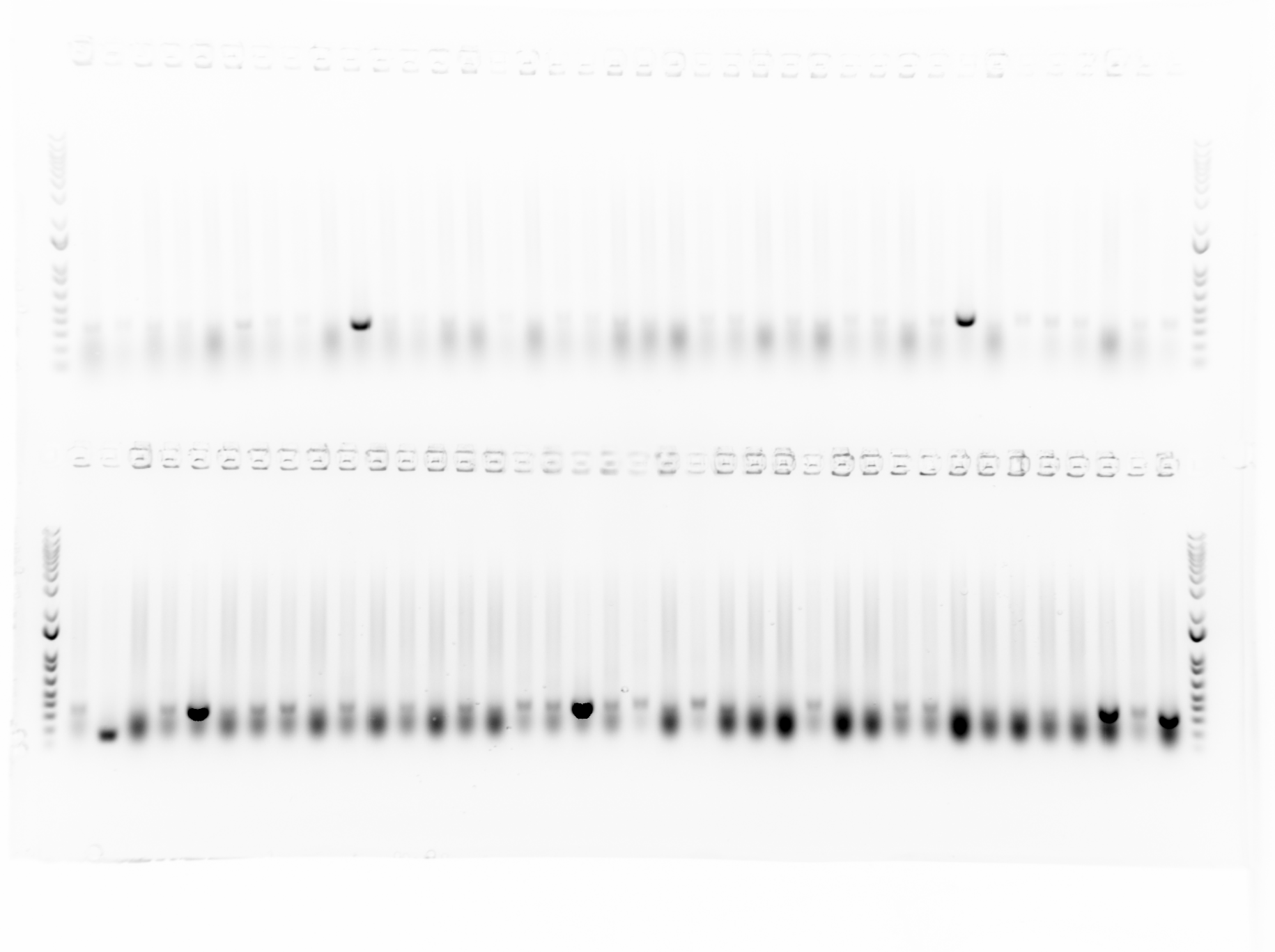

Supplement: Supplementary file 8 — Source Data [file 41467_2024_49400_MOESM8_ESM.zip › Source Data/Figure 1/Figure 1F/Figure 1F top.tif]

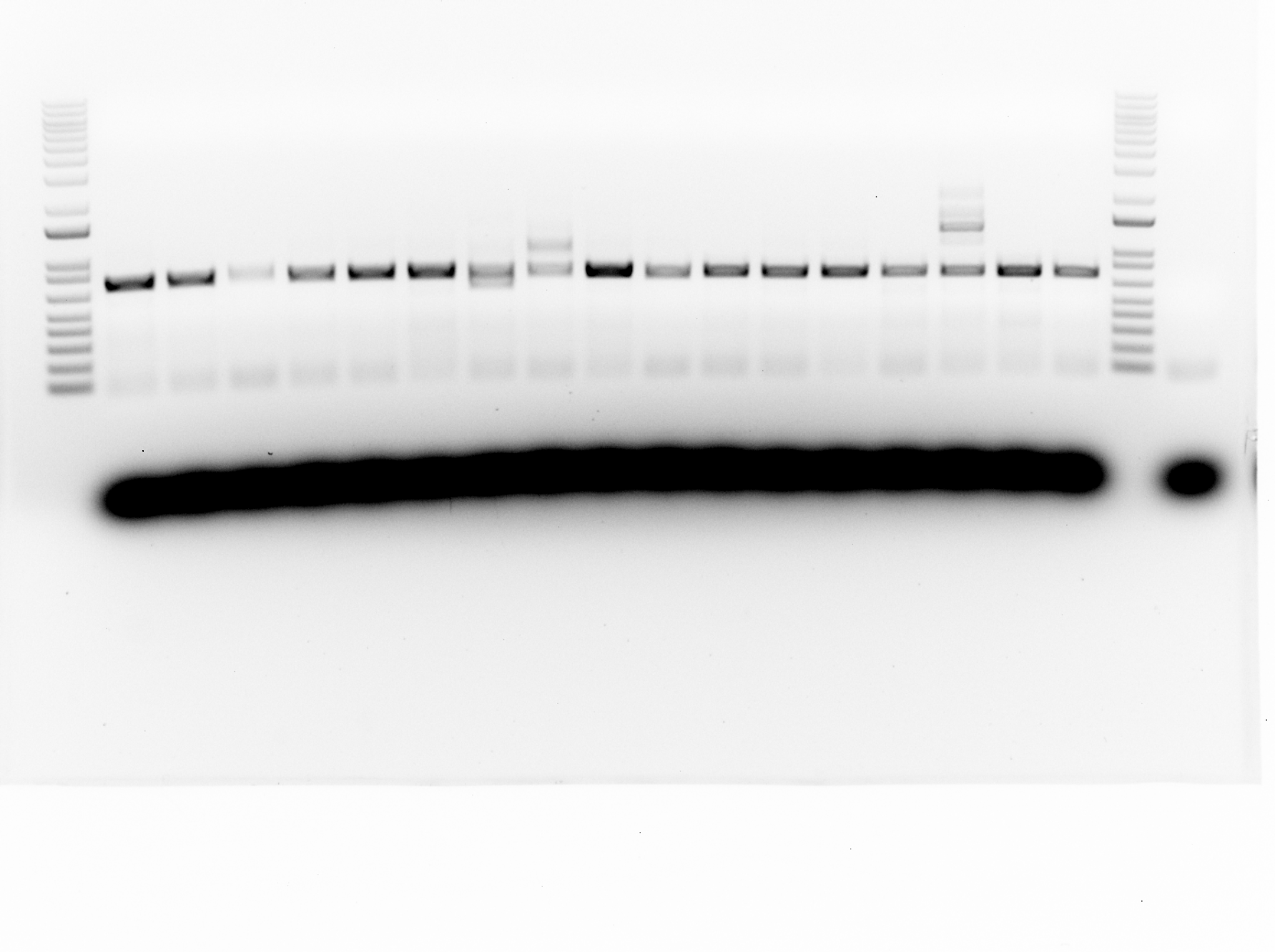

Supplement: Supplementary file 8 — Source Data [file 41467_2024_49400_MOESM8_ESM.zip › Source Data/Figure 2/Figure 2D/2D.tif]

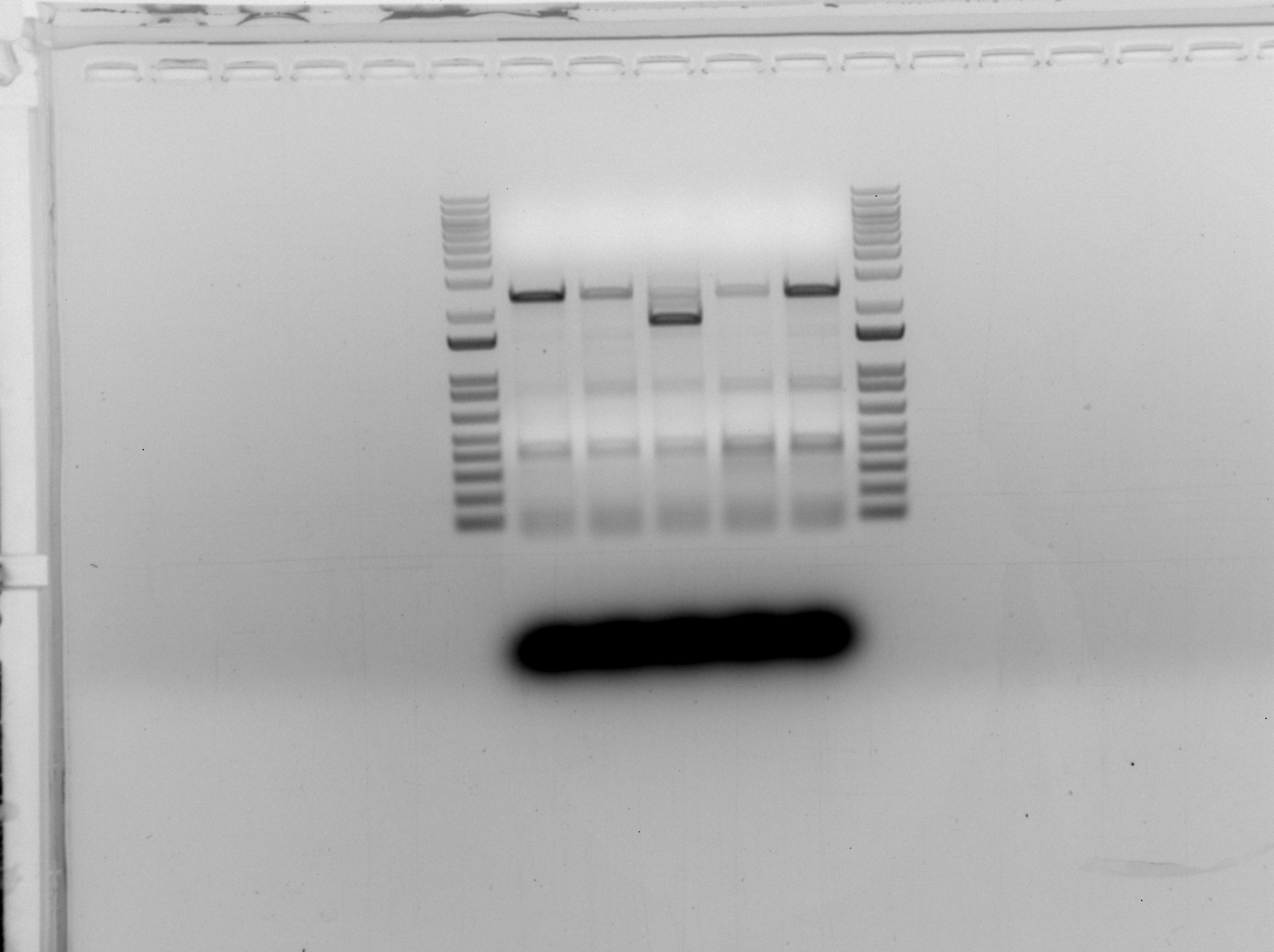

Supplement: Supplementary file 8 — Source Data [file 41467_2024_49400_MOESM8_ESM.zip › Source Data/Figure 2/Figure 2E/Figure 2E.tif]

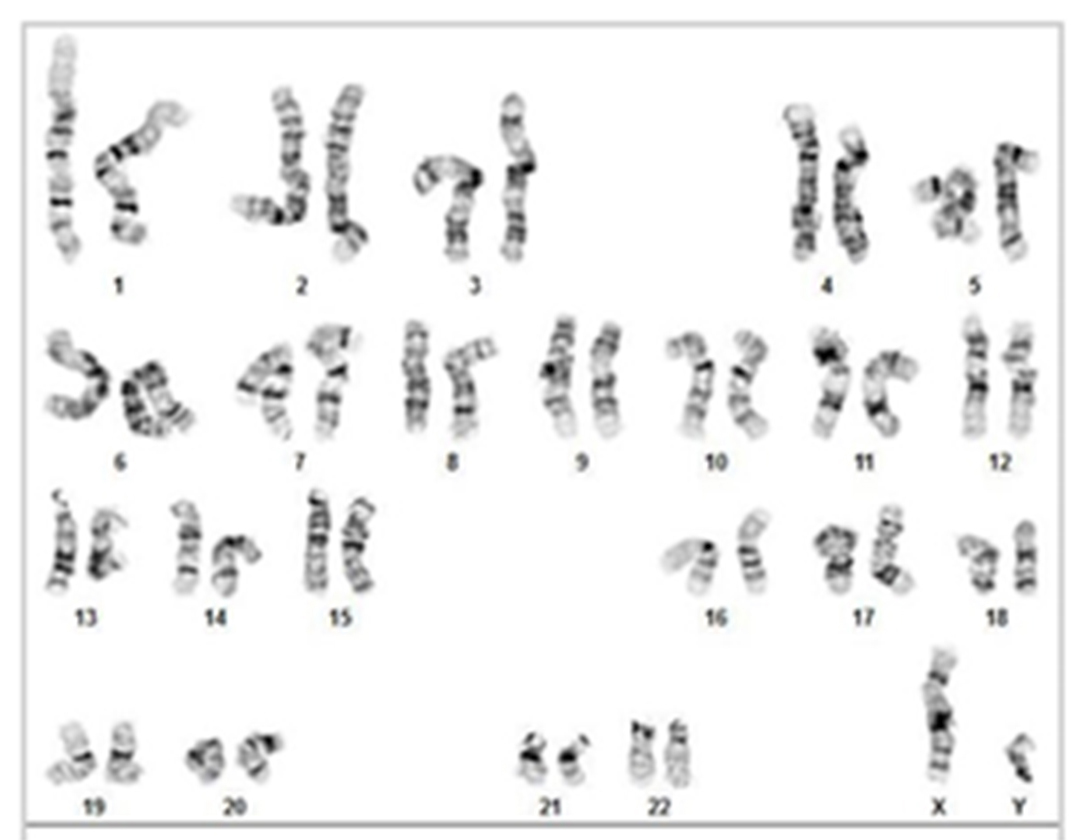

Supplement: Supplementary file 8 — Source Data [file 41467_2024_49400_MOESM8_ESM.zip › Source Data/Figure 4/Figure 4A/135-10.jpg]

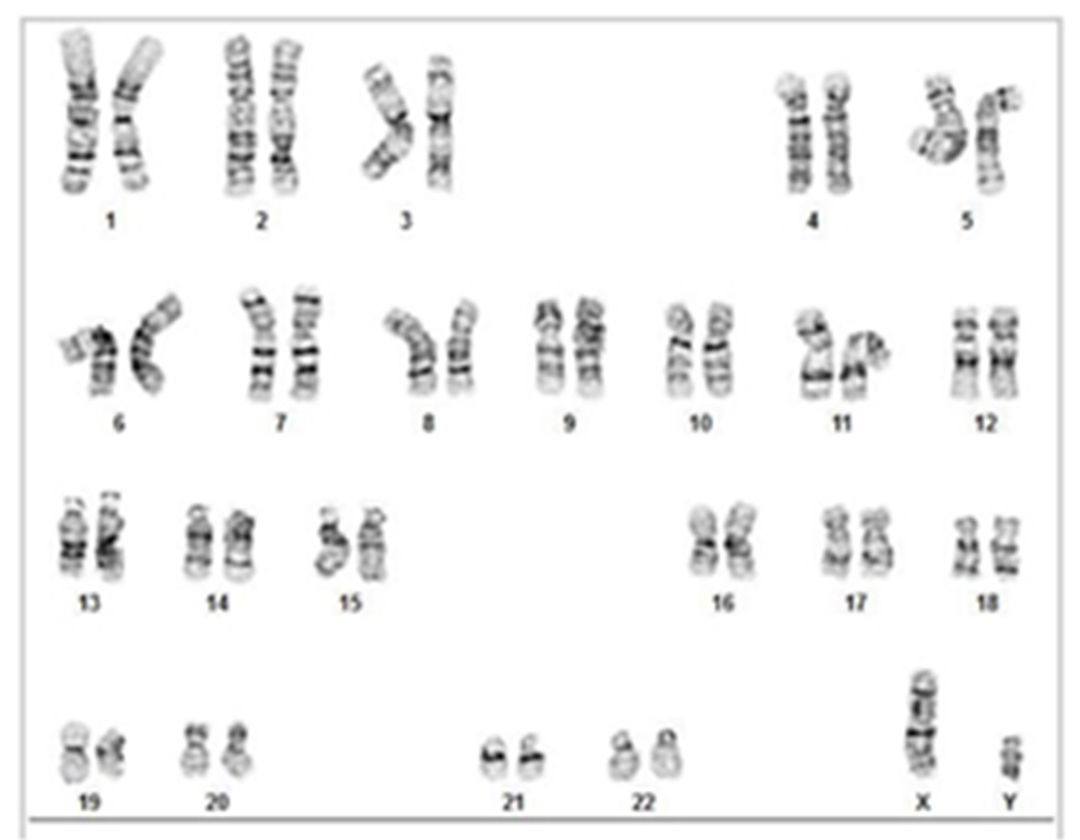

Supplement: Supplementary file 8 — Source Data [file 41467_2024_49400_MOESM8_ESM.zip › Source Data/Figure 4/Figure 4A/135-24.jpg]

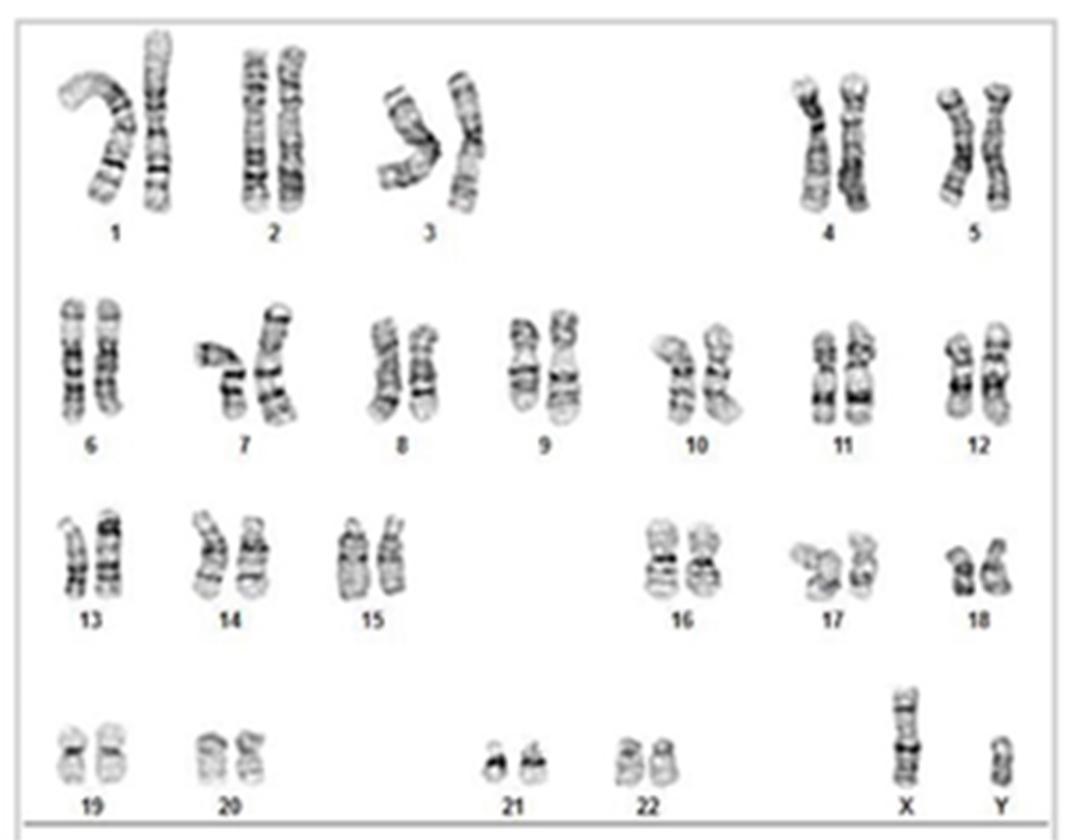

Supplement: Supplementary file 8 — Source Data [file 41467_2024_49400_MOESM8_ESM.zip › Source Data/Figure 4/Figure 4A/135-S21.jpg]

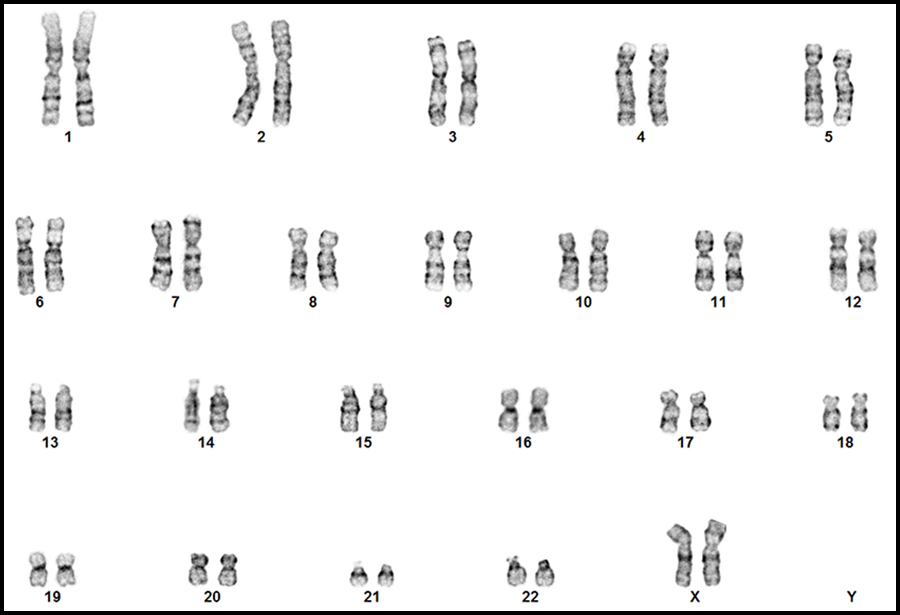

Supplement: Supplementary file 8 — Source Data [file 41467_2024_49400_MOESM8_ESM.zip › Source Data/Figure 4/Figure 4A/C02-65B.tif]

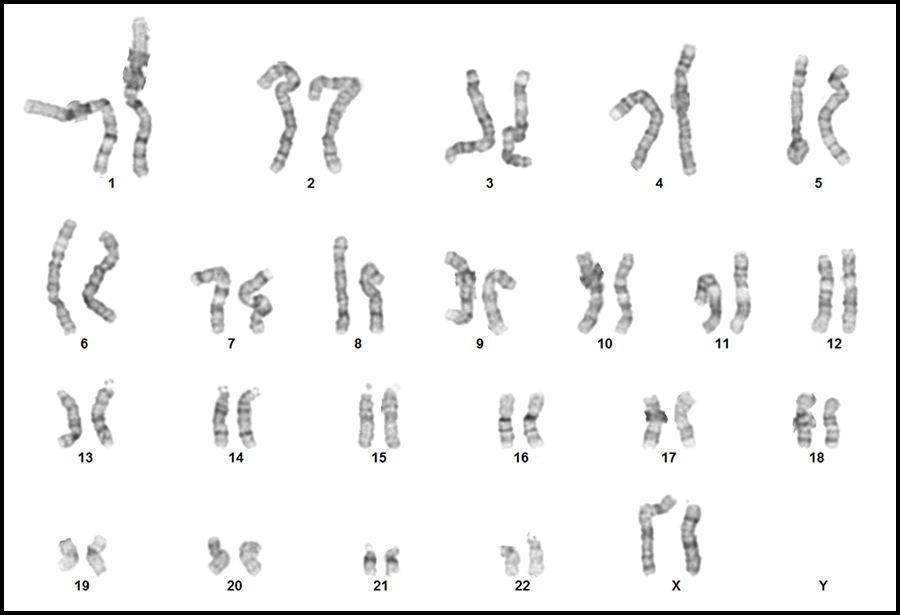

Supplement: Supplementary file 8 — Source Data [file 41467_2024_49400_MOESM8_ESM.zip › Source Data/Figure 4/Figure 4A/CO1-131.tif]

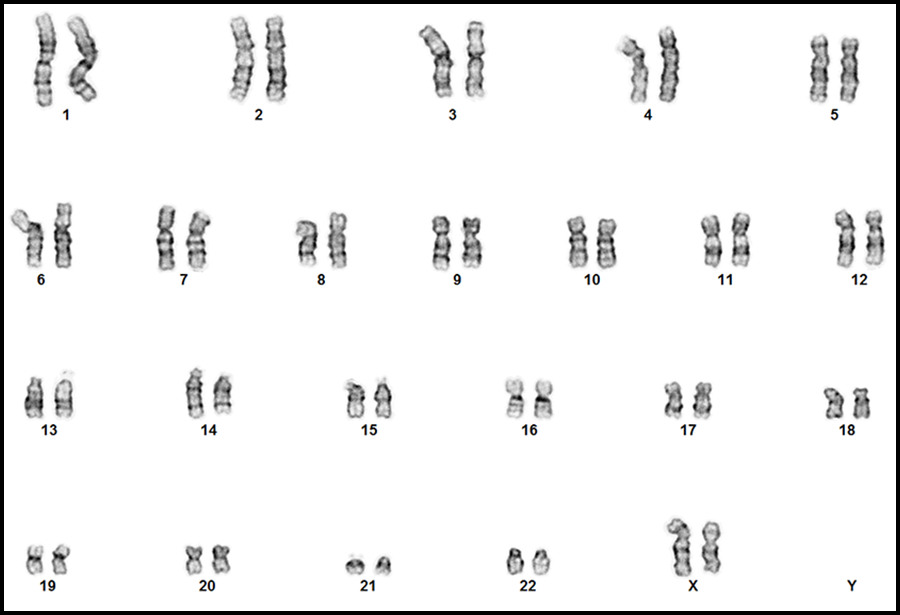

Supplement: Supplementary file 8 — Source Data [file 41467_2024_49400_MOESM8_ESM.zip › Source Data/Figure 4/Figure 4A/CO1-173.tif]

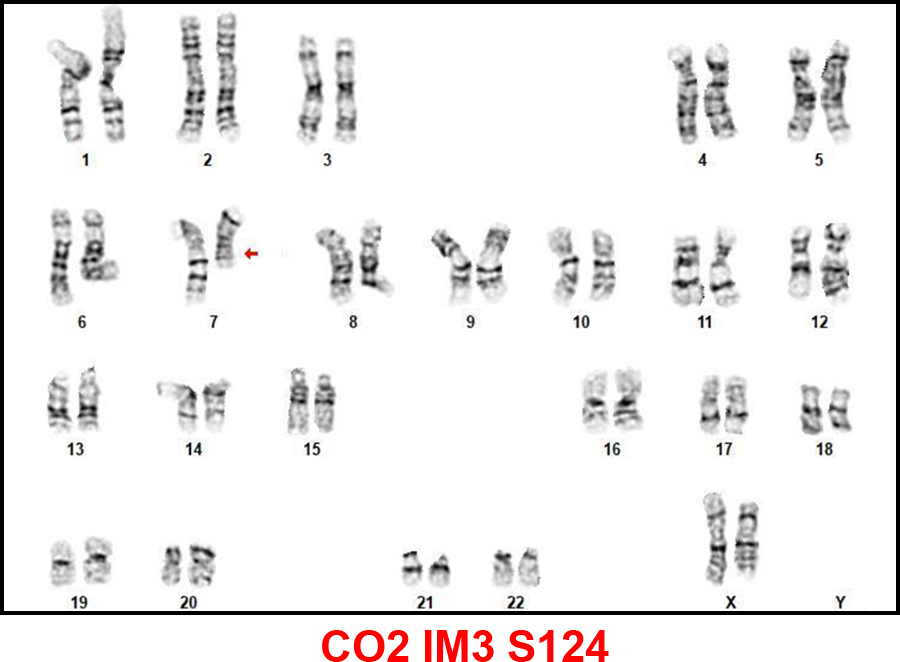

Supplement: Supplementary file 8 — Source Data [file 41467_2024_49400_MOESM8_ESM.zip › Source Data/Figure 4/Figure 4A/CO2-124C.jpg]

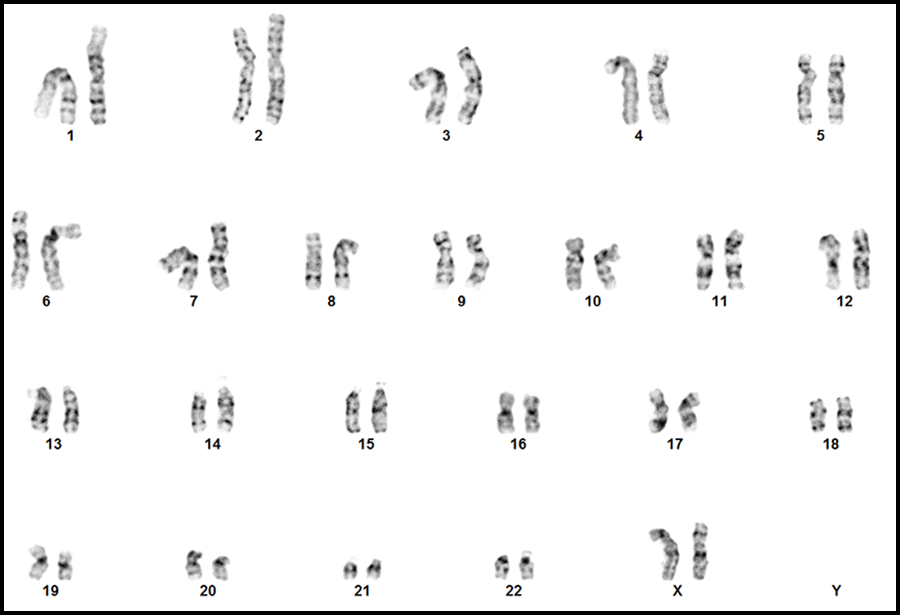

Supplement: Supplementary file 8 — Source Data [file 41467_2024_49400_MOESM8_ESM.zip › Source Data/Figure 4/Figure 4A/CO2-36A.tif]

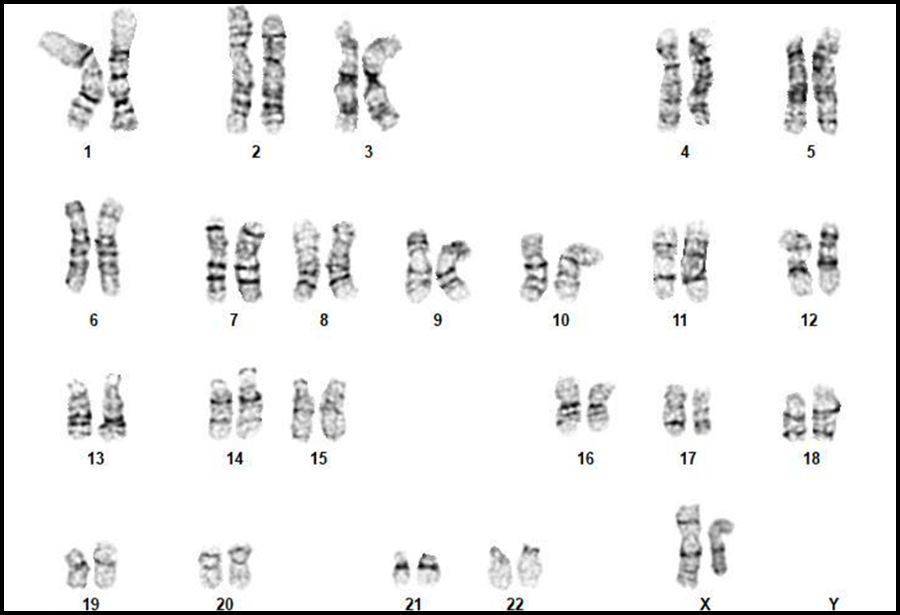

Supplement: Supplementary file 8 — Source Data [file 41467_2024_49400_MOESM8_ESM.zip › Source Data/Figure 4/Figure 4A/CO2-48C.tif]

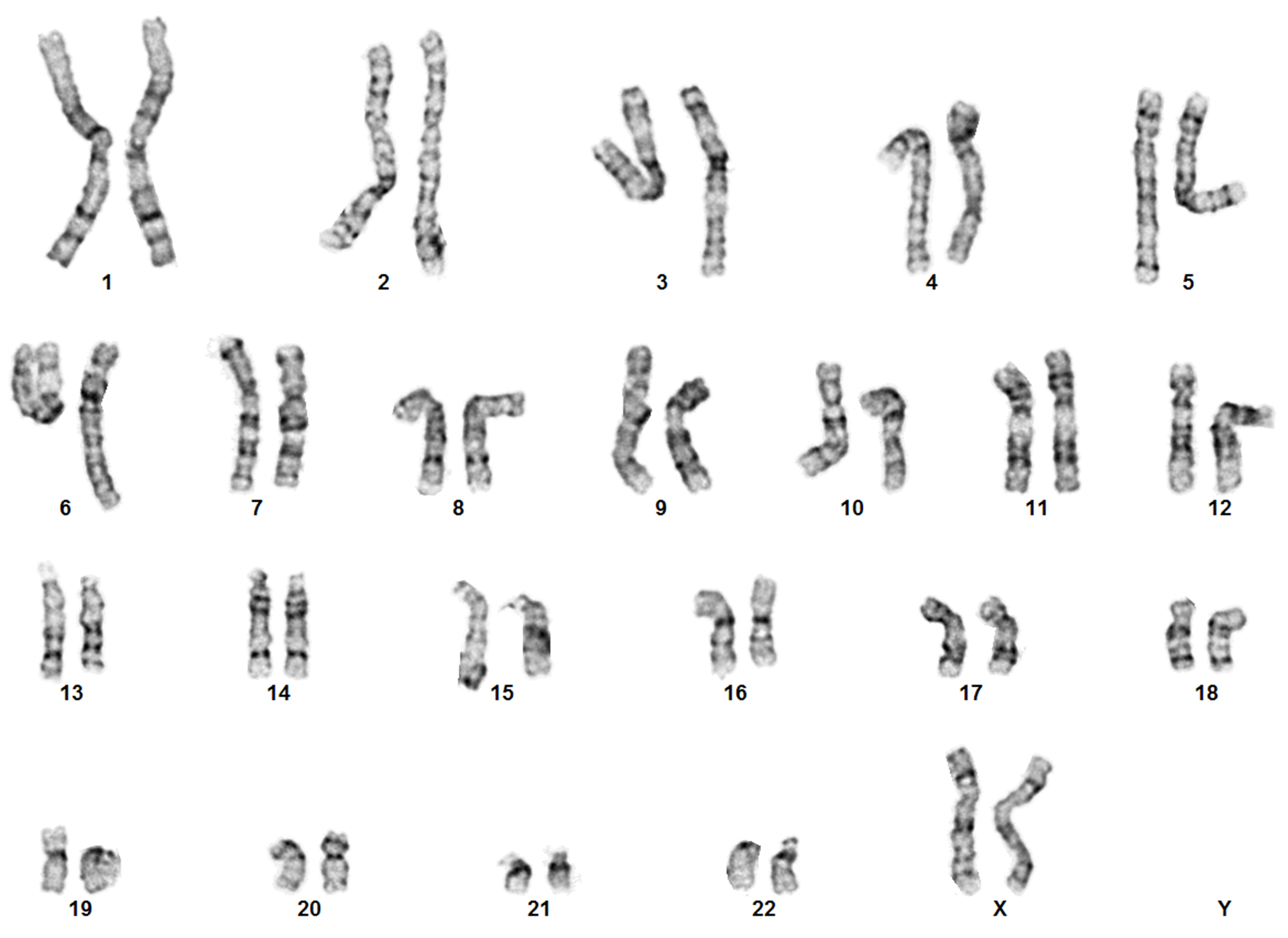

Supplement: Supplementary file 8 — Source Data [file 41467_2024_49400_MOESM8_ESM.zip › Source Data/Figure 4/Figure 4A/CO2-OS25.jpg]

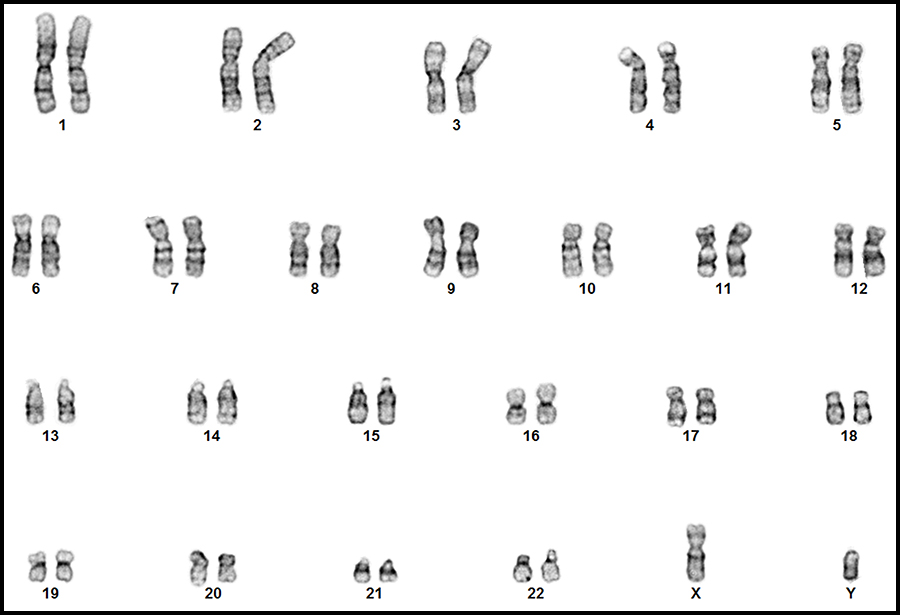

Supplement: Supplementary file 8 — Source Data [file 41467_2024_49400_MOESM8_ESM.zip › Source Data/Figure 4/Figure 4A/DEB125-1.jpg]

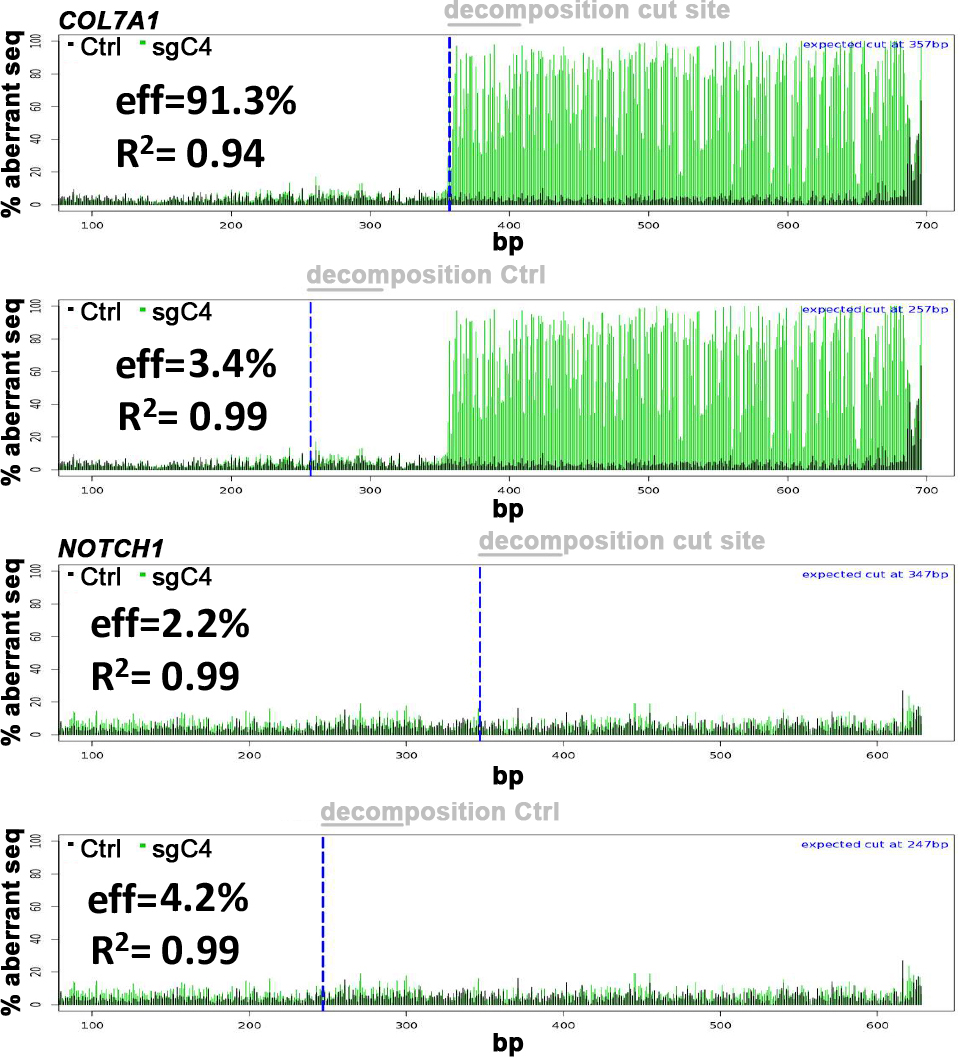

Supplement: Supplementary file 8 — Source Data [file 41467_2024_49400_MOESM8_ESM.zip › Source Data/Figure 4/Figure 4E/Figure 4E.jpg]

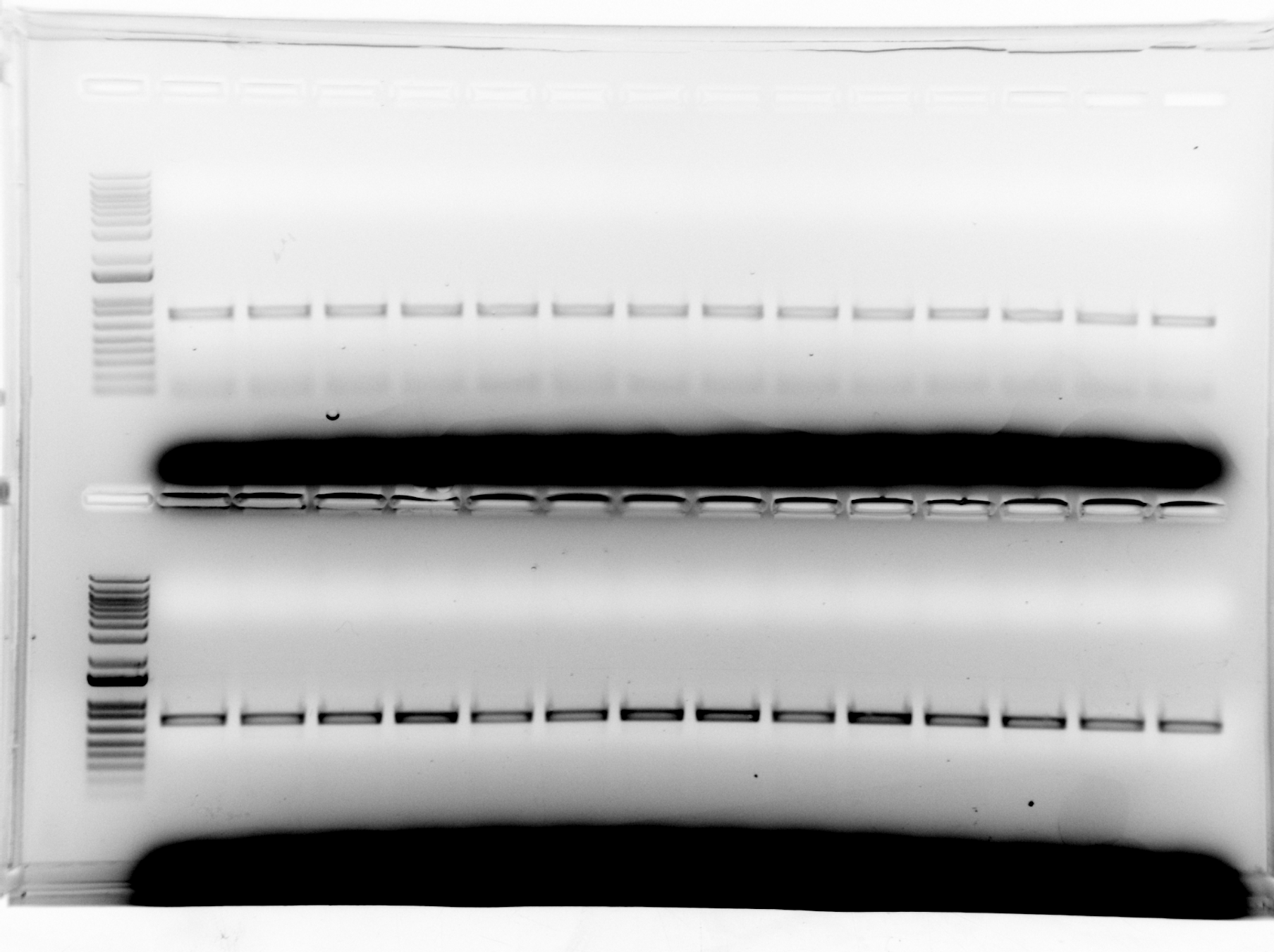

Supplement: Supplementary file 8 — Source Data [file 41467_2024_49400_MOESM8_ESM.zip › Source Data/Supplemental Figure 1/Supplemental Figure 1B/Supplemental Figure 1B.tif]

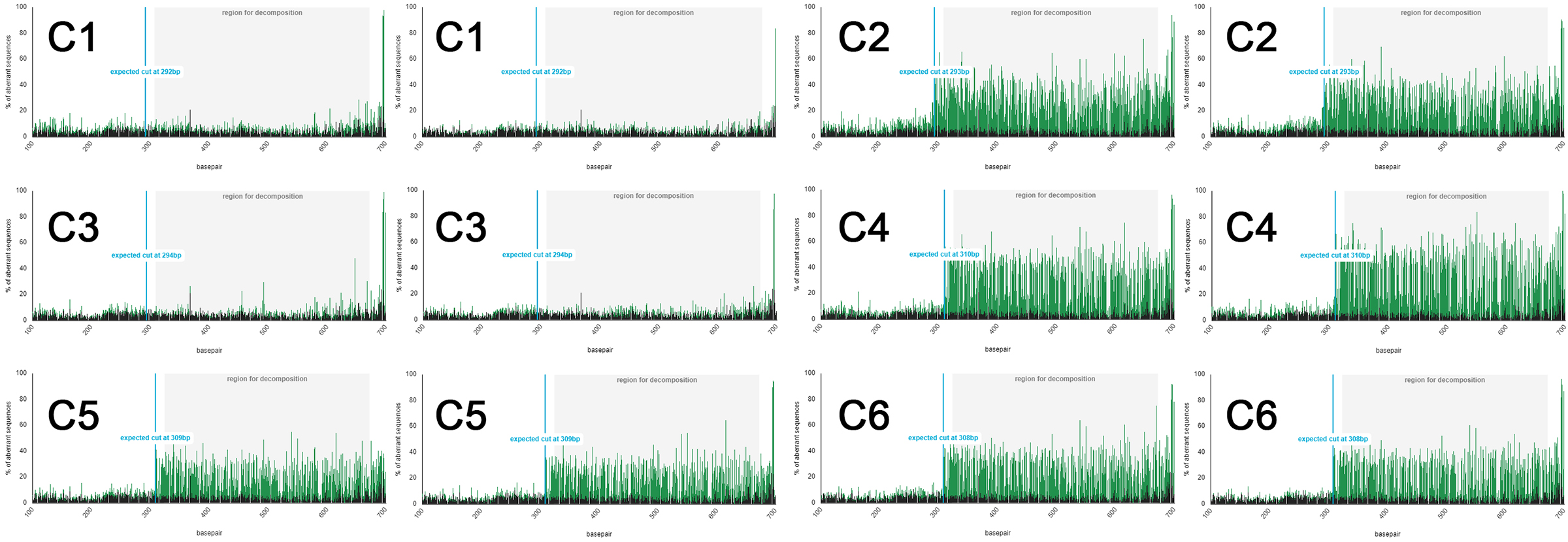

Supplement: Supplementary file 8 — Source Data [file 41467_2024_49400_MOESM8_ESM.zip › Source Data/Supplemental Figure 1/Supplemental Figure 1C/Supplemental Figure 1C.jpg]

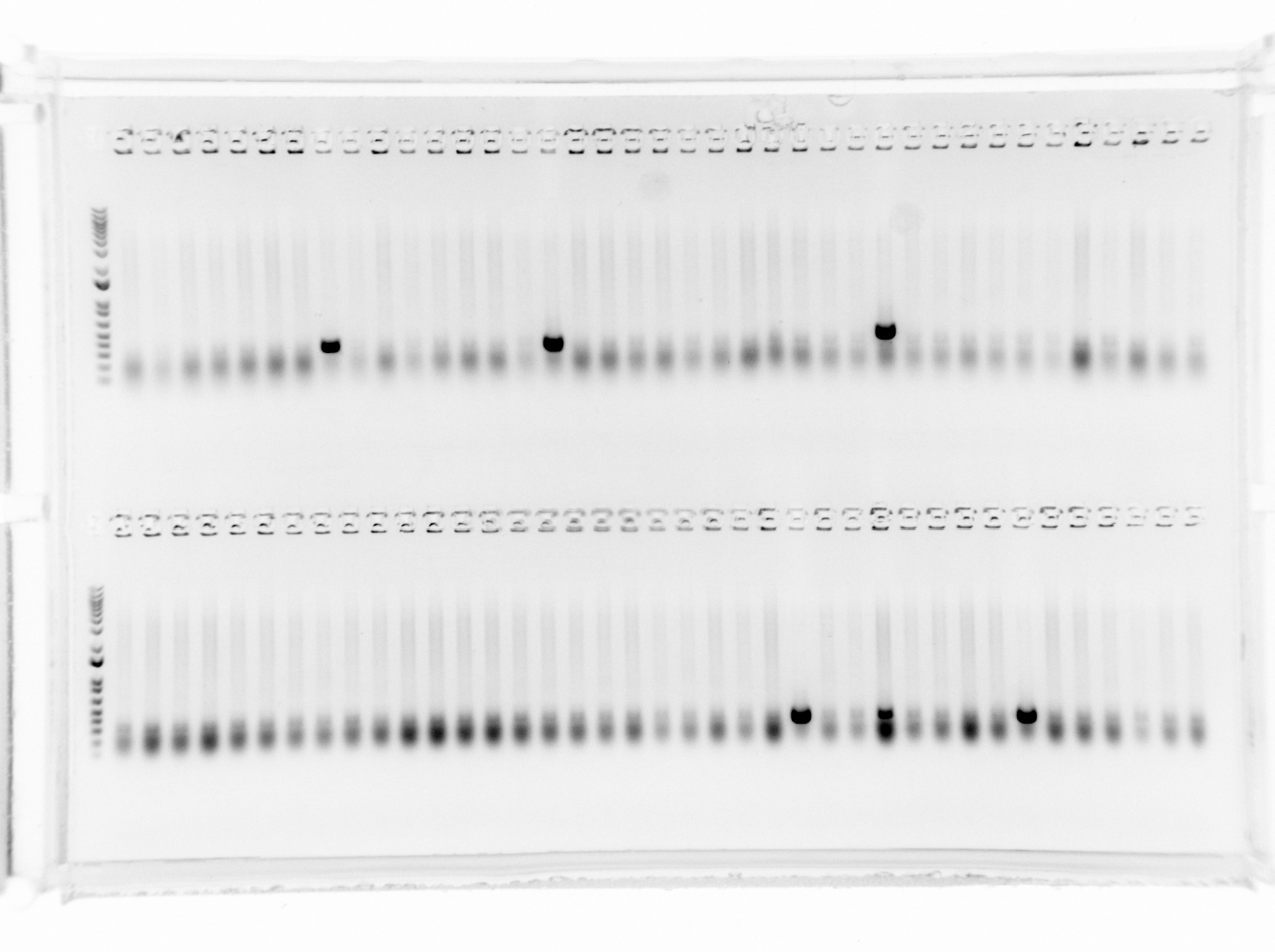

Supplement: Supplementary file 8 — Source Data [file 41467_2024_49400_MOESM8_ESM.zip › Source Data/Supplemental Figure 1/Supplemental Figure 1F/Supplemental Figure 1F.tif]

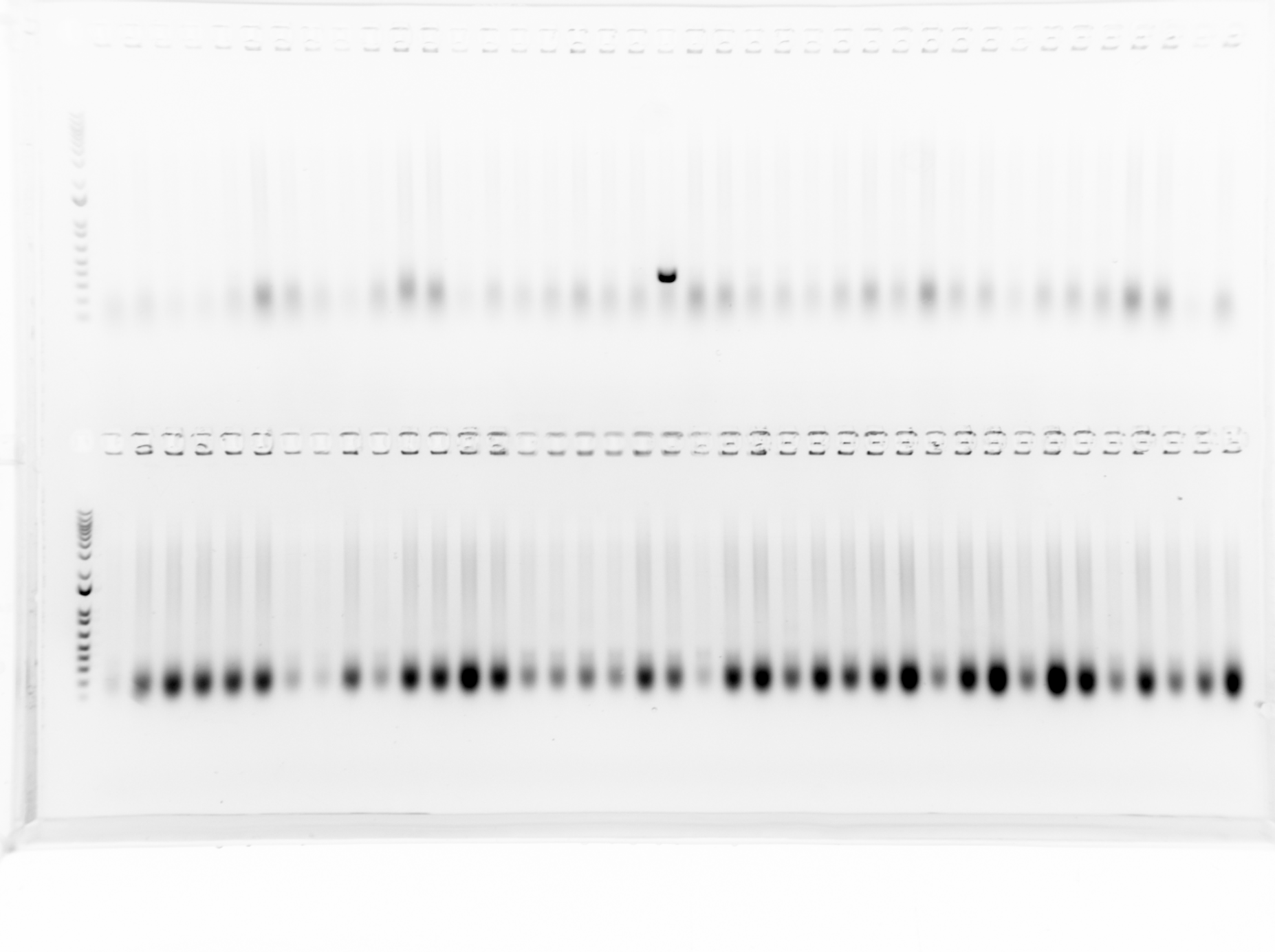

Supplement: Supplementary file 8 — Source Data [file 41467_2024_49400_MOESM8_ESM.zip › Source Data/Supplemental Figure 1/Supplemental Figure 1G/Supplemental Figure 1G.tif]

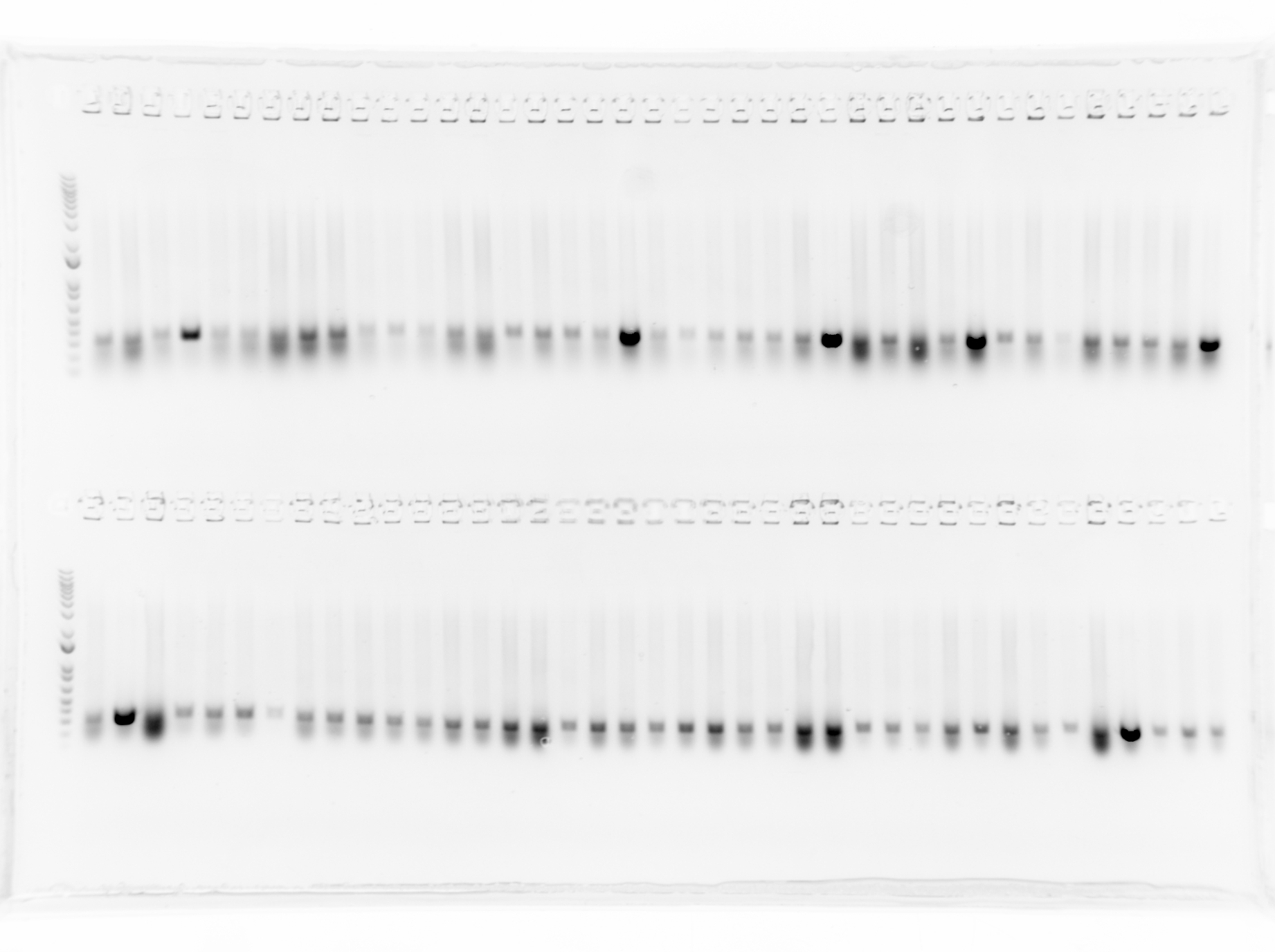

Supplement: Supplementary file 8 — Source Data [file 41467_2024_49400_MOESM8_ESM.zip › Source Data/Supplemental Figure 1/Supplemental Figure 1H/Supplemental Figure 1H.tif]

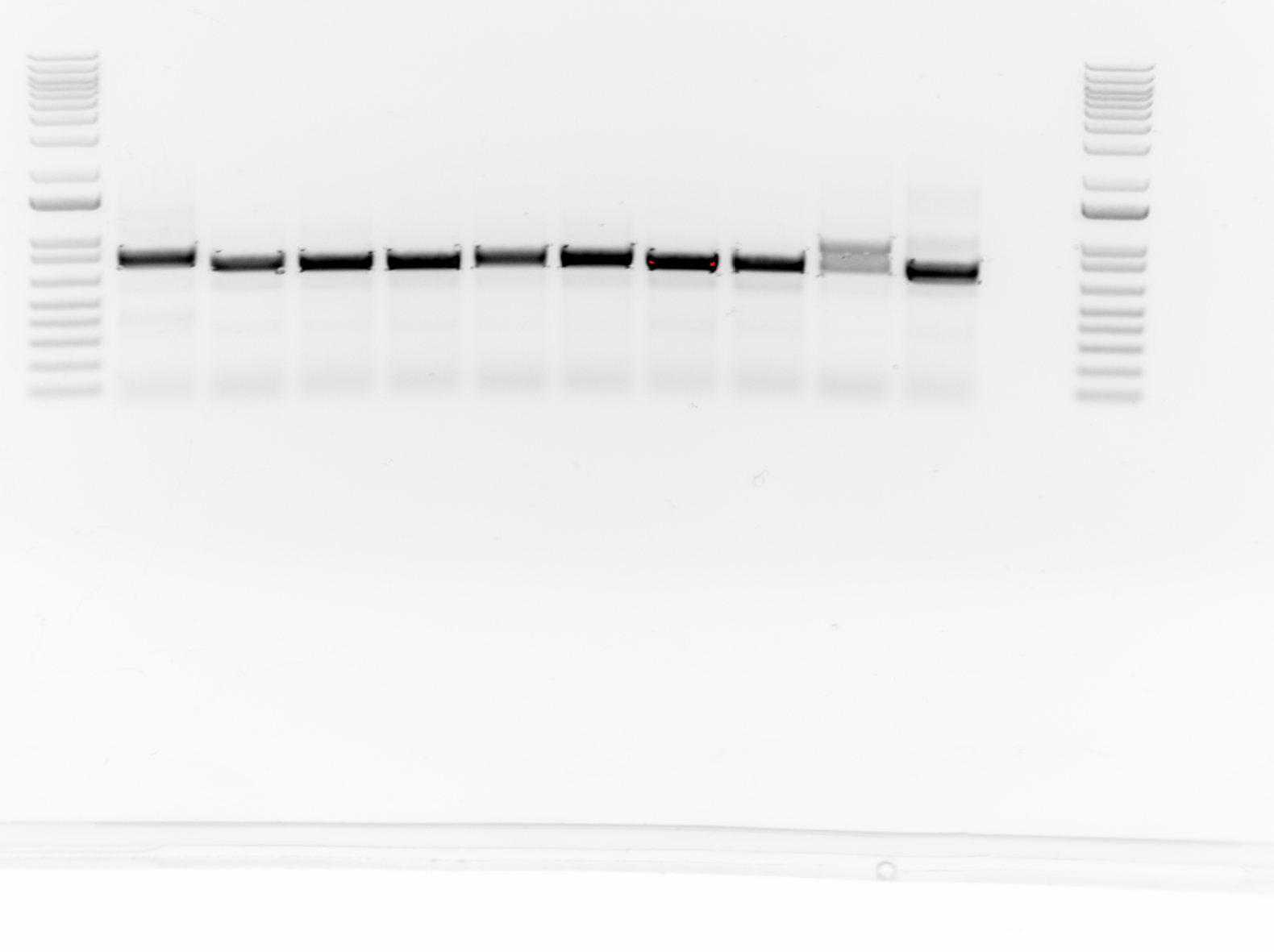

Supplement: Supplementary file 8 — Source Data [file 41467_2024_49400_MOESM8_ESM.zip › Source Data/Supplemental Figure 2/Supplemental Figure 2B/Supplemental Figure 2B.tif]

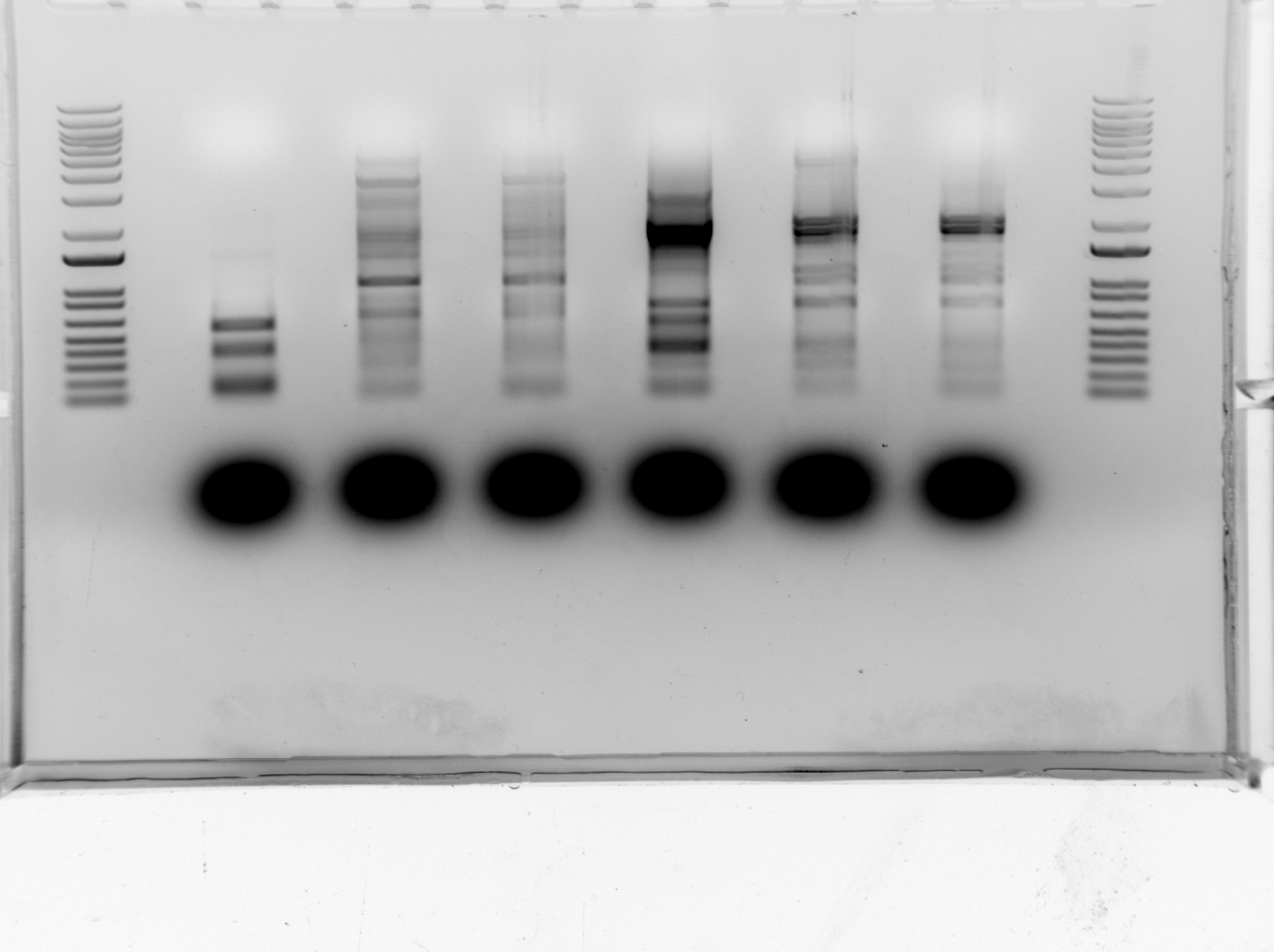

Supplement: Supplementary file 8 — Source Data [file 41467_2024_49400_MOESM8_ESM.zip › Source Data/Supplemental Figure 2/Supplemental Figure 2C/Supplemental Figure 2C.tif]

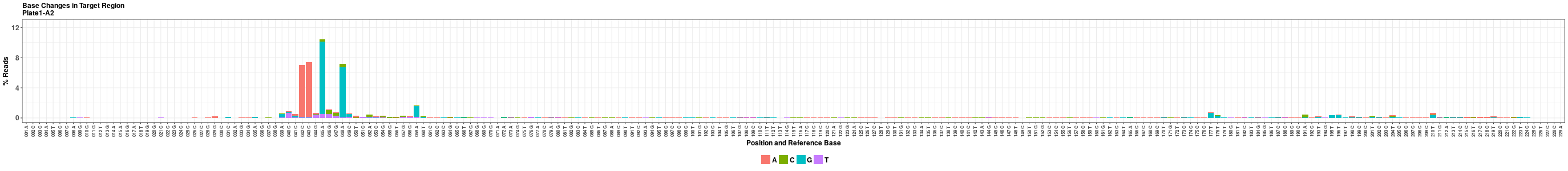

Supplement: Supplementary file 8 — Source Data [file 41467_2024_49400_MOESM8_ESM.zip › Source Data/Supplemental Figure 2/Supplemental Figure 2G/C2.png]

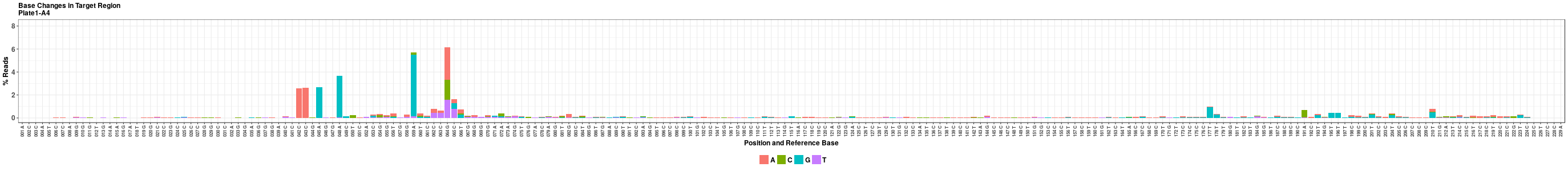

Supplement: Supplementary file 8 — Source Data [file 41467_2024_49400_MOESM8_ESM.zip › Source Data/Supplemental Figure 2/Supplemental Figure 2G/C4.png]

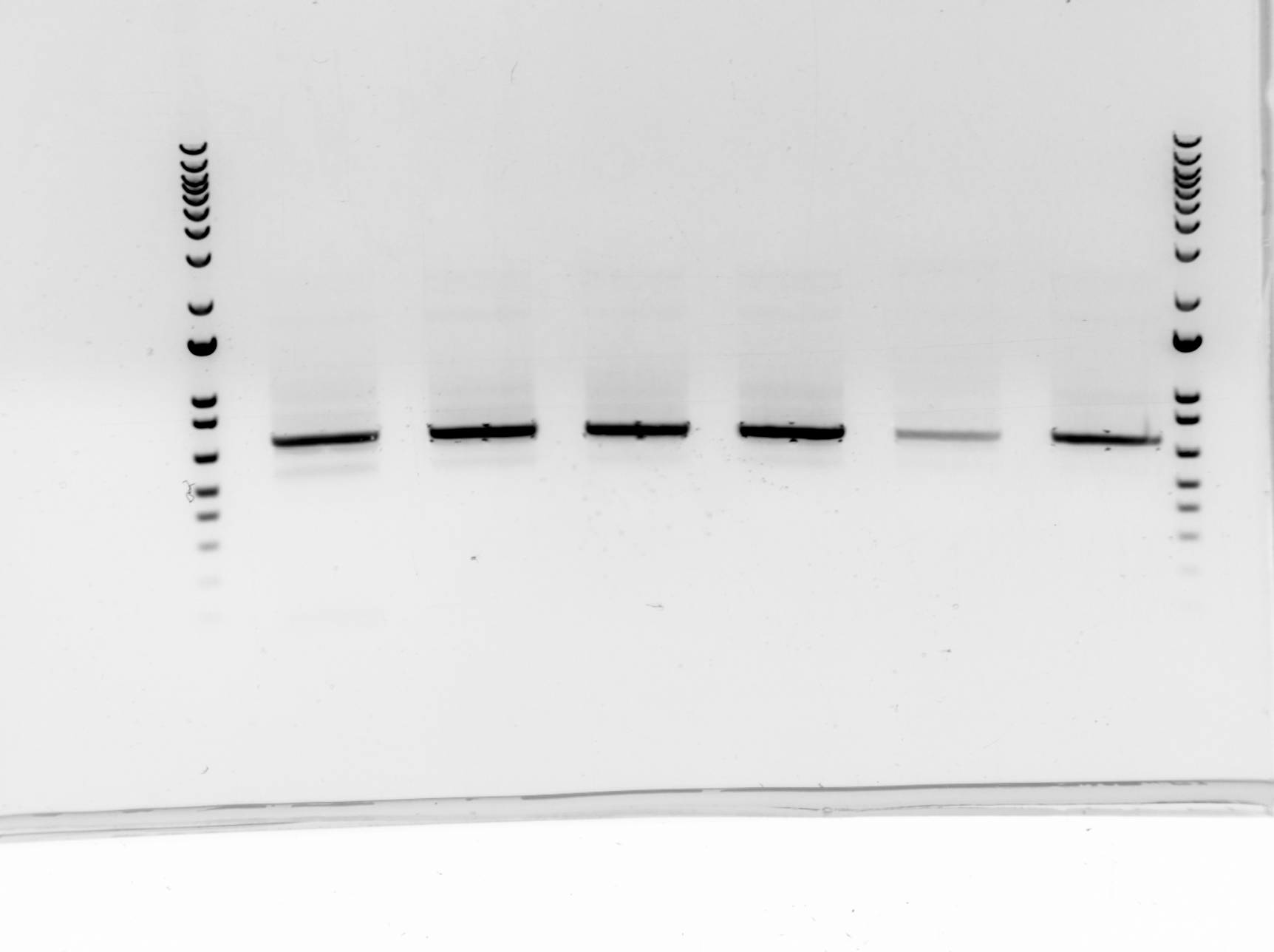

Supplement: Supplementary file 8 — Source Data [file 41467_2024_49400_MOESM8_ESM.zip › Source Data/Supplemental Figure 5/Supplemental Figure 5E/Supplemental Figure 5E.tif]
